# Total synthesis and complete configurational assignment of amphirionin-2†

**DOI:** 10.1039/d0sc06021f

**Published:** 2020-11-20

**Authors:** Shota Kato, Daichi Mizukami, Tomoya Sugai, Masashi Tsuda, Haruhiko Fuwa

**Affiliations:** Department of Applied Chemistry, Faculty of Science and Engineering, Chuo University 1-13-27 Kasuga, Bunkyo-ku Tokyo 112-8551 Japan hfuwa.50m@g.chuo-u.ac.jp; Center for Advanced Marine Core Research and Department of Agriculture and Marine Science, Kochi University Nankoku Kochi 783-8502 Japan

## Abstract

Amphirionin-2 is a linear polyketide metabolite that exhibits potent and selective cytotoxic activity against certain human cancer cell lines. We disclose herein the first total synthesis of amphirionin-2 and determination of its absolute configuration. Our synthesis featured an extensive use of cobalt-catalyzed Mukaiyama-type cyclization of γ-hydroxy olefins for stereoselective formation of all the tetrahydrofuran rings found in the natural product, and a late-stage Stille-type coupling for convergent assembly of the entire carbon backbone. Four candidate diastereomers of amphirionin-2 were synthesized in a unified, convergent manner, and their spectroscopic/chromatographic properties were compared with those of the authentic material. The present study culminated in the reassignment of the C5/C7 relative configuration, assignment of the C12/C18 relative configuration, and determination of the absolute configuration of amphirionin-2.

## Abstract 2

An extensive application of cobalt-catalyzed Mukaiyama-type cyclization of γ-hydroxy olefins and a late-stage Stille-type reaction enabled syntheses of four diastereomers of amphirionin-2 to establish its absolute configuration.

## Graphical abstract

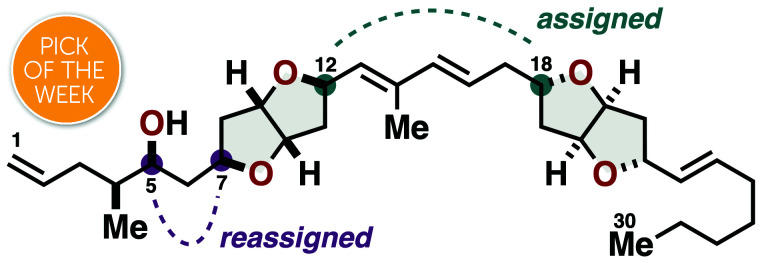

## Introduction

Marine polyketides are an important source of new chemotherapeutic agents for the treatment of cancer.^1^ As such, the structure, synthesis, and biological function of this class of natural products have gained significant interest from the chemical community.^2^ Marine polyketides are mostly non-crystalline, scarcely available substances from natural sources, and their complex structures are characterized mainly by NMR spectroscopic analysis. Integrated with quantum chemical calculations that enable the prediction of chemical shifts and ^3^*J*_H,H_ values,^3,4^ NMR-based structural assignment of stereochemically complex natural products has become more feasible than ever. Unfortunately, however, configurational assignment of remote stereogenic centers between which only negligible, if any, stereoelectronic and/or steric interactions exist, is still beyond the reach of NMR spectroscopic analysis and computational simulations.^5^ Orchestration of chemical synthesis, NMR and other spectroscopic techniques and, where appropriate, chromatographic analysis is indispensable for achieving complete configurational assignment of complex natural products.^6–8^

**Fig. 1:**
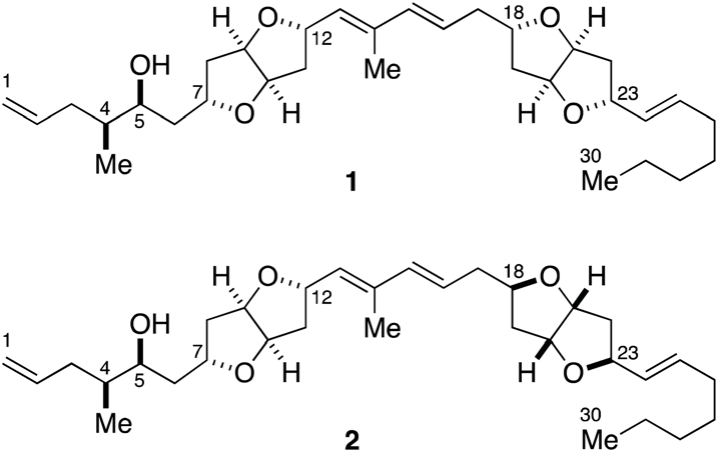
Putative structures 1 and 2 of amphirionin-2.

Amphirionin-2 (putative structures 1 and 2, Fig. 1) is a linear polyketide metabolite, isolated from cultured cells of the marine benthic dinoflagellate *Amphidinium* sp. KCA09051 strain.^9^ Amphirionin-2 exhibited potent cytotoxic activity against the human colon carcinoma Caco-2 cell line and the human non-small cell lung adenocarcinoma A549 cell line with IC_50_ values of 0.1 and 0.6 μg mL^−1^, respectively, whereas it showed only moderate cytotoxicity against the human cervix adenocarcinoma HeLa cell line (20% growth inhibition at 1 μg mL^−1^). Furthermore, amphirionin-2 displayed *in vivo* antitumor activity against murine tumor P388 cells (T/C 120% at 0.5 mg kg^−1^).

The gross structure of amphirionin-2 was determined on the basis of extensive 2D-NMR analyses. The relative configurations of two unique hexahydrofuro[3,2-*b*]furan moieties were individually characterized based on NOESY correlations. The relative configurations of C4/C5 and C5/C7 were deduced from conformational analyses based on *J* values and NOESY correlations. The absolute configuration of C5 was assigned on the basis of a modified Mosher analysis.^10^ However, the relative configuration between two remote stereogenic centers C12 and C18 could not be correlated by means of NMR-based structure analysis. Thus, the complete stereochemical assignment of amphirionin-2 needs to await its total synthesis.

Here we describe a unified, convergent total synthesis of amphirionin-2 and its three diastereomers for the first time to determine the absolute configuration of this natural product in an unambiguous manner.

## Results and discussion

**Scheme 1:**
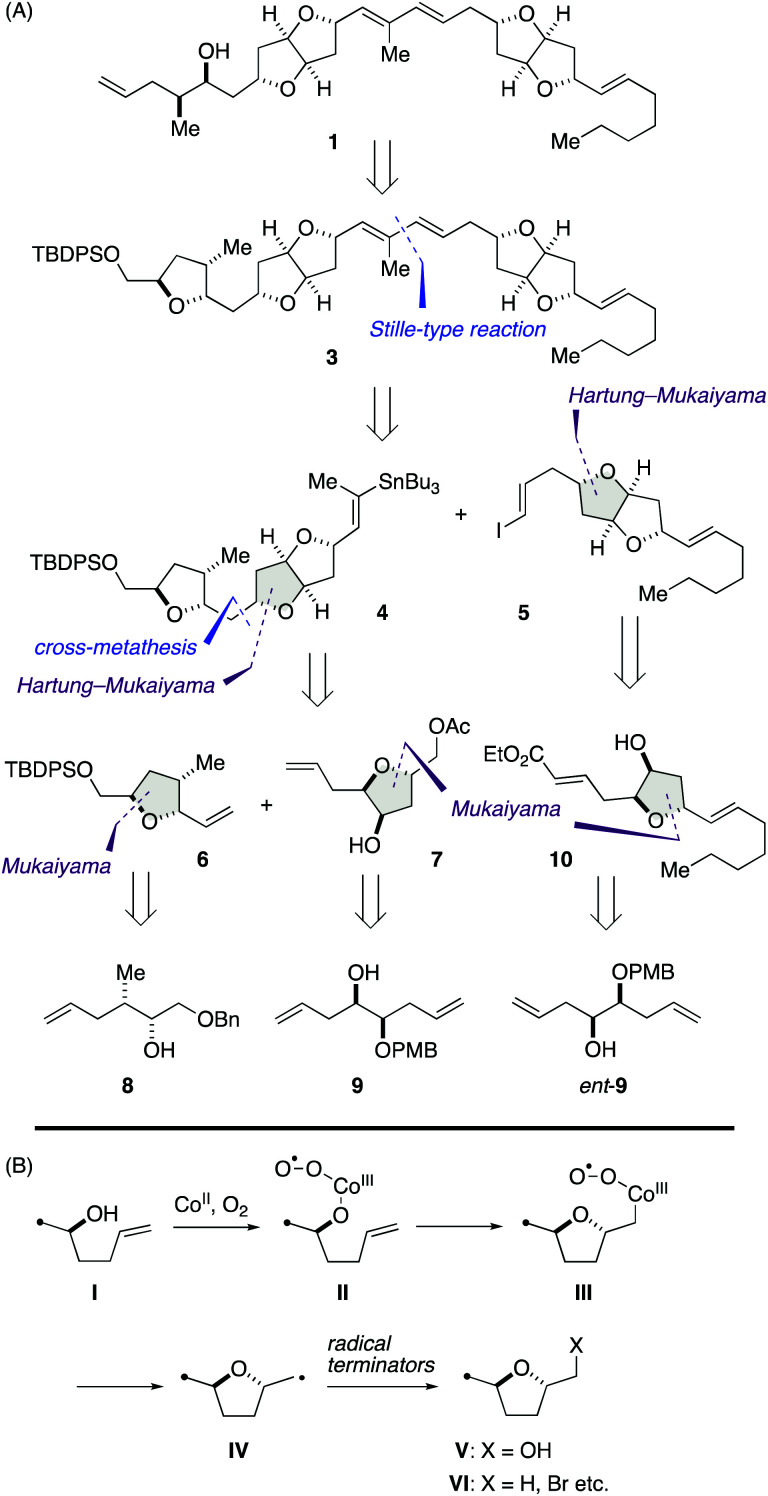
(A) Synthetic blueprint toward 1. (B) Mechanism of cobalt-catalyzed Mukaiyama and Hartung–Mukaiyama cyclizations.

Our synthetic blueprint toward 1 is summarized in Scheme 1A. The target structure 1 could be derived from 3 by a reductive cleavage of the left-end tetrahydrofuran ring. We envisaged that all the tetrahydrofuran rings found in 3 would be synthesizable by an extensive use of cobalt-catalyzed Mukaiyama-type cyclization of γ-hydroxy olefins. As shown in Scheme 1B, Inoki and Mukaiyama have reported that the reaction provides a diastereoselective access to 2,5-*trans*-2-hydroxymethyl tetrahydrofuran derivatives V from γ-hydroxy olefins I in the presence of appropriate cobalt(ii) chelate complexes under O_2_ atmosphere (hereafter referred to as Mukaiyama cyclization),^11^ and its mechanism involves radical intermediates II, III, and IV.^11,12^ Later, the Hartung group has demonstrated that the carbon-centered radical intermediate IV can be trapped with various radical terminators to deliver 2,5-*trans*-tetrahydrofuran derivatives VI (hereafter referred to as Hartung–Mukaiyama cyclization).^13^

We envisioned that 3 should be synthesized *via* a Stille-type reaction^14^ of vinylstannane 4 and iodoolefin 5. This late-stage fragment assembly would also enable an access to diastereomer 2 from 4 and *ent*-5 (latter not shown). Vinylstannane 4 would be accessible from olefins 6 and 7 through an olefin cross-metathesis^15^ and subsequent Hartung–Mukaiyama cyclization of the derived internal olefin. We were aware of the uncertainty of this retrosynthetic disconnection because Mukaiyama-type cyclization has mainly been applied to terminal olefins at early stages of total synthesis^16^ and its versatility toward internal olefins remained ambiguous. Moreover, Hartung–Mukaiyama cyclization has rarely been utilized in complex molecule synthesis.^16*g*^ Nevertheless, it appeared worthwhile to pursue this approach that allows for a convergent access to the tricyclic ether skeleton of 4. Olefins 6 and 7 were traced back to γ-hydroxy olefins 8 and 9 by considering Mukaiyama cyclization, respectively. Meanwhile, iodoolefin 5 would be derived from γ-hydroxy olefin 10*via* a Hartung–Mukaiyama cyclization. In turn, 10 would be available from γ-hydroxy olefin *ent*-9 by means of a Mukaiyama cyclization.

**Scheme 2:**
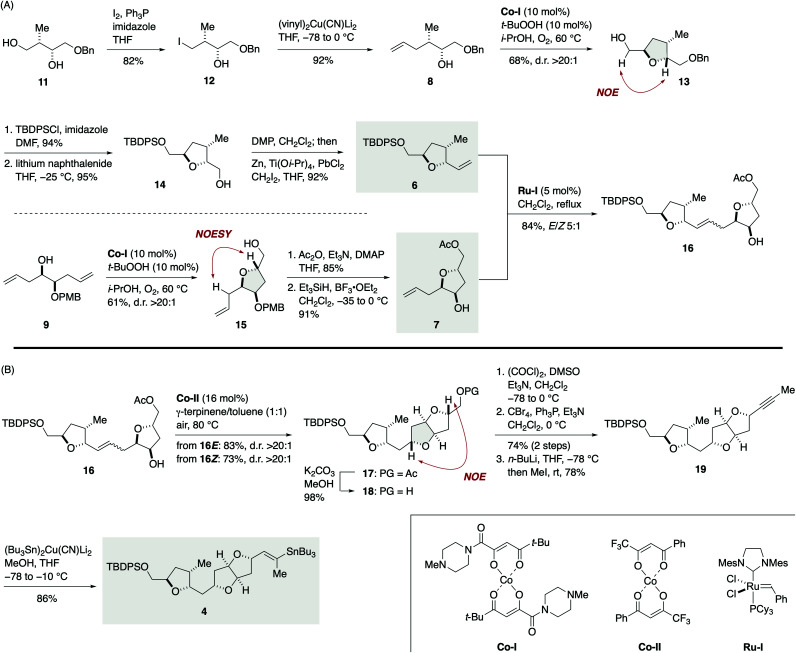
(A) Synthesis of olefin 16*via* olefin cross-metathesis of olefins 6 and 7. (B) Synthesis of vinylstannane 4.

The synthesis of vinylstannane 4 commenced with selective iodination of diol 11 (ref. 17) to give iodide 12 (82%), which was reacted with (vinyl)_2_Cu(CN)Li_2_ to deliver γ-hydroxy olefin 8 (92%, Scheme 2A). Mukaiyama cyclization of 8 (Co-I (10 mol%),^18^*t*-BuOOH (10 mol%), i-PrOH, 60 °C under O_2_) afforded 2,5-*trans*-tetrahydrofuran 13 in 68% yield with greater than 20 : 1 diastereoselectivity. The relative configuration of 13 was confirmed by an NOE experiment as shown. Silylation of 13 with TBDPSCl/imidazole (94%) and debenzylation with lithium naphthalenide^19^ gave alcohol 14 (95%), which was subjected to an oxidation/methylenation sequence (Dess–Martin periodinane (DMP), CH_2_Cl_2_; then Zn, Ti(Oi-Pr)_4_, PbCl_2_, CH_2_I_2_, THF)^20^ to deliver olefin 6 (92%) without isolation of the intermediate aldehyde.^21^ The TPAP oxidation/Wittig methylenation protocol reported by Ley *et al.*^22^ was less effective in this case presumably because of the sensitivity of the intermediate aldehyde toward basic conditions. Meanwhile, the coupling partner olefin 7 was synthesized from γ-hydroxy olefin 9.^23^ Mukaiyama cyclization of 9 (Co-I (10 mol%), *t*-BuOOH (10 mol%), i-PrOH, 60 °C under O_2_) afforded 2,5-*trans*-tetrahydrofuran 15 in 61% yield as a single diastereoisomer (d.r. >20 : 1). Acetylation (85%) followed by removal of the PMB group^24^ delivered olefin 7 (91%). Removal of the PMB group at this stage was crucial for the success of subsequent olefin cross-metathesis reaction. Olefin cross-metathesis of 6 and 7 was most efficiently achieved by the action of Ru-I (5 mol%)^25^ in dichloromethane under reflux to provide olefin 16 in 84% yield with *E*/*Z* 5 : 1 selectivity. These *E*/*Z* isomers were readily separable by flash column chromatography using silica gel. This fragment-assembly olefin cross-metathesis required extensive screening of ruthenium catalysts and reaction conditions (Tables S1 and S2, ESI†).

Now the stage was set for the crucial Hartung–Mukaiyama cyclization (Scheme 2B). To our delight, exposure of the major 16*E* isomer to Co-II (16 mol%)^13^ in 1 : 1 γ-terpinene/toluene at 80 °C under air furnished 2,5-*trans*-tetrahydrofuran 17 in 83% yield as a single stereoisomer (d.r. >20 : 1). The minor 16*Z* isomer could also be efficiently cyclized under the same conditions to deliver 17 in a comparable 73% yield (d.r. >20 : 1). Thus, the Hartung–Mukaiyama cyclization of 16*E*/Z proceeded cleanly regardless of their double bond configuration^26^ and provided the desired 17 in excellent yields. The relative configuration of 17 was established by an NOE correlation as shown. Removal of the acetyl group of 17 gave alcohol 18 (98%). Oxidation, dibromoolefination (74%, two steps), and subsequent alkynylation/methylation^27^ provided alkyne 19 (78%). Finally, stannylcupration^28^ using (Bu_3_Sn)_2_Cu(CN)Li_2_ (MeOH, THF, −78 to −10 °C) afforded vinylstannane 4 in 86% yield.

**Scheme 3:**
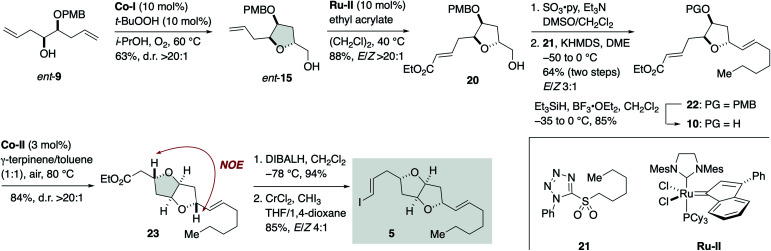
Synthesis of iodoolefin 5.

Meanwhile, the synthesis of iodoolefin 5 started from γ-hydroxy olefin *ent*-9 (Scheme 3). Mukaiyama cyclization of *ent*-9 by the action of Co-I (10 mol%) and *t*-BuOOH (10 mol%) in i-PrOH at 60 °C under O_2_ delivered 2,5-*trans*-tetrahydrofuran *ent*-15 in 63% yield as a single stereoisomer (d.r. >20 : 1). Olefin cross-metathesis of *ent*-15 with ethyl acrylate under the influence of Ru-II (10 mol%)^29^ gave α,β-unsaturated ester 20 in 88% yield (*E*/*Z* > 20 : 1). Oxidation and subsequent Julia–Kocienski olefination^30^ with sulfone 21 generated olefin 22 in 64% yield for the two steps with *E*/*Z* 3 : 1 selectivity. The undesired *Z* isomer was separated by flash column chromatography using silver nitrate-impregnated silica gel.^31^ Removal of the PMB group of 22 (Et_3_SiH, BF_3_·OEt_2_) afforded alcohol 10 (85%). Hartung–Mukaiyama cyclization of 10 by using Co-II (3 mol%) in 1 : 1 γ-terpinene/toluene at 80 °C under air provided 2,5-*trans*-tetrahydrofuran 23 (84%) with complete diastereoselectivity (d.r. >20 : 1). The stereochemical consequence was confirmed by an NOE experiment as shown. DIBALH reduction (94%) followed by Takai iodoolefination^32^ furnished iodoolefin 5 in 85% yield (*E*/*Z* 4 : 1). The *Z* isomer could be separated by flash column chromatography using silica gel. The enantiomer of 5, *i.e.*, *ent*-5, was prepared in the same manner from γ-hydroxy olefin 9 (see Scheme S5, ESI† for details).

**Scheme 4:**
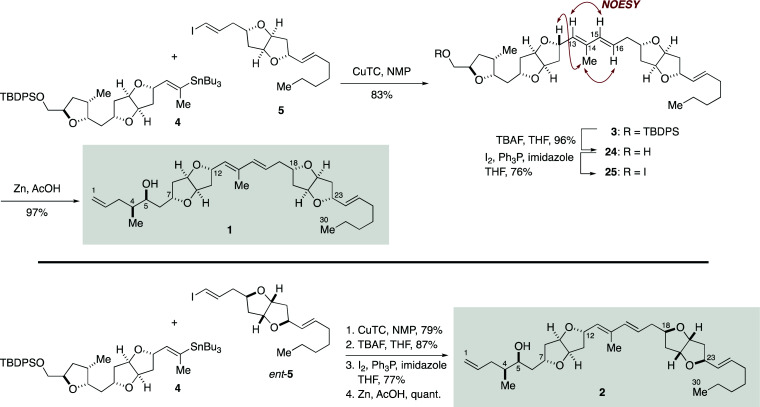
Total syntheses of putative structures 1 and 2 of amphirionin-2.

Completion of the total synthesis of 1 and 2 is illustrated in Scheme 4. Stille-type reaction of vinylstannane 4 (1 equiv.) with iodoolefin 5 (1.1 equiv.) was non-trivial and required optimization of reaction conditions. Initial experiments showed that the reaction under palladium catalysis ([Pd_2_(dba)_3_·CHCl_3_]/Ph_3_As with or without CuI) provided (*E*,*E*)-diene 3 in only low yield and resulted in significant side reactions, including isomerization of the C15–C16 double bond and homodimerization of 5 (Table S3, ESI†). It was eventually found that the reaction was best performed by using CuTC in NMP^33^ at room temperature, giving 3 in 83% yield with essentially no erosion of the configuration of the double bonds. The configuration of the diene moiety of 3 was confirmed to be *E*,*E* by NOESY correlations and a coupling constant (^3^*J*_H15,H16_ = 15.6 Hz). Cleavage of the TBDPS ether of 3 with TBAF delivered alcohol 24 in 96% yield. After iodination (I_2_, Ph_3_P, imidazole, 76%), the derived iodide 25 was exposed to excess zinc dust in acetic acid to furnish 1 (97%). The diastereomer 2 was synthesized from 4 and *ent*-5 in the same manner.

The ^1^H and ^13^C NMR spectra of 1 and 2 revealed that both compounds were not identical with natural amphirionin-2 (for assignment of ^1^H and ^13^C NMR signals, see Tables S4 and S5, ESI†). These results indicated the necessity of re-examination of the original structural assignment of the natural product. The ^1^H NMR chemical shifts of the C1–C12 moiety of synthetic 1 and 2 were significantly deviated from those of the corresponding moiety of the natural product, whereas the ^13^C NMR signals of synthetic 1 and 2 were similar to those of the authentic material and inconsistencies were limited to the C5–C10 moiety. Significantly, 1 and 2 were distinguishable from each other by ^1^H NMR analysis despite the C12 and C18 stereogenic centers being separated by six carbon–carbon bonds. Careful comparison of the ^1^H NMR spectra of 1 and 2 revealed subtle differences in signals assigned for H-11, H-16, H-17, and H-18 (Fig. S2, ESI†). With respect to these protons, the ^1^H NMR chemical shifts of 2 rather than 1 were in better agreement with those of natural amphirionin-2. It is known that stereoelectronic and/or steric interactions between two stereogenic centers separated by two or more methylene units are negligible by NMR spectroscopy.^5^ In the present case, the C13–C16 conjugated diene would be responsible for unusual long-range stereochemical interactions between the C12 and C18 stereogenic centers.^34^

We considered that the relative configuration of C12/C18 of natural amphirionin-2 might be same as that of synthetic 2, and that the relative configuration of C4/C5 and/or C5/C7 of the original stereochemical assignment should have been incorrectly assigned. Re-Examination of NOESY correlations and ^3^*J*_H,H_ values of natural amphirionin-2 suggested that the relative configuration of C5/C7 of the natural product might be opposite to that of the proposed structures 1 and 2 (Fig. S1, ESI†).

**Scheme 5:**
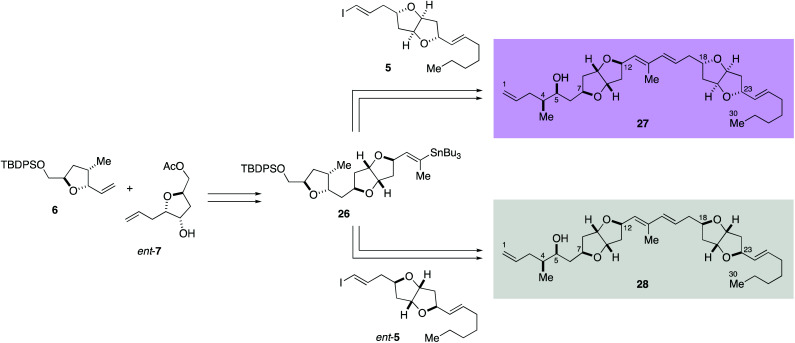
Syntheses of correct structure 27 of amphirionin-2 and its diastereomer 28.

**Fig. 2:**
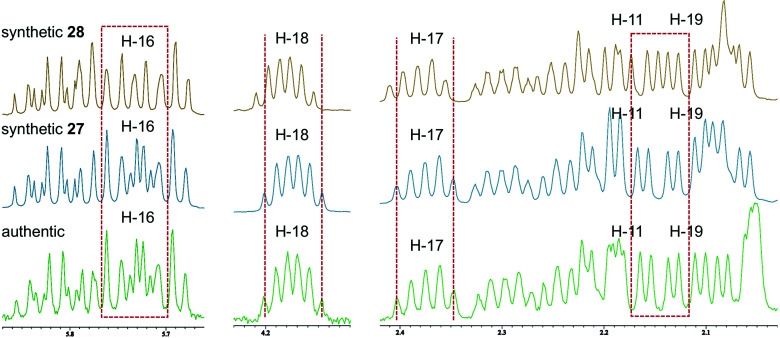
Comparison of ^1^H NMR spectra of 27, 28, and natural amphirionin-2. Inconsistency observed around 2.04–2.10 ppm is ascribable to 5-OH signal.

Accordingly, diastereomers 27 and 28 were synthesized from olefins 6 and *ent*-7*via* Stille-type coupling of vinylstannane 26 and iodoolefins 5/*ent*-5 (Scheme 5, details are provided in Schemes S7 and S8, ESI†). The ^1^H NMR spectra of synthetic 27 and 28 were almost identical with each other, as anticipated, but small but significant differences were observed in H-11, H-16, H-17, H-18, and H-19 signals (Fig. 2). The apparent inconsistency around 2.04–2.10 ppm in the ^1^H NMR spectra of synthetic 27 and 28 and natural amphirionin-2 was due to the 5-OH signal, which was firmly assigned on the basis of COSY experiments. The 5-OH signal disappeared upon addition of a drop of D_2_O (Fig. S3 and S4, ESI†). Therefore, we determined that the ^1^H NMR spectrum of 27 matched that of natural amphirionin-2. The ^13^C NMR spectra of synthetic 27 and 28 were completely indistinguishable from each other.

Moreover, chiral HPLC analysis (Chiralpak IB N-5: 4.6 mm I.D. × 250 mm; eluent: 10% i-PrOH/*n*-hexane; flow rate: 1.0 mL min^−1^; UV detection: 254 nm) demonstrated that the retention time of 27, 28, and natural amphirionin-2 was 8.9, 7.7, and 8.8 min, respectively (Fig. S5, ESI†). Co-Injection of synthetic 27 and authentic amphirionin-2 resulted in a single peak under the above analytical conditions. Thus, we concluded that the relative configuration of amphirionin-2 is same as that of 27. The specific rotation value of 27 ([*α*]_D_^20^ + 2.5 (*c* 0.18, CHCl_3_)) was in accordance with that of the authentic material ([*α*]_D_^20^ + 5 (*c* 0.8, CHCl_3_)^9^). Because of the small magnitude of the specific rotation value, it was further confirmed that the circular dichroism (CD) spectrum of natural amphirionin-2 was consistent with that of 27 (Fig. S6, ESI†). Accordingly, we established that the absolute configuration of amphirionin-2 is represented by the structure 27.

**Fig. 3:**
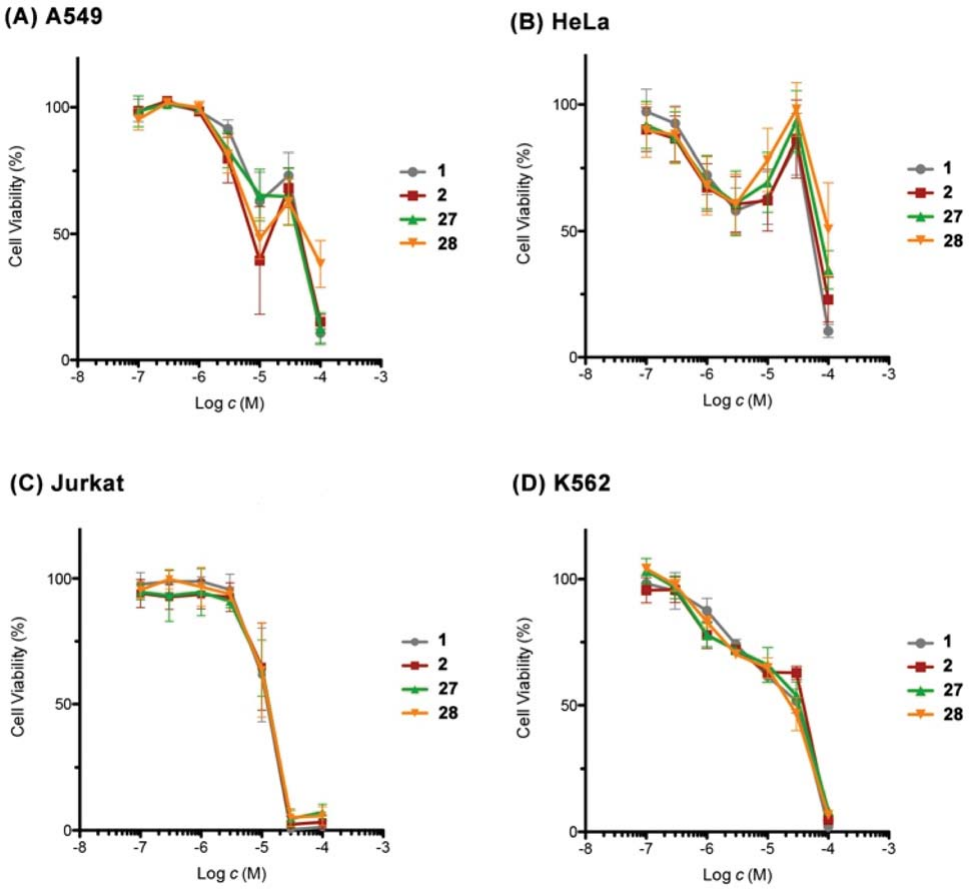
Cytotoxic activity of synthetic amphirionin-2 (27) and its diastereomers 1, 2, and 28. For an enlarged version of this figure, see Fig. S7, ESI.†

Finally, we evaluated the cytotoxic activity of our synthetic 1, 2, 27, and 28 against a small panel of human cancer cell lines, including the non-small cell lung adenocarcinoma A549, the cervix adenocarcinoma HeLa, the acute T cell leukemia Jurkat, and the chronic myelogenous leukemia K562 cell lines by WST-8 assay (Fig. 3). A549 cells showed biphasic response to synthetic 1, 2, 27, and 28. The viability of A549 cells decreased to 39–65% at 10 μM, increased to 63–73% at 30 μM, and then underwent to 11–38% at 100 μM. The sensitivity of A549 cells toward synthetic 27 was more moderate than that expected from the results reported in the isolation paper.^9^ This apparent discrepancy would be ascribable to the difference of the source of cells and/or experimental conditions, as a similar difference in potency was observed for the positive control doxorubicin: IC_50_ 0.6 μM (this work) *versus* IC_50_ 0.07 μM (ref. 9). A similar biphasic response was observed for HeLa cells upon exposure to our synthetic compounds; the cell viability declined to around 60% at 3 μM, restored to 86–98% at 30 μM, and dropped to 10–51% at 100 μM. These results suggested the possibility that our synthetic compounds would have at least two different mechanisms of action in A549 and HeLa cells. In contrast, Jurkat and K562 cells responded to our synthetic compounds in a dose-dependent manner. Jurkat cells were found to be more sensitive than K562 cells toward these compounds. Overall, our synthetic 1, 2, 27, and 28 showed cell line-dependent cytotoxic activity, whilst their stereochemistry did not have significant correlation with their cytotoxic potency.

## Conclusions

A unified total synthesis of four candidate stereoisomers 1, 2, 27, and 28 of amphirionin-2 was completed in 17 linear steps from diol 11 or 19 linear steps from benzyloxyacetaldehyde. The salient feature of the present work is an extensive use of cobalt-catalyzed Mukaiyama-type cyclization of γ-hydroxy olefins in stereocontrolled construction of all the tetrahydrofuran rings of amphirionin-2. The present study illuminates the versatility of Hartung–Mukaiyama cyclization of γ-hydroxy olefins, and also expands the reaction scope by demonstrating the synthesis of complex 2,5-*trans*-substituted tetrahydrofurans from internal olefins (*e.g.*, 16*E*/16*Z* → 17). A late-stage CuTC-mediated Stille-type reaction for convergent assembly of two hexahydrofuro[3,2-*b*]furan moieties is another important feature of our synthesis, which enabled rapid access to four candidate stereoisomers.

The ^1^H and ^13^C NMR spectroscopic data of originally assigned structures 1 and 2 showed non-identity of these compounds with natural amphirionin-2 and suggested the necessity to reassign the C5/C7 relative configuration. Eventually, the relative configuration of this natural product was fully established on the basis of the ^1^H and ^13^C NMR spectroscopic data and chiral HPLC chromatograms of 27 and 28 with authentic reference. The absolute configuration was determined by comparing the specific rotation value and CD spectroscopic data of 27 and 28 with those of the authentic material. Thus, it was concluded that the absolute configuration of amphirionin-2 is shown by the structure 27.

## Author contributions

H. F. conceived the project and directed the research. S. K. and D. M. executed synthetic experiments and collected compound characterization data. H. F., S. K., D. M., T. S. performed NMR analysis of key compounds. M. T. provided authentic amphirionin-2 and significant intellectual contribution for its configurational assignment. All authors composed the manuscript and the ESI.†

## Conflicts of interest

There are no conflicts to declare.

## Supplementary Material

SC-012-D0SC06021F-s001
