# Supplementary material for: Total synthesis and complete configurational assignment of amphirionin-2
Source: Chem Sci. 2020 Nov 20;12(3):872–9. doi: 10.1039/d0sc06021f (PMC8179035; doi:10.1039/d0sc06021f)
Supplement: SC-012-D0SC06021F-s001 [file SC-012-D0SC06021F-s001.pdf]

Electronic Supplementary Material (ESI) for Chemical Science.
This journal is © The Royal Society of Chemistry 2020

*Electronic Supplementary Information for*

# Total synthesis and complete configurational assignment of amphirionin-2

Shota Kato,[a] Daichi Mizukami,[a] Tomoya Sugai,[a] Masashi Tsuda[b] and Haruhiko Fuwa*[a]

[a]Department of Applied Chemistry, Faculty of Science and Engineering, Chuo University, 1-13-27 Kasuga, Bunkyo-ku, Tokyo 112-8551, Japan.

[b]Center for Advanced Marine Core Research and Department of Agriculture and Marine Science, Kochi University, Nankoku, Kochi 783-8502, Japan.

*Email: hfuwa.50m@g.chuo-u.ac.jp

**Table of Contents**


1. General remarks……………………………………………………………………...S3

2. Synthesis of olefins **6** and **7** (Scheme S1)……………………………………………S4

3. Optimization of fragment-assembly olefin cross-metathesis (Tables S1 and S2, Scheme S2)……S15

4. Synthesis of vinylstannane **4** (Scheme S3)…………………………………………..S18

5. Synthesis of iodoolefin **5** (Scheme S4)……………………………………………...S24

6. Synthesis of iodoolefin *ent*-**5** (Scheme S5)…………………………………………S32

7. Optimization of Stille-type reaction of vinylstannane **4** and iodoolefin **5** (Table S3)……………S34

8. Total synthesis of putative structures **1** and **2** of amphirionin-2 (Scheme S6)……………………S36

9. Synthesis of vinylstannane **26** (Scheme S7)…………………………………………S44

10. Total synthesis of correct structure **27** of amphirionin-2 and its diastereomer **28** (Scheme S8)….S50

11. Conformational analysis of the C1–C10 moiety of **1**, **2**, **27**, and **28** (Fig. S1)…………………S58

12. Comparison of $^{1}$H NMR data of **1**, **2**, **27**, **28**, and natural amphirionin-2 (Table S4)……………S59

13. Comparison of $^{13}$C NMR data of **1**, **2**, **27**, **28**, and natural amphirionin-2 (Table S5)……………S61

14. Comparison of $^{1}$H NMR spectra of **1**, **2**, and natural amphirionin-2 (Fig. S2)………………S63

15. Comparison of $^{1}$H NMR spectra of **27**, **28**, and natural amphirionin-2 (Fig. S3, S4)……………S64

16. Chiral HPLC analysis of **27**, **28**, and natural amphirionin-2 (Fig. S5)………………………...S66

17. CD spectra of **1**, **2**, **27**, **28**, and natural amphirionin-2 (Fig. S6)…………………………...S68

18. Cell culture experiments (Fig. S7)…………………………………………………..……...S69

19. Copies of $^{1}$H and $^{13}$C NMR spectra…………………………………………………S71

## 1. General remarks

All reactions sensitive to moisture and/or air were carried out under an atmosphere of argon in dry solvents under anhydrous conditions using oven-dried glassware unless otherwise noted. Where appropriate, solvents were degassed by the freeze–thaw technique immediately prior to use. Anhydrous dichloromethane ($CH_2Cl_2$), tetrahydrofuran (THF), and toluene were purchased from Kanto Chemical Co. Inc. and used directly. Acetonitrile, boron trifluoride diethyl etherate ($BF_3$•$OEt_2$), 1,2-dichloroethane, dimethylsulfoxide (DMSO), 2,6-lutidine, methanol, pyridine, and triethylamine were distilled from calcium hydride under an atmosphere of argon. DMF was distilled over $MgSO_4$ under reduced pressure. All other chemicals were purchased at highest commercial grade and used directly. Analytical thin-layer chromatography was performed using E. Merck silica gel 60 $F_{254}$ plates (0.25-mm thickness). Flash column chromatography was carried out using Kanto Chemical silica gel 60N (40–100 mesh, spherical, neutral), Fuji Silysia silica gel PSQ100B or Fuji Silysia silica gel BW-300. Optical rotations were recorded on a JASCO P-1020 digital polarimeter. IR spectra were recorded on a JASCO FT/IR-4100 spectrometer. $^1H$ and $^{13}C$ NMR spectra were recorded on a JEOL ECA-500 spectrometer, a JEOL ECZ-500R spectrometer or a Varian Mercury 400 spectrometer, and chemical shift values are reported in ppm (δ) downfield from tetramethylsilane with reference to internal residual solvent [$^1H$ NMR, $C\mathit{H}Cl_3$ (7.24), $C_6\mathit{H}D_5$ (7.15); $^{13}C$ NMR, $CDCl_3$ (77.0), $C_6D_6$ (128.0)] unless otherwise noted. Coupling constants (*J*) are reported in Hertz (Hz). The following abbreviations were used to designate the multiplicities: s = singlet; d = doublet; t = triplet; q = quartet; m = multiplet; br = broad. High-resolution mass spectra were measured on a JEOL JMS-T100LC AccuTOF spectrometer. CD spectra were recorded on a JASCO J-820 spectrometer.

## 2. Synthesis of olefins 6 and 7

### 2-1. Synthesis of olefin **6**

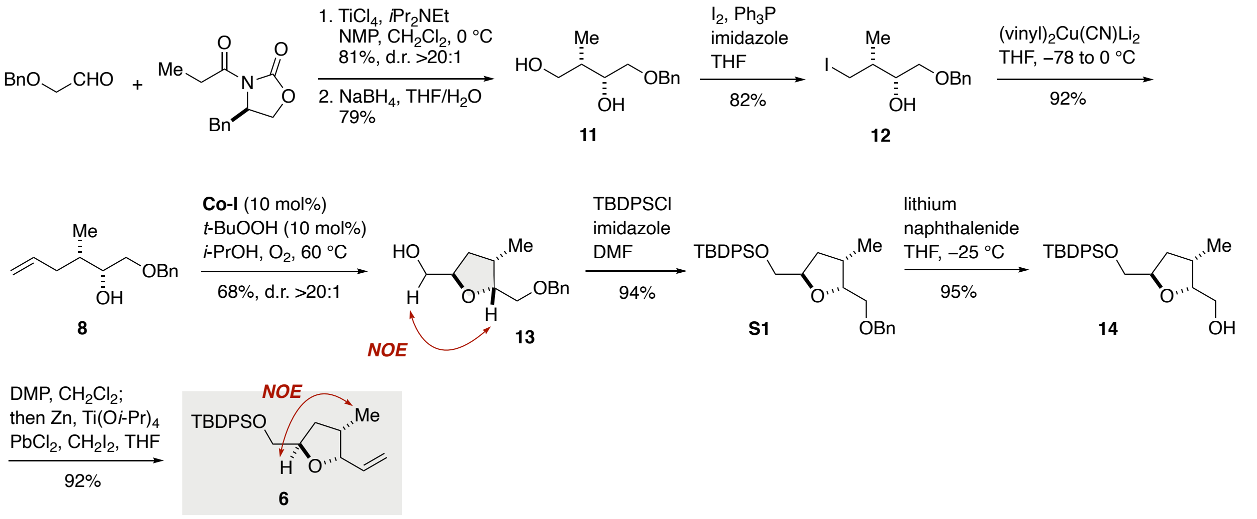


### 2-2. Synthesis of olefin **7**

D-DET route for diol **S6**

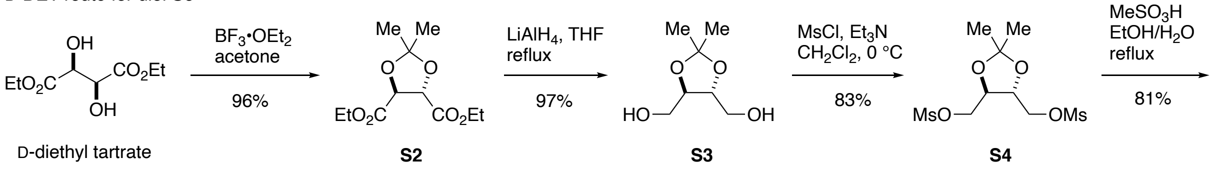


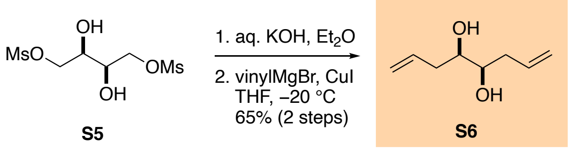


**S6**: J. Wang, B. L. Pagenkopf, *Org. Lett.* **2007**, *9*, 3703–3706;
M. A. Robbins, P. N. Devine, T. Oh, *Org. Synth.* **1999**, *76*, 101–105.

---

Sharpless AD route for diol **S6**

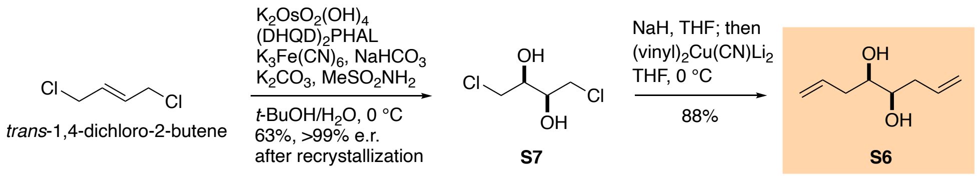


**S7**: K. P. M. Vanhessche, Z.-M. Wang, K. B. Sharpless
*Tetrahedron Lett.* **1994**, *35*, 3469–3472.

---

Elaboration of olefin **7** from **S6**

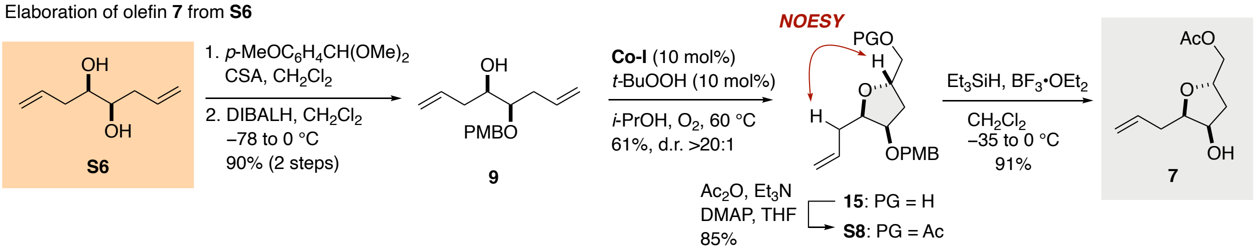


**Scheme S1** Synthesis of olefins **6** and **7**.

## 2-1. Synthesis of olefin 6

**Diol 11.** To a solution of (*R*)-(−)-4-benzyl-3-propionyl-2-oxazolidinone (2.55 g, 10.9 mmol) in $CH_2Cl_2$ (110 mL) at 0 °C was added $TiCl_4$ (1.25 mL, 11.4 mmol), and the resultant mixture was stirred at 0 °C for 15 min. To the reaction mixture was added *i*-$Pr_2NEt$ (2.04 mL, 12.0 mmol), and the resultant mixture was stirred at 0 °C for 40 min. To the reaction mixture was added NMP (2.10 mL, 21.8 mmol), and the resultant mixture was stirred at 0 °C for 10 min. To the reaction mixture was added a solution of (benzyloxy)acetaldehyde (1.97 g, 13.1 mmol) in $CH_2Cl_2$ (20.0 mL + 5.00 mL rinse), and the resultant mixture was stirred at 0 °C for 2 h. The reaction was quenched with saturated aqueous $NH_4Cl$ solution at 0 °C. The resultant mixture was extracted with EtOAc, and the organic layer was washed with $H_2O$ and brine, dried ($Na_2SO_4$), filtered, and concentrated under reduced pressure. Purification of the residue by flash column chromatography (silica gel, 10 to 40% EtOAc/hexanes) gave an aldol product (3.40 g, 81%, d.r. >20:1) as a yellow oil: $^1$H NMR (400 MHz, $CDCl_3$) δ 7.36–7.24 (m, 8H), 7.19–7.17 (m, 2H), 4.58 (m, 1H), 4.53 (s, 2H), 4.18–4.05 (m, 3H), 3.93 (dq, *J* = 6.8, 4.8 Hz, 1H), 3.53 (d, *J* = 6.0 Hz, 2H), 3.22 (dd, *J* = 13.2, 3.2 Hz, 1H), 2.75 (dd, *J* = 13.2, 9.6 Hz, 1H), 1.28 (d, *J* = 6.8 Hz, 1H), one proton missing due to H/D exchange.

To a solution of the above alcohol (3.88 g, 10.1 mmol) in THF (80.0 mL) at 0 °C was added a solution of $NaBH_4$ (1.54 g, 40.7 mmol) in $H_2O$ (20.0 mL), and the resultant mixture was stirred at room temperature for 2 h. The reaction was quenched with saturated aqueous potassium sodium tartrate solution at 0 °C. The resultant mixture was diluted with EtOAc and stirred at room temperature for 12 h. The resultant mixture was repeatedly extracted with EtOAc and washed with brine. The organic layers were combined, dried ($MgSO_4$), filtered, and concentrated under reduced pressure. Purification of the residue by flash column chromatography (silica gel, 40% EtOAc/toluene) gave diol **11** (1.67 g, 79%) as a colorless oil: $[\alpha]_D^{21}$ −1.4 (*c* 0.85, $CHCl_3$). The $^1$H and $^{13}$C NMR data of this material matched those reported previously.[1] However, previous literatures reported inconsistent

[1] (a) B. G. Lawhorn, S. B. Boga, S. E. Wolkenberg, D. A. Colby, C.-M. Gauss, M. R. Swingle, L. Amable, R. E. Honkanen and D. L. Boger, *J. Am. Chem. Soc.*, 2006, **128**, 16720–16732; (b) A. K. Ghosh and J.-H. Kim, *Org. Lett.*, 2003, **5**, 1063–1066; (c) B. Ganganna, P. Srihari and J. S. Yadav, *Tetrahedron Lett.*, 2017, **58**, 2685–2689.

specific rotation values for **11**: lit.[1a] $[\alpha]_D^{25}$ +5.7 (*c* 1.3, $CHCl_3$); lit.[1b] $[\alpha]_D^{23}$ −2.8 (*c* 0.85, $CHCl_3$); lit.[1c] $[\alpha]_D^{24}$ +4.8 (*c* 1.2, $CHCl_3$). Accordingly, the absolute configuration of **11** was confirmed by a modified Mosher analysis at a later stage of the synthesis.

**Iodide 12.** To a solution of diol **11** (582.8 mg, 2.772 mmol) in THF (50 mL) were added imidazole (575.9 mg, 8.459 mmol), $Ph_3P$ (754.2 mg, 2.875 mmol), and $I_2$ (729.6 mg, 2.875 mmol), and the resultant mixture was stirred at room temperature for 30 min. The reaction was quenched with saturated aqueous $Na_2SO_3$ solution at 0 °C. The resultant mixture was extracted with EtOAc, and the organic layer was washed with $H_2O$ and brine, dried ($Na_2SO_4$), filtered, and concentrated under reduced pressure. Purification of the residue by flash column chromatography (silica gel, 10 to 20% EtOAc/hexanes) gave iodide **12** (725.8 mg, 82%) as a colorless oil: $[\alpha]_D^{24}$ –8.8 (*c* 1.01, $CHCl_3$); IR (film) 3444, 2865, 1496, 1454, 1200, 1105, 738 $cm^{-1}$; $^1H$ NMR (400 MHz, $CDCl_3$) δ 7.37–7.27 (m, 5H), 4.54 (s, 2H), 3.88 (ddd, *J* = 7.6, 4.4, 3.6 Hz, 1H), 3.50 (dd, *J* = 9.2, 3.6 Hz, 1H), 3.44 (dd, *J* = 9.2, 7.6 Hz, 1H), 3.31 (dd, *J* = 10.0, 6.4 Hz, 1H), 3.08 (dd, *J* = 10.0, 6.4 Hz, 1H), 2.23 (br s, 1H), 1.77 (qddd, *J* = 6.4, 6.4, 6.4, 4.4 Hz, 1H), 1.03 (d, *J* = 6.4 Hz, 3H); $^{13}C$ NMR (100 MHz, $CDCl_3$) δ 137.7, 128.5 (2C), 127.9, 127.7 (2C), 73.4, 72.4 (2C), 37.9, 15.3, 12.1; HRMS (ESI) *m/z* calcd for $C_{12}H_{18}O_2I^+$ $[(M + H)^+]$ 321.0346, found 321.0352.

**Olefin 8.** To a solution of tetra(vinyl)tin (0.670 mL, 3.78 mmol) in THF (5.00 mL) at −78 °C was added MeLi (1.42 M solution in cyclopentyl methyl ether, 8.80 mL, 12.5 mmol), and the resultant solution was stirred at −78 °C for 2 h and then warmed to room temperature over a period of 10 min. The resultant solution was transferred to a suspension of CuCN (557.9 mg, 6.229 mmol) in THF (5.00 mL) at −78 °C (rinsed with 3.00 mL of THF), and the resultant mixture was stirred at −78 °C for 10 min and then at 0 °C for 20 min. To the reaction mixture at −78 °C was added a solution of iodide **12** (666.4 mg, 2.082 mmol) in THF (5.00 mL + 3.00 mL rinse), and the resultant mixture was stirred at 0 °C for 1 h. The reaction was quenched with a 9:1 (v/v) mixture of saturated aqueous $NH_4Cl$

solution/28% $NH_4OH$ solution at 0 °C. The resultant mixture was extracted with *t*-BuOMe, and the organic layer was washed with $H_2O$ and brine, dried ($Na_2SO_4$), filtered, and concentrated under reduced pressure. Purification of the residue by flash column chromatography (silica gel, 10 to 20% *t*-BuOMe/hexanes) gave olefin **8** (421.5 mg, 92%) as a colorless oil: $[\alpha]_D^{24}$ −11.3 (*c* 0.91, $CHCl_3$); IR (film) 3460, 2908, 2872, 1639, 1454, 1100, 994, 912 $cm^{-1}$; $^1H$ NMR (400 MHz, $CDCl_3$) δ 7.37–7.27 (m, 5H), 5.75 (dddd, *J* = 16.8, 10.0, 7.2, 6.8 Hz, 1H), 5.04–4.97 (m, 2H), 4.54 (s, 2H), 3.74 (ddd, *J* = 8.0, 4.8, 3.2 Hz, 1H), 3.51 (dd, *J* = 9.2, 3.2 Hz, 1H), 3.42 (dd, *J* = 9.2, 8.0 Hz, 1H), 2.22 (ddddd, *J* = 13.6, 6.8, 6.8, 1.6, 1.6 Hz, 1H), 2.18 (br s, 1H), 1.91 (ddddd, *J* = 13.6, 8.0, 8.0, 1.6, 1.6 Hz, 1H), 1.66 (m, 1H), 0.91 (d, *J* = 6.8 Hz, 3H); $^{13}C$ NMR (100 MHz, $CDCl_3$) δ 137.9, 137.0, 128.4 (2C), 127.75, 127.70 (2C), 116.2, 73.3, 72.89, 72.87, 37.7, 35.4, 14.2; HRMS (ESI) *m/z* calcd for $C_{14}H_{20}O_2Na^+$ [(M + Na)$^+$] 243.1356, found 243.1356.

The absolute configuration of **8** was confirmed by a modified Mosher analysis, as shown below:

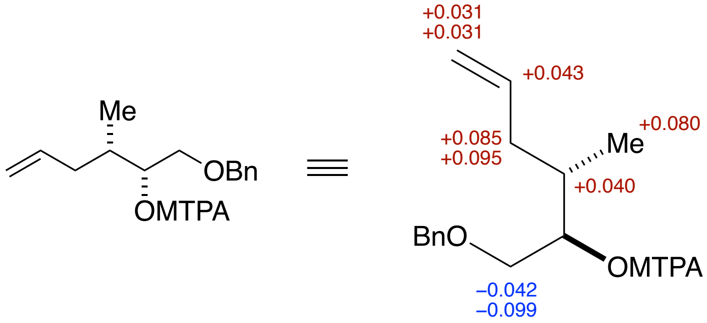


**Alcohol 13.** To a solution of olefin **8** (0.46 g, 2.1 mmol) in 2-propanol (20.0 mL) were added **Co-I** (121.0 mg, 0.2139 mmol) and *t*-BuOOH (6.43 M solution in isooctane, 0.032 mL, 0.21 mmol), and the resultant mixture was stirred at 60 °C under an atmosphere of $O_2$ (balloon) for 3.5 h. The resultant mixture was cooled to room temperature and concentrated under reduced pressure. Purification of the residue by flash column chromatography (silica gel, 40 to 60% EtOAc/hexanes) gave alcohol **13** (411.1 mg, 68%, d.r. >20:1) as a colorless oil: $[\alpha]_D^{23}$ −4.1 (*c* 0.94, $CHCl_3$); IR (film) 3432, 2927, 2874, 1454, 1096 $cm^{-1}$; $^1H$ NMR (400 MHz, $CDCl_3$) δ 7.34–7.24 (m, 5H), 4.59 (d, *J* = 12.0 Hz, 1H), 4.49 (d, *J* = 12.0 Hz, 1H), 4.22 (dddd, *J* = 7.2, 7.2, 6.0, 3.2 Hz, 1H), 4.15 (dt, *J* = 5.6, 5.6 Hz, 1H), 3.60 (dd, *J* = 12.0, 3.2 Hz, 1H), 3.49 (d, *J* = 5.6 Hz, 2H), 3.44 (dd, *J* = 12.0, 6.0 Hz, 1H), 2.39–2.32 (m, 2H), 1.85

(ddd, $J$ = 12.4, 7.2, 7.2 Hz, 1H), 1.65 (ddd, $J$ = 12.4, 7.2, 4.8 Hz, 1H), 0.92 (d, $J$ = 7.2 Hz, 3H); $^{13}$C NMR (100 MHz, $CDCl_3$) δ 138.1, 128.3 (2C), 127.6 (2C), 127.4, 80.2, 78.2, 73.4, 70.4, 65.3, 35.4, 35.3, 14.0; HRMS (ESI) *m*/*z* calcd for $C_{14}H_{20}O_3Na^+$ [(M + Na)$^+$] 259.1305, found 259.1306.

**Silyl ether S1.** To a solution of alcohol **13** (191.6 mg, 0.8108 mmol) in DMF (4.00 mL) were added imidazole (133.6 mg, 1.962 mmol) and TBDPSCl (0.250 mL, 0.964 mmol), and the resultant mixture was stirred at room temperature for 4 h 40 min. To the reaction mixture was added TBDPSCl (0.060 mL, 0.23 mmol), and the resultant mixture was stirred at room temperature for 1 h. The reaction mixture was diluted with *t*-BuOMe, washed with $H_2O$ and brine, dried ($Na_2SO_4$), filtered, and concentrated under reduced pressure. Purification of the residue by flash column chromatography (silica gel, 5% *t*-BuOMe/hexanes) gave silyl ether **S1** (362.0 mg, 94%) as a colorless oil: $[\alpha]_D^{25}$ +0.57 (*c* 0.24, $CHCl_3$); IR (film) 2929, 2856, 1427, 1112, 700 cm$^{-1}$; $^1$H NMR (400 MHz, $CDCl_3$) δ 7.68–7.64 (m, 4H), 7.39–7.31 (m, 11H), 4.59 (d, $J$ = 12.4 Hz, 1H), 4.50 (d, $J$ = 12.4 Hz, 1H), 4.22 (m, 1H), 4.11 (ddd, $J$ = 5.6, 5.6, 5.6 Hz, 1H), 3.65 (dd, $J$ = 10.4, 4.8 Hz, 1H), 3.60 (dd, $J$ = 10.4, 5.2 Hz, 1H), 3.49 (d, $J$ = 5.6 Hz, 2H), 2.38 (m, 1H), 2.03 (ddd, $J$ = 12.4, 6.8, 6.8 Hz, 1H), 1.70 (ddd, $J$ = 12.4, 7.2, 4.8 Hz, 1H), 1.03 (s, 9H), 0.93 (d, $J$ = 6.8 Hz, 3H); $^{13}$C NMR (100 MHz, $CDCl_3$) δ 138.4, 135.61 (2C), 135.60 (2C), 133.64, 133.62, 129.4, 129.52, 128.3 (2C), 127.65 (2C), 127.59 (4C), 127.5, 80.2, 78.0, 73.4, 70.4, 66.6, 36.1, 35.0, 26.8 (3C), 19.2, 14.1; HRMS (ESI) *m*/*z* calcd for $C_{30}H_{38}O_3SiNa^+$ [(M + Na)$^+$] 497.2482, found 497.2485.

**Alcohol 14.** To a solution of naphthalene (518.0 mg, 4.042 mmol) in THF (10.0 mL) was added lithium (22.0 mg, 3.17 mmol), and the resultant mixture was stirred at room temperature for 2 h. The lithium naphthalenide solution thus obtained was used in the following reaction.

To a solution of silyl ether **S1** (146.6 mg, 0.3088 mmol) in THF (2.00 mL) at −25 °C was added dropwise the above lithium naphthalenide solution until dark green color persisted, and the resultant solution was stirred at −25 °C for 40 min. The reaction was quenched with saturated aqueous $NH_4Cl$

solution. The resultant mixture was diluted with *t*-BuOMe, washed with $H_2O$ and brine, dried ($Na_2SO_4$), filtered, and concentrated under reduced pressure. Purification of the residue by flash column chromatography (silica gel, 20–30% EtOAc/hexanes) gave alcohol **14** (113.1 mg, 95%) as a colorless oil: $[\alpha]_D^{25}$ −9.8 (*c* 0.21, $CHCl_3$); IR (film) 3424, 2930, 2857, 1427, 1112, 1040, 702 $cm^{-1}$; $^1H$ NMR (400 MHz, $CDCl_3$) δ 7.68–7.64 (m, 4H), 7.43–7.34 (m, 6H), 4.20 (ddt, *J* = 6.8, 5.2, 4.8 Hz, 1H), 4.01 (dt, *J* = 7.2, 6.8 Hz, 1H), 3.61 (d, *J* = 4.8 Hz, 2H), 3.54 (d, *J* = 6.8 Hz, 2H), 2.38 (dddq, *J* = 7.2, 7.2, 7.2, 7.2 Hz, 1H), 2.03 (ddd, *J* = 12.4, 7.2, 5.2 Hz, 1H), 1.66 (ddd, *J* =12.4, 7.2, 6.8 Hz, 1H), 1.03 (s, 9H), 0.94 (d, *J* = 7.2 Hz, 3H), one proton missing due to H/D exchange; $^{13}C$ NMR (100 MHz, $CDCl_3$) δ 135.61 (2C), 135.59 (2C), 133.60, 133.55, 129.6 (2C), 127.6 (4C), 81.7, 78.0, 66.6, 62.6, 36.0, 34.4, 26.8 (3C), 19.2, 13.8; HRMS (ESI) *m*/*z* calcd for $C_{23}H_{32}O_3SiNa^+$ $[(M + Na)^+]$ 407.2013, found: 407.2011.

**Olefin 6.** To a solution of alcohol **14** (32.6 mg, 0.0857 mmol) in $CH_2Cl_2$ (0.90 mL) was added Dess-Martin periodinane (52.7 mg, 0.124 mmol), and the resultant mixture was stirred at room temperature for 4 h. In a separate flask, activated zinc powder (423.9 mg, 6.484 mmol) and $PbCl_2$ (42.7 mg, 0.154 mmol) was suspended in THF (5.00 mL). To this suspension was added $CH_2I_2$ (0.240 mL, 2.97 mmol), and the resultant mixture was stirred at room temperature for 1 h. To the mixture at 0 °C was added Ti(O*i*-Pr)$_4$ (0.230 mL, 0.777 mmol), and the resultant mixture was stirred at room temperature for 1 h. To this mixture at 0 °C was added the above aldehyde solution (rinsed with $CH_2Cl_2$ (0.70 mL)), and the resultant mixture was stirred at room temperature for 30 min. The reaction was quenched with saturated aqueous $NaHCO_3$ solution at 0 °C, and the resultant mixture was stirred at room temperature for 30 min before filtered through a pad of Celite. The filtrate was diluted with EtOAc, washed with $H_2O$ and brine, dried ($Na_2SO_4$), filtered, and concentrated under reduced pressure. Purification of the residue by flash column chromatography (silica gel, 2% *t*-BuOMe/hexanes) gave olefin **6** (30.1 mg, 92%) as a colorless oil: $[\alpha]_D^{24}$ −16.6 (*c* 0.34, $CHCl_3$); IR (film) 2960, 2930, 2857, 1427, 1113, 702 $cm^{-1}$; $^1H$ NMR (400 MHz, $CDCl_3$) δ 7.69–7.65 (m, 4H), 7.42–7.33 (m, 6H), 5.80 (ddd, *J* = 16.8, 10.4,

6.8 Hz, 1H), 5.23 (ddd, *J* = 16.8, 2.0, 1.2 Hz, 1H), 5.15 (ddd, *J* = 10.4, 2.0, 1.2 Hz, 1H), 4.39 (dddd, *J* = 6.8, 6.8, 1.2, 1.2 Hz, 1H), 4.23 (dddd, *J* = 7.6, 5.2, 5.2, 5.2 Hz, 1H), 3.65 (dd, *J* = 10.4, 5.2 Hz, 1H), 3.61 (dd, *J* = 10.4, 5.2 Hz, 1H), 2.36 (dddq, *J* = 6.8, 6.8, 6.8, 6.8 Hz, 1H), 2.05 (ddd, *J* = 12.4, 6.8, 5.2 Hz, 1H), 1.68 (ddd, *J* = 12.4, 7.6, 6.8 Hz, 1H), 1.04 (s, 9H), 0.91 (d, *J* = 6.8 Hz, 3H); $^{13}$C NMR (100 MHz, $CDCl_3$) δ 136.2, 135.6 (4C), 133.6 (2C), 129.55, 129.53, 127.6 (4C), 116.1, 83.1, 77.9, 66.6, 36.8, 35.6, 26.8 (3C), 19.2, 14.7; HRMS (ESI) *m*/*z* calcd for $C_{24}H_{32}O_2SiNa^+$ [(M + Na)$^+$] 403.2064, found: 403.2063.

**2-2. Synthesis of olefin 7**

Two approaches for known diol **S6** were examined as shown in Scheme S1. According to the procedure described by Robbins et al., **S6** was prepared in six steps using D-diethyl tartrate as a starting material. Diol **S6** was also accessible in two steps with high enantiomeric purity (>99% e.r.) by Sharpless asymmetric dihydroxylation of *trans*-1,4-dichloro-2-butene, followed by an epoxidation/vinylcuprate addition.

**Diol S6.** To a suspension of NaH (60% in mineral oil, 160.0 mg, 4.000 mmol) in THF (4.00 mL) at 0 °C was added a solution of diol **S7**[2] (>99% e.r., 243.6 mg, 1.542 mmol) in THF (3.00 mL + 1.00 mL rinse), and the resultant mixture was stirred at room temperature for 1.5 h to give a THF solution of diepoxide.

To a solution of tetra(vinyl)tin (0.520 mL, 2.93 mmol) in THF (5.00 mL) at −78 °C was added MeLi (1.42 M solution in cyclopentyl methyl ether, 6.90 mL, 9.80 mmol), and the resultant solution was stirred at −78 °C for 2 h and then at room temperature for 40 min. The resultant solution of vinyl lithium was transferred to a suspension of CuCN (414.2 mg, 4.626 mmol) in THF (4.50 mL) at −78 °C (rinsed with 2.00 mL of THF), and the resultant mixture was stirred at −78 °C for 10 min and then at

[2] K. P. M. Vanhessche, Z.-M. Wang and K. B. Sharpless, *Tetrahedron Lett.*, 1994, **35**, 3469–3472.

0 °C for 20 min. To the reaction mixture was added the above diepoxide solution (rinsed with 2.00 mL of THF), and the resultant mixture was stirred at 0 °C for 1 h. The reaction was quenched with 9:1 (v/v) mixture of saturated aqueous $NH_4Cl$ solution/28% $NH_4OH$ solution. The resultant mixture was stirred at room temperature for 20 min and then extracted with EtOAc. The organic layer was washed with $H_2O$ and brine, dried ($Na_2SO_4$), filtered, and concentrated under reduced pressure. Purification of the residue by flash column chromatography (silica gel, 20 to 30% EtOAc/hexanes) gave diol **S6** (192.4 mg, 88%) as colorless crystals: $[\alpha]_D^{23}$ +41.5 (*c* 1.00, EtOH); lit.[3] $[\alpha]_D^{22}$ +42.4 (*c* 0.87, EtOH). The $^1H$ and $^{13}C$ NMR data of this material matched those of the enantiomer reported previously.[4]

**Alcohol 9.** To a solution of diol **S6** (1.34 g, 9.42 mmol) in $CH_2Cl_2$ (90.0 mL) were added *p*-$MeOC_6H_4CH(OMe)_2$ (1.91 mL, 12.2 mmol) and (±)-CSA (218.3 mg, 0.9423 mmol), and the resultant solution was stirred at room temperature for 1 h. The reaction mixture was neutralized with $Et_3N$ and concentrated under reduced pressure. Purification of the residue by flash column chromatography (silica gel, 5% EtOAc/hexanes) gave crude *p*-methoxybenzylidene acetal as a colorless oil, which was used in the next reaction without further purification.

To a solution of the above *p*-methoxybenzylidene acetal in $CH_2Cl_2$ (95.0 mL) at −78 °C was added DIBALH (1.02 M solution in *n*-hexane, 37.0 mL, 37.8 mmol), and the resultant solution was stirred at −78 °C for 10 min and then at 0 °C for 1 h. The reaction was quenched with MeOH. The resultant mixture was diluted with saturated aqueous potassium sodium tartrate solution and EtOAc and stirred vigorously at room temperature for 16 h. The organic layer was separated, washed with brine, dried ($Na_2SO_4$), filtered, and concentrated under reduced pressure. Purification of the residue by flash column chromatography (silica gel, 0 to 3 to 10% EtOAc/hexanes) gave alcohol **9** (2.22 g, 90% for the two steps) as a colorless oil. The optical purity of this material was assessed by chiral HPLC analysis (column: Chiralpak IB N-5, 4.6 mm I.D. × 250 mm; eluent: 5% *i*-PrOH/*n*-hexane; flow rate:

---

[3] H. Fujioka, N. Matsunaga, H. Kitagawa, Y. Nagatomi, M. Kondo and Y. Kita, *Tetrahedron Asymmetry*, 1995, **6**, 2117–2120.
[4] J. Wang and B. L. Pagenkopf, *Org. Lett.*, 2007, **9**, 3703–3706.

1.0 mL/min$^{-1}$; detection: 254 nm; $t_R$ = 6.8 min) to be >99% e.r.: $[\alpha]_D^{23}$ −48.1 (*c* 1.24, $CHCl_3$); IR (film) 3455, 3072, 2910, 1613, 1513, 1247, 1069, 1038, 916 cm$^{-1}$; $^1$H NMR (400 MHz, $CDCl_3$) δ 7.27–7.23 (m, 2H), 6.88–6.85 (m, 2H), 5.89–5.77 (m, 2H), 5.14 (m 1H), 5.10–5.05 (m, 3H), 4.61 (d, *J* = 10.8 Hz, 1H), 4.40 (d, *J* = 10.8 Hz, 1H), 3.79 (s, 3H), 3.60 (ddd, *J* = 7.6, 4.8, 4.8 Hz, 1H), 3.35 (ddd, *J* = 5.6, 5.6, 5.6 Hz, 1H), 2.46 (ddd, *J* = 14.0, 6.8, 6.8 Hz, 1H), 2.34–2.23 (m, 4H); $^{13}$C NMR (100 MHz, $CDCl_3$) δ 159.3, 134.8, 134.2, 130.2, 129.5 (2C), 117.5, 117.3, 113.8 (2C), 80.2, 71.9, 71.7, 55.2, 37.9, 34.7; HRMS (ESI) *m*/*z* calcd for $C_{16}H_{22}O_3Na^+$ [(M + Na)$^+$] 285.1461, found 285.1457.

At this stage, the absolute configuration of **9** was confirmed by a modified Mosher analysis as shown below:

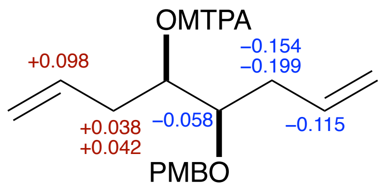


**Alcohol 15.** To a solution of alcohol **9** (1.06 g, 4.04 mmol) in 2-propanol (40.4 mL) were added **Co-I** complex (229.7 mg, 0.4061 mmol) and *t*-BuOOH (6.43 M solution in isooctane, 0.065 mL, 0.42 mmol), and the resultant mixture was stirred at 60 °C under an atmosphere of $O_2$ (balloon) for 3 h. The reaction mixture was cooled to room temperature and concentrated under reduced pressure. Purification of the residue by flash column chromatography (silica gel, 40 to 50% EtOAc/hexanes) gave alcohol **15** (686.0 mg, 61%, d.r. >20:1) as a colorless oil: $[\alpha]_D^{23}$ −39.7 (*c* 0.66, $CHCl_3$); IR (film) 3425, 2929, 1612, 1513, 1246, 1069, 1038, 916 cm$^{-1}$; $^1$H NMR (400 MHz, $CDCl_3$) δ 7.25–7.22 (m, 2H), 6.87–6.84 (m, 2H), 5.79 (dddd, *J* = 17.2, 10.4, 6.8, 6.8 Hz, 1H), 5.10 (dd, *J* = 17.2, 1.6 Hz, 1H), 5.02 (dd, *J* = 10.4, 1.6 Hz, 1H), 4.53 (d, *J* = 11.6 Hz, 1H), 4.35 (d, *J* = 11.6 Hz, 1H), 4.28 (m, 1H), 3.99 (m, 1H), 3.92 (ddd, *J* = 7.2, 7.2, 3.6 Hz, 1H), 3.78 (s, 3H), 3.70 (dd, *J* = 11.6, 2.8 Hz, 1H), 3.46 (dd, *J* = 12.0, 5.2 Hz, 1H), 2.43 (ddd, *J* = 6.8, 6.8, 1.2 Hz, 2H), 2.09 (ddd, *J* =13.6, 6.4, 1.6 Hz, 1H), 2.02 (br s, 1H), 1.81 (ddd, *J* = 13.6, 9.2, 4.8 Hz, 1H); $^{13}$C NMR (100 MHz, $CDCl_3$) δ 159.1, 135.1, 130.2, 129.1 (2C), 116.8, 113.7 (2C), 81.9, 79.1, 77.6, 70.9, 64.6, 55.2, 33.8, 32.8; HRMS (ESI) *m*/*z* calcd for $C_{16}H_{22}O_4Na^+$ [(M + Na)$^+$] 301.1410, found 301.1411.

**Acetate S8.** To a solution of alcohol **15** (358.9 mg, 1.289 mmol) in THF (12.0 mL) at 0 °C were added $Et_3N$ (0.715 mL, 5.16 mmol), $Ac_2O$ (0.370 mL, 3.91 mmol), and DMAP (33.0 mg, 0.270 mmol), and the resultant solution was stirred at room temperature for 1.5 h. The reaction was quenched with MeOH at 0 °C. The resultant mixture was diluted with EtOAc, washed with $H_2O$ and brine, dried ($Na_2SO_4$), filtered, and concentrated under reduced pressure. Purification of the residue by flash column chromatography (silica gel, 15 to 20% EtOAc/hexanes) gave acetate **S8** (352.1 mg, 85%) as a colorless oil: $[\alpha]_D^{23}$ −35.7 (*c* 0.61, $CHCl_3$); IR (film) 2934, 1738, 1512, 1242, 1035 $cm^{-1}$; $^1H$ NMR (400 MHz, $CDCl_3$) δ 7.25–7.22 (m, 2H), 6.88–6.84 (m, 2H), 5.77 (dddd, *J* = 17.2, 10.4, 7.2, 7.2 Hz, 1H), 5.10 (dddd *J* = 17.2, 2.0, 1.2, 1.2 Hz, 1H), 5.02 (dddd, *J* = 10.4, 2.0, 1.2, 1.2 Hz, 1H), 4.53 (d, *J* = 11.6 Hz, 1H), 4.39 (dddd, *J* = 9.2, 6.4, 6.4, 3.2 Hz, 1H), 4.35 (d, *J* = 11.6 Hz, 1H), 4.14 (dd, *J* = 11.6, 3.2 Hz, 1H), 3.99 (dd, *J* = 11.6, 6.4 Hz, 1H), 3.99–3.92 (m, 2H), 3.79 (s, 3H), 2.51–2.39 (m, 2H), 2.18 (ddd, *J* = 13.2, 6.4, 1.2 Hz, 1H), 2.06 (s, 3H), 1.70 (ddd, *J* = 13.2, 9.2, 4.8 Hz, 1H); $^{13}C$ NMR (100 MHz, $CDCl_3$) δ 171.1, 159.1, 134.9, 130.2, 129.1 (2C), 116.9, 113.7 (2C), 82.0, 78.5, 74.9, 70.9, 66.5, 55.3, 33.8, 33.7, 21.0; HRMS (ESI) *m/z* calcd for $C_{18}H_{24}O_5Na^+$ [$(M + Na)^+$] 343.1516, found 343.1517.

**Alcohol 7.** To a solution of acetate **S8** (270.3 mg, 0.8436 mmol) and $Et_3SiH$ (0.400 mL, 2.51 mmol) in $CH_2Cl_2$ (8.00 mL) at −35 °C was added $BF_3$•$OEt_2$ (0.160 mL, 1.27 mmol), and the resultant solution was gradually warmed to 0 °C and stirred at 0 °C for 2.5 h. The reaction was quenched with saturated aqueous $NaHCO_3$ solution at 0 °C. The resultant mixture was diluted with EtOAc, washed with $H_2O$ and brine, dried ($Na_2SO_4$), filtered, and concentrated under reduced pressure. Purification of the residue by flash column chromatography (silica gel, 40 to 50% EtOAc/hexanes) gave alcohol **7** (153.6 mg, 91%) as a colorless oil: $[\alpha]_D^{23}$ +5.8 (*c* 1.00, $CHCl_3$); IR (film) 3447, 2936, 1737, 1370, 1238, 1034 $cm^{-1}$; $^1H$ NMR (400 MHz, $CDCl_3$) δ 5.79 (dddd, *J* = 17.2, 10.4, 7.2, 7.2 Hz, 1H), 5.11 (dddd, *J* = 17.2, 2.0, 1.6, 1.6 Hz, 1H), 5.03 (dddd, *J* = 10.4, 2.0, 1.2, 1.2 Hz, 1H), 4.41 (dddd, *J* = 9.6, 6.4, 6.4, 3.2 Hz, 1H), 4.24 (br t, *J* = 3.2 Hz, 1H), 4.08 (dd, *J* = 12.0, 3.2 Hz, 1H), 3.95 (dd, *J* = 12.0, 6.4 Hz, 1H), 3.84

(ddd, $J$ = 8.0, 6.4, 3.2 Hz, 1H), 2.46–2.31 (m, 2H), 2.23 (br s, 1H), 2.03 (s, 3H), 2.02 (dd, $J$ = 13.6, 6.4 Hz, 1H), 1.81 (ddd, $J$ = 13.6, 9.6, 4.8 Hz, 1H); $^{13}C$ NMR (100 MHz, $CDCl_3$) δ 171.1, 134.3, 117.2, 81.9, 74.7, 72.3, 66.4, 37.4, 33.4, 20.8; HRMS (ESI) *m/z* calcd for $C_{10}H_{16}O_4Na^+$ $[(M + Na)^+]$ 223.0941, found 223.0948.

### 3. Optimization of fragment-assembly olefin cross-metathesis

**Table S1** Screening of reaction conditions using olefins **6** and **15**.[a]

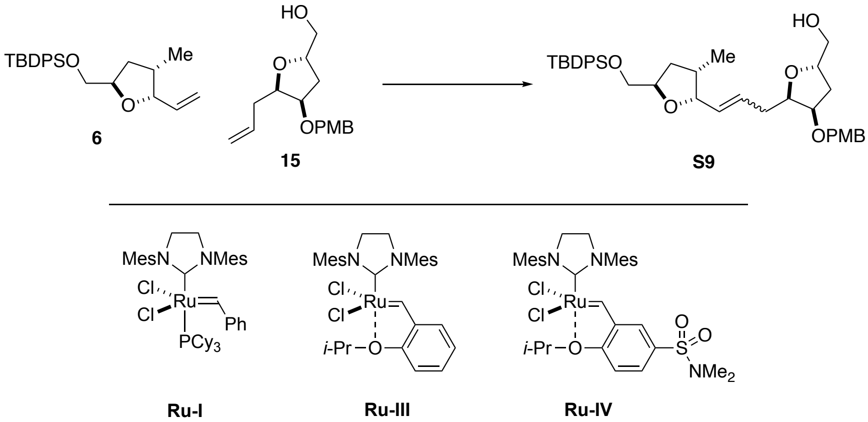


| Entry | Catalyst | Solvent | Temp./°C | Yield/% | *E*/*Z*[b] | Recov. **6** |
|---|---|---|---|---|---|---|
| 1 | **Ru-I** | DCE | 60 | 33 | 4:1 | 7 |
| 2 | **Ru-III** | DCE | reflux | 24 | 1:1 | N/A[c] |
| 3 | **Ru-III** | $CH_2Cl_2$ | 40 | 42 | >20:1 | 28 |
| 4 | **Ru-III** | $CH_2Cl_2$ | reflux | 46 | >20:1 | 29 |
| 5 | **Ru-III** | toluene | 60 | 40 | >20:1 | 21 |
| 6 | **Ru-IV** | DCE | 60 | 26 | 4:1 | 9 |

[a]All reactions were performed using **6** (1 equiv), **15** (2 equiv), ruthenium complex (5 mol%) in dry, degassed solvent at indicated temperature for 20 h. [b]Estimated by $^1$H NMR analysis on purified mixture. [c]N/A = not attained.

At the outset, we examined olefin cross-metathesis of olefins **6** and **15** using **Ru-I** in DCE at 60 °C for 20 h. Under these conditions, **S9** was isolated in 33% yield with *E*/*Z* 4:1 selectivity, along with 7% of recovered **6** (entry 1). $^1$H NMR analysis of crude reaction mixture indicated homo-dimerization of **6** and **15** should be responsible for the unsatisfactory result. Although changing the ruthenium complex to **Ru-III** was an unproductive attempt (entry 2), running the reaction in $CH_2Cl_2$ at 40 °C provided **S9** in 42% yield (*E*/*Z* >20:1) along with recovered **6** in 28% yield (entry 3). The present reaction was best performed by the action of **Ru-III** in refluxing $CH_2Cl_2$, delivering **S9** in 46% yield, with 29% recovery of **6** (entry 4). Toluene was less effective than $CH_2Cl_2$ (entry 5). Fast-initiating ruthenium alkylidene

complex **Ru-IV** was not significantly superior to **Ru-I** or **Ru-III** (entry 6).

After obtaining **S9** in an acceptable yield, we acetylated **S9** and subsequently made some attempts to remove the PMB group using DDQ or $Et_3SiH/BF_3{\bullet}OEt_2$, as shown in Scheme S2. However, all of our attempts were unfruitful and resulted in complex mixtures that contained **16** in only low yield (<25%). Thus, we decided to remove the PMB group prior to fragment-assembly olefin cross-metathesis.

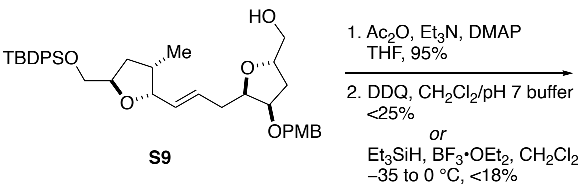


**Scheme S2** Unsuccessful attempts to synthesize olefin **16**.

**Table S2** Screening of reaction conditions using olefins **6** and **7**.[a]

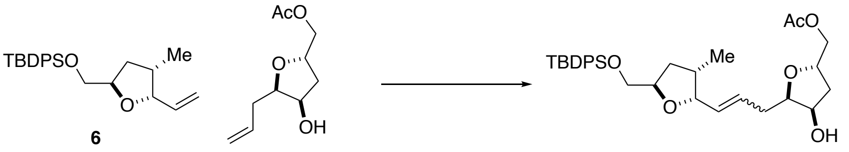


| Entry | Catalyst | Solvent | Temp./°C | Yield/% | *E*/*Z*[b] |
|---|---|---|---|---|---|
| 1 | **Ru-III** | $CH_2Cl_2$ | reflux | 65 | 4:1 |
| 2 | **Ru-III** | toluene | reflux | 42 | 4:1 |
| 3 | **Ru-I** | $CH_2Cl_2$ | reflux | 78 | 5:1 |
| 4[c] | **Ru-I** | $CH_2Cl_2$ | reflux | 84 | 5:1 |
| 5 | **Ru-IV** | toluene | 60 | no reaction | |

[a]All reactions were performed using **6** (1 equiv), **7** (2 equiv), ruthenium complex (5 mol%) in dry, degassed solvent at indicated temperature for 20 h. [b]Estimated by $^1$H NMR analysis on purified mixture. [c]0.658 mmol scale.

Our optimized reaction conditions (Table S1, entry 4) were applied to olefin cross-metathesis of olefins **6** and **7** (**Ru-III**, $CH_2Cl_2$, reflux) to deliver hydroxy olefin **16** in 65% yield (entry 1). Notably,

the starting material **6** was consumed completely, and the product yield was much better than that of Table S1, entry 4, suggesting that olefin **7** would be a superior substrate to **15**. While running the reaction in toluene at higher temperature was detrimental (entry 2), changing the ruthenium catalyst to **Ru-I** turned out to be beneficial (entries 3 and 4). Thus, it was found that exposure of a mixture of **6** and **7** to **Ru-I** (5 mol%) in refluxing $CH_2Cl_2$ for 20 h provided **16** in around 80% yield with excellent reproducibility. **Ru-IV** was completely ineffective in the present case (entry 5).

## 4. Synthesis of vinylstannane 4

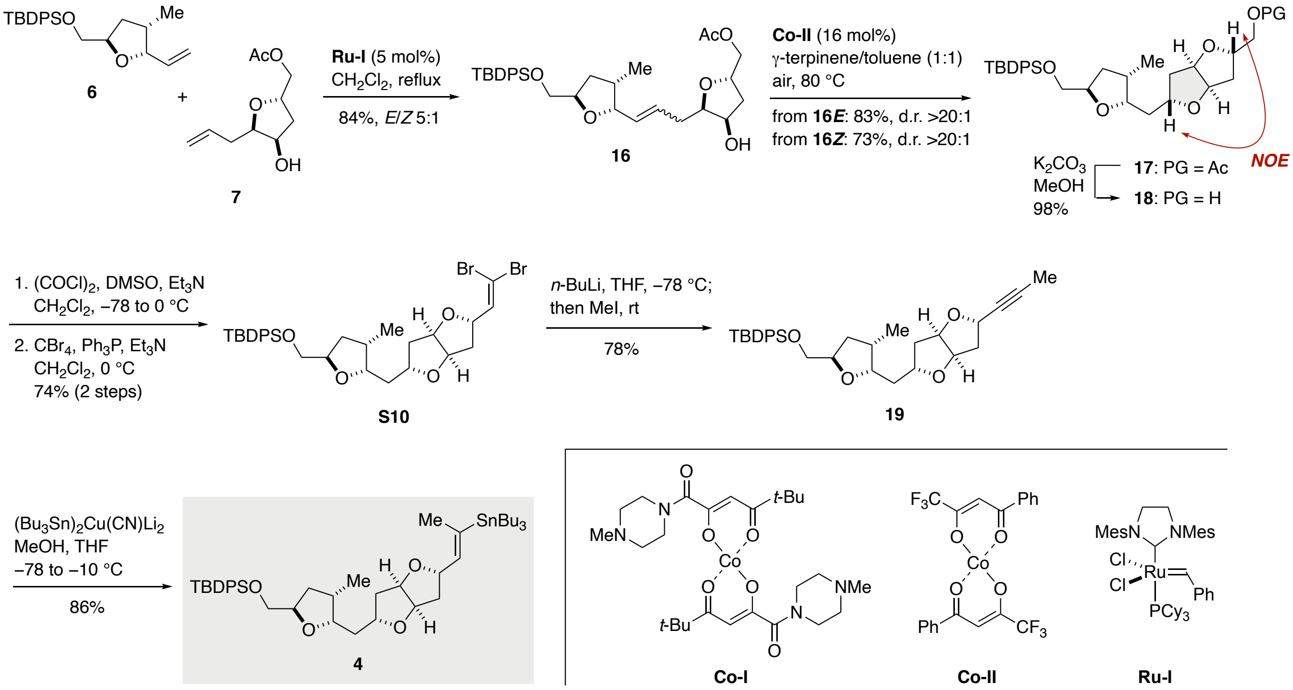

**Scheme S3** Synthesis of vinylstannane **4**.

**Olefins 16*E* and 16*Z*.** A solution of olefin **6** (250.3 mg, 0.6576 mmol), alcohol **7** (254.6 mg, 1.272 mmol), and the second-generation Grubbs complex (**Ru-I**, 29.6 mg, 0.0349 mmol) in degassed $CH_2Cl_2$ (12.00 mL) was stirred at 50 °C for 21.5 h. The resultant mixture was cooled to room temperature, stirred at room temperature under air for 1 h, and then concentrated under reduced pressure. Purification of the residue by flash column chromatography (silica gel, 30 to 40% EtOAc/hexanes) gave olefin **16*E*** (253.1 mg, 70%) and olefin **16*Z*** (51.9 mg, 14%) as brown colored oils: Data for **16*E***: $[\alpha]_D^{23}$ −5.0 (*c* 0.86, $CHCl_3$); IR (film) 3444, 2932, 2859, 1741, 1238, 1110, 1038, 705 $cm^{-1}$; $^1H$ NMR (400 MHz, $CDCl_3$) δ 7.68–7.64 (m, 4H), 7.42–7.32 (m, 6H), 5.67 (ddd, *J* = 15.2, 7.2, 6.0 Hz, 1H), 5.58 (dd, *J* = 15.2, 6.8 Hz, 1H), 4.45 (dddd, *J* = 9.6, 6.8, 6.8, 3.2 Hz, 1H), 4.35 (dd, *J* = 6.8, 6.8 Hz, 1H), 4.28 (br t, *J* = 3.6 Hz, 1H), 4.20 (dddd, *J* = 7.2, 5.2, 5.2, 4.8 Hz, 1H), 4.13 (dd, *J* = 11.6, 3.2 Hz, 1H), 3.98 (dd, *J* = 11.6, 6.8 Hz, 1H), 3.88 (ddd, *J* = 8.0, 6.0, 3.6 Hz, 1H), 3.63 (dd, *J* = 10.4, 4.8 Hz, 1H), 3.59 (dd, *J* = 10.4, 5.2 Hz, 1H), 2.50 (ddd, *J* =14.0, 6.0, 6.0 Hz, 1H), 2.41 (ddd, *J* = 14.0, 8.0, 7.2 Hz, 1H), 2.33 (dddq, *J* = 6.8, 6.8, 6.8, 6.8 Hz, 1H), 2.08–2.03 (m, 2H), 2.07 (s, 3H), 1.84 (ddd, *J* =

13.2, 9.6, 3.6 Hz, 1H), 1.67 (ddd, *J* = 13.2, 6.8, 5.2 Hz, 1H), 1.03 (s, 9H), 0.89 (d, *J* = 6.8 Hz, 3H), one proton missing due to H/D exchange; $^{13}$C NMR (100 MHz, $CDCl_3$) δ 171.0, 135.6 (2C), 135.6 (2C), 133.6 (2C), 130.8, 129.6, 129.5, 128.0, 127.6 (4C), 82.7, 82.0, 77.8, 74.8, 72.4, 66.6, 66.5, 37.5, 36.9, 35.7, 32.0, 26.8 (3C), 20.9, 19.2, 14.8; HRMS (ESI) *m/z* calcd for $C_{32}H_{44}O_6SiNa^+$ [(M + Na)$^+$] 575.2799, found 575.2791. Data for **16*Z***: $[\alpha]_D^{23}$ +1.3 (*c* 0.86, $CHCl_3$); IR (film) 3425, 2932, 2860, 1740, 1430, 1238, 1110, 1038, 705 cm$^{-1}$; $^1$H NMR (400 MHz, $CDCl_3$) δ 7.69–7.65 (m, 4H), 7.42–7.33 (m, 6H), 5.65–5.57 (m, 2H), 4.82 (dd, *J* = 6.0, 6.0 Hz, 1H), 4.49 (dddd, *J* = 9.6, 6.4, 6.4, 2.8 Hz, 1H), 4.22–4.16 (m, 2H), 4.16 (dd, *J* = 11.6, 2.8 Hz, 1H), 3.98 (dd, *J* = 11.6, 6.4 Hz, 1H), 3.87 (ddd, *J* = 10.8, 4.4, 2.4 Hz, 1H), 3.76 (d, *J* = 8.0 Hz, 1H), 3.61 (d, *J* = 4.8 Hz, 2H), 2.87 (m, 1H), 2.37–2.33 (m, 2H), 2.07 (s, 3H), 2.03 (dd, *J* = 12.4, 5.6 Hz, 1H), 2.02 (dd, *J* = 14.8, 6.4 Hz, 1H), 1.76 (ddd, *J* = 14.8, 9.6, 4.8 Hz, 1H), 1.70 (ddd, *J* = 12.4, 7.2, 5.2 Hz, 1H), 1.04 (s, 9H), 0.95 (d, *J* = 7.2 Hz, 3H); $^{13}$C NMR (100 MHz, $CDCl_3$) δ 171.1, 135.59 (2C), 135.56 (2C), 133.4, 133.3, 130.4, 129.5 (2C), 128.0, 127.6 (4C), 82.5, 77.7, 76.9, 75.4, 71.1, 66.7, 66.2, 36.9, 35.8, 35.7, 28.2, 26.7 (3C), 20.9, 19.2, 14.3; HRMS (ESI) *m/z* calcd for $C_{32}H_{44}O_6SiNa^+$ [(M + Na)$^+$] 575.2799, found 575.2791.

**Tetrahydrofuran 17.** To a solution of olefin **16*E*** (270.9 mg, 0.4901 mmol) in toluene/γ-terpinene (1:1, v/v, 5.00 mL) was added **Co-II** (41.5 mg, 0.0790 mmol), and the resultant mixture was stirred at 80 °C under air for 4 h. The resultant mixture was cooled to room temperature and concentrated under reduced pressure. Purification of the residue by flash column chromatography (silica gel, 30 to 40% *t*-BuOMe/hexanes) gave tetrahydrofuran **17** (223.7 mg, 83%, d.r. >20:1) as a greenish oil: $[\alpha]_D^{24}$ −3.5 (*c* 1.03, $CHCl_3$); IR (film) 2935, 2860, 1742, 1430, 1237, 1109, 1041, 705 cm$^{-1}$; $^1$H NMR (400 MHz, $C_6D_6$) δ 7.85–7.82 (m, 4H), 7.31–7.25 (m, 6H), 4.47 (dd, *J* = 4.4, 4.4 Hz, 1H), 4.44 (dd, *J* = 4.4, 4.4 Hz, 1H), 4.35 (m, 1H), 4.18 (dddd, *J* = 9.2, 5.6, 5.6, 3.6 Hz, 1H), 4.15 (dddd, *J* = 12.0, 7.6, 4.8, 4.8 Hz, 1H), 3.99 (dd, *J* = 11.6, 3.6 Hz, 1H), 3.94 (m, 1H), 3.93 (dd, *J* = 11.6, 5.6 Hz, 1H), 3.67 (dd, *J* = 10.8, 4.8 Hz, 1H), 3.57 (dd, *J* = 10.8, 4.8 Hz, 1H), 2.20 (dd, *J* = 13.2, 5.2 Hz, 1H), 2.03 (ddd, *J* = 14.0, 9.2, 5.2 Hz, 1H), 1.98 (m, 1H), 1.89 (dd, *J* =13.2, 5.6 Hz, 1H), 1.81 (ddd, *J* = 12.4, 7.6, 7.6 Hz, 1H), 1.65

(s, 3H), 1.42 (ddd, $J$ = 14.0, 7.6, 4.4 Hz, 1H), 1.38–1.31 (m, 3H), 1.19 (s, 9H), 0.72 (d, $J$ = 6.8 Hz, 3H); $^{13}$C NMR (100 MHz, $C_6D_6$) δ 170.0, 136.1 (2C), 136.0 (2C), 134.13, 134.06, 129.9 (2C), 128.1 (4C), 84.5, 83.3, 78.6, 77.9, 77.8, 77.6, 67.1, 66.3, 41.0, 37.7, 36.9, 36.5, 35.8, 27.0 (3C), 20.4, 19.5, 14.3; HRMS (ESI) *m*/*z* calcd for $C_{32}H_{44}O_6SiNa^+$ [(M + Na)$^+$] 575.2799, found 575.2798.

**Alcohol 18.** To a solution of tetrahydrofuran **17** (33.6 mg, 0.0608 mmol) in MeOH/THF (1:1, v/v, 2.00 mL) was added $K_2CO_3$ (1.5 mg, 0.0108 mmol), and the resultant mixture was stirred at room temperature for 4 h. To this mixture was added an additional portion of $K_2CO_3$ (0.7 mg, 0.005 mmol), and the resultant mixture was stirred at room temperature for 3 h. The reaction was quenched with saturated aqueous $NH_4Cl$ solution. The resultant mixture was diluted with EtOAc, washed with $H_2O$ and brine, dried ($Na_2SO_4$), filtered, and concentrated under reduced pressure. Purification of the residue by flash column chromatography (silica gel, 40% EtOAc/hexanes) gave alcohol **18** (30.5 mg, 98%) as a colorless oil: $[\alpha]_D^{23}$ −10.1 (*c* 0.77, $CHCl_3$); IR (film) 3442, 2931, 2861, 1465, 1429, 1108, 1043, 704 cm$^{-1}$; $^1$H NMR (400 MHz, $CDCl_3$) δ 7.68–7.65 (m, 4H), 7.40–7.34 (m, 6H), 4.70 (dd, $J$ = 4.8, 4.8 Hz, 1H), 4.67 (dd, $J$ = 4.8, 4.8 Hz, 1H), 4.22–5.15 (m, 3H), 3.95 (ddd, $J$ = 9.2, 4.4, 4.4 Hz, 1H), 3.73 (dd, $J$ =11.6, 2.8 Hz, 1H), 3.63 (dd, $J$ = 10.8, 4.8 Hz, 1H), 3.59 (dd, $J$ = 10.8, 4.8 Hz, 1H), 3.44 (dd, $J$ = 11.6, 4.8 Hz, 1H), 2.25 (m, 1H), 2.22 (dd, $J$ = 13.2, 4.8 Hz, 1H), 2.05 (dd, $J$ = 13.2, 7.2 Hz, 1H), 2.01 (dd, $J$ = 13.2, 4.8 Hz, 1H), 1.88 (ddd, $J$ = 13.2, 8.0, 5.6 Hz, 1H), 1.84 (ddd, $J$ = 13.2, 9.2, 4.4 Hz, 1H), 1.66 (ddd, $J$ = 13.2, 9.2, 4.8 Hz, 1H), 1.65 (ddd, $J$ = 13.2, 4.8, 4.0 Hz, 1H), 1.51 (ddd, $J$ = 13.2, 7.2, 4.4 Hz, 1H), 1.03 (s, 9H), 0.90 (d, $J$ = 7.2 Hz, 3H), one proton missing due to H/D exchange; $^{13}$C NMR (100 MHz, $CDCl_3$) δ 135.6 (4C), 133.6 (2C), 129.57, 129.53, 127.6 (4C), 84.3, 83.6, 80.3, 78.6, 77.9, 77.2, 66.5, 64.1, 40.8, 36.4, 36.27, 36.24, 35.7, 26.8 (3C), 19.3, 14.3; HRMS (ESI) *m*/*z* calcd for $C_{30}H_{42}O_5SiNa^+$ [(M + Na)$^+$] 533.2694, found 533.2699.

**Dibromoolefin S10.** To a solution of $(COCl)_2$ (0.110 mL, 1.71 mmol) in $CH_2Cl_2$ (4.00 mL) at −78 °C was added DMSO (0.180 mL, 2.53 mmol), and the resultant mixture was stirred at −78 °C for 15 min.

To this mixture was added a solution of alcohol **18** (165.3 mg, 0.3236 mmol) in $CH_2Cl_2$ (2.00 mL + 1.00 mL rinse), and the resultant mixture was stirred at −78 °C for 30 min. To the reaction mixture was added $Et_3N$ (0.530 mL, 3.82 mmol), and the resultant mixture was allowed to warm to 0 °C for 1.5 h. The reaction was quenched with saturated aqueous $NaHCO_3$ solution at 0 °C. The resultant mixture was diluted with *t*-BuOMe, washed with $H_2O$ and brine, dried ($MgSO_4$), filtered, and concentrated under reduced pressure to give crude aldehyde as a yellow oil, which was used in the next reaction without further purification.

To a solution of $CBr_4$ (427.4 mg, 1.289 mmol) in $CH_2Cl_2$ (4.00 mL) at 0 °C was added $Ph_3P$ (679.0 mg, 2.589 mmol), and the resultant mixture was stirred at 0 °C for 30 min. To this mixture were added $Et_3N$ (0.540 mL, 3.89 mmol) and a solution of the above aldehyde in $CH_2Cl_2$ (2.00 mL + 1.00 mL rinse), and the resultant mixture was stirred at 0 °C for 3 h. The reaction was quenched with saturated aqueous $NaHCO_3$ solution at 0 °C. The resultant mixture was diluted with EtOAc, washed with $H_2O$ and brine, dried ($MgSO_4$), filtered, and concentrated under reduced pressure. Purification of the residue by flash column chromatography (silica gel, 10% EtOAc/hexanes) gave dibromoolefin **S10** (159.5 mg, 74% for the two steps) as a yellow oil: $[\alpha]_D^{24}$ +3.1 (*c* 0.66, $CHCl_3$); IR (film) 2932, 2860, 1430, 1108, 704 $cm^{-1}$; $^1H$ NMR (400 MHz, $CDCl_3$) δ 7.69–7.65 (m, 4H), 7.42–7.34 (m, 6H), 6.41 (d, *J* = 7.6 Hz, 1H), 4.70–4.68 (m, 2H), 4,66 (ddd, *J* = 10.0, 7.6, 5.6 Hz, 1H), 4.26–4.16 (m, 2H), 3.95 (ddd, *J* = 9.2, 4.4, 4.4 Hz, 1H), 3.64 (dd, *J* = 10.8, 4.8 Hz, 1H), 3.59 (dd, *J* = 10.8, 4.8 Hz, 1H), 2.34 (dd, *J* = 13.2, 5.6 Hz, 1H), 2.25 (m, 1H), 2.23 (dd, *J* = 13.2, 4.8 Hz, 1H), 2.05 (ddd, *J* = 12.4, 7.2, 7.2 Hz, 1H), 1.87 (ddd, *J* = 14.0, 9.2, 5.6 Hz, 1H), 1.75–1.62 (m, 3H), 1.51 (ddd, *J* = 14.0, 7.2, 4.4 Hz, 1H), 1.03 (s, 9H), 0.90 (d, *J* = 6.8 Hz, 3H); $^{13}C$ NMR (100 MHz, $CDCl_3$) $\delta$ 138.6, 135.6 (4C), 133.6 (2C), 129.57, 129.55, 127.6 (4C), 91.2, 84.4, 83.1, 79.7, 78.5, 78.2, 77.4, 66.5, 40.6, 40.1, 36.4, 36.3, 35.7, 26.8 (3C), 19.2, 14.3; HRMS (ESI) *m/z* calcd for $C_{31}H_{40}{}^{79}Br_2O_4SiNa^+$ $[(M + Na)^+]$ 685.0955, found 685.0976.

**Alkyne 19.** To a solution of dibromoolefin **S10** (129.2 mg, 0.1944 mmol) in THF (2.00 mL) at −78 °C was added *n*-BuLi (2.67 M solution in *n*-hexane, 0.185 mL, 0.494 mmol), and the resultant solution

was stirred at −78 °C for 30 min. To the reaction mixture was added MeI (0.0700 mL, 1.12 mmol), and the resultant mixture was allowed to warm to room temperature over a period of 4 h. The reaction was quenched with saturated aqueous $NH_4Cl$ solution at 0 °C. The resultant mixture was diluted with EtOAc, washed with $H_2O$ and brine, dried ($Na_2SO_4$), filtered, and concentrated under reduced pressure. Purification of the residue by flash column chromatography (silica gel, 1 to 5% *t*-BuOMe/toluene) gave alkyne **19** (78.3 mg, 78%) as a colorless oil: $[\alpha]_D^{22}$ −13.6 (*c* 0.32, $CHCl_3$); IR (film) 2928, 2858, 1465, 1430, 1108, 704 $cm^{-1}$; $^1H$ NMR (400 MHz, $CDCl_3$) δ 7.68–7.64 (m, 4H), 7.40–7.33 (m, 6H), 4.74–4.71 (m, 2H), 4.66 (m, 1H), 4.20–4.08 (m, 2H), 3.93 (ddd, *J* = 9.2, 4.4, 4.4 Hz, 1H), 3.62 (dd, *J* = 10.4, 4.8 Hz, 1H), 3.58 (dd, *J* = 10.4, 4.8 Hz, 1H), 2.25–2.22 (m, 2H), 2.19 (dd, *J* = 13.2, 4.8 Hz, 1H), 2.09–1.99 (m, 2H), 1.84 (ddd, *J* = 13.2, 9.2, 5.6 Hz, 1H), 1.82 (d, *J* = 2.0 Hz, 3H), 1.67–1.61 (m, 2H), 1.50 (ddd, *J* = 13.2, 6.8, 4.4 Hz, 1H), 1.03 (s, 9H), 0.88 (d, *J* = 6.8 Hz, 3H); $^{13}C$ NMR (100 MHz, $CDCl_3$) δ 135.6 (4C), 133.64, 133.62, 129.5 (2C), 127.6 (4C), 83.6, 83.0, 81.4, 78.6, 77.9, 77.5, 77.2, 69.1, 66.5, 42.3, 40.1, 36.3, 36.1, 35.8, 26.8 (3C), 19.3, 14.3, 3.6; HRMS (ESI) *m*/*z* calcd for $C_{32}H_{42}O_4SiNa^+$ $[(M + Na)^+]$ 541.2745, found 541.2741.

**Vinylstannane 4.** To a suspension of CuCN (46.0 mg, 0.503 mmol) in THF (1.00 mL) at −78 °C was added *n*-BuLi (2.67 M solution in *n*-hexane, 0.375 mL, 1.00 mmol), and the resultant mixture was stirred at −40 °C for 20 min. To the reaction mixture at −78 °C was added *n*-$Bu_3SnH$ (0.270 mL, 0.100 mmol), and the resultant mixture was stirred at −40 °C for 15 min. To the reaction mixture at −78 °C was added MeOH (0.400 mL, 9.86 mmol), and the resultant mixture was stirred at −10 °C for 30 min. To the reaction mixture at −78 °C was added a solution of alkyne **19** (51.8 mg, 0.0998 mmol) in THF (0.700 mL + 0.300 mL rinse), and the resultant mixture was stirred at −78 °C for 5 min and then at −10 °C for 6 h. The reaction was quenched with a mixture of saturated aqueous $NH_4Cl$ solution/30% $NH_4OH$ solution (4:1, v/v, 5.00 mL) at −10 °C. The resultant mixture was extracted with $Et_2O$, and the organic layer was washed with brine, dried ($MgSO_4$), filtered, and concentrated under reduced pressure. Purification of the residue by flash column chromatography (silica gel, 0 to 3 to 5% EtOAc/hexanes)

gave vinylstannane **4** (69.8 mg, 86%) as a yellow oil: $[\alpha]_D^{22}$ −9.7 (*c* 1.39, $CHCl_3$); IR (film) 2955, 2926, 2855, 1458, 1428, 1111, 703 $cm^{-1}$; $^1H$ NMR (400 MHz, $C_6D_6$) δ 7.86–7.82 (m, 4H), 7.32–7.24 (m, 6H), 5.89 (m, 1H), 5.16 (ddd, *J* = 10.8, 7.6, 5.6 Hz, 1H), 4.64 (dd, *J* = 4.4, 4.4 Hz, 1H), 4.57 (dd, *J* = 4.4 Hz, 1H), 4.53 (m, 1H), 4.14 (ddd, *J* = 12.0, 7.6, 4.4 Hz, 1H), 3.98 (ddd, *J* = 9.2, 4.4, 4.4 Hz, 1H), 3.67 (dd, *J* = 10.4, 4.4 Hz, 1H), 3.57 (dd, *J* = 10.4, 4.4 Hz, 1H), 2.32 (dd, *J* = 12.8, 7.6 Hz, 1H), 2.31 (dd, *J* = 13.2, 7.6 Hz, 1H), 2.08 (ddd, *J* = 14.0, 9.2, 5.6 Hz, 1H), 2.00 (m, 1H), 1.88 (d, *J* = 1.6 Hz, 3H), 1.80 (ddd, *J* = 12.8, 7.6, 7.6 Hz, 1H), 1.62–1.50 (m, 7H), 1.50–1.30 (m, 8H), 1.19 (s, 9H), 1.10–0.83 (m, 16H), 0.73 (d, *J* = 7.2 Hz, 3H); $^{13}C$ NMR (100 MHz, $C_6D_6$) δ 142.5, 140.7, 136.1 (2C), 136.0 (2C), 134.2, 134.1, 129.9 (2C), 128.1 (4C), 84.0, 83.9, 78.7, 78.5, 77.6, 75.9, 67.1, 42.5, 41.6, 37.2, 36.5, 35.8, 29.6 (3C), 27.8 (3C), 27.1 (3C), 20.0, 19.5, 14.4, 13.9 (3C), 9.4 (3C); HRMS (ESI) *m*/*z* calcd for $C_{44}H_{70}O_4SiSnNa^+$ $[(M + Na)^+]$ 833.3958, found 833.3971.

## 5. Synthesis of iodoolefin 5

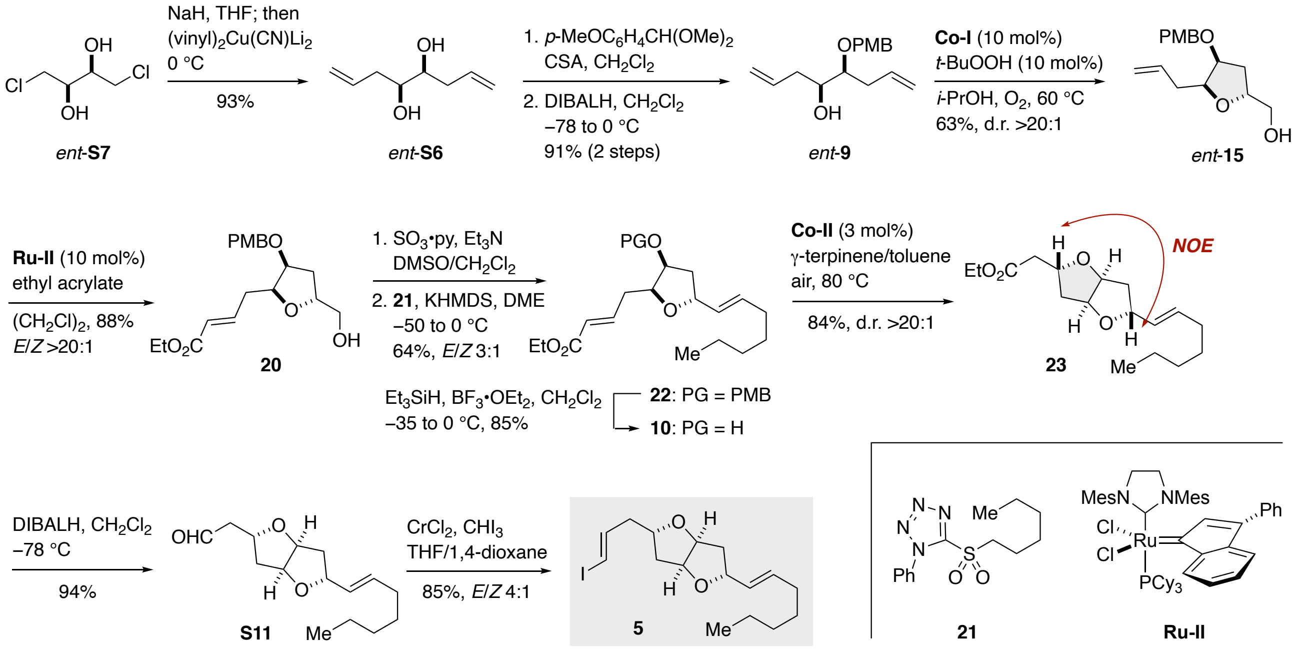


**Scheme S4** Synthesis of iodoolefin **5**.

**Diol *ent*-S6.** To a suspension of NaH (60% in mineral oil, 171.2 mg, 4.280 mmol) in THF (4.00 mL) at 0 °C was added a solution of diol *ent*-**S7** (>99% e.r., 260.3 mg, 1.648 mmol) in THF (3.00 mL + 1.00 mL rinse), and the resultant mixture was stirred at room temperature for 2 h to give a THF solution of diepoxide.

To a solution of tetra(vinyl)tin (0.540 mL, 3.05 mmol) in THF (5.00 mL) at −78 °C was added MeLi (1.42 M solution in cyclopentyl methyl ether, 7.20 mL, 10.2 mmol), and the resultant solution was stirred at −78 °C for 2 h and then at room temperature for 0.5 h. The resultant solution of vinyl lithium was transferred to a suspension of CuCN (456.1 mg, 5.093 mmol) in THF (5.00 mL) at −78 °C (rinsed with 3.00 mL of THF), and the resultant mixture was stirred at −78 °C for 10 min and then at 0 °C for 20 min. To the reaction mixture was added the above diepoxide solution (rinsed with 3.00 mL of THF), and the resultant mixture was stirred at 0 °C for 1 h. The reaction was quenched with 9:1 (v/v) mixture of saturated aqueous $NH_4Cl$ solution/28% $NH_4OH$ solution. The resultant mixture was stirred at room temperature for 1 h and then extracted with EtOAc. The organic layer was washed with $H_2O$ and brine,

dried ($Na_2SO_4$), filtered, and concentrated under reduced pressure. Purification of the residue by flash column chromatography (silica gel, 20 to 30% EtOAc/hexanes) gave diol *ent*-**S6** (214.2 mg, 93%) as colorless crystals: $[\alpha]_D^{23}$ −40.4 (*c* 1.00, EtOH); lit.[4] $[\alpha]_D$ −43.8 (*c* 0.016, EtOH); lit.[5] $[\alpha]_D^{26}$ −41.3 (*c* 1.01, EtOH). The $^1$H and $^{13}$C NMR data of this material matched those reported previously.[4,5]

**Alcohol *ent*-9.** To a solution of diol *ent*-**S6** (1.32 g, 9.28 mmol) in $CH_2Cl_2$ (90.0 mL) were added *p*-$MeOC_6H_4CH(OMe)_2$ (1.90 mL, 11.2 mmol) and (±)-CSA (216.0 mg, 0.9298 mmol), and the resultant solution was stirred at room temperature for 1 h. The reaction was quenched with $Et_3N$, and the resultant mixture was concentrated under reduced pressure. Purification of the residue by flash column chromatography (silica gel, 5% EtOAc/hexanes) gave *p*-methoxybenzylidene acetal as a colorless oil.

To a solution of the above *p*-methoxybenzylidene acetal in $CH_2Cl_2$ (90.0 mL) at −78 °C was slowly added DIBALH (1.02 M solution in *n*-hexane, 36.5 mL, 37.2 mmol), and the resultant solution was stirred at −78 °C for 10 min and then at 0 °C for 1 h. The reaction was quenched with MeOH. The resultant solution was diluted with EtOAc and saturated aqueous potassium sodium tartrate solution and stirred vigorously at room temperature for 12 h. The organic layer was separated, washed with brine, dried ($MgSO_4$), filtered, and concentrated under reduced pressure. Purification of the residue by flash column chromatography (silica gel, 10 to 20% EtOAc/hexanes) gave alcohol *ent*-**9** (2.21 g, 91% for the two steps) as a colorless oil. The optical purity of this material was assessed by chiral HPLC analysis (column: Chiralpak IB N-5, 4.6 mm I.D. × 250 mm; eluent: 5% *i*-PrOH/*n*-hexane; flow rate: 1.0 mL/min$^{-1}$; detection: 254 nm; $t_R$ = 6.4 min) to be >99% e.r.: $[\alpha]_D^{22}$ +51.9 (*c* 1.24, $CHCl_3$). The $^1$H and $^{13}$C NMR data of this material matched those of the enantiomer.

**Alcohol *ent*-15.** To a solution of alcohol *ent*-**9** (1.96 g, 7.47 mmol) in 2-propanol (70.0 mL) were added $Co(nmp)_2$ (**Co-I**, 0.43 g, 0.76 mmol) and *t*-BuOOH (6.43 M solution in isooctane, 0.115 mL, 0.739

[5] F. Yoshimura, T. Okada and K. Tanino, *Org. Lett.*, 2019, **21**, 559–562.

mmol), and the resultant mixture was stirred at 60 °C under an atmosphere of $O_2$ (balloon) for 2 h. The resultant mixture was cooled to room temperature and concentrated under reduced pressure. Purification of the residue by flash column chromatography (silica gel, 40 to 50% EtOAc/hexanes) gave alcohol *ent*-**15** (1.31 g, 63%, d.r. >20:1) as a colorless oil: $[\alpha]_D^{24}$ +45.3 (*c* 0.66, $CHCl_3$); IR (film) 3460, 2935, 2909, 1613, 1514, 1249, 1068, 1035, 915 cm$^{-1}$; $^1$H NMR (500 MHz, $CDCl_3$) δ 7.25–7.22 (m, 2H), 6.87–6.84 (m, 2H), 5.79 (dddd, *J* = 17.0, 10.0, 7.0, 7.0 Hz, 1H), 5.10 (dd, *J* = 17.0, 1.5 Hz, 1H), 5.02 (dd, *J* = 10.0, 1.5 Hz, 1H), 4.53 (d, *J* = 11.5 Hz, 1H), 4.35 (d, *J* = 11.5 Hz, 1H), 4.28 (m, 1H), 3.99 (m, 1H), 3.92 (ddd, *J* = 7.0, 7.0, 3.0 Hz, 1H), 3.78 (s, 3H), 3.70 (br d, *J* = 11.5 Hz, 1H), 3.46 (br dd, *J* = 12.0, 5.0 Hz, 1H), 2.43 (ddd, *J* = 7.0, 7.0, 1.5 Hz, 2H), 2.09 (ddd, *J* =13.5, 6.0, 1.5 Hz, 1H), 2.07 (br s, 1H), 1.81 (ddd, *J* = 13.5, 9.0, 5.0 Hz, 1H); $^{13}$C NMR (125 MHz, $CDCl_3$) δ 159.1, 135.1, 130.3, 129.1 (2C), 116.8, 113.7 (2C), 81.9, 79.1, 77.6, 70.9, 64.6, 55.2, 33.8, 32.8; HRMS (ESI) *m/z* calcd for $C_{16}H_{22}O_4Na^+$ $[(M + Na)^+]$ 301.1410, found 301.1428.

**α,β-Unsaturated ester 20.** To a degassed solution of alcohol *ent*-**15** (1.42 g, 5.10 mmol) in DCE (13.0 mL) were added ethyl acrylate (2.80 mL, 25.7 mmol) and a degassed solution of Umicore M2 (**Ru-II**, 48.0 mg, 0.0507 mmol) in DCE (12.0 mL), and the resultant solution was stirred at 40 °C for 17 h 20 min. To the reaction mixture were added additional portions of ethyl acrylate (1.10 mL, 10.1 mmol) and **Ru-II** complex (26.1 mg, 0.0275 mmol), and the resultant mixture was stirred at 40 °C for 4 h to push the reaction to completion. After consumption of the starting material, the reaction mixture was allowed to stir at room temperature under air for 15 h. The resultant mixture was concentrated under reduced pressure. Purification of the residue by flash column chromatography (silica gel, 40 to 50% EtOAc/hexanes) gave α,β-unsaturated ester **20** (1.58 g, 88%, *E/Z* >20:1 as judged by $^1$H NMR analysis) as a brown oil: $[\alpha]_D^{24}$ +38.0 (*c* 0.44, $CHCl_3$); IR (film) 3449, 2933, 1714, 1514, 1250, 1175, 1039 cm$^{-1}$; $^1$H NMR (500 MHz, $CDCl_3$) δ 7.23–7.20 (m, 2H), 6.92 (ddd, *J* = 16.0, 7.5, 7.5 Hz, 1H), 6.87–6.84 (m, 2H), 5.86 (ddd, *J* = 16.0, 1.5, 1.5 Hz, 1H), 4.53 (d, *J* = 11.5 Hz, 1H), 4.33 (d, *J* = 11.5 Hz, 1H), 4.27 (m, 1H), 4.15 (q, *J* = 7.0 Hz, 2H), 4.02–3.98 (m, 2H), 3.78 (s, 3H), 3.70 (ddd, *J* = 11.5,

3.0, 3.0 Hz, 1H), 3.46 (ddd, *J* = 11.5, 6.0, 6.0 Hz, 1H), 2.56–2.52 (m, 2H), 2.10 (ddd, *J* = 13.5, 6.0, 1.5 Hz, 1H), 2.06 (t, *J* = 6.0 Hz, 1H), 1.82 (ddd, *J* = 13.5, 9.0, 5.0 Hz, 1H), 1.26 (t, *J* = 7.0 Hz, 3H); $^{13}$C NMR (125 MHz, $CDCl_3$) δ 166.4, 159.2, 145.5, 130.0, 129.2 (2C), 123.2, 113.8 (2C), 80.7, 79.0, 77.8, 70.9, 64.5, 60.2, 55.2, 32.7, 32.5, 14.2; HRMS (ESI) *m/z* calcd for $C_{19}H_{26}O_6Na^+$ [(M + Na)$^+$] 373.1622, found 373.1620.

**Olefin 22.** To a solution of α,β-unsaturated ester **20** (922.3 mg, 2.632 mmol) and $Et_3N$ (1.45 mL, 10.5 mmol) in $CH_2Cl_2$/DMSO (1:1, v/v, 26.0 mL) at 0 °C was added $SO_3$•py (1.24 g, 7.79 mmol), and the resultant mixture was stirred at 0 °C for 75 min. The reaction was quenched with saturated aqueous $NH_4Cl$ solution, and the resultant mixture was diluted with EtOAc. After separation of the aqueous layer, the organic layer was washed with $H_2O$ and brine, dried ($MgSO_4$), filtered, and concentrated under reduced pressure. The crude aldehyde thus obtained was used in the next reaction immediately without further purification.

To a solution of sulfone **21** (1.49 g, 5.06 mmol) in DME (13.0 mL) at −50 °C were added KHMDS (0.5 M solution in toluene, 8.50 mL, 4.25 mmol) and a solution of the above crude aldehyde in DME (10.0 mL), and the resultant solution was stirred at −50 °C for 5 min and then allowed to warm to 0 °C over a period of 2 h. The reaction was quenched with saturated aqueous $NH_4Cl$ solution. The resultant mixture was diluted with EtOAc, washed with $H_2O$ and brine, dried ($MgSO_4$), filtered, and concentrated under reduced pressure. Purification of the residue by flash column chromatography (silica gel, 5 to 10% EtOAc/hexanes) gave olefin **22** (702.4 mg, 64%, *E/Z* 3:1 as judged by $^1$H NMR analysis) as a yellow oil. These *E*/*Z* isomers were separated by flash column chromatography ($AgNO_3$-impregnated silica gel, 10% EtOAc/hexanes) to give analytically pure *E* isomer (494.3 mg, 45%) and *Z* isomer (186.8 mg, 17%) as colorless oils. Data for the *E* isomer: $[\alpha]_D^{24}$ +33.7 (*c* 0.96, $CHCl_3$); IR (film) 2926, 2861, 1718, 1512, 1252, 1172, 1045 cm$^{-1}$; $^1$H NMR (500 MHz, $CDCl_3$) δ 7.24–7.22 (m, 2H), 6.93 (ddd, *J* = 15.5, 7.0, 7.0 Hz, 1H), 6.87–6.85 (m, 2H), 5.86 (ddd, *J* = 15.5, 1.5, 1.5 Hz, 1H), 5.66 (ddd, *J* = 15.5, 7.5, 7.5 Hz, 1H), 5.40 (dd, *J* = 15.5, 7.5 Hz, 1H), 4.54 (m, 1H), 4.54 (d, *J* = 11.5

Hz, 1H), 4.33 (d, $J$ = 11.5 Hz, 1H), 4.15 (q, $J$ = 7.5 Hz, 2H), 4.05 (ddd, $J$ = 6.5, 6.5, 4.0 Hz, 1H), 4.01 (dd, $J$ = 6.0, 4.0 Hz, 1H), 3.79 (s, 3H), 2.54 (ddd, $J$ = 7.0, 7.0, 1.5 Hz, 2H), 2.23 (ddd, $J$ = 13.5, 6.0, 2.0 Hz, 1H), 2.02–1.97 (m, 2H), 1.68 (ddd, $J$ = 13.5, 9.0, 4.0 Hz, 1H), 1.35 (quint, $J$ = 7.0 Hz, 2H), 1.28–1.23 (m, 4H), 1.26 (t, $J$ = 7.0 Hz, 3H), 0.86 (t, $J$ = 7.0 Hz, 3H); $^{13}$C NMR (125 MHz, $CDCl_3$) δ 166.5, 159.2, 145.7, 133.7, 130.1 (2C), 129.2 (2C), 123.1, 113.8 (2C), 80.3, 79.1, 78.4, 70.9, 60.1, 55.3, 38.2, 32.7, 32.2, 31.4, 28.7, 22.5, 14.3, 14.0; HRMS (ESI) *m/z* calcd for $C_{25}H_{36}O_5Na^+$ [(M + Na)$^+$] 439.2455, found 439.2433. Data for the *Z* isomer $[\alpha]_D^{25}$ +21.9 (*c* 1.09, $CHCl_3$); IR (film) 2926, 2861, 1718, 1514, 1252, 1173, 1045 cm$^{-1}$; $^1$H NMR (500 MHz, $CDCl_3$) δ 7.25–7.23 (m, 2H), 6.93 (ddd, $J$ = 15.5, 7.0, 7.0 Hz, 1H), 6.88–6.85 (m, 2H), 5.86 (d, $J$ = 15.5 Hz, 1H), 5.49 (ddd, $J$ = 11.0, 7.5, 7.5 Hz, 1H), 5.36 (dd, $J$ =11.0, 9.0 Hz, 1H), 4.91 (ddd, $J$ = 9.0, 9.0, 5.5 Hz, 1H), 4.54 (d, $J$ = 11.5 Hz, 1H), 4.35 (d, $J$ = 11.5 Hz, 1H), 4.15 (q, $J$ = 7.5 Hz, 2H), 4.07–4.01 (m, 2H), 3.79 (s, 3H), 2.55 (dd, $J$ = 7.0, 7.0 Hz, 2H), 2.23 (dd, $J$ = 1.35, 5.5 Hz, 1H), 2.11–2.00 (m, 2H), 1.63 (ddd, $J$ = 13.5, 9.0, 4.5 Hz, 1H), 1.38–1.23 (m, 6H), 1.24 (t, $J$ = 7.5 Hz, 3H), 0.86 (t, $J$ = 7.5 Hz, 3H); $^{13}$C NMR (125 MHz, $CDCl_3$) δ 166.5, 159.2, 145.7, 133.1, 130.2, 130.1, 129.3 (2C), 123.1, 113.8 (2C), 80.3, 79.3, 73.0, 71.0, 60.1, 55.3, 38.5, 32.7, 31.4, 29.3, 27.6, 22.5, 14.3, 14.0; HRMS (ESI) *m/z* calcd for $C_{25}H_{36}O_5Na^+$ [(M + Na)$^+$] 439.2455, found 439.2452.

**Alcohol 10.** To a solution of olefin **22** (350.0 mg, 0.8402 mmol) and $Et_3SiH$ (0.400 mL, 2.51 mmol) in $CH_2Cl_2$ (8.50 mL) at –35 °C was added $BF_3$•$OEt_2$ (0.160 mL, 1.27 mmol), and the resultant solution was allowed to warm to 0 °C over a period of 1 h. The reaction was quenched with saturated aqueous $NaHCO_3$ solution, and the resultant mixture was stirred vigorously at room temperature for 15 min. The resultant mixture was diluted with EtOAc, washed with $H_2O$ and brine, dried ($MgSO_4$), filtered, and concentrated under reduced pressure. Purification of the residue by flash column chromatography (silica gel, 15 to 20% EtOAc/hexanes) gave alcohol **10** (211.8 mg, 85%) as a colorless oil: $[\alpha]_D^{22}$ −6.8 (*c* 0.74, $CHCl_3$); IR (film) 3439, 2926, 2862, 1713, 1316, 1269, 1173, 1040 cm$^{-1}$; $^1$H NMR (500 MHz, $CDCl_3$) δ 6.97 (ddd, $J$ = 15.5, 7.0, 7.0 Hz, 1H), 5.92 (d, $J$ = 15.5 Hz, 1H), 5.67 (ddd, $J$ = 15.5, 7.0, 7.0

Hz, 1H), 5.40 (dd, *J* = 15.5, 7.0 Hz, 1H), 4.61 (br ddd, *J* = 9.0, 7.0, 7.0 Hz, 1H), 4.31 (m, 1H), 4.15 (q, *J* = 7.5 Hz, 2H), 3.97 (ddd, *J* = 7.0, 7.0, 3.0 Hz, 1H), 2.60–2.50 (m, 2H), 2.09 (dd, *J* = 13.0, 7.0 Hz, 1H), 2.02–1.97 (m, 2H), 1.86 (ddd, *J* = 13.0, 9.0, 5.0 Hz, 1H), 1.64 (d, *J* = 5.5 Hz, 1H), 1.35 (quint, *J* = 7.5 Hz, 2H), 1.29–1.23 (m, 4H), 1.25 (t, *J* = 7.5 Hz, 3H), 0.85 (t, *J* = 7.5 Hz, 3H); $^{13}$C NMR (125 MHz, $CDCl_3$) δ 166.4, 145.1, 133.6, 130.0, 123.4, 80.6, 78.3, 73.2, 60.3, 42.3, 32.4, 32.1, 31.4, 28.7, 22.5, 14.2, 14.0; HRMS (ESI) *m*/*z* calcd for $C_{17}H_{28}O_4Na^+$ [(M + Na)$^+$] 319.1880, found 319.1875.

**Ester 23.** To a solution of alcohol **10** (360.0 mg, 1.215 mmol) in toluene/γ-terpinene (1:1, v/v, 12.00 mL) was added **Co-II** (18.9 mg, 0.0360 mmol), and the resultant solution was stirred at 80 °C under an atmosphere of air for 4.5 h. The resultant solution was cooled to room temperature and concentrated under reduced pressure. Purification of the residue by flash column chromatography (silica gel, 15% *t*-BuOMe/hexanes) gave ester **23** (302.7 mg, 84%, d.r. >20:1) as a green oil: $[\alpha]_D^{23}$ +5.4 (*c* 0.47, $CHCl_3$); IR (film) 2928, 1736, 1149, 1083, 1035 cm$^{-1}$; $^1$H NMR (500 MHz, $C_6D_6$) δ 5.57 (ddd, *J* = 15.0, 7.0, 7.0 Hz, 1H), 5.39 (dd, *J* = 15.0, 5.0, 1H), 4.55 (m, 1H), 4.49 (ddd, *J* = 5.0, 5.0, 5.0 Hz, 1H), 4.45 (dd, *J* = 5.5, 5.5 Hz, 1H), 4.42 (m, 1H), 3.93 (q, *J* = 7.0 Hz, 2H), 2.46 (dd, *J* = 15.0, 7.5 Hz, 1H), 2.22 (dd, *J* = 15.0, 6.0 Hz, 1H), 2.15 (dd, *J* = 13.5, 5.5 Hz, 1H), 2.09 (dd, *J* = 13.0, 5.0 Hz, 1H), 1.91 (ddd, *J* = 7.0, 7.0, 7.0 Hz, 2H), 1.43 (ddd, *J* = 13.0, 10.0, 5.0 Hz, 1H), 1.34 (ddd, *J* = 13.5, 10.0, 4.5 Hz, 1H), 1.26 (quint, *J* = 7.5 Hz, 2H), 1.22–1.15 (m, 4H), 0.93 (t, *J* = 7.0 Hz, 3H), 0.84 (t, *J* = 7.0 Hz, 3H); $^{13}$C NMR (125 MHz, $C_6D_6$) δ 170.5, 132.6, 130.9, 84.2, 83.7, 80.8, 76.5, 60.2, 42.2, 41.4, 40.9, 32.5, 31.6, 29.1, 22.8, 14.20, 14.18; HRMS (ESI) *m*/*z* calcd for $C_{17}H_{28}O_4Na^+$ [(M + Na)$^+$] 319.1880, found 319.1865.

**Aldehyde S11.** To a solution of ester **23** (200.4 mg, 0.7436 mmol) in $CH_2Cl_2$ (7.00 mL) at −78 °C was slowly added DIBALH (1.03 M solution in *n*-hexane, 1.25 mL, 1.27 mmol), and the resultant solution was stirred at −78 °C for 35 min. The reaction was quenched with MeOH. The resultant solution was diluted with EtOAc and saturated aqueous potassium sodium tartrate solution and stirred vigorously

at room temperature for 7.5 h. The aqueous layer was separated, and the organic layer was washed with brine, dried ($MgSO_4$), filtered, and concentrated under reduced pressure. Purification of the residue by flash column chromatography (silica gel, 20% EtOAc/hexanes) gave aldehyde **S11** (176.2 mg, 94%) as a colorless oil: $[\alpha]_D^{23}$ +10.4 (*c* 1.11, $CHCl_3$); IR (film) 2926, 2857, 1726, 1084, 1035, 970 $cm^{-1}$; $^1H$ NMR (500 MHz, $CDCl_3$) δ 9.77 (br t, *J* = 1.5 Hz, 1H), 5.70 (ddd, *J* = 15.0, 7.0, 7.0 Hz, 1H), 5.33 (dd, *J* = 15.0, 7.0 Hz, 1H), 4.71 (m, 2H), 4.49 (dddd, *J* = 12.0, 10.5, 7.0, 5.0 Hz, 1H), 4.42 (ddd, *J* = 10.0, 7.0, 5.0 Hz, 1H), 2.65–2.59 (m, 2H), 2.99 (dd, *J* = 13.5, 5.0 Hz, 1H), 2.17 (dd, *J* = 13.5, 5.0 Hz, 1H), 1.99 (ddd, *J* = 7.0 Hz, 2H), 1.73–1.62 (m, 2H), 1.34 (quint, *J* = 7.0 Hz, 2H), 1.28–1.21 (m, 4H), 0.85 (t, 7.0 Hz, 3H); $^{13}C$ NMR (125 MHz, $CDCl_3$) δ 200.6, 134.4, 129.3, 84.2, 83.3, 81.0, 74.6, 49.2, 41.6, 41.2, 32.1, 31.3, 28.6, 22.5, 14.0; HRMS (ESI) *m*/*z* calcd for $C_{15}H_{25}O_3^+$ [$(M + H)^+$] 253.1798, found 253.1778.

**Iodoolefin 5.** To a suspension of $CrCl_2$ (816.8 mg, 6.646 mmol) in 1,4-dioxane/THF (6:1, v/v, 4.20 mL) at 0 °C was added a solution of aldehyde **S11** (167.3 mg, 0.6634 mmol) and $CHI_3$ (786.4 mg, 1.997 mmol) in 1,4-dioxane/THF (6:1, v/v, 1.40 mL + 1.40 mL), and the resultant mixture was stirred at room temperature for 3.5 h. The reaction was quenched with saturuated aqueous $Na_2SO_3$ solution. The resultant mixture was diluted with *t*-BuOMe and washed with $H_2O$ and brine. The aqueous layers were extracted with *t*-BuOMe. The organic layers were combined, dried ($MgSO_4$), filtered, and concentrated under reduced pressure. Purification of the residue by flash column chromatography (silica gel, 5 to 10% *t*-BuOMe/hexanes) gave iodoolefin **5** (213.8 mg, 85%, *E*/*Z* 4:1) as a colorless oil. These *E*/*Z* isomers were separated by flash column chromatography (silica gel, 3% *t*-BuOMe/hexanes) to give analytically pure *E* isomer (111.3 mg, 45%) and *Z* isomer (39.1 mg, 15%) as colorless oils. Data for the *E* isomer: $[\alpha]_D^{24}$ +4.3 (*c* 0.53, $CHCl_3$); IR (film) 2925, 2861, 1083,1038, 957 $cm^{-1}$; $^1H$ NMR (500 MHz, $C_6D_6$) δ 6.42 (ddd, *J* = 14.0, 7.5, 7.5 Hz, 1H), 5.75 (ddd, *J* = 14.0, 1.5, 1.5 Hz, 1H), 5.65 (ddd, *J* = 15.5, 7.5, 7.5 Hz, 1H), 5.44 (dd, *J* = 15.5, 5.0 Hz, 1H), 4.42 (ddd, *J* = 10.5, 5.0, 5.0 Hz, 1H), 4.39 (dd, *J* = 5.0, 5.0 Hz, 1H), 4.33 (dd, *J* = 5.0, 5.0 Hz, 1H), 3.85 (dddd, *J* = 11.0, 5.5, 5.5, 5.5

Hz, 1H), 2.04 (dd, $J$ = 13.0, 5.0 Hz, 1H), 1.94 (ddd, $J$ = 7.5, 7.5, 7.5 Hz, 2H), 1.87 (m, 1H), 1.87 (dd, $J$ = 13.5, 5.5 Hz, 1H), 1.79 (dddd, $J$ = 14.0, 7.5, 5.5, 1.5 Hz, 1H), 1.43 (ddd, $J$ = 13.0, 10.0, 5.0 Hz, 1H), 1.29 (quint, $J$ = 7.5 Hz, 2H), 1.25–11.7 (m, 4H), 1.12 (ddd, $J$ = 13.5, 11.0, 5.0 Hz, 1H), 0.85 (t, $J$ = 7.0 Hz, 3H); $^{13}$C (125 MHz, $C_6D_6$) δ 142.8, 132.5, 131.0, 84.1, 83.7, 80.8, 78.4, 77.1, 42.3, 41.7, 40.8, 32.5, 31.6, 29.2, 22.8, 14.2; HRMS (ESI) *m*/*z* calcd for $C_{16}H_{26}O_2I^+$ [(M + H)$^+$] 377.0972, found 377.0955. Data for the *Z* isomer $[\alpha]_D^{25}$ +1.6 (*c* 0.51, $CHCl_3$); IR (film) 2925, 2856, 1083, 969 cm$^{-1}$; $^1$H NMR (500 MHz, $C_6D_6$) δ 6.00–5.93 (m, 2H), 5.64 (ddd, $J$ = 15.5, 7.0, 7.0 Hz, 1H), 5.43 (dd, $J$ = 15.5, 6.5 Hz, 1H), 4.45 (ddd, $J$ = 11.0, 6.5, 6.5 Hz, 1H), 4.43 (dd, $J$ = 4.5, 4.5 Hz, 1H), 4.38 (dd, $J$ = 4.5, 4.5 Hz, 1H), 4.04 (dddd, $J$ = 11.5, 6.0, 6.0, 6.0 Hz, 1H), 2.26 (dd, $J$ = 6.0, 6.0 Hz, 2H), 2.08 (dd, $J$ = 13.0, 6.5 Hz, 1H), 1.96 (dd, $J$ = 13.5, 6.0 Hz, 1H), 1.93 (ddd, $J$ = 7.0, 7.0, 7.0 Hz, 2H), 1.45 (ddd, $J$ = 13.0, 11.0, 4.5 Hz, 1H), 1.29 (quint, $J$ = 7.0 Hz, 2H), 1.27 (m, 1H), 1.23–1.16 (m, 4H), 0.85 (t, $J$ = 7.0 Hz, 3H); $^{13}$C NMR (125 MHz, $C_6D_6$) δ 138.0, 132.5, 131.0, 84.11, 84.06, 83.8, 80.8, 78.4, 42.3, 41.0, 40.9, 32.5, 31.6, 29.2, 22.8, 14.2; HRMS (ESI) *m*/*z* calcd for $C_{16}H_{26}O_2I^+$ [(M + H)$^+$] 377.0972, found 377.0963.

## 6. Synthesis of iodoolefin *ent*-5

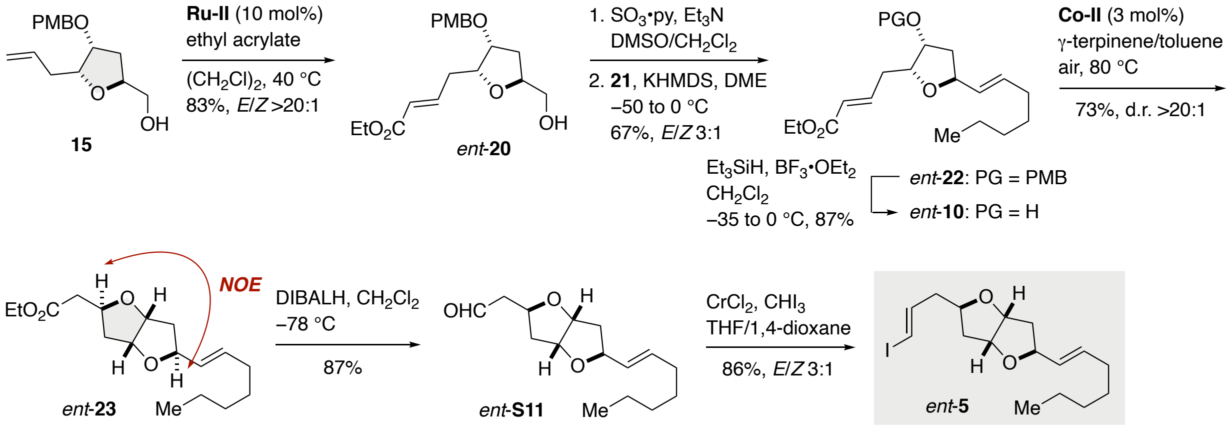


**Scheme S5** Synthesis of Iodoolefin *ent*-**5**.

**α,β-Unsaturated ester *ent*-20.** According to the procedure described for **20**, alcohol **15** (28.4 mg, 0.102 mmol) was converted to α,β-unsaturated ester *ent*-**20** (29.8 mg, 83%, *E*/*Z* >20:1) as a brown oil: $[\alpha]_D^{25}$ −34.7 (*c* 0.43, $CHCl_3$). The $^1H$ and $^{13}C$ NMR data were in accordance with those reported for α,β-unsaturated ester **20**.

**Olefin *ent*-22.** According to the procedure described for olefin **22**, α,β-unsaturated ester *ent*-**20** (295.3 mg, 0.8427 mmol) was converted to olefin *ent*-**22** (270.3 mg, 67 % for the two steps, *E*/*Z* 3:1) as a colorless oil. Data for the *E* isomer: $[\alpha]_D^{23}$ −34.9 (*c* 0.96, $CHCl_3$). The $^1H$ and $^{13}C$ NMR data were in accordance with those reported for olefin **22** (*E* isomer). Data for the *Z* isomer: $[\alpha]_D^{23}$ −22.9 (*c* 1.09, $CHCl_3$). The $^1H$ and $^{13}C$ NMR data were in accordance with those reported for olefin **22** (*Z* isomer).

**Alcohol *ent*-10.** According to the procedure described for alcohol **10**, olefin *ent*-**22** (281.7 mg, 0.6763 mmol) was converted to alcohol *ent*-**10** (172.7 mg, 87%) as a colorless oil: $[\alpha]_D^{23}$ +7.4 (*c* 0.74, $CHCl_3$). The $^1H$ and $^{13}C$ NMR data were in accordance with those reported for alcohol **10**.

**Ester *ent*-23.** According to the procedure described for ester **23**, alcohol *ent*-**10** (165.0 mg, 0.5567

mmol) was converted to ester *ent*-**23** (116.9 mg, 73%, d.r. >20:1) as a yellow oil: $[\alpha]_D^{20}$ −5.9 (*c* 0.47, $CHCl_3$). The $^1H$ and $^{13}C$ NMR data were in accordance with those reported for ester **23**.

**Aldehyde *ent*-S11.** According to the procedure described for aldehyde **S11**, ester *ent*-**23** (32.2 mg, 0.109 mmol) was converted to aldehyde *ent*-**S11** (23.9 mg, 87%) as a colorless oil: $[\alpha]_D^{18}$ –14.5 (*c* 1.11, $CHCl_3$). The $^1H$ and $^{13}C$ NMR data were in accordance with those reported for aldehyde **S11**.

**Iodoolefin *ent*-5.** According to the procedure described for iodoolefin **5**, aldehyde *ent*-**S11** (25.7 mg, 0.102 mmol) was converted to iodoolefin *ent*-**5** (32.3 mg, 86%, *E*/*Z* 3:1) as a colorless oil. Data for the *E* isomer: $[\alpha]_D^{22}$ −4.2 (*c* 0.53, $CHCl_3$). The $^1H$ and $^{13}C$ NMR data were in accordance with those reported for iodoolefin **5** (*E* isomer). Data for the *Z* isomer: $[\alpha]_D^{22}$ −1.5 (*c* 0.51, $CHCl_3$). The $^1H$ and $^{13}C$ NMR data were in accordance with those reported for iodoolefin **5** (*Z* isomer).

## 7. Optimization of Stille-type reaction of vinylstannane 4 and iodoolefin 5

**Table S3** Screening of reaction conditions.[a]

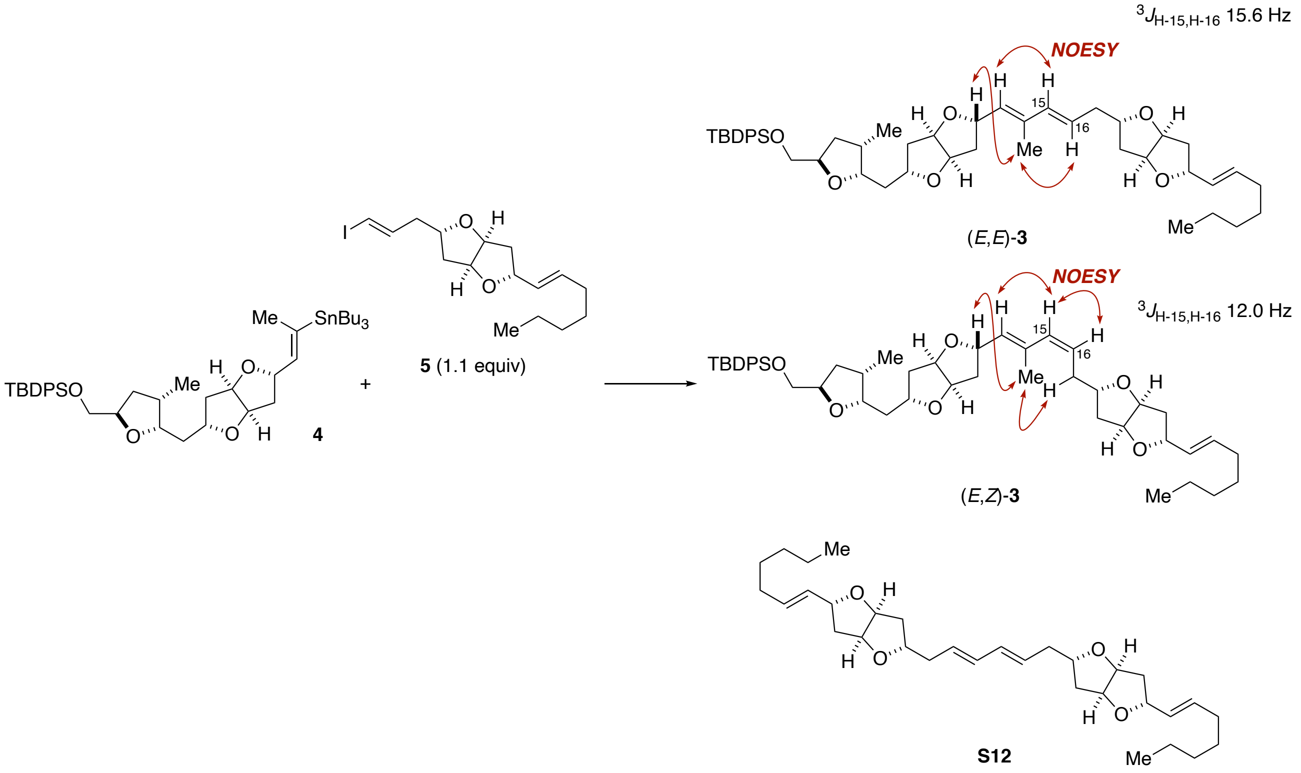


| Entry | Reagents and Conditions (mol%) | Yield (%) | | | |
|---|---|---|---|---|---|
| | | **3**[b] | (*E*,*E*)/(*E*,*Z*)[c] | **S12**[d] | Recov. **4** |
| 1 | $Pd_2(dba)_3$•$CHCl_3$ (10), $Ph_3As$ (80) DMSO/THF (1:1, v/v), rt | 4 | 1:1 | 18 | 86 |
| 2[e] | $Pd_2(dba)_3$•$CHCl_3$ (9), $Ph_3As$ (80), CuI (100) DMSO/THF (1:1, v/v), rt to 40 °C | 30 | 1:7 | 39 | 8 |
| 3 | $Pd_2(dba)_3$•$CHCl_3$ (9), $Ph_3As$ (80), CuTC (150) DMSO/THF (1:1, v/v), rt | 30 | 3:1 | 22 | 31 |
| 4 | CuTC (400), NMP, rt | 83 | >20:1 | 16 | 6 |

[a]All reactions were performed using vinylstannane **4** (1 equiv) and iodoolefin **5** (1.1 equiv) in dry, degassed solvents at indicated temperature for 24 h. [b]Combined yield of (*E*,*E*)-**3** and (*E*,*Z*)-**3**. [c]Estimated by $^1$H NMR analysis of purified mixture. [d]Containing unidentified side product(s).

Stille-type reaction of vinylstannane **4** (1 equiv) and nearly equimolar amount of iodoolefin **5** (1.1 equiv) required optimization experiments. Our initial attempt was to run the reaction under the influence of $Pd_2(dba)_3$•$CHCl_3$/$Ph_3As$[6] in DMSO/THF (1:1) at room temperature for 24 h (entry 1). However, the reaction did not proceed appreciably and the coupling product **3** was isolated in only 4% yield as an inseparable 1:1 mixture of (*E*,*E*)- and (*E*,*Z*)-isomers. As a side product, diene **S12**, presumably arose from homo-coupling of **5**, was also obtained in ca. 18% yield. The side product **S12** could not be isolated in a pure form and the structure was tentatively assigned on the basis of $^1$H NMR and ESIMS data of crude mixture. To overcome the low reactivity of **4** and **5**, we examined the use of copper(I) salt as an additive to accelerate the reaction. Actually, running the reaction in the presence of $Pd_2(dba)_3$•$CHCl_3$/$Ph_3As$ with CuI[7] in DMSO/THF (1:1) at room temperature to 40 °C effectively consumed **4** and provided **3** in 30% yield (entry 2). In this case, however, significant isomerization of the C15–C16 double bond occurred and **3** was obtained as a 1:7 mixture of (*E*,*E*)- and (*E*,*Z*)-isomers. Also, **S12** was obtained in ca. 39% yield. Changing CuI to CuTC proved to be beneficial, as the reaction by the action of $Pd_2(dba)_3$•$CHCl_3$/$Ph_3As$ with CuTC in DMSO/THF at room temperature[8] afforded **3** in 30% yield as a 3:1 mixture of (*E*,*E*)- and (*E*,*Z*)-isomers (entry 3). It was finally found that the coupling of **4** and **5** could be most effectively performed by using a *stoichiometric* amount of CuTC in NMP at room temperature[9] (entry 4). These conditions have been successfully applied to Stille-type reactions using vinylstannanes with low reactivity. After 24 h, **3** was isolated in an excellent 83% yield as an essentially single stereoisomer, as judged by $^1$H NMR analysis. The configuration of the double bonds of (*E*,*E*)-**3** and (*E*,*Z*)-**3** was confirmed by NOESY correlations and $^3J_{H,H}$ values, as shown.

---

[6] V. Farina and B. Krishnan, *J. Am. Chem. Soc.*, 1991, **113**, 9585–9595.
[7] V. Farina, S. Kapadia, B. Krishnan, C. Wang and L. S. Liebeskind, *J. Org. Chem.*, 1994, **59**, 5905–5911.
[8] H. Fuwa, M. Ebine, A. J. Bourdelais, D. G. Baden and M. Sasaki, *J. Am. Chem. Soc.*, 2006, **128**, 16989–16999.
[9] H. Fuwa, N. Yamagata, Y. Okuaki, Y. Ogata, A. Saito and M. Sasaki, *Chem. Eur. J.*, 2016, **22**, 6815–6829.

## 8. Total synthesis of putative structures 1 and 2 of amphirionin-2

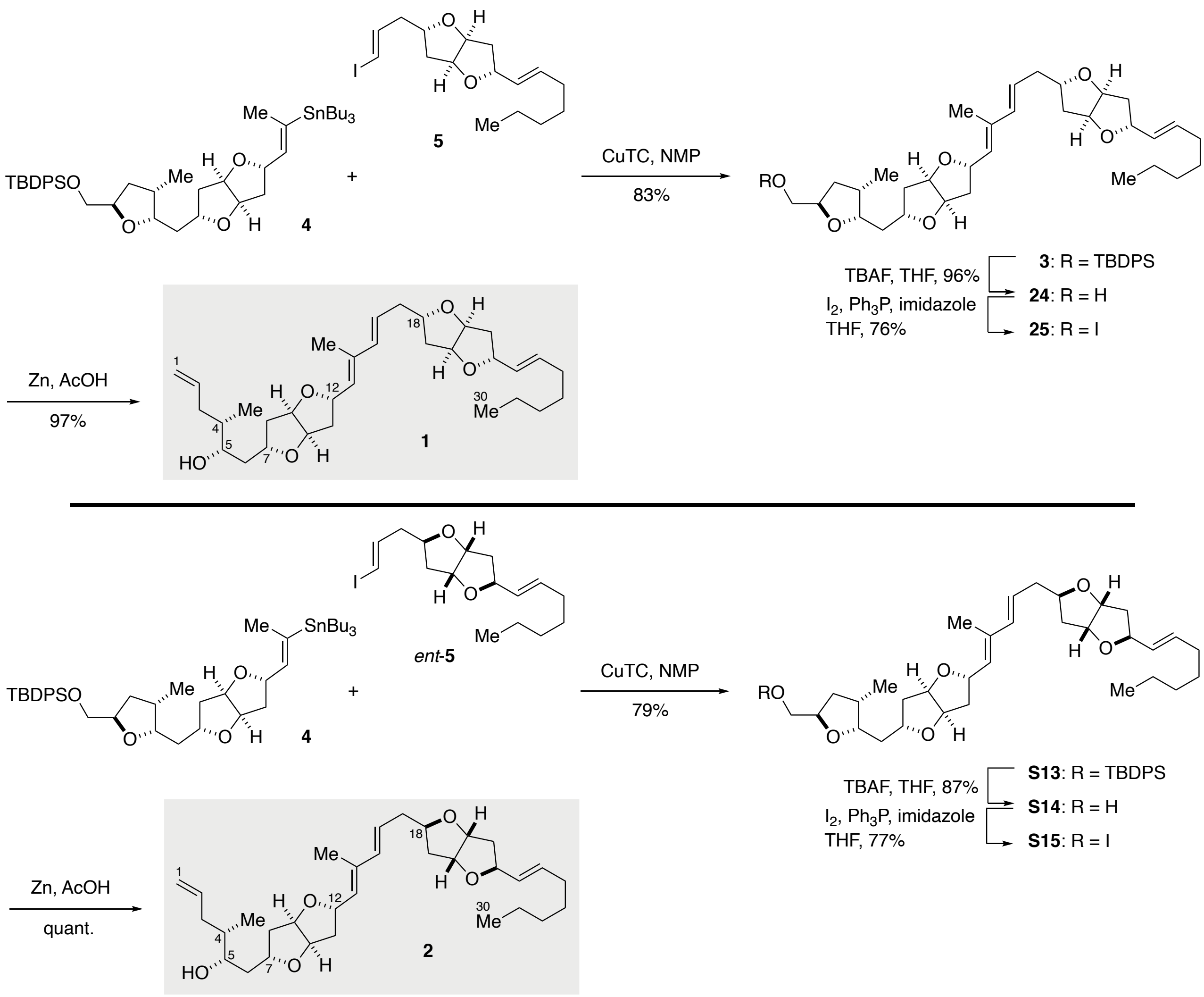


**Scheme S6** Synthesis of putative structures **1** and **2** of amphirionin-2.

**Diene 3.** To a solution of vinylstannane **4** (38.7 mg, 0.0478 mmol) and iodoolefin **5** (20.2 mg, 0.0537 mmol) in degassed NMP (1.00 mL) was added CuTC (36.2 mg, 0.190 mmol), and the resultant mixture was stirred at room temperature for 24 h. The reaction was quenched with 5% $NH_4OH$ solution. The resultant mixture was stirred at room temperature for 30 min and then extracted with EtOAc. The organic layer was washed with brine, dried ($Na_2SO_4$), filtered, and concentrated under reduced pressure. Purification of the residue by flash column chromatography (silica gel, 10 to 30% *t*-BuOMe/hexanes) gave diene **3** (30.6 mg, 83%) as a colorless oil: $[\alpha]_D^{24}$ −6.0 (*c* 1.00, $CHCl_3$); IR (film)

2928, 2858, 1430, 1104, 1090, 705 $cm^{-1}$; $^1$H NMR (400 MHz, $CDCl_3$) δ 7.68–7.65 (m, 4H), 7.39–7.33 (m, 6H), 6.08 (d, *J* = 15.6 Hz, 1H), 5.70 (ddd, *J* = 15.2, 6.8, 6.8 Hz, 1H), 5.61 (ddd, *J* = 15.6, 6.8, 6.8 Hz, 1H), 5.34 (dd, *J* = 15.2, 7.2 Hz, 1H), 5.29 (d, *J* = 8.2 Hz, 1H), 4.82 (ddd, *J* =10.0, 8.4, 4.8 Hz, 1H), 4.72–4.66 (m, 4H), 4.40 (ddd, *J* = 10.4, 7.2, 5.2 Hz, 1H), 4.25 (dddd, *J* = 12.0, 6.8, 5.2, 5.2 Hz, 1H), 4.19 (dddd, *J* = 7.2, 7.2, 4.4, 4.4 Hz, 1H), 4.11 (dddd, *J* = 11.2, 6.0, 6.0, 6.0 Hz, 1H), 3.96 (ddd, *J* = 9.2, 4.4, 4.4 Hz, 1H), 3.63 (dd, *J* = 10.4, 4.4 Hz, 1H), 3.59 (dd, *J* = 10.4, 4.4 Hz, 1H), 2.36 (ddd, *J* = 13.6, 6.8, 6.0 Hz, 1H), 2.30–2.21 (m, 3H), 2.20–2.10 (m, 3H), 2.03 (m, 1H), 2.00 (ddd, *J* = 6.8, 6.8, 6.8 Hz, 2H), 1.88 (ddd, *J* = 13.6, 9.2, 5.2 Hz, 1H), 1.75 (s, 3H), 1.71–1.58 (m, 5H), 1.51 (ddd, *J* = 13.6, 6.8, 4.4 Hz, 1H), 1.34 (q, *J* = 6.8 Hz, 2H), 1.30–1.22 (m, 4H), 1.03 (s, 9H), 0.90 (d, *J* = 7.2 Hz, 3H), 0.85 (t, *J* = 6.8 Hz, 3H); $^{13}$C NMR (100 MHz, $CDCl_3$) δ 136.4, 136.1, 135.6 (4C), 134.2, 133.59, 133.56, 130.2, 129.54, 129.51, 129.45, 127.6 (4C), 125.1, 83.9, 83.72, 83.68, 83.59, 80.9, 79.6, 78.6, 78.3, 77.2, 76.2, 66.5, 42.0, 41.8, 40.9, 40.6, 38.7, 36.5, 36.4, 35.7, 32.1, 31.3, 28.6, 26.8 (3C), 22.5, 19.2, 14.3, 14.0, 13.0; HRMS (ESI) *m/z* calcd for $C_{48}H_{68}O_6SiNa^+$ [$(M + Na)^+$] 791.4677, found 791.4695.

**Alcohol 24.** To a solution of diene **3** (10.4 mg, 0.0135 mmol) in THF (1.00 mL) was added TBAF (1.0 M solution in THF, 0.140 mL, 0.140 mmol), and the resultant solution was stirred at room temperature for 2 h. The reaction was quenched with saturated aqueous $NH_4Cl$ solution. The resultant mixture was extracted with EtOAc, and the organic layer was washed with $H_2O$ and brine, dried ($Na_2SO_4$), filtered, and concentrated under reduced pressure. Purification of the residue by flash column chromatography (silica gel, 60 to 80% EtOAc/hexanes) gave alcohol **24** (6.9 mg, 96%) as a colorless oil: $[\alpha]_D^{22}$ −10.7 (*c* 0.20, $CHCl_3$); IR (film) 3446, 2926, 2870, 1435, 1378, 1084, 1039, 968 $cm^{-1}$; $^1$H NMR (400 MHz, $CDCl_3$) δ 6.08 (d, *J* = 15.6 Hz, 1H), 5.69 (ddd, *J* = 15.6, 6.8, 6.8 Hz, 1H), 5.63 (ddd, *J* = 15.6, 6.8, 6.8 Hz, 1H), 5.34 (dd, *J* = 15.6, 7.6 Hz, 1H), 5.30 (d, *J* = 8.4 Hz, 1H), 4.82 (ddd, *J* = 10.4, 8.4, 5.2 Hz, 1H), 4.72–4.71 (m, 2H), 4.68–4.66 (m, 2H), 4.39 (ddd, *J* = 10.0, 7.6, 5.2 Hz, 1H), 4.24–4.15 (m, 2H), 4.10 (dddd, *J* = 10.8, 5.6, 5.6, 5.6 Hz, 1H), 3.94 (ddd, *J* = 8.8, 4.8, 4.8 Hz, 1H), 3.59 (dd, *J* = 11.6, 3.2

Hz, 1H), 3.44 (dd, *J* = 11.6, 6.4 Hz, 1H), 2.40–2.16 (m, 6H), 2.14 (dd, *J* = 13.6, 13.6, 4.8 Hz, 1H), 1.99 (ddd, *J* = 6.8, 6.8, 6.8 Hz, 2H), 1.91–1.78 (m, 2H), 1.77 (s, 3H), 1.71–1.57 (m, 5H), 1.50 (ddd, *J* = 13.6, 6.8, 4.8 Hz, 1H), 1.34 (q, *J* = 6.8 Hz, 2H), 1.26–1.22 (m, 4H), 0.91 (d, *J* = 6.8 Hz, 3H), 0.85 (t, *J* = 6.8 Hz, 3H), one proton missing due to H/D exchange; $^{13}$C NMR (100 MHz, $CDCl_3$) δ 136.4, 136.2, 134.3, 130.1, 129.5, 125.3, 84.0, 83.8, 83.6 (2C), 80.9, 79.7, 78.7, 78.1, 77.2, 76.4, 65.5, 41.9, 41.8, 41.1, 40.6, 38.7, 36.3, 36.2, 35.3, 32.2, 31.4, 28.7, 22.5, 14.3, 14.0, 13.1; HRMS (ESI) *m*/*z* calcd for $C_{32}H_{50}O_6Na^+$ [(M + Na)$^+$] 553.3500, found 553.3509.

**Iodide 25.** To a solution of alcohol **24** (3.7 mg, 0.0070 mmol) in THF (0.500 mL) were added imidazole (10.7 mg, 0.157 mmol), $Ph_3P$ (33.6 mg, 0.128 mmol), and $I_2$ (28.4 mg, 0.112 mmol), and the resultant mixture was stirred at room temperature for 1 h. The reaction was quenched with saturated aqueous $Na_2SO_3$ solution. The resultant mixture was extracted with EtOAc, and the organic layer was washed with $H_2O$ and brine, dried ($Na_2SO_4$), filtered, and concentrated under reduced pressure. Purification of the residue by flash column chromatography (silica gel, 0 to 20% EtOAc/hexanes) gave iodide **25** (3.4 mg, 76%) as a colorless oil: $[\alpha]_D^{23}$ +5.2 (*c* 0.34, $CHCl_3$); IR (film) 2926, 2864, 1433, 1155, 1083, 1038, 968 cm$^{-1}$; $^1$H NMR (400 MHz, $CDCl_3$) δ 6.08 (d, *J* = 15.6 Hz, 1H), 5.69 (ddd, *J* = 15.2, 6.8, 6.8 Hz, 1H), 5.62 (ddd, *J* = 15.6, 6.8, 6.8 Hz, 1H), 5.34 (dd, *J* = 15.2, 7.6 Hz, 1H), 5.30 (d, *J* = 8.4 Hz, 1H), 4.82 (ddd, *J* = 10.0, 8.4, 5.2 Hz, 1H), 4.74–4.72 (m, 2H), 4.68–4.66 (m, 2H), 4.40 (ddd, *J* = 10.0, 7.6, 4.8 Hz, 1H), 4.23–4.04 (m, 4H), 3.25 (dd, *J* = 9.6, 4.4 Hz, 1H), 3.14 (dd, *J* = 9.6, 7.6 Hz, 1H), 2.38–2.15 (m, 6H), 2.14 (ddd, *J* = 14.0, 14.0, 5.2 Hz, 1H), 1.99 (ddd, *J* = 6.8, 6.8, 6.8 Hz, 2H), 1.89–1.80 (m, 2H), 1.78 (s, 3H), 1.72–1.47 (m, 5H), 1.50 (ddd, *J* = 14.0, 6.8, 4.8 Hz, 1H), 1.34 (q, *J* = 6.8 Hz, 2H), 1.27–1.23 (m, 4H), 0.91 (d, *J* = 7.2 Hz, 3H), 0.85 (t, *J* = 6.8 Hz, 3H); $^{13}$C NMR (100 MHz, $CDCl_3$) δ 136.4, 136.2, 134.3, 130.1, 129.5, 125.2, 84.0, 83.8, 83.66, 83.63, 80.9, 79.7, 79.2, 78.0, 76.4, 76.3, 41.9, 41.8, 40.9, 40.6, 40.3, 38.7, 36.8, 36.1, 32.2, 31.4, 28.7, 22.5, 14.3, 14.0, 13.1, 12.0; HRMS (ESI) *m*/*z* calcd for $C_{32}H_{49}O_5INa^+$ [(M + Na)$^+$] 663.2517, found 663.2526.

**Putative structure 1 of amphirionin-2.** To a solution of iodide **25** (2.3 mg, 0.0036 mmol) in AcOH (0.50 mL) was added freshly activated zinc dust (26.0 mg, 0.398 mmol), and the resultant mixture was stirred at room temperature for 6 h. The reaction mixture was filtered through a pad of Celite, and the filtrate was neutralized with saturated aqueous $NaHCO_3$ solution at 0 °C. The resultant mixture was extracted with EtOAc. The organic layer was washed with $H_2O$ and brine, dried ($Na_2SO_4$), filtered, and concentrated under reduced pressure. Purification of the residue by flash column chromatography (silica gel, 25% EtOAc/hexanes) gave putative structure **1** of amphirionin-2 (1.8 mg, 97%) as a colorless oil: $[\alpha]_D^{23}$ −2.4 (*c* 0.22, $CHCl_3$); IR (film) 3518, 2926, 2863, 1726, 1436, 1081, 1037, 971, 915 $cm^{-1}$; $^1H$ NMR (500 MHz, $C_6D_6$, undeuterated solvent as internal standard: 7.20 ppm) δ 6.22 (d, *J* = 15.5 Hz, 1H), 5.92 (dddd, *J* = 16.5, 10.0, 7.5, 7.5 Hz, 1H), 5.78 (ddd, *J* = 15.5, 7.0, 7.0 Hz, 1H), 5.70 (ddd, *J* = 15.5, 6.5, 6.5 Hz, 1H), 5.53 (d, *J* = 8.0 Hz, 1H), 5.51 (dd, *J* = 15.5, 6.5 Hz, 1H), 5.18 (dd, *J* = 16.5, 1.5 Hz, 1H), 5.11 (dd, *J* = 10.0, 1.5 Hz, 1H), 4.91 (ddd, *J* = 10.0, 8.0, 5.0 Hz, 1H), 4.58 (m, 1H), 4.56 (dd, *J* = 4.5, 4.5 Hz, 1H), 4.54 (dd, *J* = 4.5, 4.5 Hz, 1H), 4.43 (dd, *J* = 4.5, 4.5 Hz, 1H), 4.33 (dd, *J* = 4.5, 4.5 Hz, 1H), 4.19 (dddd, *J* = 10.0, 5.5, 5.5, 5.5 Hz, 1H), 4.13 (m, 1H), 3.83 (br d, *J* = 10.0 Hz, 1H), 3.55 (s, 1H), 2.55 (ddd, *J* = 14.0, 7.5, 7.5 Hz, 1H), 2.38 (ddd, *J* = 14.0, 7.0, 5.5 Hz, 1H), 2.25 (ddd, *J* = 14.0, 7.0, 5.5 Hz, 1H), 2.21 (dd, *J* = 13.0, 5.0 Hz, 1H), 2.12 (dd, *J* = 13.0, 5.5 Hz, 1H), 2.08 (ddd, *J* = 14.0, 7.5, 7.5 Hz, 1H), 2.06–2.01 (m, 2H), 1.98 (q, *J* = 6.5 Hz, 2H), 1.75 (s, 3H), 1.61 (m, 1H), 1.55 (ddd, *J* = 13.0, 9.5, 4.5 Hz, 1H), 1.46–1.37 (m, 3H), 1.37–1.30 (m, 3H), 1.30–1.21 (m, 5H), 1.07 (d, *J* = 7.0 Hz, 3H), 0.90 (t, *J* = 7.0 Hz, 3H); $^{13}C$ NMR (125 MHz, $C_6D_6$) δ 138.3, 136.7, 135.7, 132.5, 131.4, 131.1, 126.0, 115.9, 84.6, 84.1, 83.9, 83.0, 81.5, 80.9, 79.9, 76.6, 74.4, 42.5, 42.4, 42.2, 41.3, 40.1, 39.4, 39.3, 38.1, 32.5, 31.6, 29.2, 22.8, 14.2, 14.1, 13.1; HRMS (ESI) *m*/*z* calcd for $C_{32}H_{50}O_5Na^+$ $[(M + Na)^+]$ 537.3551, found 537.3551.

**Diene S13.** To a solution of vinylstannane **4** (37.6 mg, 0.0464 mmol) and iodoolefin *ent*-**5** (18.7 mg, 0.0497 mmol) in degassed NMP (1.00 mL) was added CuTC (36.4 mg, 0.191 mmol), and the resultant mixture was stirred at room temperature for 13 h. The reaction was quenched with 5% $NH_4OH$ solution,

and the resultant mixture was stirred at room temperature for 30 min. The resultant mixture was extracted with EtOAc. The organic layer was washed with brine, dried ($Na_2SO_4$), filtered, and concentrated under reduced pressure. Purification of the residue by flash column chromatography (silica gel, 20 to 30% *t*-BuOMe/hexanes) gave diene **S13** (28.2 mg, 79%) as a colorless oil: $[\alpha]_D^{23}$ −4.6 (*c* 1.00, $CHCl_3$); IR (film) 2928, 2856, 1428, 1108, 1088, 965, 704 cm$^{-1}$; $^1$H NMR (500 MHz, $CDCl_3$) δ 7.68–7.65 (m, 4H), 7.41–7.34 (m, 6H), 6.08 (d, *J* = 16.0 Hz, 1H), 5.70 (ddd, *J* = 15.0, 7.0, 7.0 Hz, 1H), 5.61 (ddd, *J* = 16.0, 7.0, 7.0 Hz, 1H), 5.34 (dd, *J* = 15.0, 7.5 Hz, 1H), 5.29 (d, *J* = 8.5 Hz, 1H), 4.82 (ddd, *J* = 10.0, 8.5, 5.0 Hz, 1H), 4.72–4.67 (m, 4H), 4.40 (ddd, *J* = 10.5, 7.5, 5.0, 5.0 Hz, 1H), 4.25 (dddd, *J* = 10.5, 7.5, 5.5, 5.5 Hz, 1H), 4.19 (dddd, *J* = 7.5, 7.5, 4.5, 4.5 Hz, 1H), 4.10 (dddd, *J* = 11.0, 5.5, 5.5, 5.5 Hz, 1H), 3.96 (ddd, *J* = 9.0, 4.5, 4.5 Hz, 1H), 3.63 (dd, *J* = 10.5, 4.5 Hz, 1H), 3.60 (dd, *J* = 10.5, 4.5 Hz, 1H), 2.37 (ddd, *J* = 14.0, 7.0, 5.5 Hz, 1H), 2.29–2.22 (m, 3H), 2.19–2.11 (m, 3H), 2.04 (ddd, *J* = 12.0, 7.5, 7.5 Hz, 1H), 1.99 (dddd, *J* = 7.0, 7.0, 7.0, 7.0 Hz, 2H), 1.88 (ddd, *J* = 14.0, 9.0, 5.5 Hz, 1H), 1.75 (s, 3H), 1.71–1.58 (m, 5H), 1.51 (ddd, *J* = 14.0, 7.5, 4.5 Hz, 1H), 1.35 (quint, *J* = 7.0 Hz, 2H), 1.29–1.23 (m, 4H), 1.03 (s, 9H), 0.90 (d, *J* = 7.5 Hz, 3H), 0.85 (t, *J* = 7.0 Hz, 3H); $^{13}$C NMR (125 MHz, $CDCl_3$) δ 136.5, 136.1, 135.6 (4C), 134.2, 133.7 (2C), 130.3, 129.56, 129.54, 129.52, 127.6 (4C), 125.2, 84.0, 83.8, 83.7, 83.6, 80.9, 79.7, 78.6, 78.4, 77.4, 76.3, 66.5, 42.0, 41.8, 40.9, 40.7, 38.9, 36.5, 36.4, 35.7, 32.1, 31.4, 28.7, 26.8 (3C), 22.5, 19.2, 14.3, 14.0, 13.0; HRMS (ESI) *m*/*z* calcd for $C_{48}H_{68}O_6SiNa^+$ [(M + Na)$^+$] 791.4677, found 791.4678.

**Alcohol S14.** To a solution of diene **S13** (8.7 mg, 0.011 mmol) in THF (1.00 mL) was added TBAF (1.0 M solution in THF, 0.110 mL, 0.110 mmol), and the resultant solution was stirred at room temperature for 2 h. The reaction was quenched with saturated aqueous $NH_4Cl$ solution. The resultant mixture was extracted with EtOAc, and the organic layer was washed with $H_2O$ and brine, dried ($Na_2SO_4$), filtered, and concentrated under reduced pressure. Purification of the residue by flash column chromatography (silica gel, 60 to 70% EtOAc/hexanes) gave alcohol **S14** (5.2 mg, 87%) as a colorless oil: $[\alpha]_D^{23}$ −9.8 (*c* 0.27, $CHCl_3$); IR (film) 3448, 2926, 2864, 1455, 1084, 1040, 968 cm$^{-1}$;

$^{1}H$ NMR (500 MHz, $CDCl_3$) δ 6.08 (d, *J* = 15.5 Hz, 1H), 5.69 (ddd, *J* = 15.5, 6.5, 6.5 Hz, 1H), 5.62 (ddd, *J* = 15.5, 7.0, 7.0 Hz, 1H), 5.34 (dd, *J* = 15.5, 7.5 Hz, 1H), 5.29 (d, *J* = 8.0 Hz, 1H), 4.82 (ddd, *J* = 10.0, 8.0, 5.0 Hz, 1H), 4.73–4.70 (m, 2H), 4.69–4.67 (m, 2H), 4.40 (ddd, *J* = 10.5, 7.5, 5.5 Hz, 1H), 4.23–4.15 (m, 2H), 4.10 (dddd, *J* = 10.5, 5.5, 5.5, 5.5 Hz, 1H), 3.94 (ddd, *J* = 8.5, 5.0, 5.0 Hz, 1H), 3.59 (dd, *J* = 11.5, 3.5 Hz, 1H), 3.44 (dd, *J* = 11.5, 6.5 Hz, 1H), 2.37 (ddd, *J* = 13.5, 7.0, 5.5 Hz, 1H), 2.31–2.26 (m, 2H), 2.24 (dd, *J* = 13.0, 5.0 Hz, 1H), 2.18 (dd, *J* = 13.5, 5.0 Hz, 1H), 2.16 (dd, *J* = 13.0, 5.5 Hz, 1H), 2.12 (dd, *J* = 13.0, 5.5 Hz, 1H), 1.99 (ddd, *J* = 6.5, 6.5, 6.5 Hz, 2H), 1.87 (ddd, *J* = 13.5, 8.5, 5.0 Hz, 1H), 1.83 (ddd, *J* = 12.0, 7.0, 7.0 Hz, 1H), 1.78 (s, 3H), 1.75 (br s, 1H), 1.71–1.57 (m, 5H), 1.50 (ddd, *J* = 13.5, 7.0, 5.0 Hz, 1H), 1.34 (quint, *J* = 6.5 Hz, 2H), 1.29–1.23 (m, 4H), 0.91 (d, *J* = 7.0 Hz, 3H), 0.85 (t, *J* = 6.5 Hz, 3H); $^{13}C$ NMR (125 MHz, $CDCl_3$) δ 136.4, 136.1, 134.2, 130.1, 129.5, 125.4, 84.0, 83.8, 83.6 (2C), 80.9, 79.7, 78.7, 78.1, 77.3, 76.4, 65.6, 41.9, 41.8, 41.1, 40.7, 38.9, 36.3, 36.2, 35.3, 32.1, 31.4, 28.7, 22.5, 14.2, 14.0, 13.1; HRMS (ESI) *m*/*z* calcd for $C_{32}H_{50}O_6Na^+$ [(M + Na)$^+$] 553.3500, found 553.3501.

**Iodide S15.** To a solution of alcohol **S14** (3.0 mg, 0.0057 mmol) in THF (0.500 mL) were added imidazole (9.3 mg, 0.14 mmol), $Ph_3P$ (25.4 mg, 0.097 mmol), and $I_2$ (28.9 mg, 0.11 mmol), and the resultant mixture was stirred at room temperature for 40 min. The reaction was quenched with saturated aqueous $Na_2SO_3$ solution. The resultant mixture was extracted with EtOAc, and the organic layer was washed with $H_2O$ and brine, dried ($Na_2SO_4$), filtered, and concentrated under reduced pressure. Purification of the residue by flash column chromatography (silica gel, 0 to 25% EtOAc/hexanes) gave iodide **S15** (2.8 mg, 77%) as a colorless oil: $[\alpha]_D^{23}$ +5.0 (*c* 0.13, $CHCl_3$); IR (film) 2925, 2861, 1732, 1458, 1084, 1032, 966 cm$^{-1}$; $^{1}H$ NMR (500 MHz, $CDCl_3$) δ 6.08 (d, *J* = 15.5 Hz, 1H), 5.69 (ddd, *J* = 15.5, 6.5, 6.5 Hz, 1H), 5.62 (ddd, *J* = 15.5, 7.0, 7.0 Hz, 1H), 5.34 (dd, *J* = 15.5, 7.5 Hz, 1H), 5.30 (d, *J* = 8.0 Hz, 1H), 4.82 (ddd, *J* = 10.5, 8.0, 5.0 Hz, 1H), 4.75–4.70 (m, 2H), 4.69–4.67 (m, 2H), 4.40 (ddd, *J* = 10.5, 7.5, 5.5 Hz, 1H), 4.23–4.05 (m, 4H), 3.25 (dd, *J* = 9.5, 4.5 Hz, 1H), 3.15 (dd, *J* = 9.5, 8.0 Hz, 1H), 2.40–2.26 (m, 3H), 2.24 (dd, *J* = 13.5, 5.0 Hz, 1H), 2.19–2.14 (m, 2H),

2.12 (dd, $J$ = 13.5, 5.0 Hz, 1H), 1.99 (ddd, $J$ = 6.5, 6.5, 6.5 Hz, 2H), 1.88–1.82 (m, 2H), 1.78 (s, 3H), 1.71–1.53 (m, 5H), 1.50 (ddd, $J$ = 13.5, 6.5, 4.5 Hz, 1H), 1.35 (quint, $J$ = 6.5 Hz, 2H), 1.29–1.23 (m, 4H), 0.91 (d, $J$ = 7.0 Hz, 3H), 0.85 (t, $J$ = 6.5 Hz, 3H); $^{13}$C NMR (125 MHz, $CDCl_3$) δ 136.4, 136.2, 134.2, 130.2, 129.5, 125.3, 84.0, 83.8, 83.7, 83.6, 80.9, 79.7, 79.2, 78.0, 76.4, 76.3, 41.9, 41.8, 41.0, 40.7, 40.3, 38.9, 36.8, 36.2, 32.2, 31.4, 28.7, 22.5, 14.2, 14.0, 13.1, 12.0; HRMS (ESI) *m/z* calcd for $C_{32}H_{49}O_5INa^+$ [$(M + Na)^+$] 663.2517, found 663.2529.

**Putative structure 2 of amphirionin-2.** To a solution of iodide **S15** (1.5 mg, 0.0023 mmol) in AcOH (0.50 mL) was added freshly activated zinc dust (32.4 mg, 0.496 mmol), and the resultant mixture was stirred at room temperature for 1.5 h. The reaction mixture was filtered through a pad of Celite, and the filtrate was neutralized with saturated aqueous $NaHCO_3$ solution at 0 °C. The resultant mixture was extracted with EtOAc. The organic layer was washed with $H_2O$ and brine, dried ($Na_2SO_4$), filtered, and concentrated under reduced pressure. Purification of the residue by flash column chromatography (silica gel, 20 to 25% EtOAc/hexanes) gave putative structure **2** of amphirionin-2 (1.2 mg, quant.) as a colorless oil: $[\alpha]_D^{18}$ −3.6 (*c* 0.32, $CHCl_3$); IR (film) 3521, 2926, 2864, 1725, 1433, 1081, 1036, 969, 915 $cm^{-1}$; $^1$H NMR (500 MHz, $C_6D_6$, undeuterated solvent as internal standard: 7.20 ppm) δ 6.22 (d, $J$ = 15.5 Hz, 1H), 5.92 (dddd, $J$ = 17.0, 10.5, 7.0, 7.0 Hz, 1H), 5.76 (ddd, $J$ = 15.5, 7.0, 7.0 Hz, 1H), 5.71 (ddd, $J$ = 15.5, 6.5, 6.5 Hz, 1H), 5.53 (d, $J$ = 8.0 Hz, 1H), 5.51 (dd, $J$ = 15.5, 7.0 Hz, 1H), 5.18 (d, $J$ = 17.0 Hz, 1H), 5.11 (d, $J$ = 10.5 Hz, 1H), 4.91 (ddd, $J$ = 10.0, 8.0, 5.5 Hz, 1H), 4.59 (m, 1H), 4.55 (dd, $J$ = 4.5, 4.5 Hz, 1H), 4.54 (dd, $J$ = 4.5, 4.5 Hz, 1H), 4.43 (dd, $J$ = 4.5, 4.5 Hz, 1H), 4.34 (dd, $J$ = 4.5, 4.5 Hz, 1H), 4.17 (dddd, $J$ = 10.5, 5.5, 5.5, 5.5 Hz, 1H), 4.13 (m, 1H), 3.83 (br d, $J$ = 10.5 Hz, 1H), 3.54 (s, 1H), 2.55 (ddd, $J$ = 13.5, 7.0, 7.0 Hz, 1H), 2.38 (ddd, $J$ = 14.0, 7.0, 5.5 Hz, 1H), 2.24 (ddd, $J$ = 14.0, 7.0, 5.5 Hz, 1H), 2.20 (dd, $J$ = 13.0, 5.5 Hz, 1H), 2.12 (dd, $J$ = 13.5, 5.5 Hz, 1H), 2.10–2.00 (m, 3H), 1.99 (q, $J$ = 6.5 Hz, 2H), 1.74 (s, 3H), 1.61 (m, 1H), 1.55 (ddd, $J$ = 13.0, 10.5, 4.5 Hz, 1H), 1.47–1.37 (m, 3H), 1.36–1.29 (m, 3H), 1.28–1.21 (m, 5H), 1.07 (d, $J$ = 7.0 Hz, 3H), 0.90 (t, $J$ = 6.5 Hz, 3H); $^{13}$C NMR (125 MHz, $C_6D_6$) δ 138.3, 136.7, 135.8, 132.5, 131.4, 131.1, 126.0, 115.9, 84.6,

84.1, 83.9, 83.0, 81.5, 80.9, 79.9, 76.6, 74.4, 42.5, 42.4, 42.2, 41.3, 40.1, 39.5, 39.3, 38.1, 32.5, 31.6, 29.2, 22.8, 14.2, 14.0, 13.1; HRMS (ESI) *m*/*z* calcd for $C_{32}H_{50}O_5Na^+$ [$(M + Na)^+$] 537.3551, found 537.3558.

## 9. Synthesis of vinylstannane 26

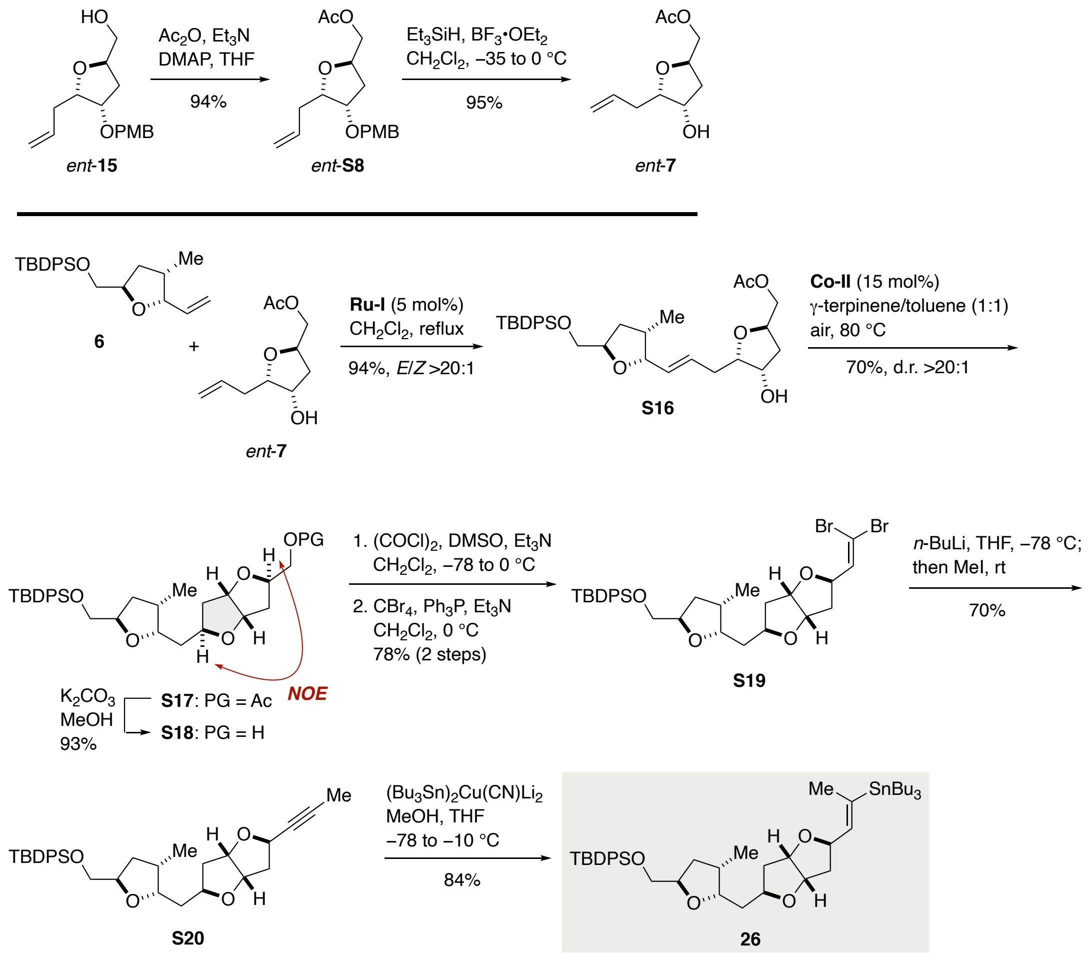


**Scheme S7** Synthesis of vinylstannane **26**.

**Acetate *ent*-S8.** According to the procedure described for acetate **S8**, alcohol *ent*-**15** (103.1 mg, 0.3704 mmol) was converted to acetate *ent*-**S8** (111.3 mg, 94%) as a colorless oil: $[\alpha]_D^{23}$ +35.8 (*c* 0.61, $CHCl_3$); The $^1H$ and $^{13}C$ NMR data were in accordance with those reported for **S8**.

**Alcohol *ent*-7.** According to the procedure described for alcohol **7**, acetate *ent*-**S8** (95.8 mg, 0.299 mmol) was converted to alcohol *ent*-**7** (56.7 mg, 95%) as a colorless oil: $[\alpha]_D^{24}$ −6.2 (*c* 1.00, $CHCl_3$); The $^1H$ and $^{13}C$ NMR data were in accordance with those reported for **7**.

**Olefin S16.** To a solution of olefin **6** (103.9 mg, 0.2729 mmol) and alcohol *ent*-**7** (110.2 mg, 0.5504

mmol) in degassed $CH_2Cl_2$ (2.70 mL) was added a solution of **Ru-I** complex (11.6 mg, 0.0136 mmol) in degassed $CH_2Cl_2$ (2.70 mL), and the resultant solution was refluxed for 21 h. After being cooled to room temperature, DMSO (0.100 mL, 1.44 mmol) was added to the reaction mixture. The resultant mixture was stirred at room temperature under air for 20 h, and then concentrated under reduced pressure. Purification of the residue by flash column chromatography (silica gel, 30 to 50% EtOAc/hexanes) gave olefin **S16** (142.2 mg, 94%, *E*/*Z* >20:1) as a brownish oil: $[\alpha]_D^{23}$ −3.4 (*c* 0.69, $CHCl_3$); IR (film) 3445, 2930, 2857, 1741, 1430, 1238, 1112, 1038, 704 $cm^{-1}$; $^1H$ NMR (400 MHz, $CDCl_3$) δ 7.68–7.63 (m, 4H), 7.42–7.32 (m, 6H), 5.67 (ddd, *J* = 15.2, 7.2, 6.4 Hz, 1H), 5.57 (dd, *J* = 15.6, 6.8 Hz, 1H), 4.45 (dddd, *J* = 8.8, 6.4, 6.4, 3.2 Hz, 1H), 4.34 (dd, *J* = 6.8, 6.8 Hz, 1H), 4.28 (br t, *J* = 3.6 Hz, 1H), 4.20 (dddd, *J* = 7.6, 5.2, 5.2, 4.4 Hz, 1H), 4.13 (dd, *J* = 11.6, 3.2 Hz, 1H), 3.98 (dd, *J* = 11.6, 6.4 Hz, 1H), 3.87 (ddd, *J* = 8.4, 6.4, 2.8 Hz, 1H), 3.62 (dd, *J* = 10.4, 4.4 Hz, 1H), 3.59 (dd, *J* = 10.4, 5.2 Hz, 1H), 2.51–2.38 (m, 2H), 2.33 (m, 1H), 2.08–2.00 (m, 2H), 2.06 (s, 3H), 1.87 (m, 1H), 1.83 (ddd, *J* = 13.6, 8.8, 4.8 Hz, 1H), 1.67 (ddd, *J* = 12.8, 7.6, 5.6 Hz, 1H), 1.03 (s, 9H), 0.89 (d, *J* = 6.8 Hz, 3H); $^{13}C$ NMR (100 MHz, $CDCl_3$) δ 171.0, 135.6 (4C), 133.6, 130.8 (2C), 129.6 (2C), 128.1, 127.6 (4C), 82.7, 82.0, 77.8, 74.8, 72.3, 66.6, 66.5, 37.5, 36.9, 35.8, 32.2, 26.8 (3C), 20.9, 19.2, 14.8; HRMS (ESI) calcd for $C_{32}H_{44}O_6SiNa^+$ $[(M + Na)^+]$ 575.2799, found 575.2803.

**Tetrahydrofuran S17.** To a solution of olefin **S16** (43.1 mg, 0.0780 mmol) in γ-terpinene/toluene (1:1, v/v, 0.800 mL) was added **Co-II** complex (6.0 mg, 0.011 mmol), and the resultant solution was stirred at 80 °C under air for 2.5 h. After being cooled to room temperature, the reaction mixture was concentrated under reduced pressure. Purification of the residue by flash column chromatography (silica gel, 5 to 10% EtOAc/hexanes) gave tetrahydrofuran **S17** (30.0 mg, 70%, d.r. >20:1) as a yellow oil: $[\alpha]_D^{23}$ −18.8 (*c* 0.93, $CHCl_3$), IR (film) 2931, 2857, 1742, 1428, 1236, 1111, 1037, 704 $cm^{-1}$; $^1H$ NMR (400 MHz, $C_6D_6$) δ 7.87–7.81 (m, 4H), 7.31–7.22 (m, 6H), 4.44 (dd, *J* = 4.8, 4.8 Hz, 1H), 4.40 (dd, *J* = 4.4, 4.4 Hz, 1H), 4.33 (m, 1H), 4.25–4.11 (m, 3H), 3.98 (dd, *J* = 11.6, 3.6 Hz, 1H), 3.94 (dd, *J* = 11.6, 6.0 Hz, 1H), 3.70 (dd, *J* = 10.4, 4.4 Hz, 1H), 3.62 (dd, *J* = 10.4, 4.8 Hz, 1H), 2.22 (dd, *J* =

13.2, 4.8 Hz, 1H), 2.00 (m, 1H), 1.91 (dd, $J$ = 13.2, 6.0 Hz, 1H), 1.86 (ddd, $J$ = 12.0, 7.2, 7.2 Hz, 1H), 1.65 (s, 3H), 1.57 (m, 2H), 1.40–1.30 (m, 3H), 1.20 (s, 9H), 0.76 (d, $J$ = 7.2 Hz, 3H); $^{13}$C NMR (100 MHz, $C_6D_6$) δ 170.0, 136.1 (4C), 134.16, 134.14, 129.92, 129.91, 128.1 (4C), 84.5, 83.2, 79.1, 78.0, 77.8, 77.4, 67.2, 66.3, 42.3, 37.7, 37.6, 36.6, 36.1, 27.1 (3C), 20.4, 19.5, 14.4; HRMS (ESI) calcd for $C_{32}H_{44}O_6SiNa^+$ [(M + Na)$^+$] 575.2799, found 575.2807.

**Alcohol S18.** To a solution of tetrahydrofuran **S17** (41.8 mg, 0.0756 mmol) in THF/MeOH (1:1, v/v, 0.760 mL) was added $K_2CO_3$ (3.1 mg, 0.022 mmol), and the resultant mixture was stirred at room temperature for 2.5 h. The reaction was quenched with saturated aqueous $NH_4Cl$ solution. The resultant mixture was extracted with EtOAc, and the organic layer was washed with $H_2O$ and brine, dried ($Na_2SO_4$), filtered, and concentrated under reduced pressure. Purification of the residue by flash column chromatography (silica gel, 30 to 40% EtOAc/hexanes) gave alcohol **S18** (35.9 mg, 93%) as a yellow oil: $[\alpha]_D^{23}$ −20.7 (*c* 0.70, $CHCl_3$); IR (film) 3445, 2931, 2857, 1428, 1112, 1037, 703 cm$^{-1}$; $^1$H NMR (400 MHz, $CDCl_3$) δ 7.70–7.64 (m, 4H), 7.42–7.33 (m, 6H), 4.70–4.67 (m, 2H), 4.22–4.12 (m, 3H), 4.04 (ddd, $J$ = 8.0, 4.8, 4.8 Hz, 1H), 3.71 (dd, $J$ =11.6, 2.8 Hz, 1H), 3.63 (dd, $J$ = 10.8, 4.8 Hz, 1H), 3.60 (dd, $J$ = 10.8, 4.8 Hz, 1H), 3.41 (dd, $J$ = 11.6, 4.8 Hz, 1H), 2.24 (m, 1H), 2.24 (dd, $J$ = 13.6, 4.8 Hz, 1H), 2.03 (ddd, $J$ = 12.4, 7.2, 7.2 Hz, 1H), 1.99 (dd, $J$ = 13.6, 5.6 Hz, 1H), 1.82 (ddd, $J$ = 13.6, 8.0, 5.6 Hz, 1H), 1.74 (br s, 1H), 1.67–1.56 (m, 4H), 1.03 (s, 9H), 0.90 (d, $J$ = 7.2 Hz, 3H); $^{13}$C NMR (100 MHz, $CDCl_3$) δ 135.62 (2C), 135.60 (2C), 133.68, 133.66, 129.54, 129.52, 127.6 (4C), 84.3, 83.4, 80.2, 78.9, 77.8, 77.4, 66.6, 64.0, 41.9, 36.9, 36.2, 36.1, 35.9, 26.8 (3C), 19.2, 14.3; HRMS (ESI) calcd for $C_{30}H_{42}O_5SiNa^+$ [(M + Na)$^+$] 533.2694, found 533.2696.

**Dibromoolefin S19.** To a solution of $(COCl)_2$ (0.030 mL, 0.35 mmol) in $CH_2Cl_2$ (0.60 mL) at −78 °C was added DMSO (0.040 mL, 0.58 mmol), and the resultant solution was stirred at −78 °C for 10 min. To the reaction mixture was added a solution of alcohol **S18** (48.9 mg, 0.0957 mmol) in $CH_2Cl_2$ (0.30 mL + 0.10 mL rinse), and the resultant mixture was stirred at −78 °C for 30 min. To the reaction

mixture was added $Et_3N$ (0.130 mL, 0.938 mmol), and the resultant mixture was allowed to warm to 0 °C over a period of 1 h. The reaction was quenched with saturated aqueous $NaHCO_3$ solution. The resultant mixture was diluted with *t*-BuOMe and washed with $H_2O$ and brine, dried ($MgSO_4$), filtered, and concentrated under reduced pressure to give crude aldehyde, which was used in the next reaction without further purification.

To a solution of $CBr_4$ (127.1 mg, 0.3833 mmol) in $CH_2Cl_2$ (2.00 mL) at 0 °C was added $Ph_3P$ (220.6 mg, 0.8411 mmol), and the resultant mixture was stirred at 0 °C for 30 min. To the reaction mixture were added $Et_3N$ (0.160 mL, 1.15 mmol) and a solution of the above aldehyde in $CH_2Cl_2$ (1.00 mL + 0.50 mL rinse), and the resultant mixture was stirred at 0 °C for 2.5 h. The reaction was quenched with saturated aqueous $NaHCO_3$ solution. The resultant mixture was extracted with EtOAc, and the organic layer was washed with $H_2O$ and brine, dried ($Na_2SO_4$), filtered, and concentrated under reduced pressure. Purification of the residue by flash column chromatography (silica gel, 10% EtOAc/hexanes) gave dibromoolefin **S19** (49.6 mg, 78% for the two steps) as a yellow oil: $[\alpha]_D^{23}$ –23.6 (*c* 0.98, $CHCl_3$); IR (film) 2930, 2856, 1428, 1112, 702 $cm^{-1}$; $^1H$ NMR (400 MHz, $CDCl_3$) δ 7.70–7.65 (m, 4H), 7.42–7.34 (m, 6H), 6.40 (d, *J* = 7.6 Hz, 1H), 4.72–4.67 (m, 2H), 4,65 (ddd, *J* = 10.0, 7.6, 6.0 Hz, 1H), 4.24–4.13 (m, 2H), 4.05 (ddd, *J* = 9.2, 4.8, 4.8 Hz, 1H), 3.64 (dd, *J* = 10.4, 4.8 Hz, 1H), 3.60 (dd, *J* = 10.4, 4.8 Hz, 1H), 2.32 (dd, *J* = 13.6, 6.0 Hz, 1H), 2.25 (m, 1H), 2.26 (dd, *J* = 13.6, 4.8 Hz, 1H), 2.03 (ddd, *J* = 12.4, 7.2, 7.2 Hz, 1H), 1.71 (ddd, *J* = 13.6, 10.0, 4.8 Hz, 1H), 1.68–1.55 (m, 4H), 1.04 (s, 9H), 0.90 (d, *J* = 6.8 Hz, 3H); $^{13}C$ NMR (100 MHz, $CDCl_3$) δ 138.6, 135.6 (4C), 133.68, 133.61, 129.5 (2C), 127.6 (4C), 91.3, 84.4, 82.9, 79.6, 78.8, 78.1, 77.2, 66.6, 41.6, 40.0, 37.1, 36.3, 35.8, 26.8 (3C), 19.2, 14.3; HRMS (ESI) calcd for $C_{31}H_{40}{}^{79}Br_2O_4SiNa^+$ $[(M + Na)^+]$ 685.0955, found 685.0961.

**Alkyne S20.** To a solution of dibromoolefin **S19** (42.0 mg, 0.0632 mmol) in THF (1.20 mL) at −78 °C was added *n*-BuLi (2.67 M solution in *n*-hexane, 0.060 mL, 0.16 mmol), and the resultant solution was stirred at −78 °C for 30 min. To the reaction mixture was added MeI (0.025 mL, 0.40 mmol), and the resultant mixture was allowed to warm to room temperature. The reaction mixture was stirred at room

temperature for 6 h. The reaction was quenched with saturated aqueous $NH_4Cl$ solution at 0 °C. The resultant mixture was extracted with EtOAc, and the organic layer was washed with $H_2O$ and brine, dried ($Na_2SO_4$), filtered, and concentrated under reduced pressure. Purification of the residue by flash column chromatography (silica gel, 3% *t*BuOMe/toluene) gave alkyne **S20** (22.9 mg, 70%) as a colorless oil: $[\alpha]_D^{23}$ −5.1 (*c* 0.66, $CHCl_3$); IR (film) 2930, 2856, 1428, 1112, 703 $cm^{-1}$; $^1H$ NMR (400 MHz, $CDCl_3$) δ 7.69–7.65 (m, 4H), 7.42–7.33 (m, 6H), 4.74–4.69 (m, 2H), 4.65 (dddd, *J* = 6.0, 6.0, 2.0, 2.0 Hz, 1H), 4.17–4.09 (m, 2H), 4.03 (ddd, *J* = 7.2, 5.2, 5.2 Hz, 1H), 3.63 (dd, *J* = 10.4, 4.8 Hz, 1H), 3.59 (dd, *J* = 10.4, 4.8 Hz, 1H), 2.25–2.17 (m, 3H), 2.04 (dd, *J* = 13.2, 7.6 Hz, 1H), 2.03 (ddd, *J* = 13.2, 9.2, 5.6 Hz, 1H), 1.81 (d, *J* = 2.0 Hz, 3H), 1.67–1.54 (m, 4H), 1.03 (s, 9H), 0.89 (d, *J* = 7.2 Hz, 3H); $^{13}C$ NMR (100 MHz, $CDCl_3$) δ 135.6 (4C), 133.68, 133.63, 129.5 (2C), 127.6 (4C), 83.6, 82.9, 81.3, 78.8, 78.0, 77.2, 77.1, 69.0, 66.5, 42.2, 41.0, 36.7, 36.2, 35.8, 26.8 (3C), 19.2, 14.3, 3.6; HRMS (ESI) calcd for $C_{32}H_{42}O_4SiNa^+$ $[(M + Na)^+]$ 541.2745, found 541.2758.

**Vinylstannane 26.** To a suspension of CuCN (26.7 mg, 0.292 mmol) in THF (1.00 mL) at −78 °C was added *n*-BuLi (2.80 M solution in *n*-hexane, 0.205 mL, 0.574 mmol), and the resultant mixture was stirred at −40 °C for 20 min. To the reaction mixture at −78 °C was added *n*-$Bu_3SnH$ (0.155 mL, 0.575 mmol), and the resultant mixture was stirred at −40 °C for 15 min. To the reaction mixture at −78 °C was added MeOH (0.230 mL, 5.67 mmol), and the resultant mixture was stirred at −10 °C for 30 min. To the reaction mixture at −78 °C was added a solution of alkyne **S20** (29.3 mg, 0.0565 mmol) in THF (0.70 mL + 0.30 mL rinse), and the resultant mixture was stirred at −78 °C for 5 min and then at −10 °C for 5 h. The reaction was quenched with a mixture of saturated aqueous $NH_4Cl$ solution/30% $NH_4OH$ solution (4:1, v/v, 5.0 mL) at −10 °C. The resultant mixture was extracted with $Et_2O$, and the organic layer was washed with brine, dried ($MgSO_4$), filtered, and concentrated under reduced pressure. Purification of the residue by flash column chromatography (silica gel, 0–3% EtOAc/hexanes) gave vinylstannane **26** (38.4 mg, 84%) as a yellow oil: $[\alpha]_D^{19}$ −6.0 (*c* 0.99, $CHCl_3$); IR (film) 2955, 2927, 2855, 1461, 1428, 1105, 702 $cm^{-1}$; $^1H$ NMR (400 MHz, $C_6D_6$) δ 7.88–7.84 (m, 4H), 7.32–7.23 (m,

6H), 5.91 (dq, *J* = 7.2, 1.6 Hz, 1H), 5.18 (ddd, *J* = 10.8, 7.2, 5.2 Hz, 1H), 4.61 (dd, *J* = 4.8, 4.8 Hz, 1H), 4.53 (dd, *J* = 4.8, 4.8 Hz, 1H), 4.50 (m, 1H), 4.14 (ddd, *J* = 7.6, 5.2, 5.2 Hz, 1H), 4.15 (dddd, *J* = 7.2, 7.2, 4.4, 4.4 Hz, 1H), 3.69 (dd, *J* = 10.4, 4.4 Hz, 1H), 3.63 (dd, *J* = 10.4, 4.4 Hz, 1H), 2.33 (dd, *J* = 13.2, 4.8 Hz, 1H), 2.33 (dd, *J* = 12.8, 5.2 Hz, 1H), 2.01 (m, 1H), 1.91 (d, *J* = 2.0 Hz, 3H), 1.83 (ddd, *J* = 12.4, 7.2, 7.2 Hz, 1H), 1.63–1.53 (m, 7H), 1.47 (ddd, *J* = 13.2, 10.0, 4.8 Hz, 1H), 1.41–1.31 (m, 8H), 1.21 (s, 9H), 1.10–0.83 (m, 16H), 0.77 (d, *J* = 6.8 Hz, 3H); $^{13}$C NMR (100 MHz, $C_6D_6$) δ 142.5, 140.8, 136.12 (2C), 136.11 (2C), 134.2, 129.91, 129.88, 129.8, 128.1 (4C), 84.1, 83.9, 79.2, 78.7, 77.5, 75.8, 67.2, 42.8, 42.4, 38.0, 36.6, 36.1, 29.6 (3C), 27.8 (3C), 27.1 (3C), 20.0, 19.5, 14.5, 13.9 (3C), 9.4 (3C); HRMS (ESI) calcd for $C_{44}H_{70}O_4SiSnNa^+$ [(M + Na)$^+$] 833.3958, found 833.3958.

## 10. Total synthesis of correct structure 27 of amphirionin-2 and its diastereomer 28

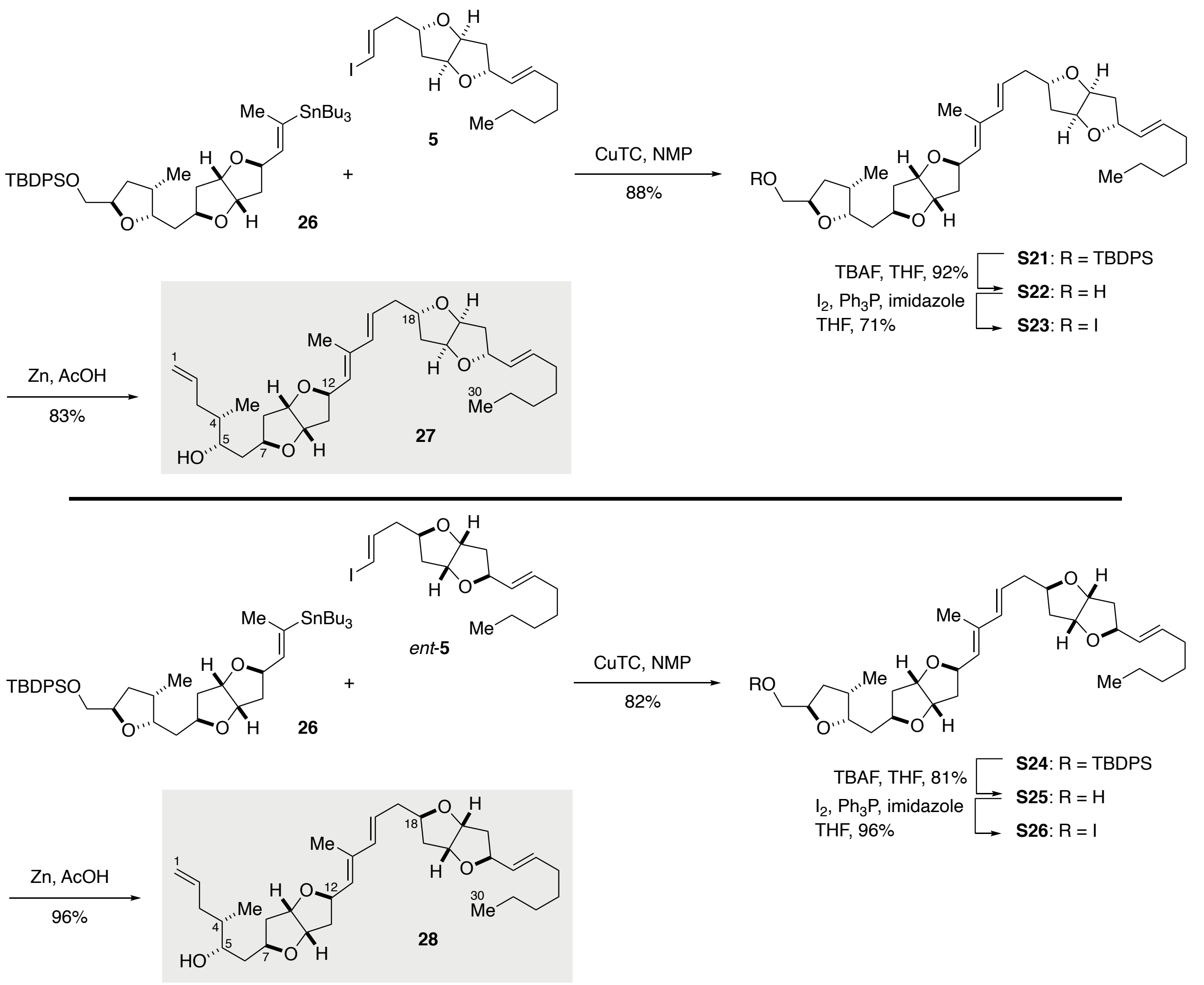


**Scheme S8** Synthesis of correct structure **27** of amphirionin-2 and its diastereomer **28**.

**Diene S21.** To a solution of vinylstannane **26** (33.4 mg, 0.0412 mmol) and iodoolefin **5** (17.6 mg, 0.0468 mmol) in degassed NMP (0.800 mL) at 0 °C was added CuTC (31.9 mg, 0.167 mmol), and the resultant mixture was stirred at room temperature for 24 h. The reaction was quenched with 5% $NH_4OH$ solution. The resultant mixture was stirred at room temperature for 30 min and then extracted with EtOAc. The organic layer was washed with brine, dried ($Na_2SO_4$), filtered, and concentrated under reduced pressure. Purification of the residue by flash column chromatography (silica gel, 25 to 30% *t*-BuOMe/hexanes) gave diene **S21** (27.8 mg, 88%) as a yellow oil: $[\alpha]_D^{18}$ −9.3 (*c* 1.00, $CHCl_3$);

IR (film) 2928, 2858, 1429, 1107, 1036, 705 cm$^{-1}$; $^1$H NMR (500 MHz, $CDCl_3$) δ 7.69–7.66 (m, 4H), 7.40–7.34 (m, 6H), 6.07 (d, *J* = 15.5 Hz, 1H), 5.70 (ddd, *J* = 15.0, 6.5, 6.5 Hz, 1H), 5.60 (ddd, *J* = 15.5, 7.0, 7.0 Hz, 1H), 5.34 (dd, *J* = 15.0, 7.5 Hz, 1H), 5.28 (d, *J* = 8.0 Hz, 1H), 4.80 (ddd, *J* = 10.5, 8.0, 5.0 Hz, 1H), 4.72 (dd, *J* = 4.5, 4.5 Hz, 1H), 4.69–4.67 (m, 3H), 4.40 (ddd, *J* = 10.5, 7.5, 5.0 Hz, 1H), 4.23 (m, 1H), 4.16 (dddd, *J* = 7.5, 7.5, 4.5, 4.5 Hz, 1H), 4.10 (dddd, *J* = 10.5, 5.5, 5.5, 5.5 Hz, 1H), 4.06 (ddd, *J* = 9.0, 4.5, 4.5 Hz, 1H), 3.62 (d, *J* = 4.5 Hz, 2H), 2.37 (ddd, *J* = 14.0, 7.0, 5.5 Hz, 1H), 2.28–2.23 (m, 3H), 2.16 (dd, *J* = 13.0, 5.0 Hz, 1H), 2.16 (dd, *J* = 13.0, 5.0 Hz, 1H), 2.12 (dd, *J* = 13.0, 5.5 Hz, 1H), 2.02 (m, 1H), 1.99 (ddd, *J* = 6.5, .6.5, 6.5 Hz, 2H), 1.71 (s, 3H), 1.68–1.56 (m, 7H), 1.35 (quint, *J* = 6.5 Hz, 2H), 1.29–1.23 (m, 4H), 1.03 (s, 9H), 0.91 (d, *J* = 7.5 Hz, 3H), 0.85 (t, *J* = 6.5 Hz, 3H); $^{13}$C NMR (125 MHz, $CDCl_3$) δ 136.5, 136.2, 135.6 (4C), 134.2, 133.69, 133.66, 130.2, 129.5 (3C), 127.6 (4C), 125.2, 84.0, 83.8, 83.6 (2C), 80.9, 79.7, 78.9, 78.3, 77.2, 76.1, 66.6, 42.0, 41.9, 41.8, 40.7, 38.8, 37.2, 36.3, 35.9, 32.1, 31.3, 28.7, 26.8 (3C), 22.5, 19.2, 14.3, 14.0, 13.0; HRMS (ESI) *m*/*z* calcd for $C_{48}H_{68}O_6SiNa^+$ [(M + Na)$^+$] 791.4677, found 791.4674.

**Alcohol S22.** To a solution of diene **S21** (9.0 mg, 0.012 mmol) in THF (1.00 mL) at 0 °C was added TBAF (1.0 M solution in THF, 0.120 mL, 0.120 mmol), and the resultant solution was stirred at room temperature for 2 h. The reaction was quenched with saturated aqueous $NH_4Cl$ solution at 0 °C. The resultant mixture was extracted with EtOAc. The organic layer was washed with $H_2O$ and brine, dried ($Na_2SO_4$), filtered, and concentrated under reduced pressure. Purification of the residue by flash column chromatography (silica gel, 60 to 80% EtOAc/hexanes) gave alcohol **S22** (5.6 mg, 92%) as a yellow oil: $[\alpha]_D^{20}$ −15.4 (*c* 0.56, $CHCl_3$); IR (film) 3445, 2926, 2869, 1434, 1378, 1320, 1085, 1038, 968 cm$^{-1}$; $^1$H NMR (500 MHz, $CDCl_3$) δ 6.08 (d, *J* = 15.5 Hz, 1H), 5.69 (ddd, *J* = 15.0, 7.0, 7.0 Hz, 1H), 5.62 (ddd, *J* = 15.5, 7.5, 7.5 Hz, 1H), 5.34 (dd, *J* = 15.0, 7.0 Hz, 1H), 5.29 (d, *J* = 8.0 Hz, 1H), 4.82 (ddd, *J* = 10.5, 8.0, 5.0 Hz, 1H), 4.72 (dd, *J* = 4.5, 4.5 Hz, 1H), 4.69–4.67 (m, 3H), 4.40 (ddd, *J* = 10.5, 7.0, 5.0 Hz, 1H), 4.23 (m, 1H), 4.14 (dddd, *J* = 7.0, 7.0, 7.0, 3.0 Hz, 1H), 4.10 (dddd, *J* = 10.5, 5.0, 5.0, 5.0 Hz, 1H), 4.04 (ddd, *J* = 9.0, 5.0, 5.0 Hz, 1H), 3.59 (dd, *J* = 11.5, 3.0 Hz, 1H), 3.45 (dd, *J*

= 11.5, 7.0 Hz, 1H), 2.37 (ddd, *J* = 13.5, 7.5, 5.0 Hz, 1H), 2.30–2.14 (m, 5H), 2.12 (dd, *J* = 13.0, 5.0 Hz, 1H), 1.99 (ddd, *J* = 7.0, 7.0, 7.0 Hz, 2H), 1.83 (ddd, *J* = 12.0, 7.0, 7.0 Hz, 1H), 1.78 (s, 3H), 1.73 (m, 1H), 1.68–1.56 (m, 7H), 1.34 (quint, *J* = 7.0 Hz, 2H), 1.29–1.21 (m, 4H), 0.92 (d, *J* = 7.0 Hz, 3H), 0.85 (t, *J* = 7.0 Hz, 3H); $^{13}$C NMR (125 MHz, $CDCl_3$) δ 136.4, 136.3, 134.2, 130.1, 129.5, 125.4, 84.0, 83.7, 83.6 (2C), 80.9, 79.7, 78.8, 78.0, 77.2, 76.1, 65.6, 42.0, 41.9, 41.8, 40.7, 38.9, 36.8, 36.5, 35.3, 32.1, 31.4, 28.7, 22.5, 14.3, 14.0, 13.1; HRMS (ESI) *m/z* calcd for $C_{32}H_{50}O_6Na^+$ [$(M + Na)^+$] 553.3500, found 553.3502.

**Iodide S23.** To a solution of alcohol **S22** (4.2 mg, 0.0079 mmol) in THF (0.500 mL) were added imidazole (12.3 mg, 0.181 mmol), $Ph_3P$ (37.7 mg, 0.144 mmol), and $I_2$ (30.3 mg, 0.119 mmol), and the resultant mixture was stirred at room temperature for 1 h. The reaction was quenched with saturated aqueous $Na_2SO_3$ solution. The resultant mixture was extracted with EtOAc. The organic layer was washed with $H_2O$ and brine, dried ($Na_2SO_4$), filtered, and concentrated under reduced pressure. Purification of the residue by flash column chromatography (silica gel, 20 to 30% EtOAc/hexanes) gave iodide **S23** (3.6 mg, 71%) as a colorless oil: $[\alpha]_D^{20}$ −2.3 (*c* 0.36, $CHCl_3$); IR (film) 2926, 2861, 1432, 1371, 1314, 1082, 1037, 967 cm$^{-1}$; $^1$H NMR (500 MHz, $C_6D_6$) δ 6.16 (d, *J* = 15.5 Hz, 1H), 5.67 (ddd, *J* = 7.0, 7.0, 7.0 Hz, 1H), 5.66 (ddd, *J* = 15.5, 7.0, 7.0 Hz, 1H), 5.54 (d, *J* = 8.0 Hz, 1H), 5.46 (dd, *J* = 15.0, 7.0 Hz, 1H), 5.02 (ddd, *J* = 10.0, 8.0, 5.5 Hz, 1H), 4.57–4.46 (m, 5H), 4.35 (dddd, *J* = 10.0, 5.5, 5.5, 5.5 Hz, 1H), 4.14–4.08 (m, 2H), 3.89 (dddd, *J* = 7.0, 7.0, 7.0, 5.5 Hz, 1H), 2.90 (dd, *J* = 10.0, 5.5 Hz, 1H), 2.73 (dd, *J* = 10.0, 7.0 Hz, 1H), 2.32 (ddd, *J* = 14.0, 7.0, 5.5 Hz, 1H), 2.23 (dd, *J* = 13.0, 5.0 Hz, 1H), 2.22–2.12 (m, 3H), 2.06 (dd, *J* = 13.0, 5.0 Hz, 1H), 1.94 (ddd, *J* = 7.0, 7.0 7.0 Hz, 2H),1.92 (m, 1H), 1.68 (s, 3H), 1.55–1.32 (m, 8H), 1.29 (quint, *J* = 7.0 Hz, 2H), 1.25–1.15 (m, 4H), 0.85 (t, *J* = 7.0 Hz, 3H), 0.65 (d, *J* = 7.0 Hz, 3H); $^{13}$C NMR (125 MHz, $C_6D_6$) δ 136.9, 135.6, 132.5, 131.7, 131.1, 125.7, 84.1, 84.0, 83.9 (2C), 80.8, 79.9, 79.7, 78.3, 76.7, 76.5, 42.6, 42.55, 42.50, 41.3, 40.3, 39.5, 37.4, 36.9, 32.5, 31.6, 29.2, 22.8, 14.2 (2C), 13.1, 11.9; HRMS (ESI) *m/z* calcd for $C_{32}H_{49}O_5INa^+$ [$(M + Na)^+$] 663.2517, found 663.2509.

**Correct structure 27.** To a solution of iodide **S23** (3.0 mg, 0.0047 mmol) in AcOH (0.50 mL) was added freshly activated zinc powder (63.2 mg, 0.967 mmol), and the resultant mixture was stirred at room temperature for 2 h. The reaction mixture was filtered through a pad of Celite, and the filtrate was neutralized with saturated aqueous $NaHCO_3$ solution at 0 °C. The resultant mixture was extracted with EtOAc. The organic layer was washed with $H_2O$ and brine, dried ($Na_2SO_4$), filtered, and concentrated under reduced pressure. Purification of the residue by flash column chromatography (silica gel, 20 to 30% EtOAc/hexanes) gave correct structure **27** of amphirionin-2 (2.0 mg, 83%) as a colorless oil: $[\alpha]_D^{18}$ +2.5 (*c* 0.18, $CHCl_3$); IR (film) 3471, 2926, 1435, 1378, 1319, 1083, 1037, 969, 915 $cm^{-1}$; $^1H$ NMR (500 MHz, $C_6D_6$, undeuterated solvent as internal standard: 7.20 ppm) δ 6.22 (d, *J* = 15.5 Hz, 1H), 5.82 (dddd, *J* = 16.0, 10.5, 7.0, 7.0 Hz, 1H), 5.75 (ddd, *J* = 15.5, 7.0, 7.0 Hz, 1H), 5.71 (ddd, *J* = 15.5, 7.0, 7.0 Hz, 1H), 5.56 (ddd, *J* = 8.0 Hz, 1H), 5.51 (dd, *J* = 15.5, 7.0 Hz, 1H), 5.09 (d, *J* = 16.0 Hz, 1H), 5.06 (d, *J* = 10.5 Hz, 1H), 5.00 (ddd, *J* = 10.5, 8.0, 5.0 Hz, 1H), 4.61–4.54 (m, 4H), 4.42 (dd, *J* = 4.5, 4.5 Hz, 1H), 4.41 (m, 1H), 4.17 (dddd, *J* = 5.0, 5.0, 5.0, 5.0 Hz, 1H), 3.83 (m, 1H), 2.38 (ddd, *J* = 14.0, 7.0, 5.0 Hz, 1H), 2.30 (ddd, *J* = 14.0, 7.0, 7.0 Hz, 1H), 2.23 (ddd, *J* = 14.0, 7.0, 5.0 Hz, 1H), 2.20 (dd, *J* = 14.0, 5.0 Hz, 1H), 2.17 (dd, *J* = 14.0, 5.0 Hz, 1H), 2.12 (dd, *J* = 13.0, 5.0 Hz, 1H), 2.07 (dd, *J* = 13.0, 5.5 Hz, 1H), 2.07 (br s, 1H), 1.99 (ddd, *J* = 7.0, 7.0, 7.0 Hz, 2H), 1.93 (ddd, *J* = 14.0, 7.0, 7.0 Hz, 1H) 1.74 (s, 3H), 1.69 (ddd, *J* = 13.5, 9.5, 4.0 Hz, 1H), 1.58–1.45 (m, 4H), 1.43–1.37 (m, 2H), 1.34 (quint, *J* = 7.0 Hz, 2H), 1.30–1.20 (m, 4H), 0.99 (d, *J* = 7.0 Hz, 3H), 0.90 (t, *J* = 7.0 Hz, 3H); $^{13}C$ NMR (125 MHz, $C_6D_6$) δ 137.9, 136.8, 135.7, 132.5, 131.6, 131.1, 125.8, 115.9, 84.1, 84.0, 83.9, 83.8, 80.9, 79.9, 78.3, 76.8, 71.2, 42.5, 42.4, 41.3, 41.1, 39.5, 39.1, 39.0, 38.2, 32.5, 31.6, 29.2, 22.8, 14.2, 14.0, 13.1; HRMS (ESI) *m*/*z* calcd for $C_{32}H_{50}O_5Na^+$ $[(M + Na)^+]$ 537.3551, found 537.3550.

**Diene S24.** To a solution of vinylstannane **26** (14.2 mg, 0.0175 mmol) and iodoolefin *ent*-**5** (8.0 mg, 0.021 mmol) in degassed NMP (0.500 mL) at 0 °C was added CuTC (14.4 mg, 0.0755 mmol), and the

resultant mixture was stirred at room temperature for 15 h. The reaction was quenched with 5% $NH_4OH$ solution, and the resultant mixture was stirred at room temperature for 0.5 h. The resultant mixture was extracted with EtOAc. The organic layer was washed with brine, dried ($Na_2SO_4$), filtered, and concentrated under reduced pressure. Purification of the residue by flash column chromatography (silica gel, 25 to 30% *t*-BuOMe/hexanes) gave diene **S24** (11.0 mg, 82%) as a colorless oil: $[\alpha]_D^{17}$ −7.9 (*c* 1.01, $CHCl_3$); IR (film) 2929, 2857, 1428, 1113, 703 $cm^{-1}$; $^1H$ NMR (500 MHz, $CDCl_3$) δ 7.69–7.66 (m, 4H), 7.41–7.34 (m, 6H), 6.07 (d, *J* = 15.5 Hz, 1H), 5.70 (ddd, *J* = 15.0, 7.0, 7.0 Hz, 1H), 5.61 (ddd, *J* = 15.5, 7.0, 7.0 Hz, 1H), 5.34 (dd, *J* = 15.0, 7.0 Hz, 1H), 5.29 (d, *J* = 8.5 Hz, 1H), 4.80 (ddd, *J* = 10.0, 8.5, 5.0 Hz, 1H), 4.72 (dd, *J* = 4.5, 4.5 Hz, 1H), 4.68–4.67 (m, 3H), 4.40 (ddd, *J* = 10.0, 7.0, 5.0 Hz, 1H), 4.23 (m, 1H), 4.16 (dddd, *J* = 7.5, 7.5, 5.0, 5.0 Hz, 1H), 4.11 (dddd, *J* = 10.5, 5.5, 5.5, 5.5 Hz, 1H), 4.06 (ddd, *J* = 9.0, 4.5, 4.5 Hz, 1H), 3.62 (d, *J* = 4.5 Hz, 2H), 2.36 (ddd, *J* = 14.5, 7.0, 5.5 Hz, 1H), 2.29–2.23 (m, 3H), 2.18–2.14 (m, 2H), 2.12 (dd, *J* = 13.5, 5.5 Hz, 1H), 2.02 (m, 1H), 1.99 (ddd, *J* = 7.0, 7.0, 7.0 Hz, 2H), 1.71 (s, 3H), 1.68–1.56 (m, 7H), 1.35 (quint, *J* = 7.0 Hz, 2H), 1.29–1.23 (m, 4H), 1.03 (s, 9H), 0.91 (d, *J* = 7.5 Hz, 3H), 0.86 (t, *J* = 7.0 Hz, 3H); $^{13}C$ NMR (125 MHz, $CDCl_3$) δ 136.5, 136.2, 135.6 (4C), 134.2, 133.72, 133.69, 130.3, 129.5 (3C), 127.6 (4C), 125.1, 84.0, 83.8, 83.63, 83.60, 80.9, 79.7, 78.9, 78.3, 77.2, 76.1, 66.6, 42.0, 41.9, 41.8, 40.6, 38.7, 37.3, 36.3, 35.9, 32.2, 31.4, 28.7, 26.8 (3C), 22.5, 19.2, 14.3, 14.0, 13.1; HRMS (ESI) *m*/*z* calcd for $C_{48}H_{68}O_6SiNa^+$ [(M + Na)$^+$] 791.4677, found 791.4676.

**Alcohol S25.** To a solution of diene **S24** (10.6 mg, 0.0138 mmol) in THF (1.00 mL) at 0 °C was added TBAF (1.0 M solution in THF, 0.140 mL, 0.140 mmol), and the resultant solution was stirred at room temperature for 2 h. The reaction was quenched with saturated aqueous $NH_4Cl$ solution at 0 °C. The resultant mixture was extracted with EtOAc. The organic layer was washed with $H_2O$ and brine, dried ($Na_2SO_4$), filtered, and concentrated under reduced pressure. Purification of the residue by flash column chromatography (silica gel, 60 to 80% EtOAc/hexanes) gave alcohol **S25** (5.9 mg, 81%) as a colorless oil: $[\alpha]_D^{19}$ −18.3 (*c* 0.66, $CHCl_3$); IR (film) 3444, 2927, 2870, 1454, 1378, 1320, 1085, 1038,

968 cm$^{-1}$; $^1$H NMR (500 MHz, $CDCl_3$) δ 6.08 (d, *J* = 15.5 Hz, 1H), 5.69 (ddd, *J* = 15.0, 7.0, 7.0 Hz, 1H), 5.62 (ddd, *J* = 15.5, 7.5, 7.5 Hz, 1H), 5.33 (dd, *J* = 15.0, 7.0 Hz, 1H), 5.29 (d, *J* = 8.0 Hz, 1H), 4.82 (ddd, *J* = 10.0, 8.0, 5.0 Hz, 1H), 4.71 (dd, *J* = 4.5, 4.5 Hz, 1H), 4.69–4.66 (m, 3H), 4.40 (ddd, *J* = 10.0, 7.0, 5.0 Hz, 1H), 4.22 (m, 1H), 4.14 (dddd, *J* = 7.0, 7.0, 7.0, 3.0 Hz, 1H), 4.11 (dddd, *J* = 10.0, 5.0, 5.0, 5.0 Hz, 1H), 4.04 (ddd, *J* = 9.5, 5.0, 5.0 Hz, 1H), 3.59 (dd, *J* = 11.5, 3.0 Hz, 1H), 3.45 (dd, *J* = 11.5, 7.0 Hz, 1H), 2.36 (ddd, *J* = 13.5, 7.5, 5.0 Hz, 1H), 2.30–2.25 (m, 2H), 2.23 (dd, *J* = 13.5, 5.0 Hz, 1H), 2.19 (dd, *J* = 13.5, 5.0 Hz, 1H), 2.15 (dd, *J* = 13.5, 5.0 Hz, 1H), 2.12 (dd, *J* = 13.5, 5.0 Hz, 1H), 1.99 (ddd, *J* = 7.0, 7.0, 7.0 Hz, 2H), 1.85 (br s, 1H), 1.83 (ddd, *J* = 12.5, 7.0, 7.0 Hz, 1H), 1.78 (s, 3H), 1.70–1.54 (m, 7H), 1.34 (quint, *J* = 7.0 Hz, 2H), 1.29–1.20 (m, 4H), 0.91 (d, *J* = 7.5 Hz, 3H), 0.85 (t, *J* = 7.0 Hz, 3H); $^{13}$C NMR (125 MHz, $CDCl_3$) δ 136.4, 136.3, 134.2, 130.1, 129.5, 125.3, 84.0, 83.7, 83.6 (2C), 80.9, 79.6, 78.8, 78.0, 77.2, 76.1, 65.6, 41.93, 41.85, 41.82, 40.6, 38.7, 36.8, 36.4, 35.3, 32.1, 31.4, 28.7, 22.5, 14.3, 14.0, 13.1; HRMS (ESI) *m*/*z* calcd for $C_{32}H_{50}O_6Na^+$ [(M + Na)$^+$] 553.3500, found 553.3498.

**Iodide S26.** To a solution of alcohol **S25** (3.0 mg, 0.0057 mmol) in THF (1.00 mL) were added imidazole (9.5 mg, 0.14 mmol), $Ph_3P$ (26.1 mg, 0.0995 mmol), and $I_2$ (21.2 mg, 0.0835 mmol), and the resultant mixture was stirred at room temperature for 1 h. The reaction was quenched with saturated $Na_2SO_3$ solution at 0 °C. The resultant mixture was extracted with EtOAc. The organic layer was washed with $H_2O$ and brine, dried ($Na_2SO_4$), filtered, and concentrated under reduced pressure. Purification of the residue by flash column chromatography (silica gel, 20 to 25% EtOAc/hexanes) gave iodide **S26** (3.5 mg, 96%) as a colorless oil: $[\alpha]_D^{18}$ −2.2 (*c* 0.35, $CHCl_3$); IR (film) 2926, 2864, 1432, 1377, 1318, 1084, 1037, 968 cm$^{-1}$; $^1$H NMR (500 MHz, $C_6D_6$) δ 6.16 (d, *J* = 15.5 Hz, 1H), 5.69 (ddd, *J* = 15.5, 7.0, 7.0 Hz, 1H), 5.66 (ddd, *J* = 15.5, 7.0, 7.0 Hz, 1H), 5.54 (d, *J* = 8.0 Hz, 1H), 5.46 (dd, *J* = 15.5, 7.0 Hz, 1H), 5.02 (ddd, *J* = 10.5, 8.0, 5.5 Hz, 1H), 4.57–4.46 (m, 5H), 4.36 (dddd, *J* = 10.5, 5.5, 5.5, 5.5 Hz, 1H), 4.15–4.09 (m, 2H), 3.89 (dddd, *J* = 7.0, 7.0, 7.0, 5.0 Hz, 1H), 2.90 (dd, *J* = 10.0, 5.0 Hz, 1H), 2.73 (dd, *J* = 10.0, 7.0 Hz, 1H), 2.33 (ddd, *J* = 13.0, 7.0, 5.5 Hz, 1H), 2.25–2.13 (m,

4H), 2.06 (dd, *J* = 13.0, 5.0 Hz, 1H), 1.94 (ddd, *J* = 7.0, 7.0, 7.0 Hz, 2H), 1.91 (m, 1H), 1.69 (s, 3H), 1.53–1.33 (m, 8H), 1.29 (quint, *J* = 7.0 Hz, 2H), 1.25–1.15 (m, 4H), 0.85 (t, *J* = 7.0 Hz, 3H), 0.65 (d, *J* = 7.0 Hz, 3H); $^{13}$C NMR (125 MHz, $C_6D_6$) δ 136.6, 135.4, 132.2, 131.5, 130.9, 125.4, 83.8, 83.7, 83.7 (2C), 80.6, 79.7, 79.4, 78.0, 76.5, 76.3, 42.33, 42.28, 42.26, 41.0, 40.1, 39.1, 37.1, 36.7, 32.3, 31.4, 28.9, 22.6, 14.0 (2C), 12.8, 11.7; HRMS (ESI) *m*/*z* calcd for $C_{32}H_{49}O_5INa^+$ [(M + Na)$^+$] 663.2517, found 663.2515.

**Diastereomer 28.** To a solution of iodide **S26** (2.9 mg, 0.0045 mmol) in AcOH (0.50 mL) was added freshly activated zinc powder (59.6 mg, 0.912 mmol), and the resultant mixture was stirred at room temperature for 3.5 h. The reaction mixture was filtered through a pad of Celite, and the filtrate was neutralized with saturated aqueous $NaHCO_3$ solution at 0 °C. The resultant mixture was extracted with EtOAc. The organic layer was washed with $H_2O$ and brine, dried ($Na_2SO_4$), filtered, and concentrated under reduced pressure. Purification of the residue by flash column chromatography (silica gel, 20 to 30% EtOAc/hexanes) gave diastereomer **28** (2.2 mg, 96%) as a colorless oil: $[\alpha]_D^{17}$ +3.7 (*c* 0.29, $CHCl_3$); IR (film) 3477, 2926, 2864, 1434, 1378, 1319, 1083, 1037, 969, 915 cm$^{-1}$; $^1$H NMR (500 MHz, $C_6D_6$, undeuterated solvent as internal standard: 7.20 ppm) δ 6.22 (d, *J* = 16.0 Hz, 1H), 5.82 (dddd, *J* = 17.0, 10.5, 7.0, 7.0 Hz, 1H), 5.75 (ddd, *J* = 16.0, 7.0, 7.0 Hz, 1H), 5.71 (ddd, *J* = 15.5, 7.0, 7.0 Hz, 1H), 5.56 (d, *J* = 8.0 Hz, 1H), 5.51 (dd, *J* = 15.5, 7.0 Hz, 1H), 5.09 (d, *J* = 17.0 Hz, 1H), 5.06 (d, *J* = 10.5 Hz, 1H), 5.00 (ddd, *J* = 10.5, 8.0, 5.5 Hz, 1H), 4.61–4.50 (m, 4H), 4.42 (dd, *J* = 4.5, 4.5 Hz, 1H), 4.41 (m, 1H), 4.18 (dddd, *J* = 5.5, 5.5, 5.5, 5.5 Hz, 1H), 3.83 (m, 1H), 2.38 (ddd, *J* = 14.0, 7.0, 5.5 Hz, 1H), 2.30 (ddd, *J* = 13.5, 7.0, 7.0 Hz, 1H), 2.23 (ddd, *J* = 14.0, 7.0, 5.5 Hz, 1H), 2.20 (dd, *J* = 13.0, 5.0 Hz, 1H), 2.16 (dd, *J* = 14.0, 6.0 Hz, 1H), 2.12 (dd, *J* = 13.0, 5.5 Hz, 1H), 2.07 (dd, *J* = 13.0, 5.0 Hz, 1H), 2.07 (br s, 1H), 1.99 (ddd, *J* = 7.0, 7.0, 7.0 Hz, 2H), 1.93 (ddd, *J* = 13.5, 7.0, 7.0 Hz, 1H), 1.74 (s, 3H), 1.69 (ddd, *J* = 14.5, 9.5, 4.0 Hz, 1H), 1.58–1.45 (m, 4H), 1.43–1.37 (m, 2H), 1.34 (quint, *J* = 7.0 Hz, 2H), 1.30–1.20 (m, 4H), 0.99 (d, *J* = 7.0 Hz, 3H), 0.90 (t, *J* = 7.0 Hz, 3H); $^{13}$C NMR (125 MHz, $C_6D_6$) δ 137.9, 136.8, 135.7, 132.5, 131.6, 131.1, 125.8, 115.9, 84.1, 84.0, 83.9, 83.8, 80.9,

79.9, 78.3, 76.8, 71.2, 42.5, 42.4, 41.3, 41.1, 39.5, 39.1, 39.0, 38.2, 32.5, 31.6, 29.2, 22.8, 14.2, 14.0, 13.1; HRMS (ESI) *m*/*z* calcd for $C_{32}H_{50}O_5Na^+$ [$(M + Na)^+$] 537.3551, found 537.3551.

## 11. Conformational analysis of the C1–C10 moiety of 1, 2, 27, and 28

(4*S*,5*S*,7*R*)-isomer (**1**, **2**)

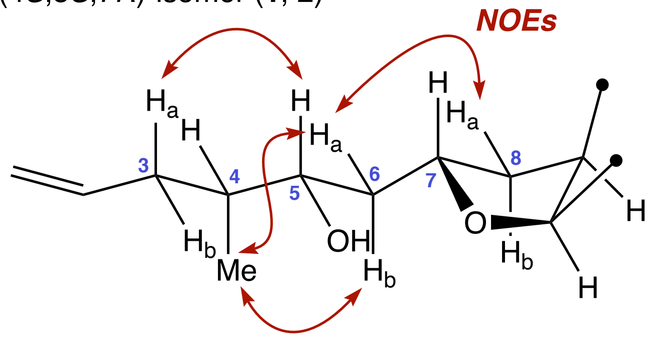


observed

| | | |
|---|---|---|
| $^{3}J_{H4,H5}$ | 3.9 Hz | H4/H5 gauche |
| $^{3}J_{H5,H6a}$ | 9.6 Hz | **H5/H6a anti (?)** |
| $^{3}J_{H5,H6b}$ | 2.2 Hz | **H5/H6b gauche (?)** |
| $^{3}J_{H6a,H7}$ | 3.6 Hz | H6a/H7 gauche |
| $^{3}J_{H6b,H7}$ | 8.1 Hz | H6b/H7 anti |

(4*S*,5*S*,7*S*)-isomer (**27**, **28**)

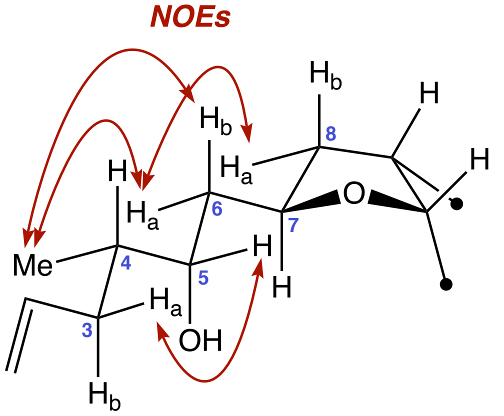


H4/H5 gauche
H5/H6a anti
H5/H6b gauche
H6a/H7 gauche
H6b/H7 anti

**Fig. S1** Conformational analysis of the C1–C10 moiety of **1**, **2**, **27**, and **28**.

We considered that the acyclic C1–C7 moiety of **1**, **2**, **27**, and **28** would adopt a zig-zag arrangement. The conformation of (4*S*,5*S*,7*R*)-isomers **1** and **2** is inconsistent with the $^{3}J_{H,H}$ data (H5/H6a and H5/H6b) of natural amphirionin-2, whereas that of (4*S*,5*S*,7*S*)-isomers **27** and **28** is in accordance with all the $^{3}J_{H,H}$ values and NOESY correlations observed for natural amphirionin-2.

## 12. Comparison of $^{1}$H NMR data of 1, 2, 27, 28, and natural amphirionin-2

**Table S4** $^{1}$H NMR data of **1**, **2**, **27**, **28**, and natural amphirionin-2.

| Position | $^{1}$H NMR (500 MHz, $C_6D_6$)$^{a}$ | | | | |
|---|---|---|---|---|---|
| | Authentic | Compound **1** | Compound **2** | Compound **27** | Compound **28** |
| | $\delta_H$/ppm | $\delta_H$/ppm | $\delta_H$/ppm | $\delta_H$/ppm | $\delta_H$/ppm |
| 1 | 5.08 | **5.18** | **5.18** | 5.09 | 5.09 |
| | 5.05 | 5.11 | 5.11 | 5.06 | 5.06 |
| 2 | 5.81 | **5.92** | **5.92** | 5.82 | 5.82 |
| 3 | 2.30 | **2.55** | **2.55** | 2.30 | 2.30 |
| | 1.92 | **2.08** | **2.08** | 1.93 | 1.93 |
| 4 | 1.54 | **1.61** | **1.61** | 1.53 | 1.53 |
| 5 | 3.83$^{b}$ | 3.83 | 3.83 | 3.83 | 3.83 |
| 6 | 1.68 | **1.44** | **1.44** | 1.69 | 1.69 |
| | 1.42 | **1.33** | **1.33** | 1.42 | 1.41 |
| 7 | 4.41 | **4.13** | **4.13** | 4.41 | 4.41 |
| 8 | 2.09 | 2.04 | 2.05 | 2.07 | 2.07 |
| | 1.48 | **1.25** | **1.25** | 1.48 | 1.48 |
| 9 | 4.43 | 4.43 | 4.43 | 4.42 | 4.42 |
| 10 | 4.54 | **4.33** | **4.34** | 4.54 | 4.54 |
| 11 | 2.18 | **2.01** | **2.02** | 2.17 | 2.16 |
| | 1.49 | **1.40** | **1.41** | 1.48 | 1.47 |
| 12 | 4.99 | **4.91** | **4.91** | 5.00 | 5.00 |
| 13 | 5.55 | 5.53 | 5.53 | 5.56 | 5.56 |
| 14 | | | | | |
| 15 | 6.21 | 6.22 | 6.22 | 6.22 | 6.22 |
| 16 | 5.73 | 5.78 | 5.76 | 5.75 | 5.75 |
| 17 | 2.37 | 2.38 | 2.38 | 2.38 | 2.38 |
| | 2.23 | 2.25 | 2.24 | 2.23 | 2.23 |
| 18 | 4.16 | 4.19 | 4.17 | 4.17 | 4.18 |
| 19 | 2.11 | 2.12 | 2.12 | 2.12 | 2.12 |
| | 1.39 | 1.40 | 1.40 | 1.39 | 1.40 |
| 20 | 4.58 | 4.56 | 4.55 | 4.56 | 4.56 |
| 21 | 4.55 | 4.54 | 4.54 | 4.54 | 4.54 |
| 22 | 2.19 | 2.21 | 2.20 | 2.20 | 2.20 |
| | 1.54 | 1.55 | 1.55 | 1.56 | 1.56 |
| 23 | 4.56 | 4.58 | 4.59 | 4.58 | 4.58 |
| 24 | 5.51 | 5.51 | 5.51 | 5.51 | 5.51 |

| | | | | | |
|---|---|---|---|---|---|
| 25 | 5.70 | 5.70 | 5.71 | 5.71 | 5.71 |
| 26 | 1.98 | 1.98 | 1.99 | 1.99 | 1.99 |
| 27 | 1.33 | 1.33 | 1.34 | 1.34 | 1.34 |
| 28 | 1.24 | 1.24 | 1.24 | 1.24 | 1.24 |
| 29 | 1.26 | 1.28 | 1.26 | 1.26 | 1.26 |
| 30 | 0.89 | 0.90 | 0.90 | 0.90 | 0.90 |
| 31 | 0.98 | 1.07 | 1.07 | 0.99 | 0.99 |
| 32 | 1.74 | 1.75 | 1.74 | 1.74 | 1.74 |
| 5-OH | | 3.55 (s) | 3.54 (s) | 2.07 (br) | 2.07 (br) |

[a]$C_6\mathit{H}D_5$: δ = 7.20 ppm. [b]Incorrectly reported in the original isolation paper.

## 13. Comparison of $^{13}$C NMR data of 1, 2, 27, 28, and natural amphirionin-2

**Table S5** $^{13}$C NMR data of **1**, **2**, **27**, **28**, and natural amphirionin-2.

| Position | $^{13}$C NMR (125 MHz, $C_6D_6$)[a] | | | | |
|---|---|---|---|---|---|
| | Authentic | Cpd **1** | Cpd **2** | Cpd **27** | Cpd **28** |
| | $\delta_C$/ppm | $\delta_C$/ppm | $\delta_C$/ppm | $\delta_C$/ppm | $\delta_C$/ppm |
| 1 | 115.9 | 115.9 | 115.9 | 115.9 | 115.9 |
| 2 | 137.9 | 138.3 | 138.3 | 137.9 | 137.9 |
| 3 | 38.2 | 38.1 | 38.1 | 38.2 | 38.2 |
| 4 | 39.1 | 39.4 | 39.3 | 39.0 | 39.0 |
| 5 | 71.2 | **74.4** | **74.4** | 71.2 | 71.2 |
| 6 | 39.2 | **40.1** | **40.1** | 39.1 | 39.1 |
| 7 | 78.3 | **81.5** | **81.5** | 78.3 | 78.3 |
| 8 | 41.2 | **42.4** | **42.4** | 41.1 | 41.1 |
| 9 | 84.0 | **83.0** | **83.0** | 84.0 | 84.0 |
| 10 | 83.8 | **84.6** | **84.6** | 83.8 | 83.8 |
| 11 | 42.4 | 42.2 | 42.2 | 42.5 | 42.5 |
| 12 | 76.8 | 76.6 | 76.6 | 76.8 | 76.8 |
| 13 | 131.5 | 131.4 | 131.4 | 131.6 | 131.6 |
| 14 | 135.7 | 135.7 | 135.8 | 135.7 | 135.7 |
| 15 | 136.8 | 136.7 | 136.7 | 136.8 | 136.8 |
| 16 | 125.8 | 126.0 | 126.0 | 125.8 | 125.8 |
| 17 | 39.4 | 39.3 | 39.5 | 39.5 | 39.5 |
| 18 | 79.9 | 79.9 | 79.9 | 79.9 | 79.9 |
| 19 | 41.3 | 41.3 | 41.3 | 41.3 | 41.3 |
| 20 | 84.1 | 83.9 | 83.9 | 83.9 | 83.9 |
| 21 | 83.9 | 84.1 | 84.1 | 84.1 | 84.1 |
| 22 | 42.5 | 42.5 | 42.5 | 42.4 | 42.4 |
| 23 | 80.9 | 80.9 | 80.9 | 80.9 | 80.9 |
| 24 | 131.1 | 131.1 | 131.1 | 131.1 | 131.1 |
| 25 | 132.5 | 132.5 | 132.5 | 132.5 | 132.5 |
| 26 | 32.5 | 32.5 | 32.5 | 32.5 | 32.5 |
| 27 | 29.2 | 29.2 | 29.2 | 29.2 | 29.2 |
| 28 | 31.6 | 31.6 | 31.6 | 31.6 | 31.6 |
| 29 | 22.8 | 22.8 | 22.8 | 22.8 | 22.8 |
| 30 | 14.2 | 14.2 | 14.2 | 14.2 | 14.2 |
| 31 | 14.0 | 14.1 | 14.0 | 14.0 | 14.0 |
| 32 | 13.1 | 13.1 | 13.1 | 13.1 | 13.1 |

[a]$C_6D_6$: $\delta = 128.0$ ppm.

## 14. Comparison of $^1$H NMR spectra of 1, 2, and natural amphirionin-2

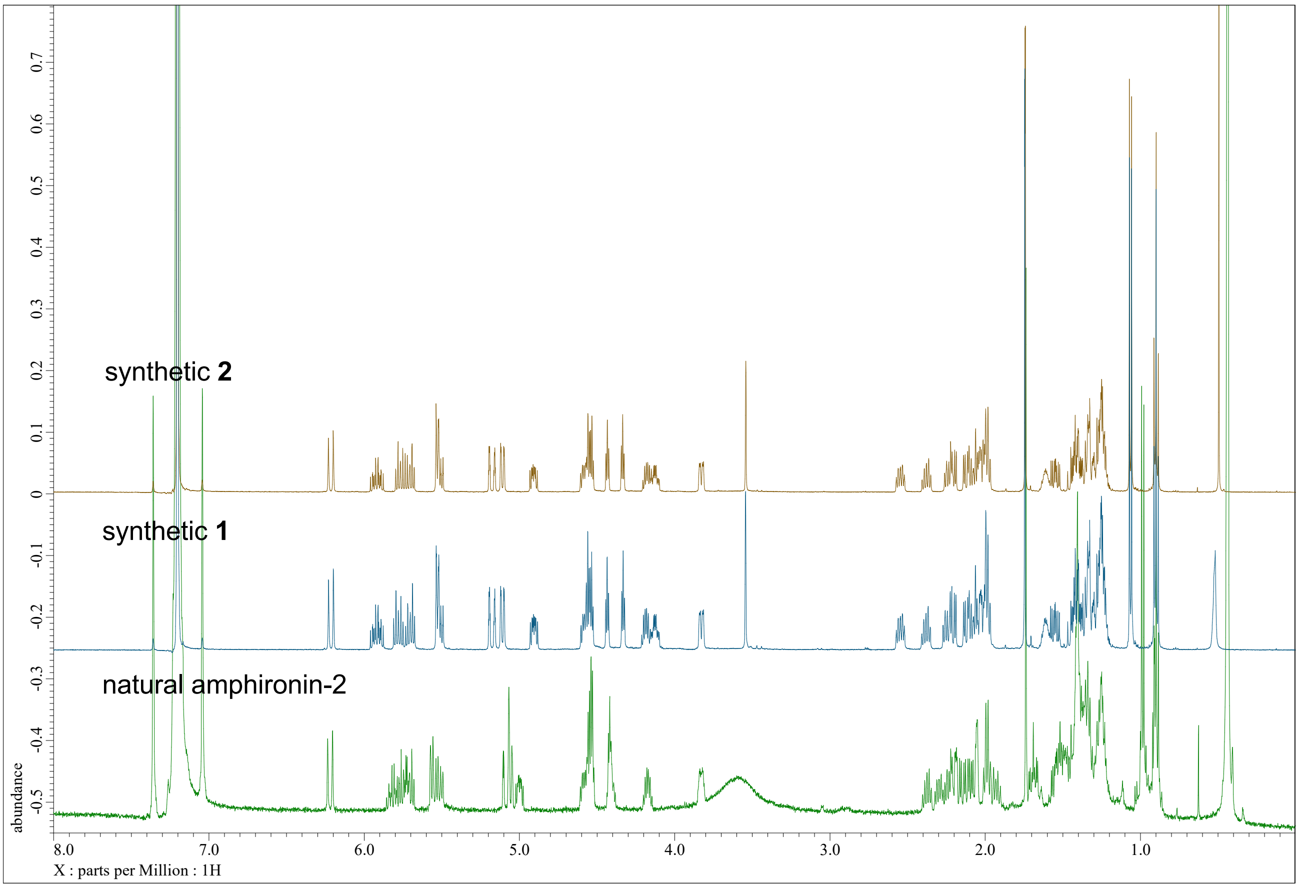


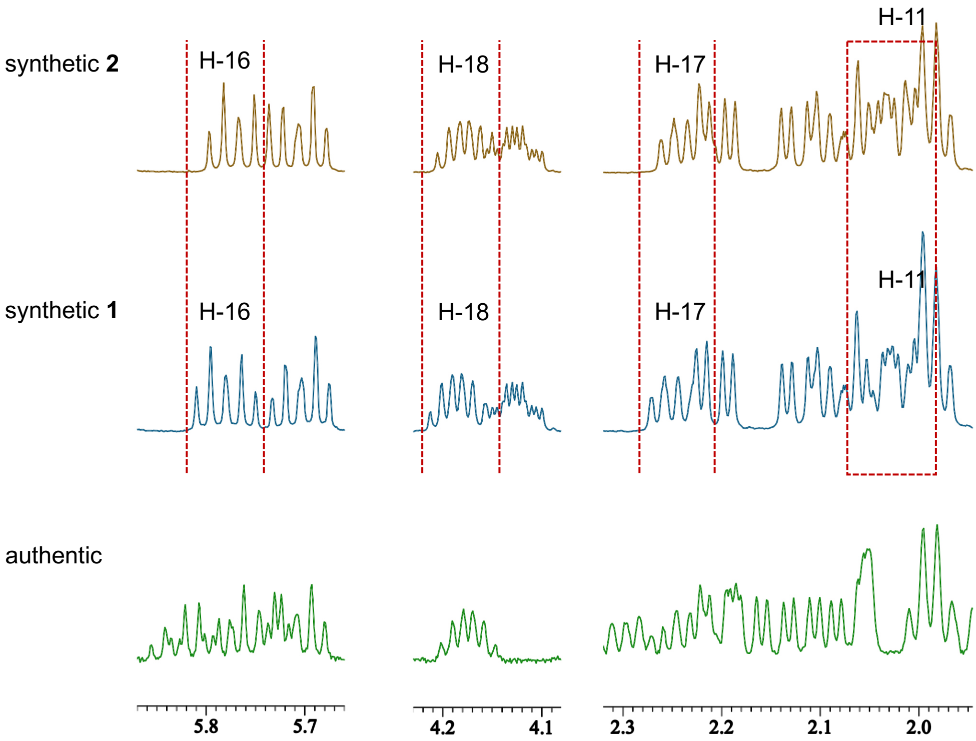


**Fig. S2** Comparison of $^1$H NMR spectra of **1**, **2**, and natural amphirionin-2.

## 15. Comparison of $^{1}$H NMR spectra of 27, 28, and natural amphirionin-2

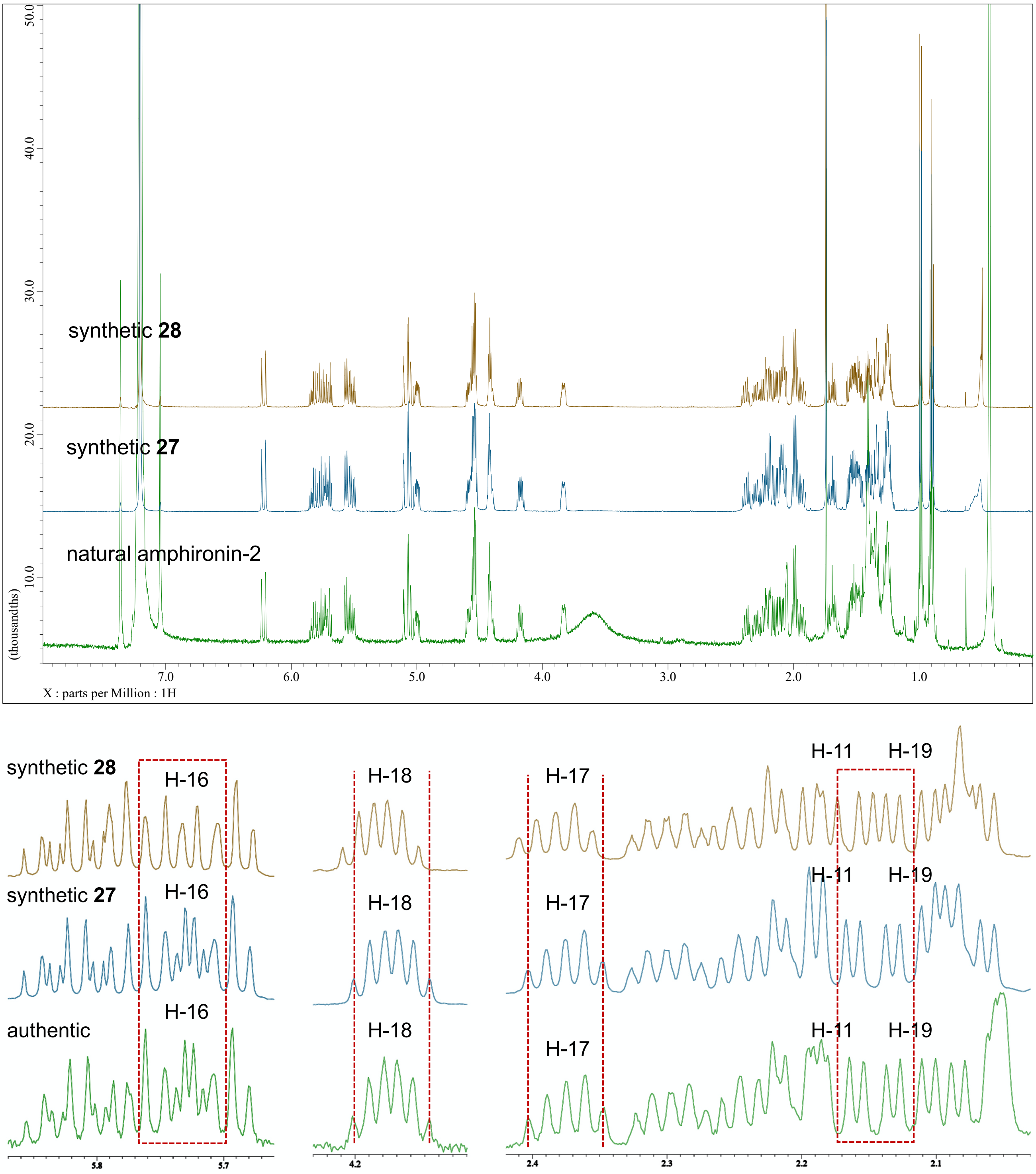


**Fig. S3** Comparison of $^{1}$H NMR spectra of **27**, **28**, and natural amphirionin-2 (500 MHz, $C_6D_6$). The $^{1}$H NMR spectrum of synthetic **27** was in accordance with that of natural amphirionin-2. The $^{1}$H NMR spectrum of synthetic **28** differed from that of natural amphirionin-2 with respect to the signals of H-11 (δ 2.16 ppm), H-16 (δ 5.75 ppm), H-17 (δ 2.38 ppm), H-18 (δ 4.18 ppm), and H-19 (δ 2.12 ppm).

Inconsistency observed around 2.04–2.10 ppm is ascribable to 5-OH signal (see Fig. S4).

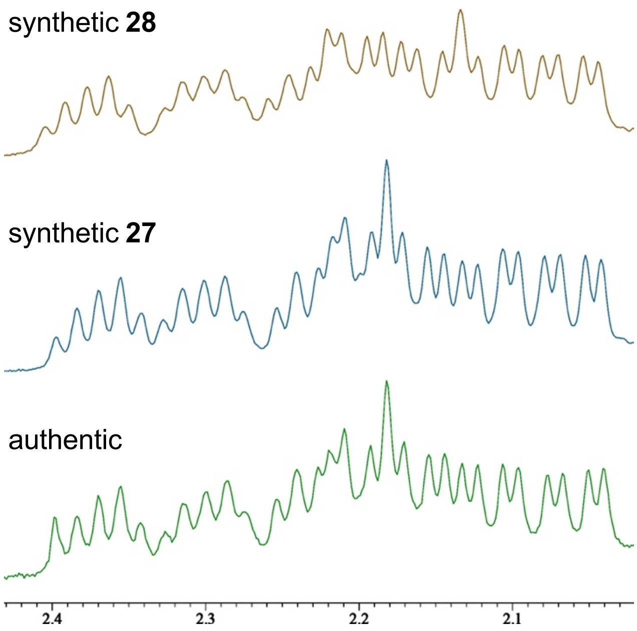


**Fig. S4** Comparison of $^1$H NMR spectra of **27**, **28**, and natural amphirionin-2 (500 MHz, $C_6D_6$ + $D_2O$ (one drop)). Inconsistency observed around 2.04–2.10 ppm of Fig. S3 was solved by the addition of $D_2O$.

## 16. Chiral HPLC analysis of 27, 28, and natural amphirionin-2

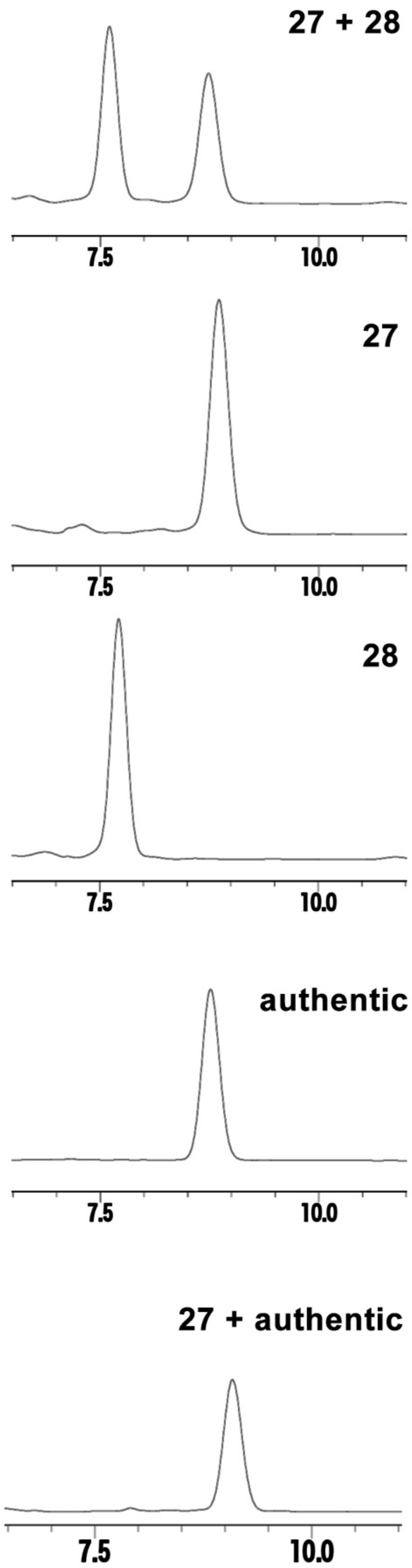


**Fig. S5** Chiral HPLC chromatogram of **27**, **28**, and natural amphironin-2. The chiral HPLC analysis (column: CHIRALPAK IB N-5 (4.6 mm I.D. × 250 mm); eluent: 10% *i*-PrOH/*n*-hexane; flow rate: 1.0

mL min$^{-1}$; UV detection: 254 nm) showed the retention time of **27**, **28**, and natural amphirionin-2 to be 8.9, 7.7, 8.8 min, respectively. Co-injection of synthetic **27** and natural amphirionin-2 resulted in a single peak.

## 17. CD spectra of 1, 2, 27, 28, and natural amphirionin-2

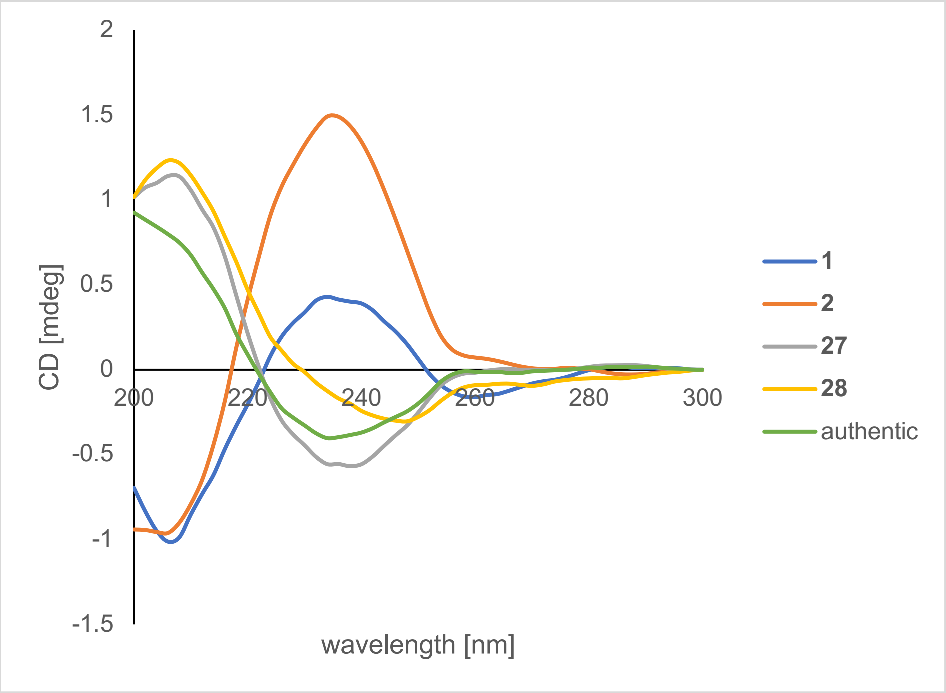


**Fig. S6** CD spectra of **1**, **2**, **27**, **28**, and natural amphirionin-2. The spectra were measured at a concentration of 10 μg/mL in MeOH.

## 18. Cell culture experiments

All tested compounds were purified by preparative reverse-phase HPLC prior to use in cell culture experiments. A549, Jurkat, and K562 cells were purchased from RIKEN Bio-Resource Center, and HeLa cells were kindly provided by Professor Kiyotake Suenaga (Keio University).

A549 and HeLa cells: Cells were cultured at 37 °C with 5% $CO_2$ in RPMI1640 with L-Gln (Nakalai Tesque, Japan) supplemented with 10% heat-inactivated FBS, 100 units/mL penicillin, and 100 μg/mL streptomycin. Cells were seeded at 5 × $10^3$ cells/well in 96-well plates and cultured overnight. Cells were then treated with various concentrations of compound solutions in DMSO and incubated for 72 h for HeLa cells, and 96 h for A549 cells. Cell proliferation was measured by the WST-8 assay. The assays were performed in triplicate and repeated at least three times. Doxorubicin was used as a positive control: $IC_{50}$ 0.60 μM for A549 cells and 0.44 μM for HeLa cells.

Jurkat and K562 cells: Cells were cultured at 37 °C with 5% $CO_2$ in RPMI1640 with L-Gln (Nakalai Tesque, Japan) supplemented with 10% heat-inactivated FBS, 100 units/mL penicillin, and 100 μg/mL streptomycin. Cells were seeded at 1–2 × $10^4$ cells/well in 96-well plates, treated with various concentrations of compound solutions in DMSO and incubated for 48 h. Cell proliferation was measured by the WST-8 assay. The assays were performed in triplicate and repeated at least three times. Doxorubicin was used as a positive control: $IC_{50}$ 0.18 μM for Jurkat cells and 0.084 μM for K562 cells.

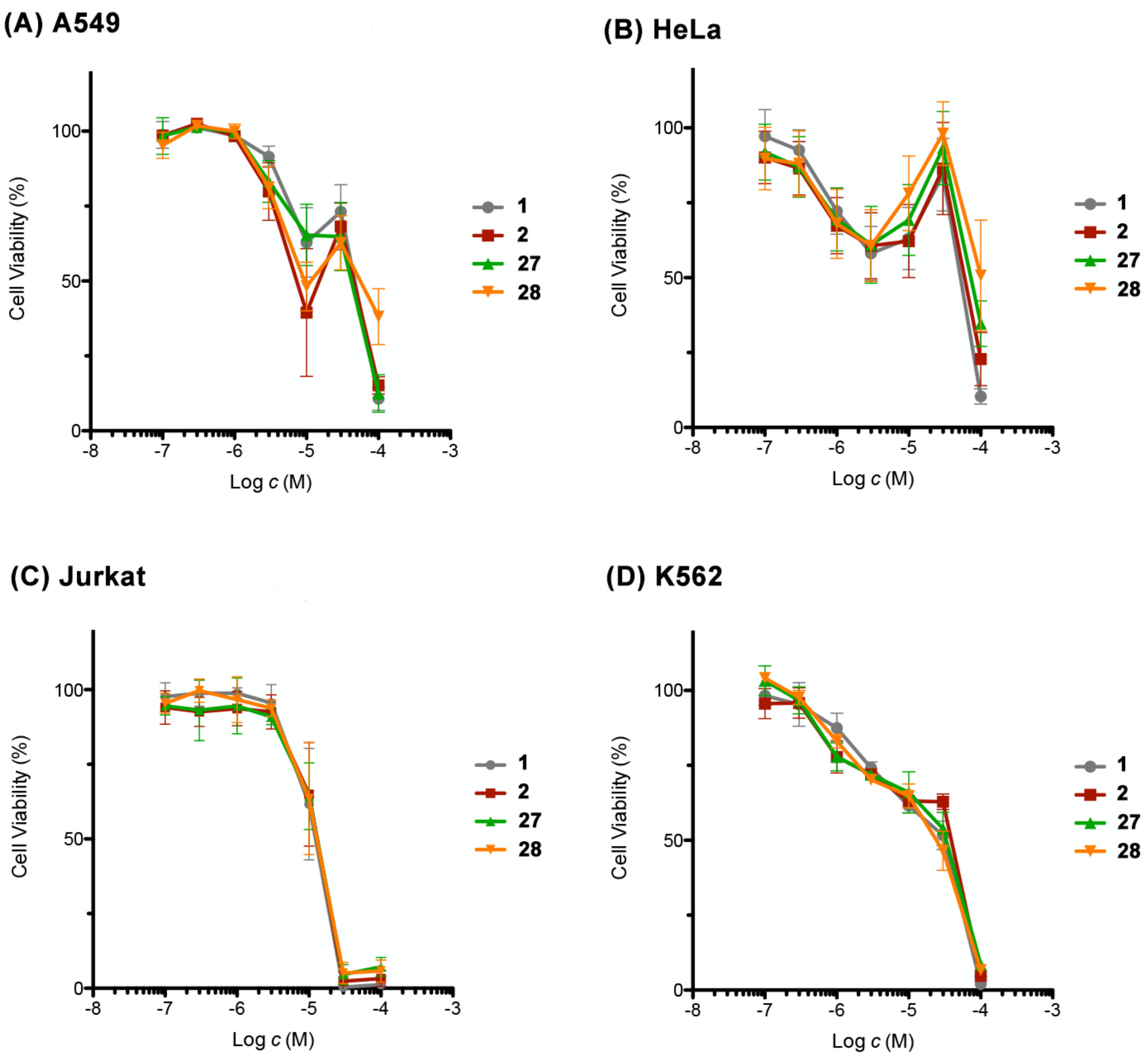


**Fig. S7** Cytotoxic activity of synthetic amphirionin-2 (**27**) and its diastereomers **1**, **2**, and **28**.

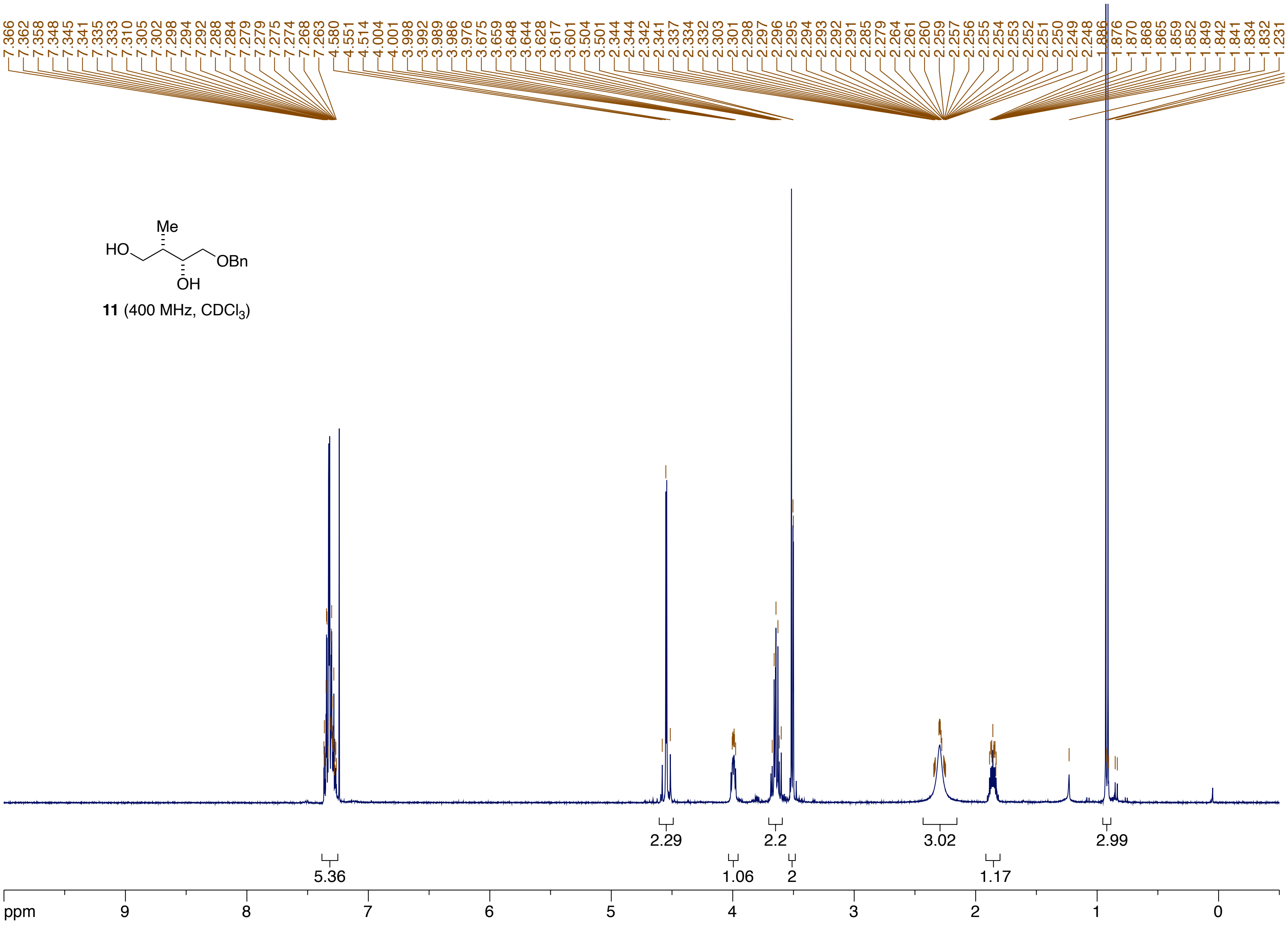
7.366
7.362
7.358
7.348
7.345
7.341
7.335
7.333
7.310
7.305
7.302
7.298
7.294
7.292
7.288
7.284
7.279
7.279
7.275
7.274
7.268
7.263
4.580
4.551
4.514
4.004
4.001
3.998
3.992
3.989
3.986
3.976
3.675
3.659
3.648
3.644
3.628
3.617
3.601
3.504
3.501
2.344
2.344
2.342
2.341
2.337
2.334
2.332
2.303
2.301
2.298
2.297
2.296
2.295
2.294
2.293
2.292
2.291
2.285
2.279
2.264
2.261
2.260
2.259
2.257
2.256
2.255
2.254
2.253
2.252
2.251
2.250
2.249
2.248
1.886
1.876
1.870
1.868
1.865
1.859
1.852
1.849
1.842
1.841
1.834
1.832
1.231
Me
HO
OBn
OH
**11** (400 MHz, $CDCl_3$)
2.29
2.2
3.02
2.99
5.36
1.06
2
1.17
ppm
9
8
7
6
5
4
3
2
1
0

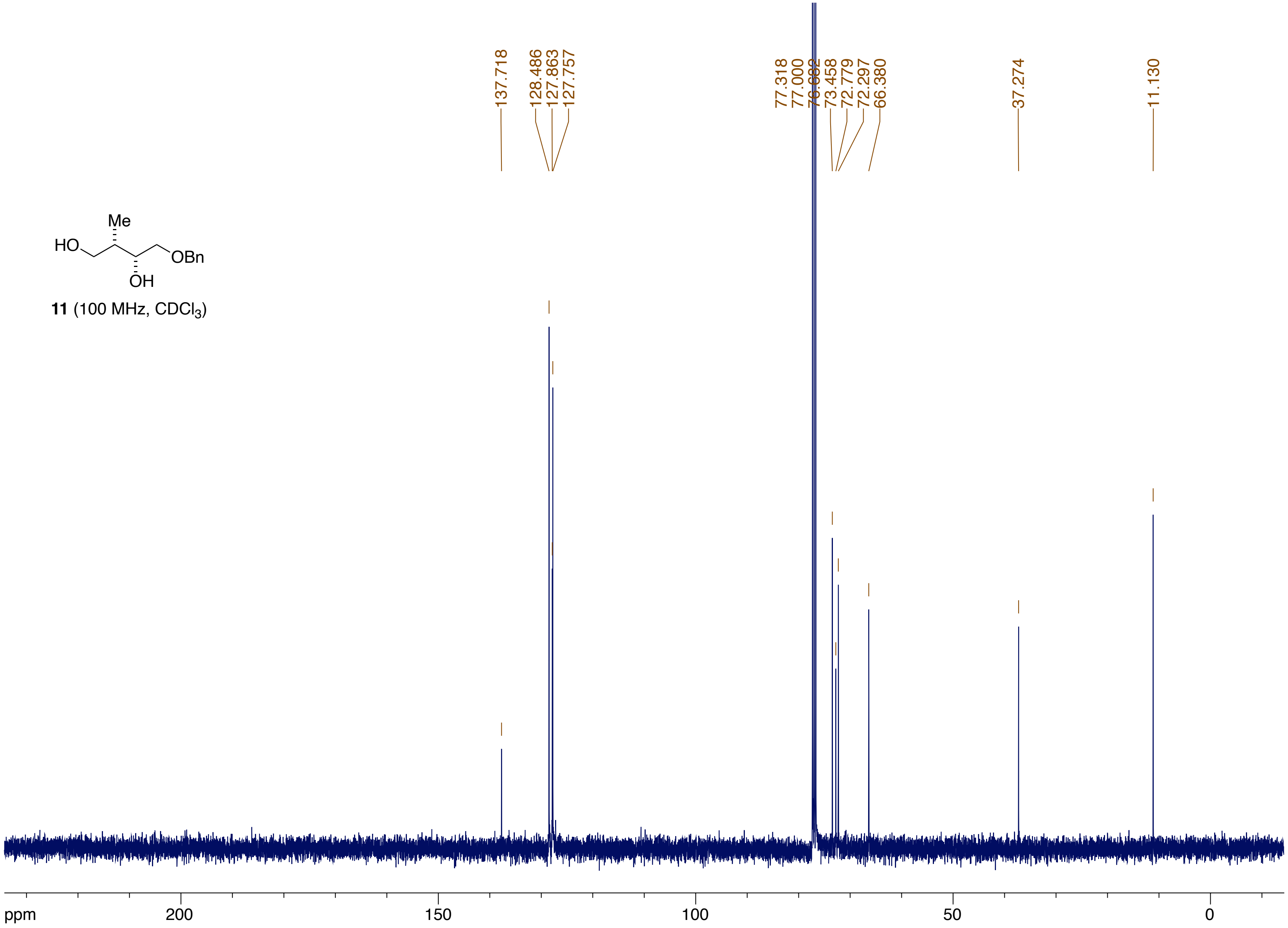
137.718
128.486
127.863
127.757
77.318
77.000
76.682
73.458
72.779
72.297
66.380
37.274
11.130
Me
HO
OBn
OH
11 (100 MHz, CDCl$_3$)
ppm
200
150
100
50
0

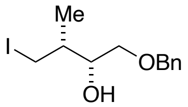


**12** (400 MHz, $CDCl_3$)

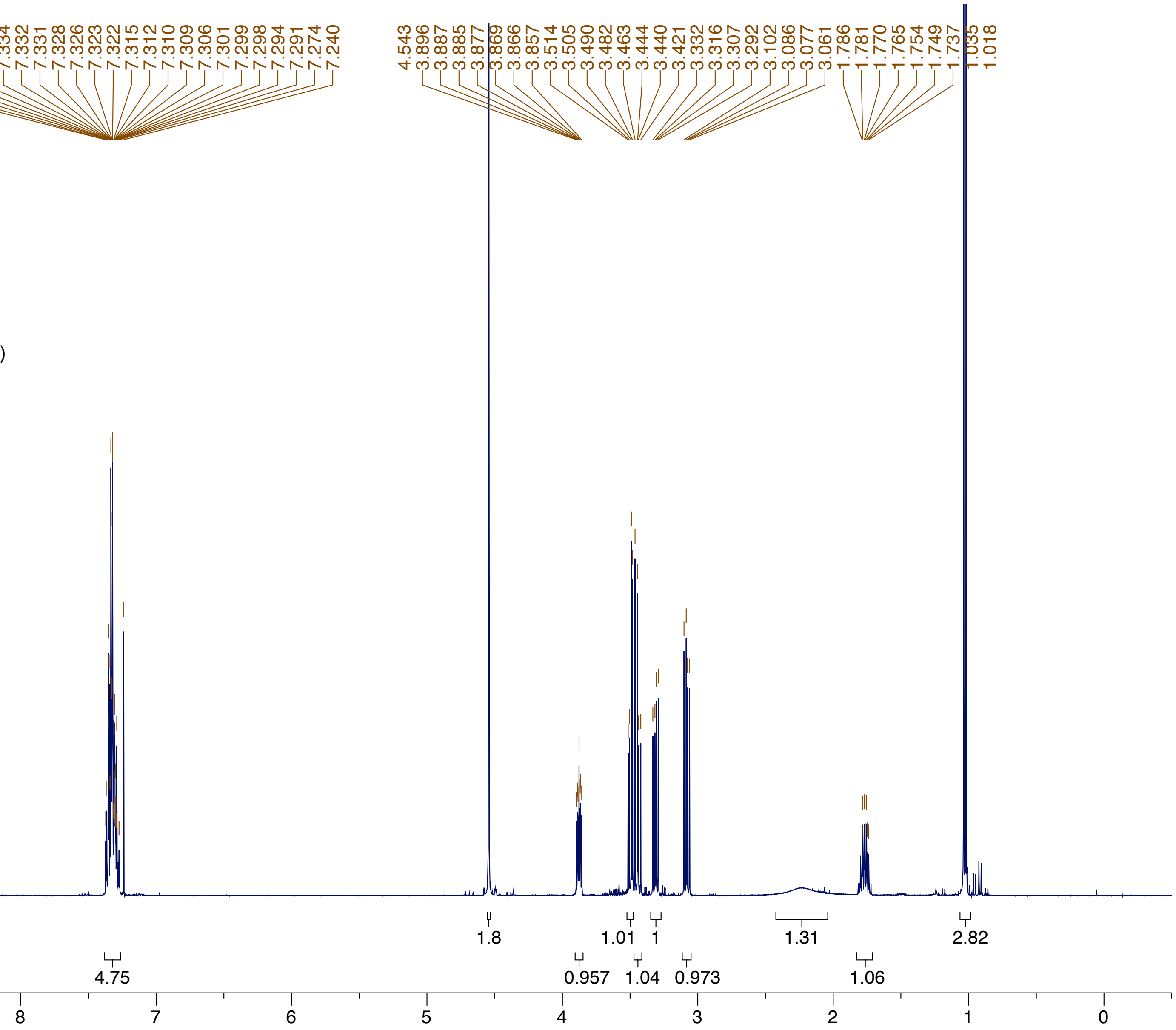

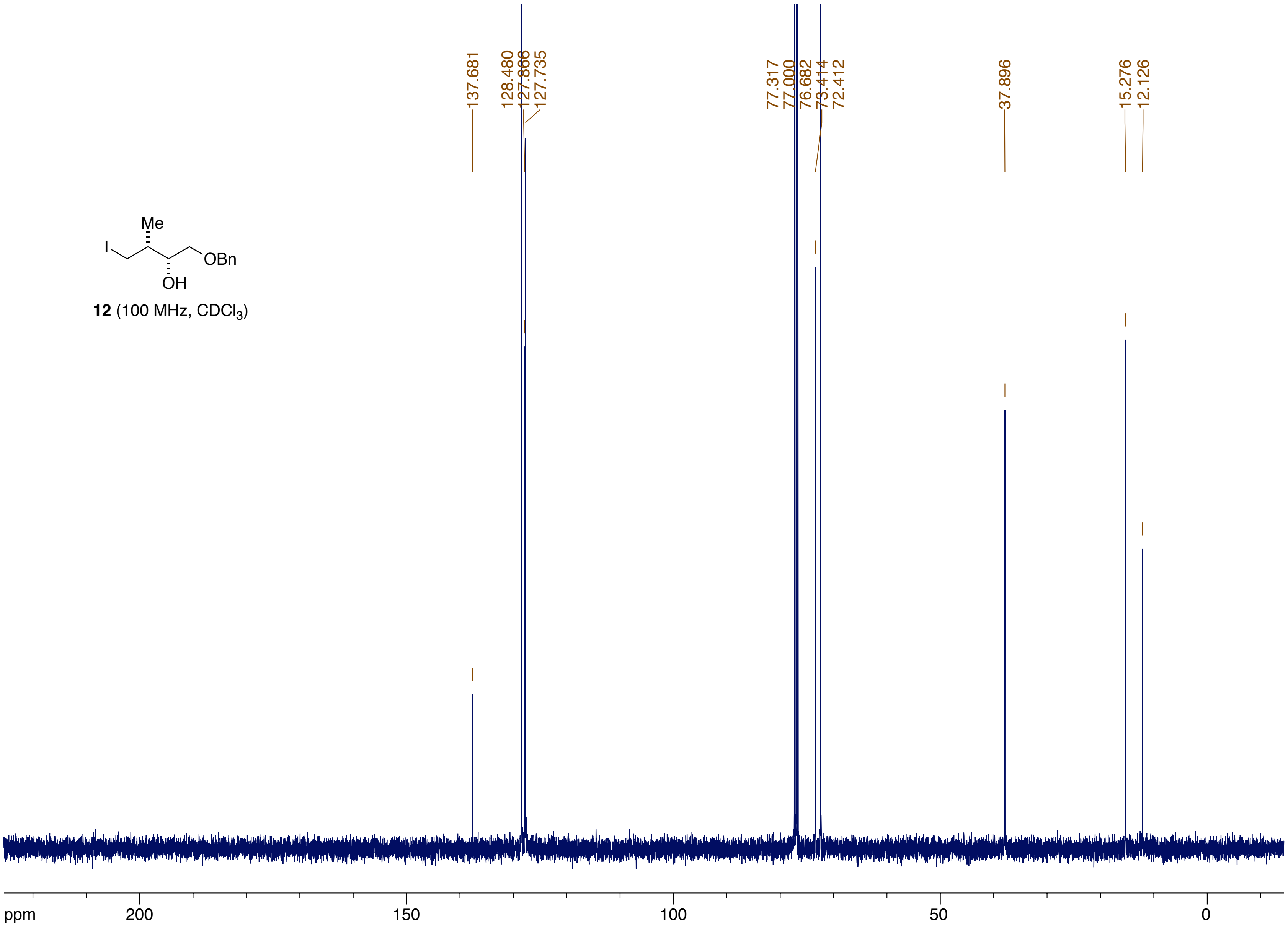

**12** (100 MHz, $CDCl_3$)

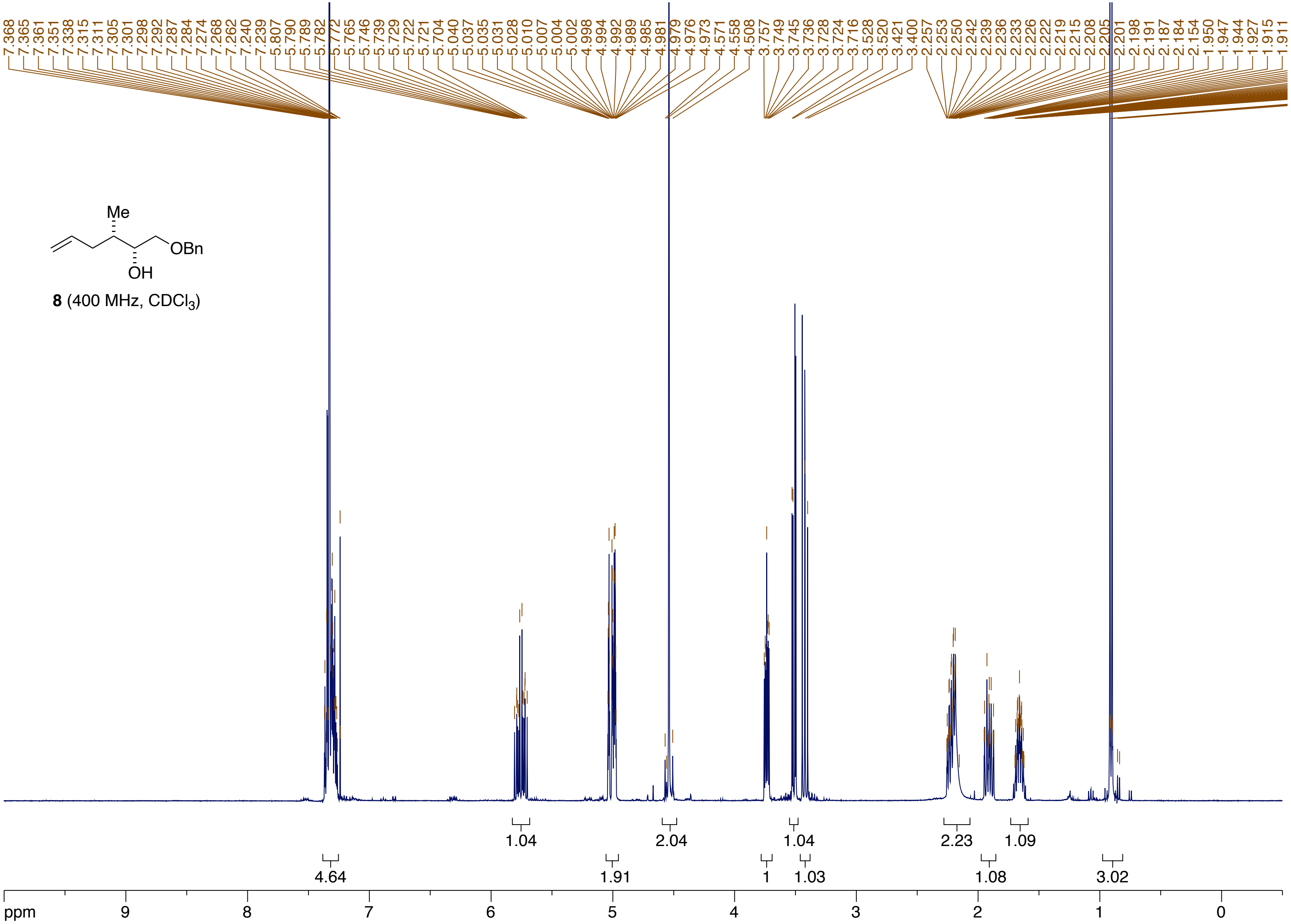
7.368 7.365 7.361 7.351 7.338 7.315 7.311 7.305 7.301 7.298 7.292 7.287 7.284 7.274 7.268 7.262 7.240 7.239 5.807 5.790 5.789 5.782 5.772 5.765 5.746 5.739 5.729 5.722 5.721 5.704 5.040 5.037 5.035 5.031 5.028 5.010 5.007 5.004 5.002 4.998 4.994 4.992 4.989 4.985 4.981 4.979 4.976 4.973 4.571 4.558 4.508 3.757 3.749 3.745 3.736 3.728 3.724 3.716 3.528 3.520 3.421 3.400 2.257 2.253 2.250 2.242 2.239 2.236 2.233 2.226 2.222 2.219 2.215 2.208 2.205 2.201 2.198 2.191 2.187 2.184 2.154 1.950 1.947 1.944 1.927 1.915 1.911
Me
OBn
OH
8 (400 MHz, CDCl3)
1.04
2.04
1.04
2.23
1.09
4.64
1.91
1
1.03
1.08
3.02
ppm
9
8
7
6
5
4
3
2
1
0

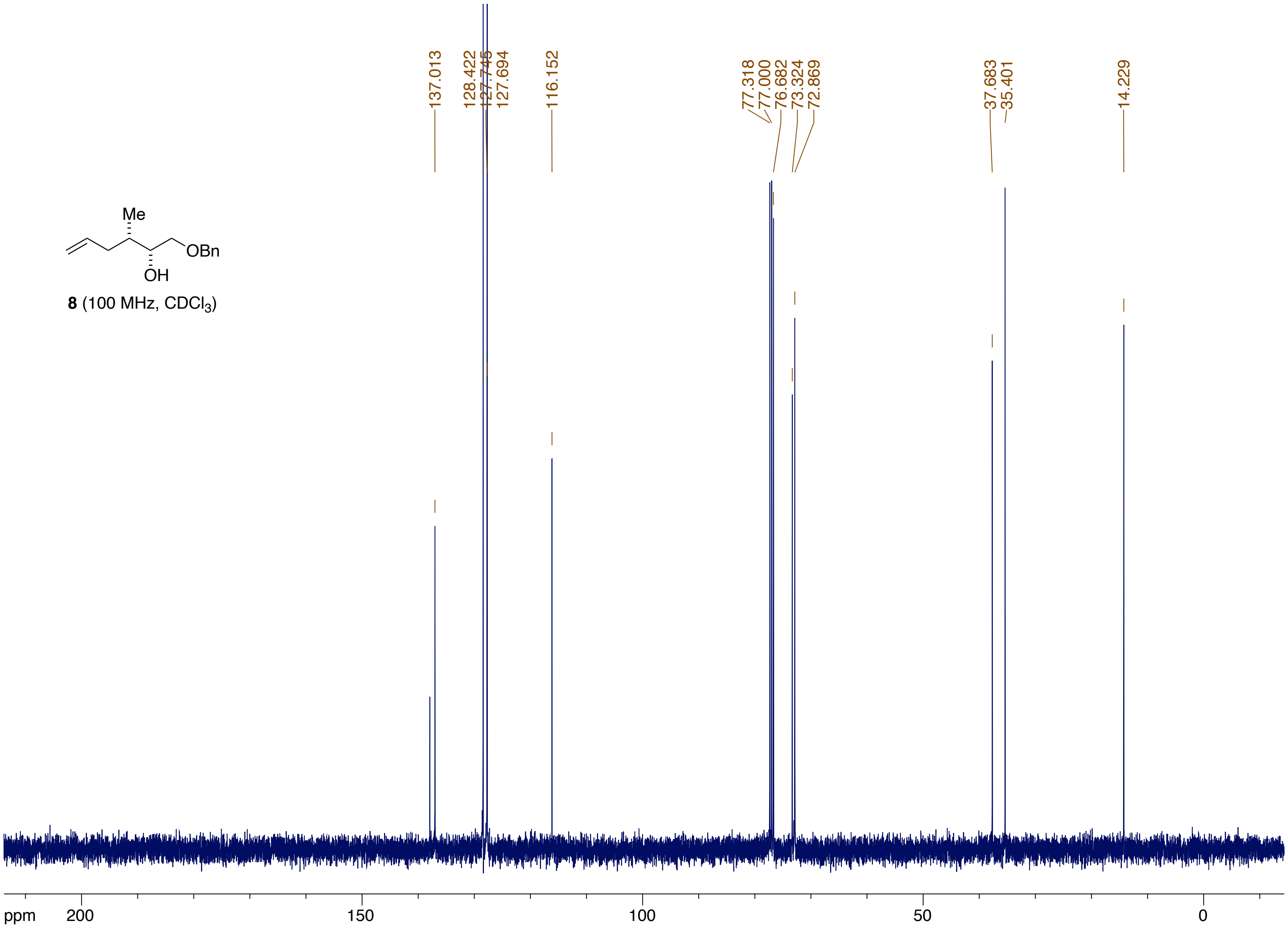


**8** (100 MHz, $CDCl_3$)
137.013
128.422
127.745
127.694
116.152
77.318
77.000
76.682
73.324
72.869
37.683
35.401
14.229
ppm
200
150
100
50
0

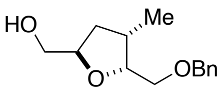


**13** (400 MHz, $CDCl_3$)

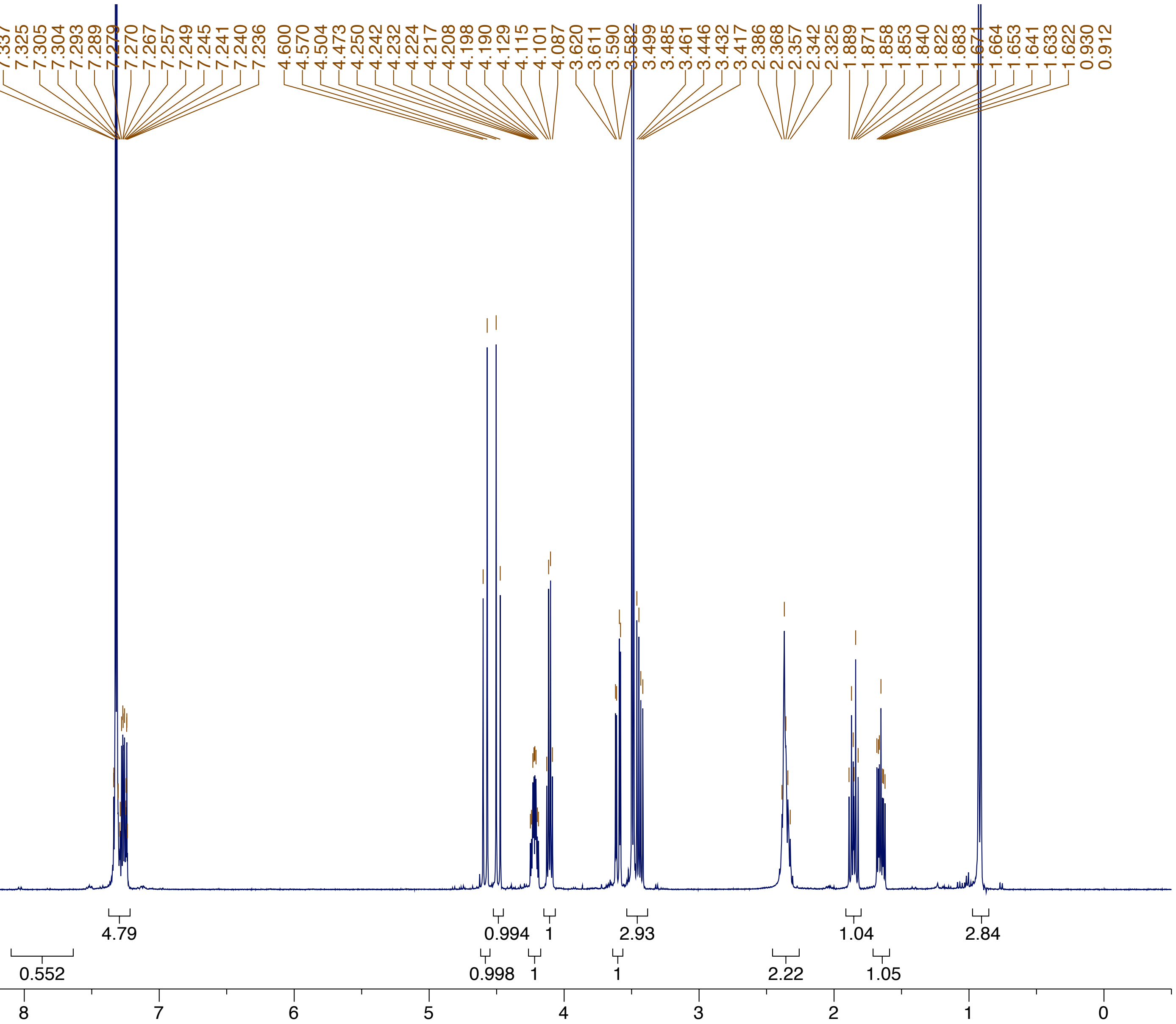

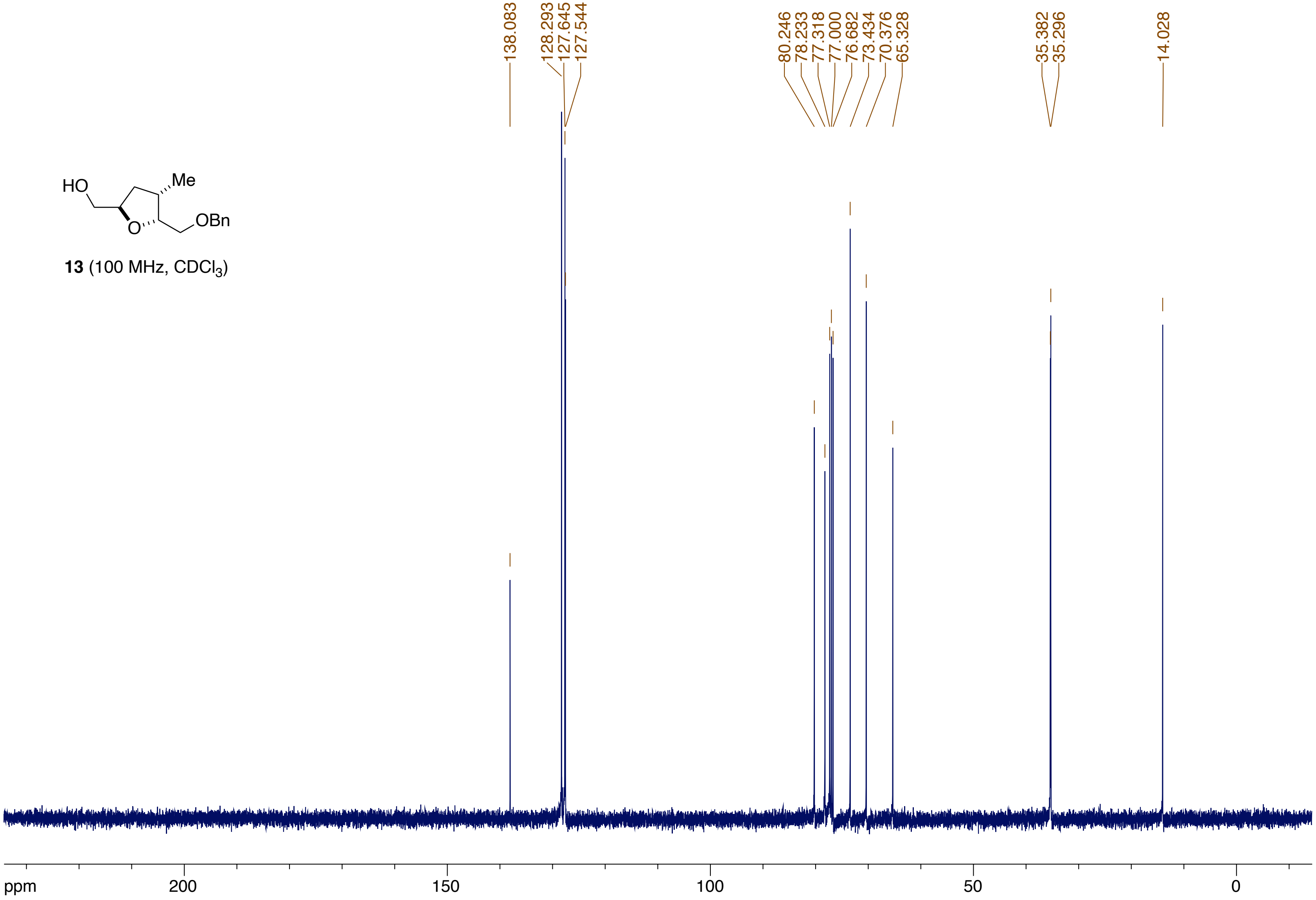


**13** (100 MHz, $CDCl_3$)

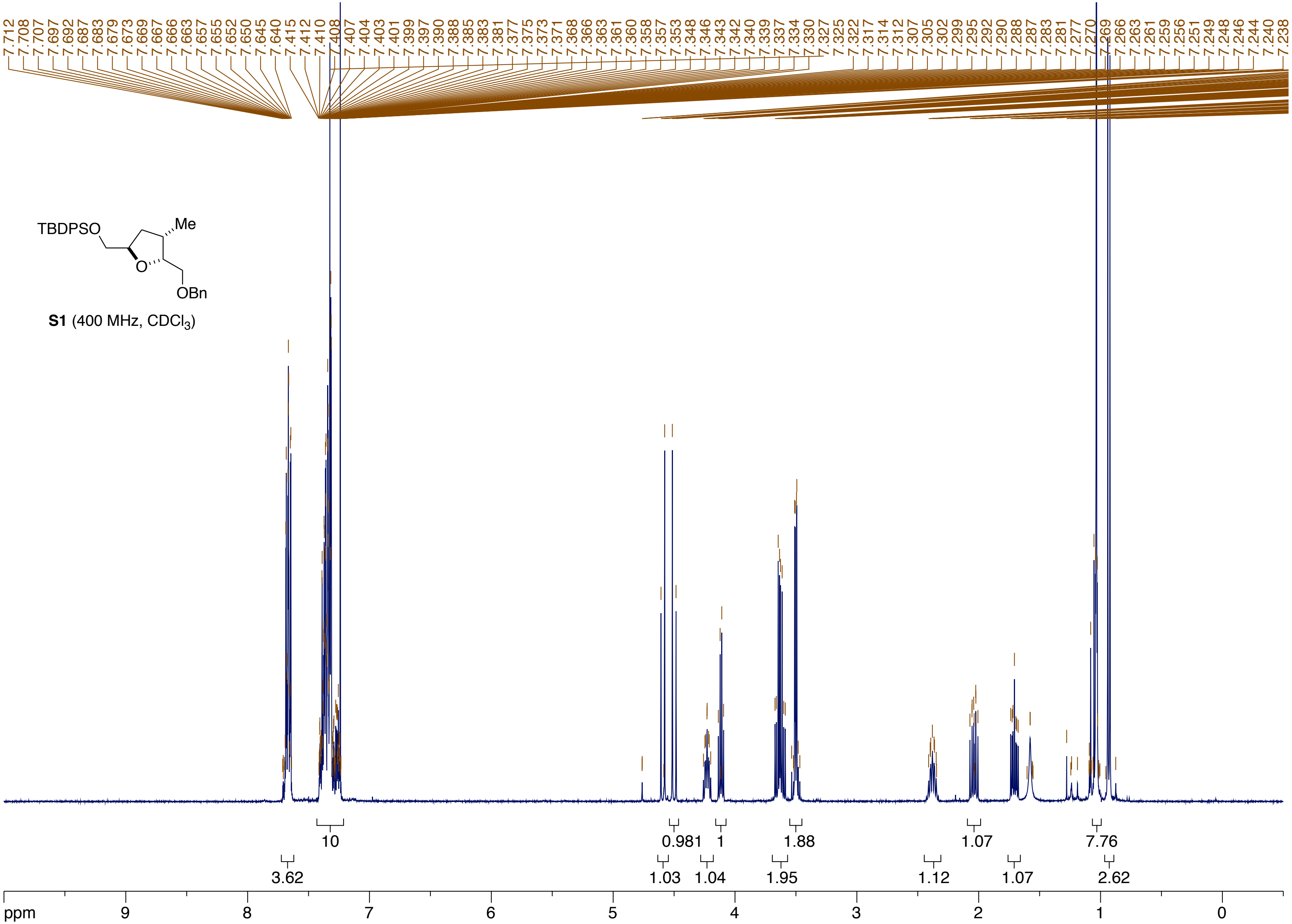
7.712 7.708 7.697 7.692 7.687 7.683 7.679 7.673 7.669 7.667 7.666 7.663 7.657 7.655 7.652 7.650 7.645 7.640 7.415 7.412 7.410 7.408 7.407 7.404 7.403 7.401 7.399 7.397 7.390 7.388 7.385 7.383 7.381 7.377 7.375 7.373 7.371 7.368 7.366 7.363 7.361 7.360 7.358 7.357 7.353 7.348 7.346 7.343 7.342 7.340 7.339 7.337 7.334 7.330 7.327 7.325 7.322 7.317 7.314 7.312 7.307 7.305 7.302 7.299 7.295 7.292 7.290 7.288 7.287 7.283 7.281 7.277 7.270 7.269 7.266 7.263 7.261 7.259 7.256 7.251 7.249 7.248 7.246 7.244 7.240 7.238
TBDPSO
Me
O
OBn
**S1** (400 MHz, $CDCl_3$)
10
0.981
1
1.88
1.07
7.76
3.62
1.03
1.04
1.95
1.12
1.07
2.62
ppm 9 8 7 6 5 4 3 2 1 0

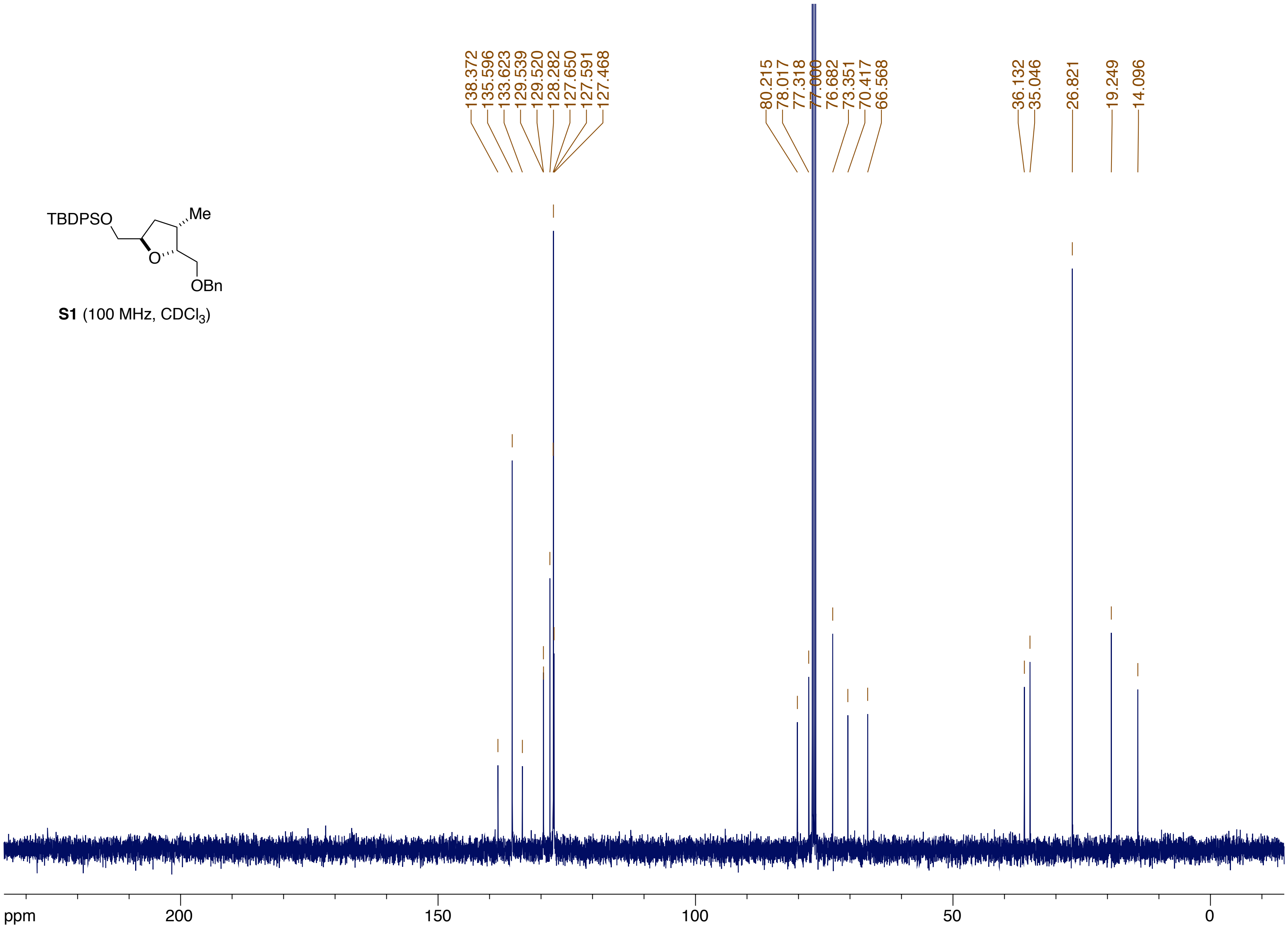


**S1** (100 MHz, $CDCl_3$)

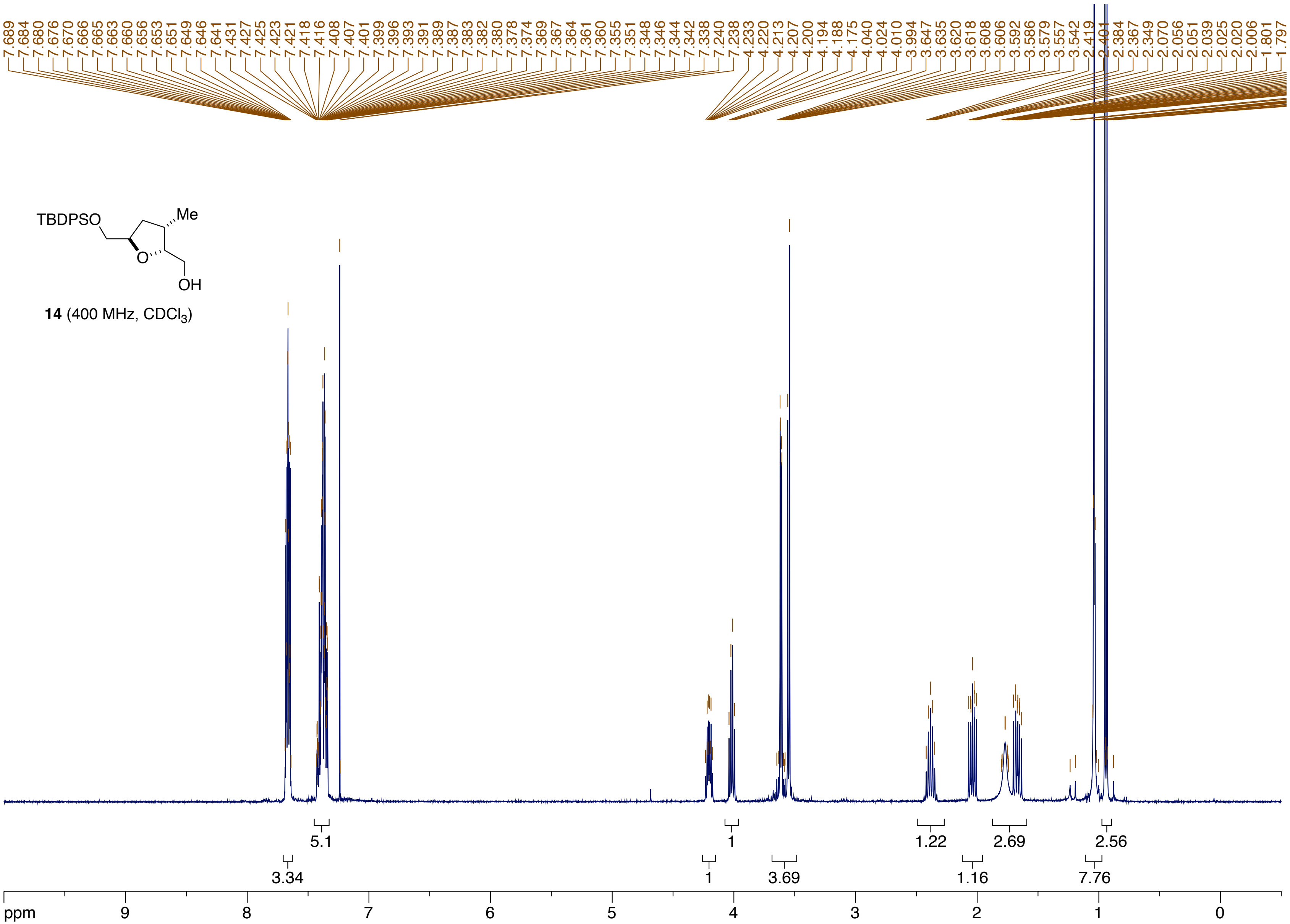
7.689 7.684 7.680 7.676 7.670 7.666 7.665 7.663 7.660 7.656 7.653 7.651 7.649 7.646 7.641 7.431 7.427 7.425 7.423 7.421 7.418 7.416 7.408 7.407 7.401 7.399 7.396 7.393 7.391 7.389 7.387 7.383 7.382 7.380 7.378 7.374 7.369 7.367 7.364 7.361 7.360 7.355 7.351 7.348 7.346 7.344 7.342 7.338 7.240 7.238 4.233 4.220 4.213 4.207 4.200 4.194 4.188 4.175 4.040 4.024 4.010 3.994 3.647 3.635 3.620 3.618 3.608 3.606 3.592 3.586 3.579 3.557 3.542 2.419 2.401 2.384 2.367 2.349 2.070 2.056 2.051 2.039 2.025 2.020 2.006 1.801 1.797
TBDPSO
Me
O
OH
14 (400 MHz, CDCl3)
3.34
5.1
1
1
3.69
1.22
1.16
2.69
7.76
2.56
ppm
9
8
7
6
5
4
3
2
1
0

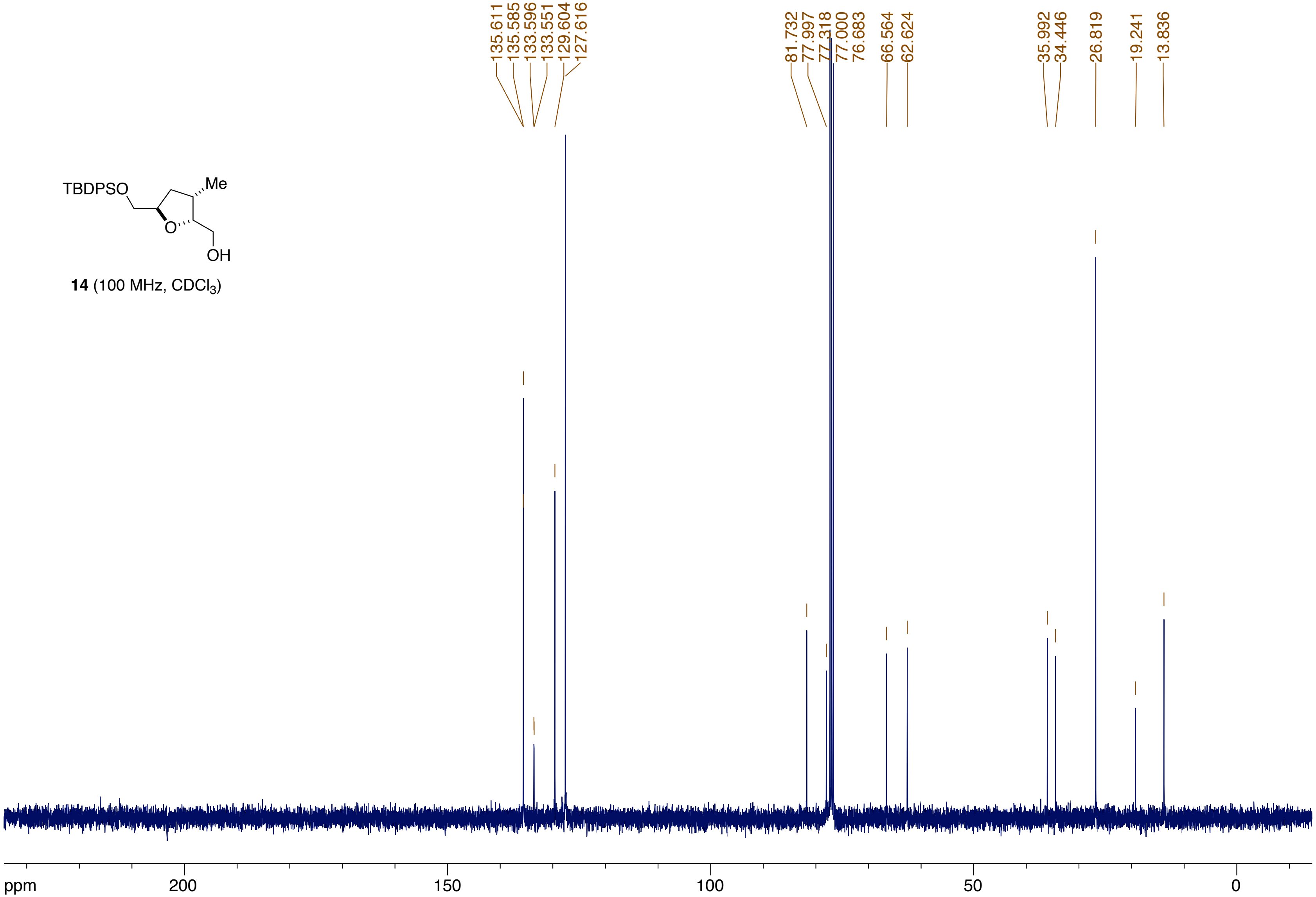


**14** (100 MHz, $CDCl_3$)

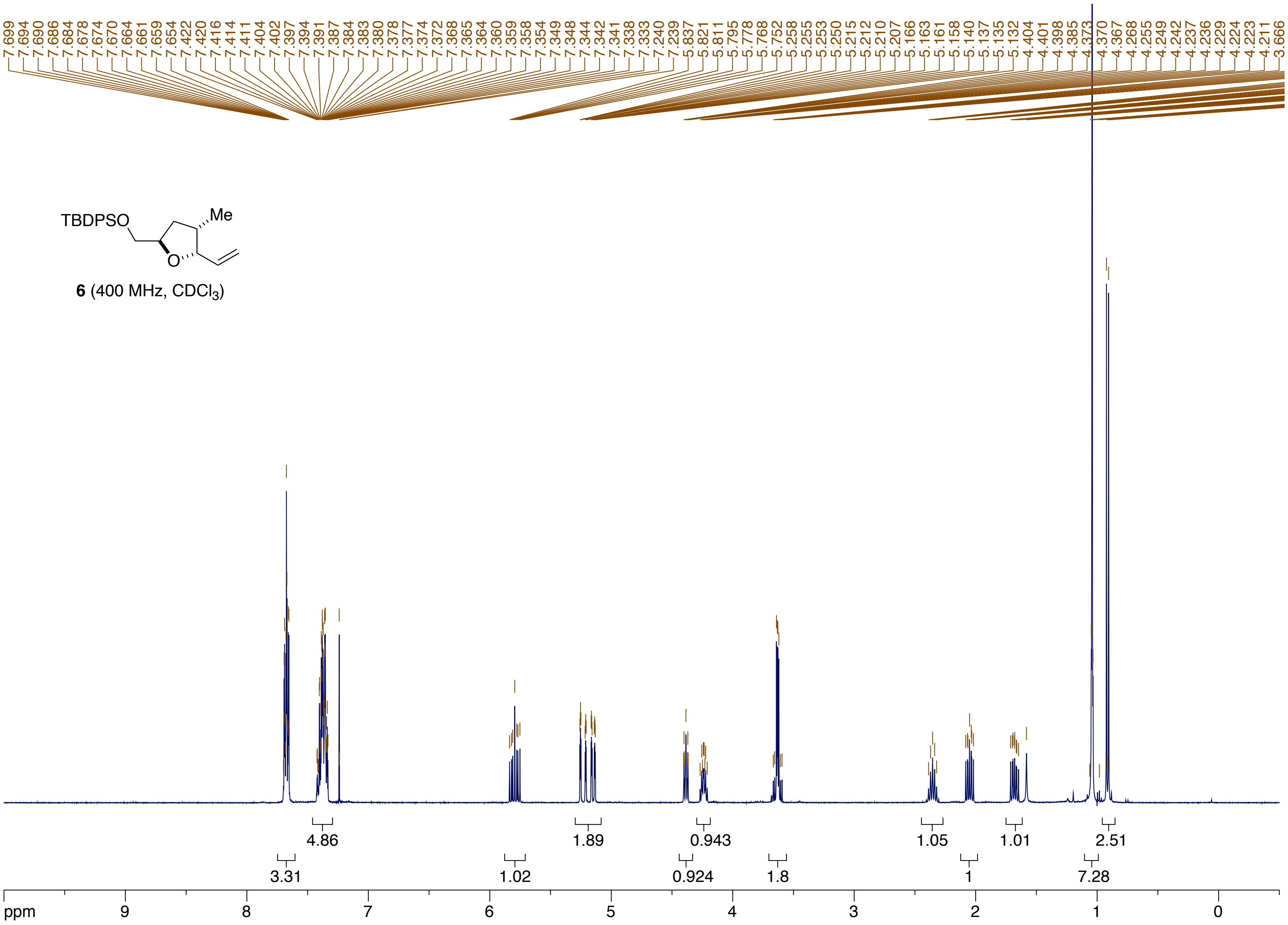
7.699 7.694 7.690 7.686 7.684 7.678 7.674 7.670 7.664 7.661 7.659 7.654 7.422 7.420 7.416 7.414 7.411 7.404 7.402 7.397 7.394 7.391 7.387 7.384 7.383 7.380 7.378 7.377 7.374 7.372 7.368 7.365 7.364 7.360 7.359 7.358 7.354 7.349 7.348 7.344 7.342 7.341 7.338 7.333 7.240 7.239 5.837 5.821 5.811 5.795 5.778 5.768 5.752 5.258 5.255 5.253 5.250 5.215 5.212 5.210 5.207 5.166 5.163 5.161 5.158 5.140 5.137 5.135 5.132 4.404 4.401 4.398 4.385 4.373 4.370 4.367 4.268 4.255 4.249 4.242 4.237 4.236 4.229 4.224 4.223 4.211 3.666
TBDPSO
Me
O
6 (400 MHz, CDCl3)
4.86 1.89 0.943 1.05 1.01 2.51
3.31 1.02 0.924 1.8 1 7.28
ppm 9 8 7 6 5 4 3 2 1 0

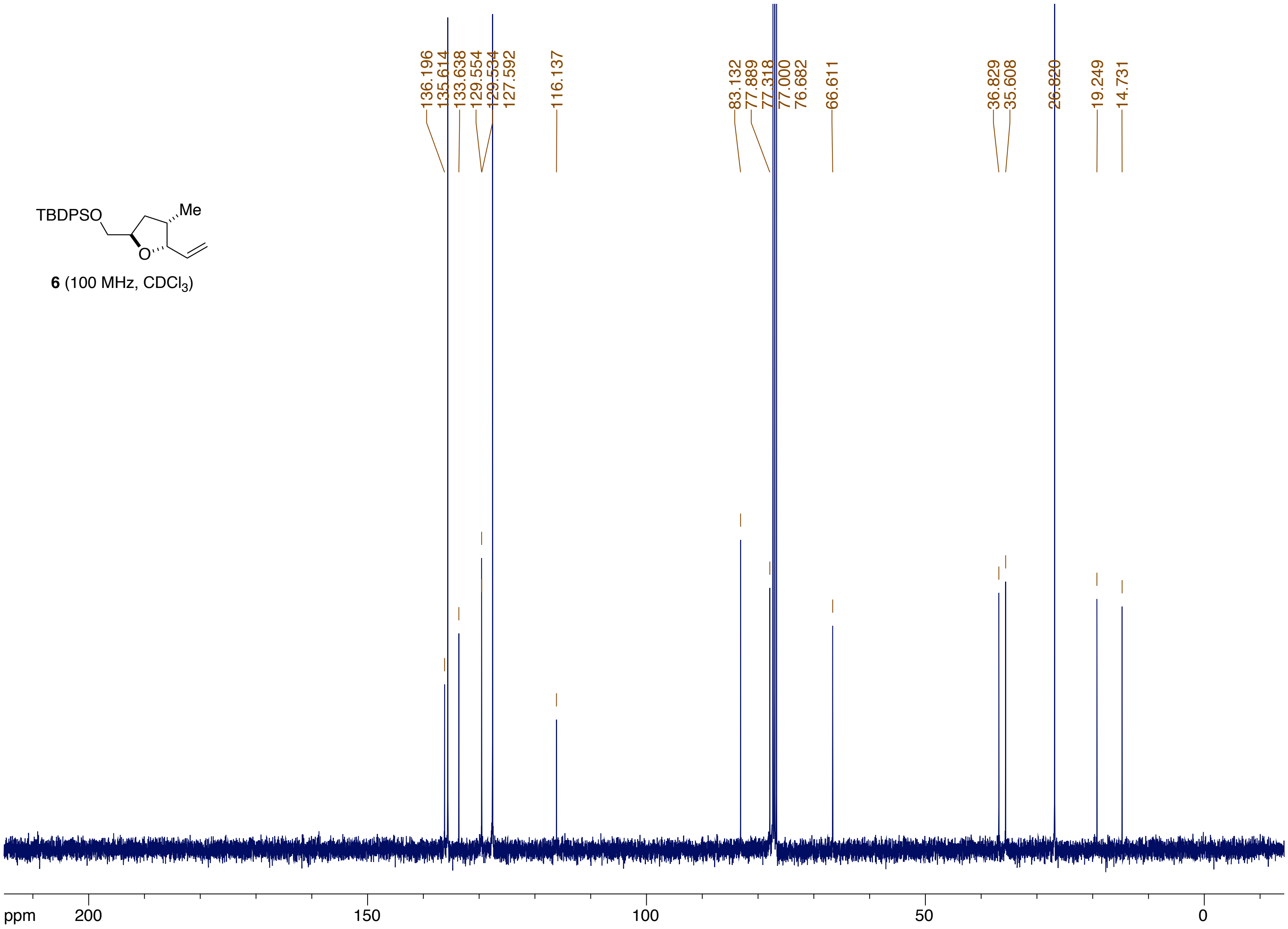


**6** (100 MHz, $CDCl_3$)

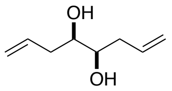

**S6** (400 MHz, $CDCl_3$)

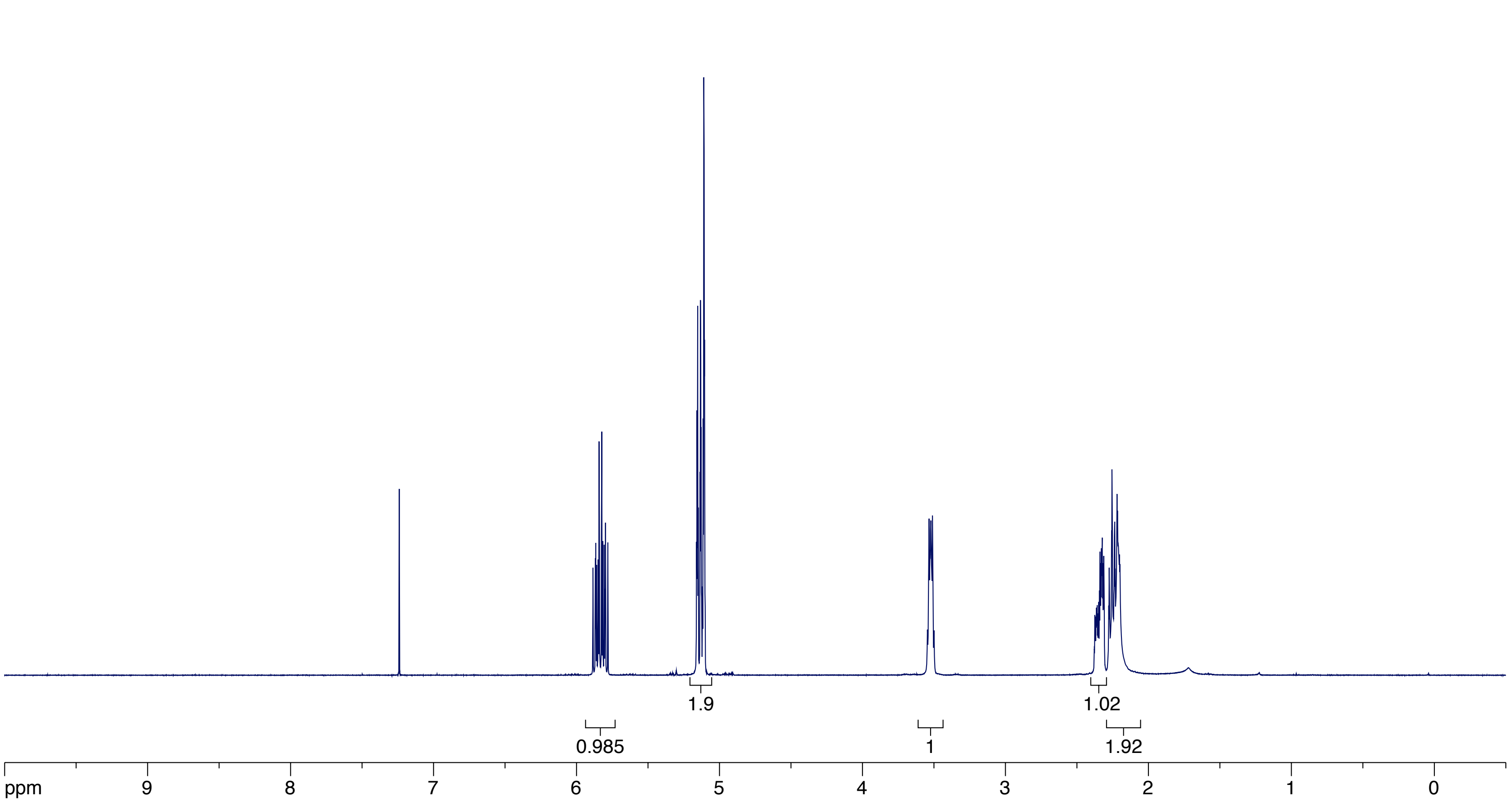

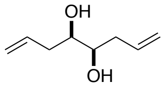


**S6** (100 MHz, $CDCl_3$)

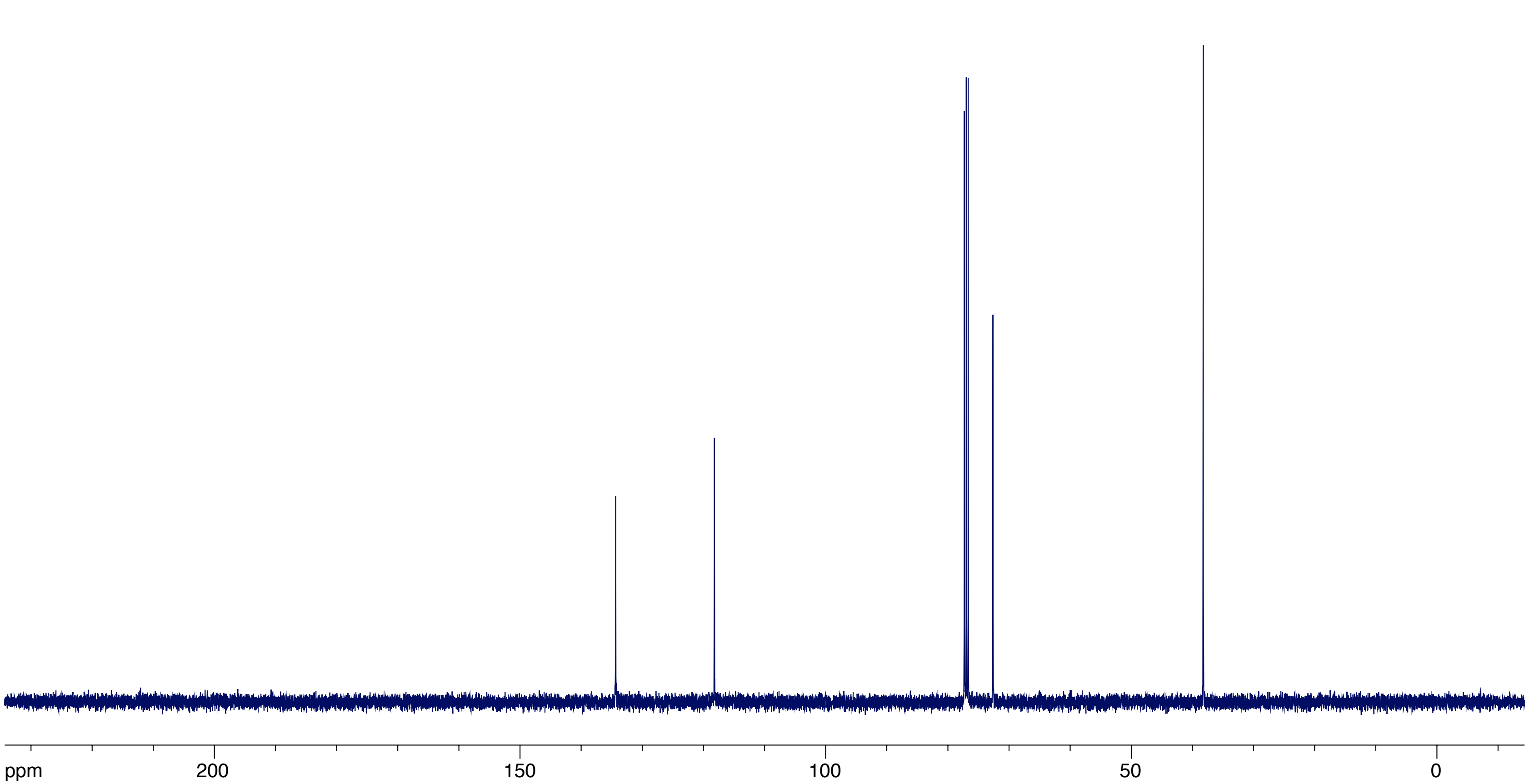

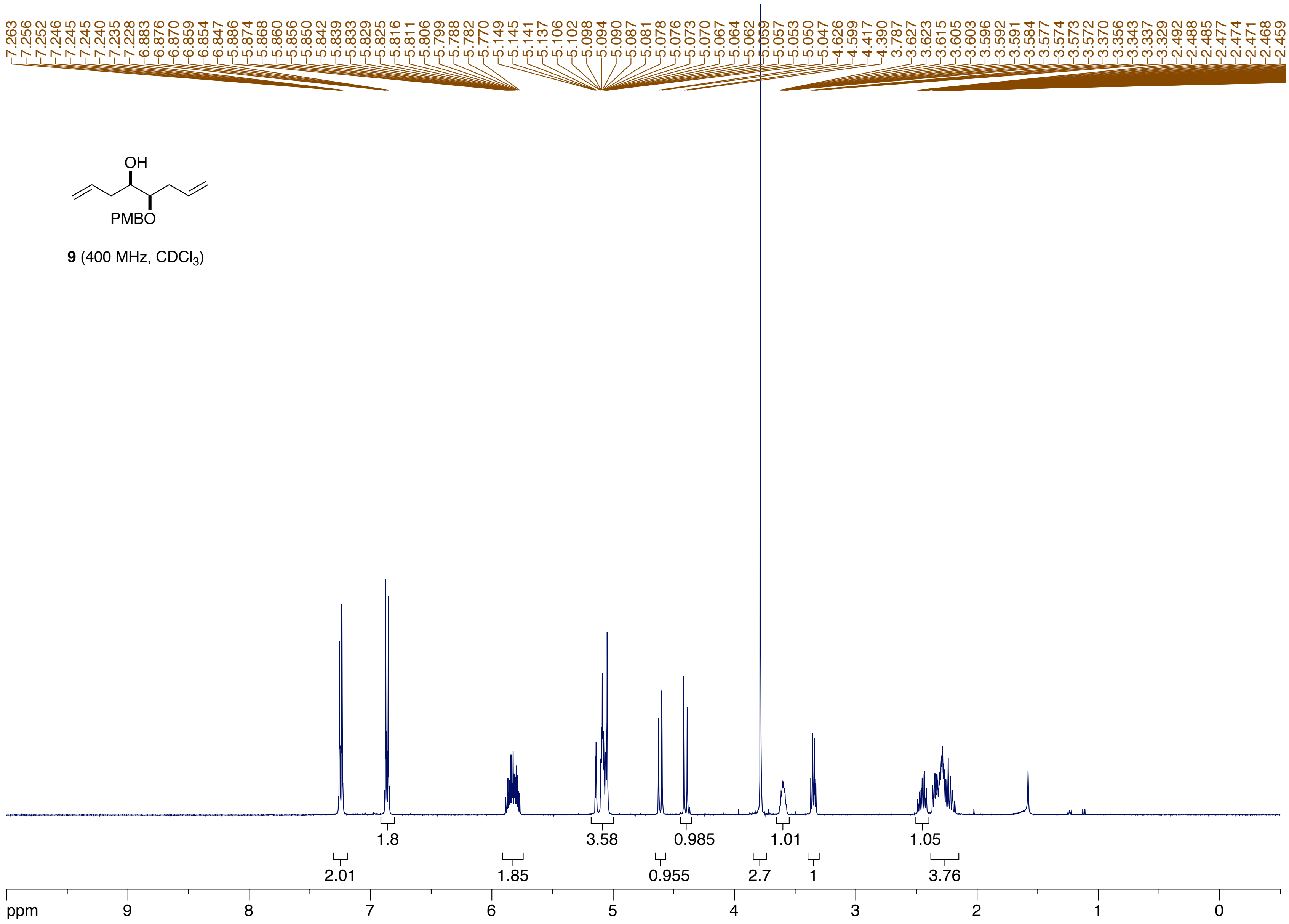
7.263 7.256 7.252 7.246 7.245 7.245 7.240 7.235 7.228 6.883 6.876 6.870 6.859 6.854 6.847 5.886 5.874 5.868 5.860 5.856 5.850 5.842 5.839 5.833 5.829 5.825 5.816 5.811 5.806 5.799 5.788 5.782 5.770 5.149 5.145 5.141 5.137 5.106 5.102 5.098 5.094 5.090 5.087 5.081 5.078 5.076 5.073 5.070 5.067 5.064 5.062 5.059 5.057 5.053 5.050 5.047 4.626 4.599 4.417 4.390 3.787 3.627 3.623 3.615 3.605 3.603 3.596 3.592 3.591 3.584 3.577 3.574 3.573 3.572 3.370 3.356 3.343 3.337 3.329 2.492 2.488 2.485 2.477 2.474 2.471 2.468 2.459
OH
PMBO
9 (400 MHz, $CDCl_3$)
1.8
3.58
0.985
1.01
1.05
2.01
1.85
0.955
2.7
1
3.76
ppm
9
8
7
6
5
4
3
2
1
0

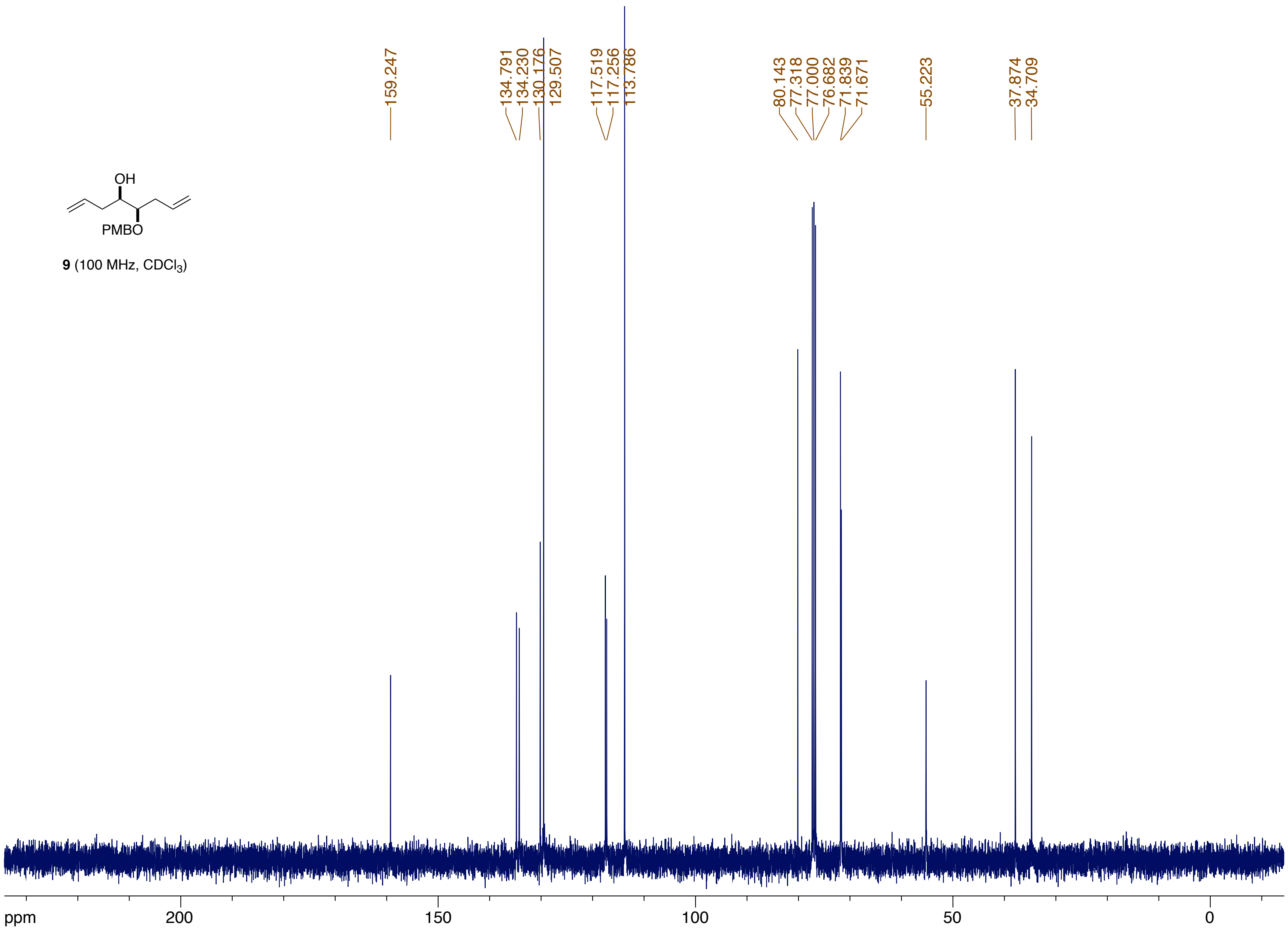


**9** (100 MHz, $CDCl_3$)

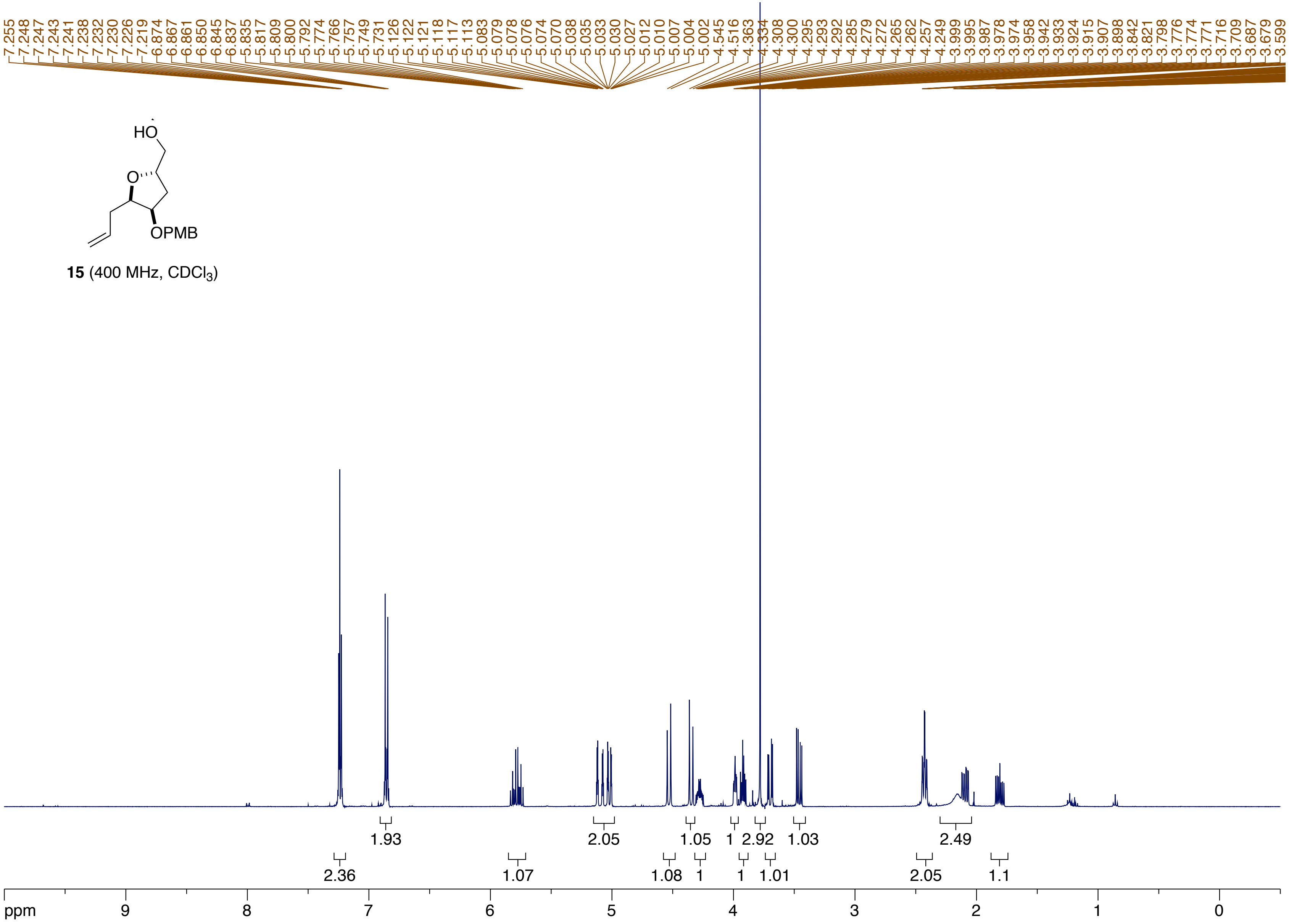


**15** (400 MHz, $CDCl_3$)

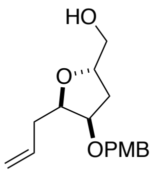


**15** (100 MHz, $CDCl_3$)

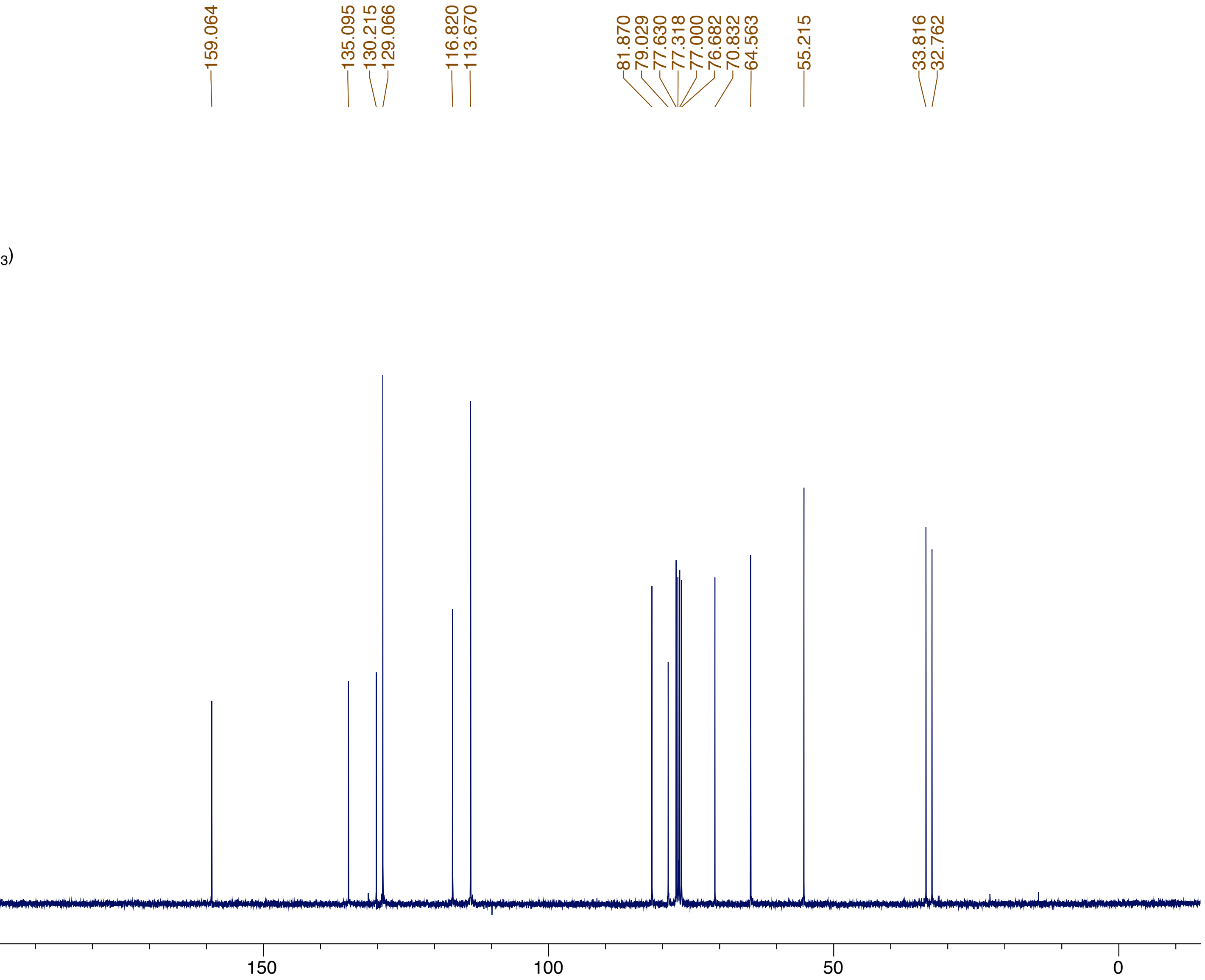

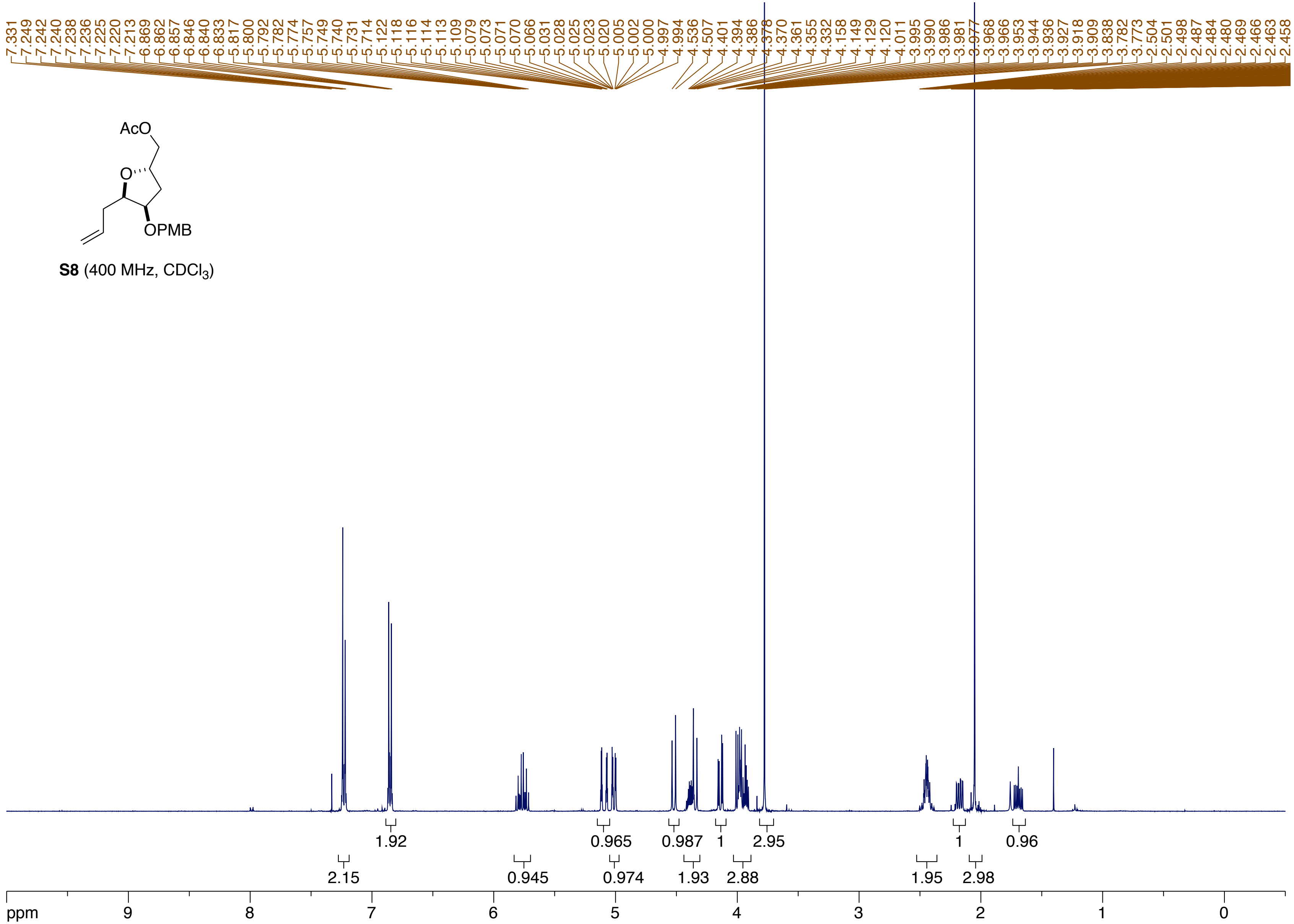
7.331 7.249 7.242 7.240 7.238 7.236 7.225 7.220 7.213 6.869 6.862 6.857 6.846 6.840 6.833 5.817 5.800 5.792 5.782 5.774 5.757 5.749 5.740 5.731 5.714 5.122 5.118 5.116 5.114 5.113 5.109 5.079 5.073 5.071 5.070 5.066 5.031 5.028 5.025 5.023 5.020 5.005 5.002 5.000 4.997 4.994 4.536 4.507 4.401 4.394 4.386 4.378 4.370 4.361 4.355 4.332 4.158 4.149 4.129 4.120 4.011 3.995 3.990 3.986 3.981 3.977 3.968 3.966 3.953 3.944 3.936 3.927 3.918 3.909 3.838 3.782 3.773 2.504 2.501 2.498 2.487 2.484 2.480 2.469 2.466 2.463 2.458
AcO
O
OPMB
S8 (400 MHz, CDCl3)
2.15
1.92
0.945
0.965
0.974
0.987
1.93
1
2.88
2.95
1.95
1
2.98
0.96
ppm 9 8 7 6 5 4 3 2 1 0

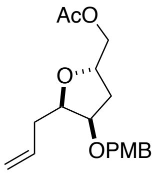


**S8** (100 MHz, $CDCl_3$)

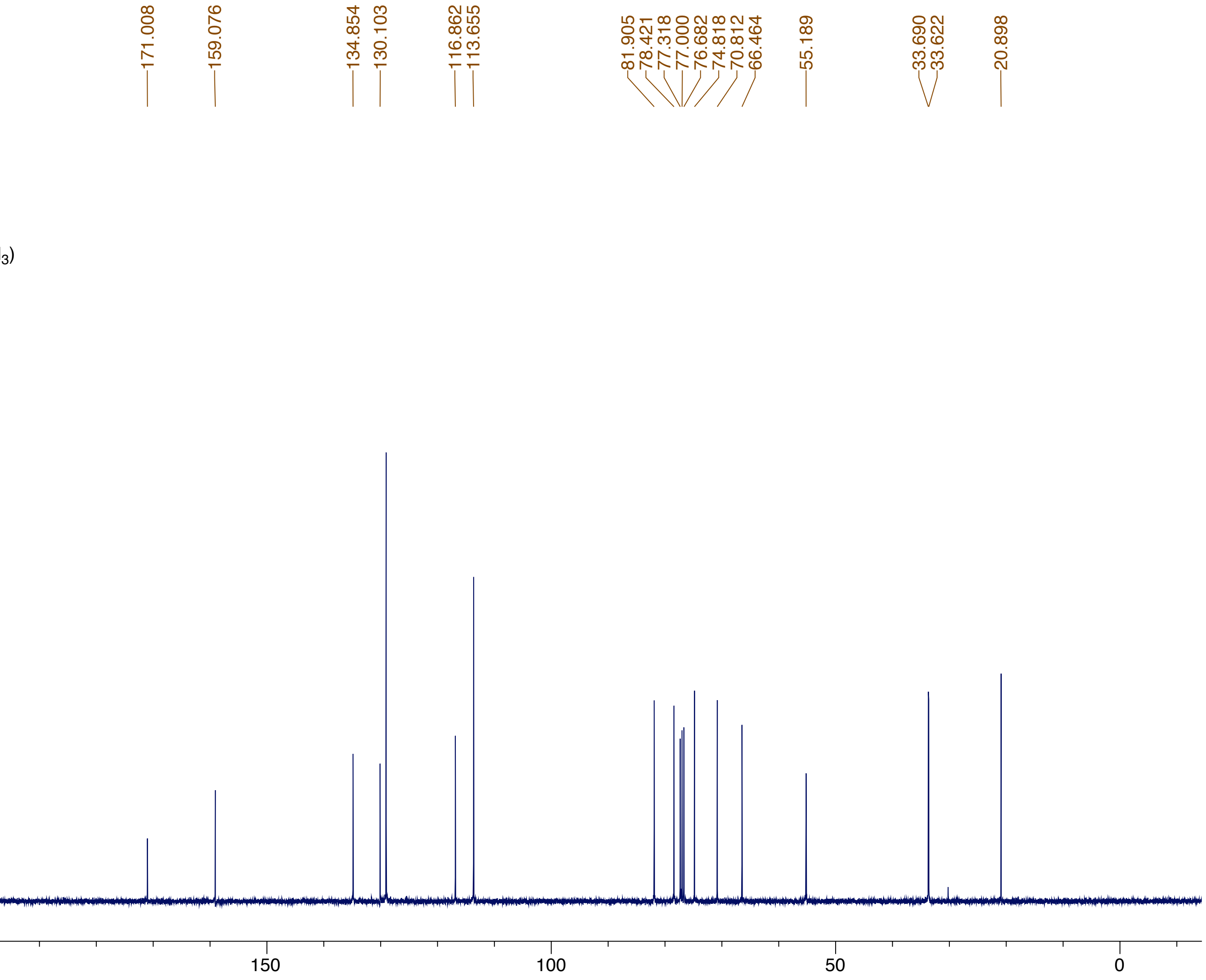

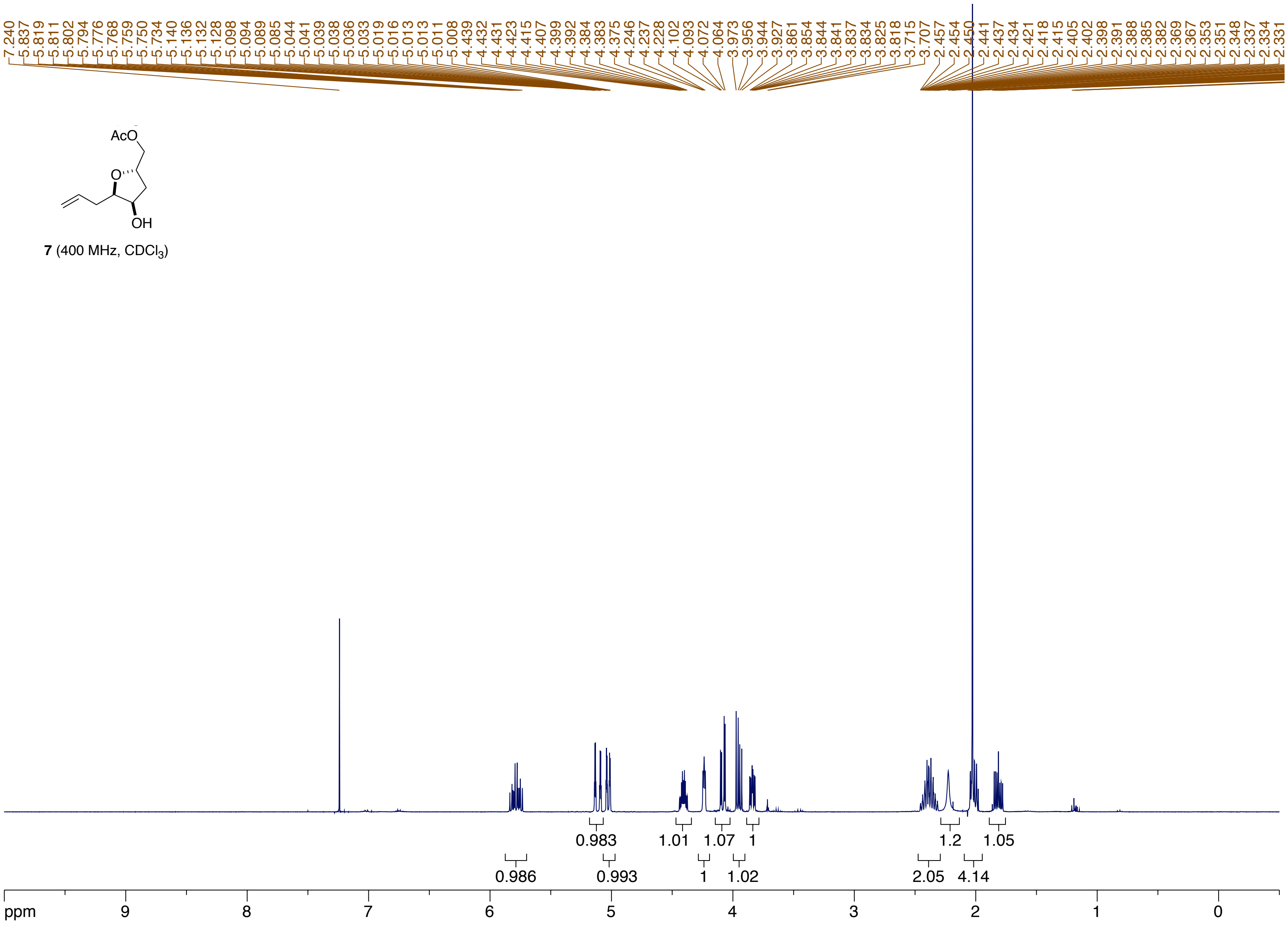


**7** (400 MHz, $CDCl_3$)

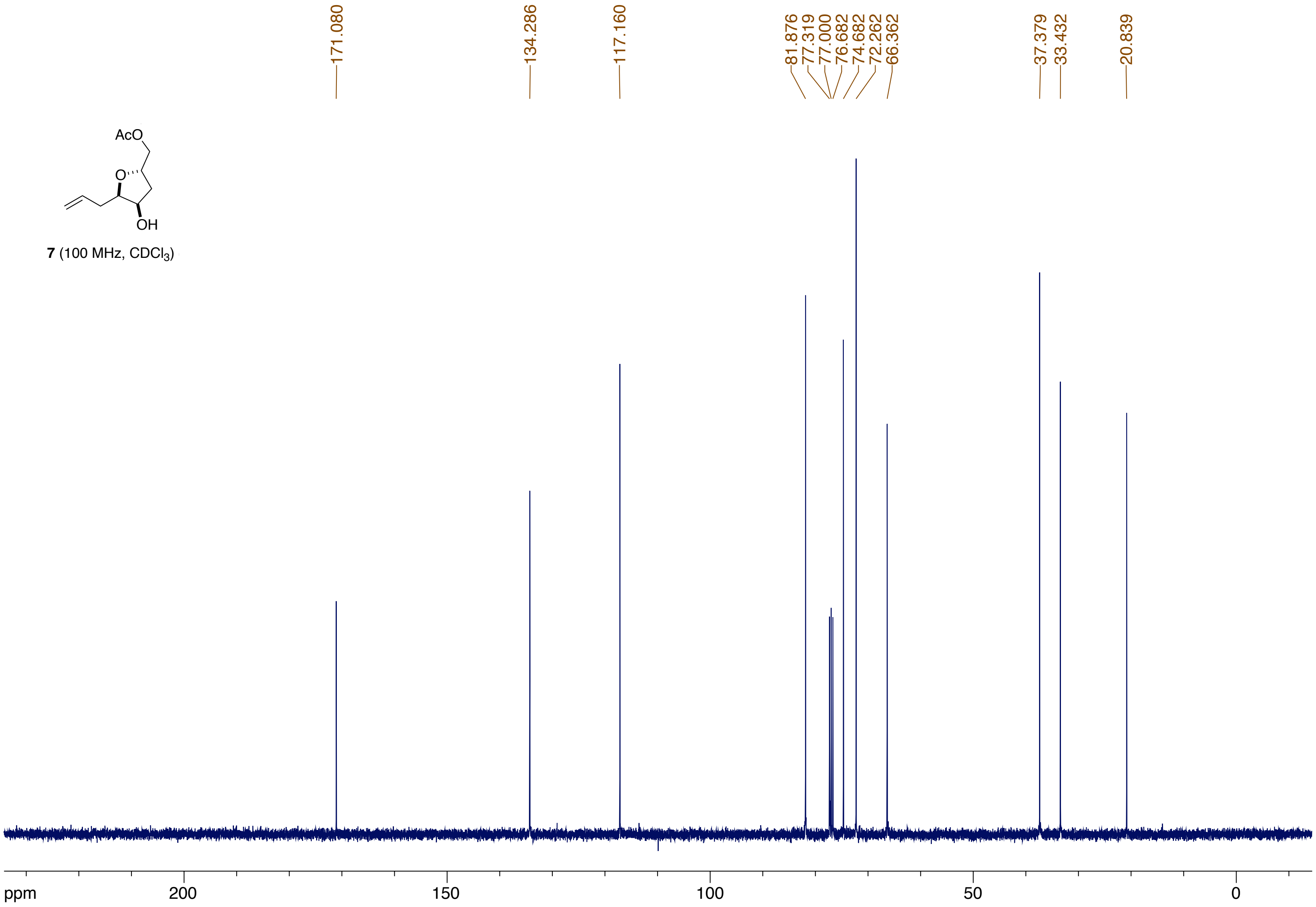


**7** (100 MHz, $CDCl_3$)

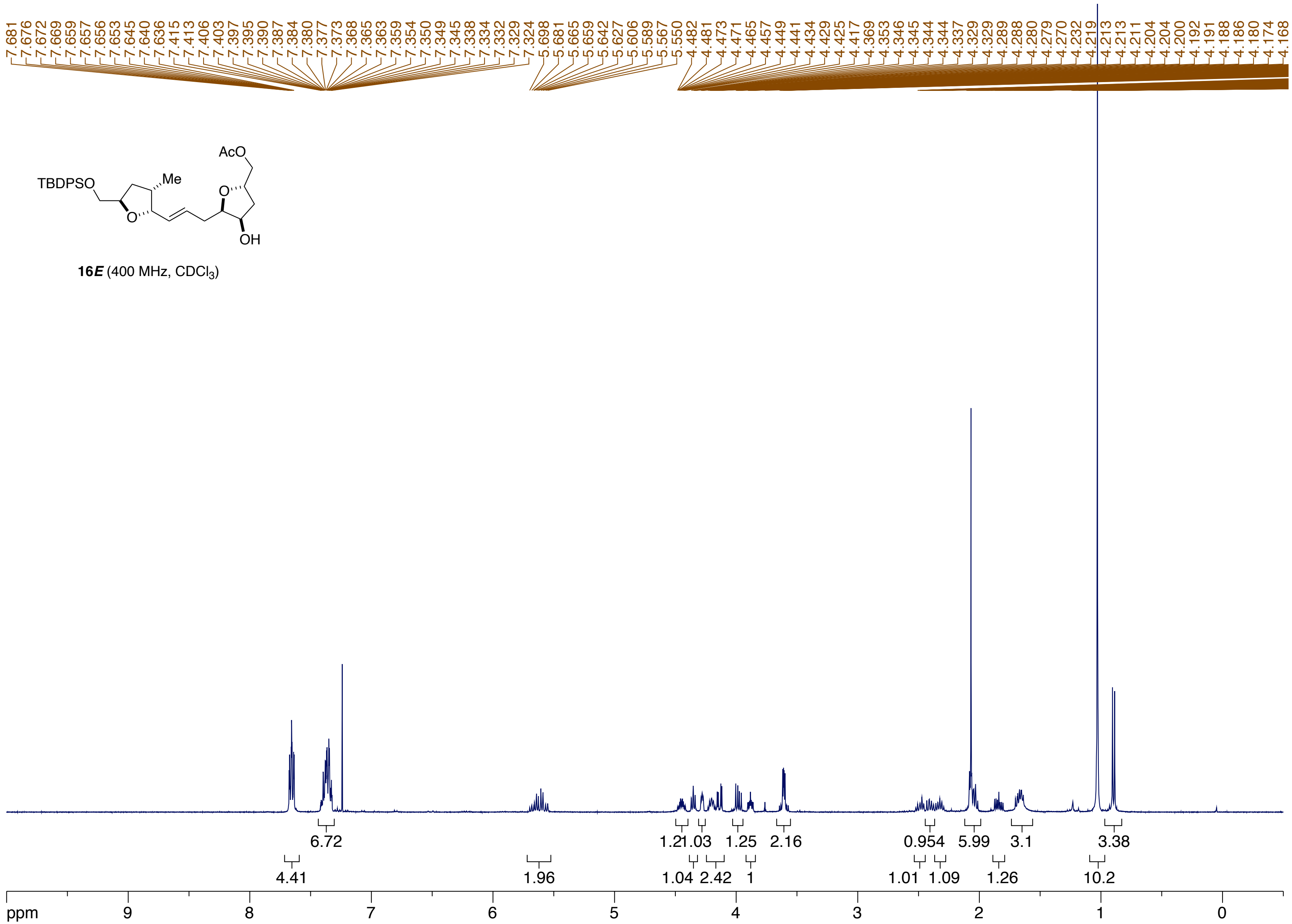
16*E* (400 MHz, CDCl3)
ppm

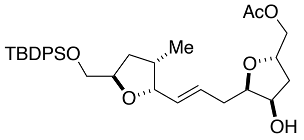

**16*E*** (100 MHz, $CDCl_3$)

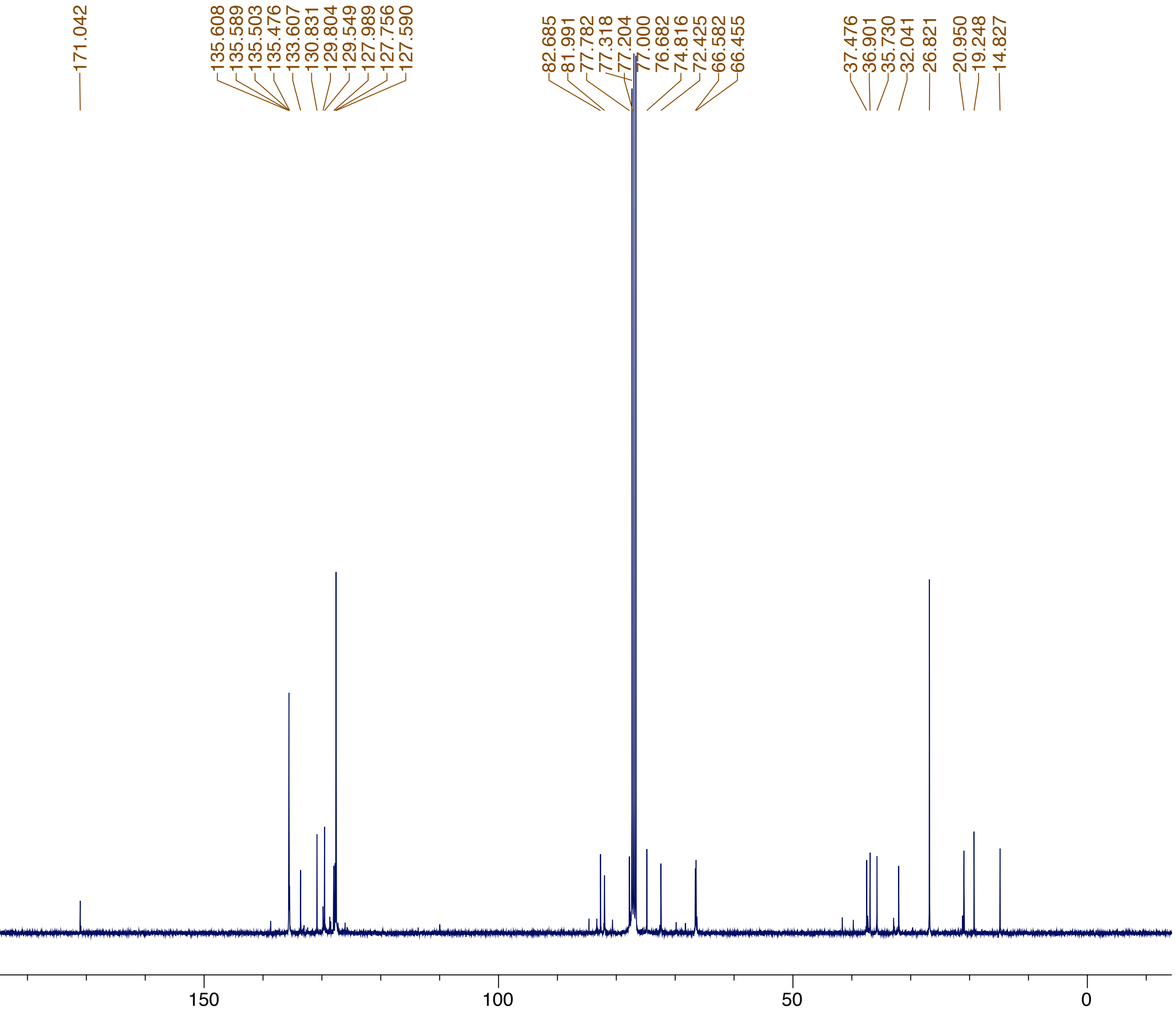

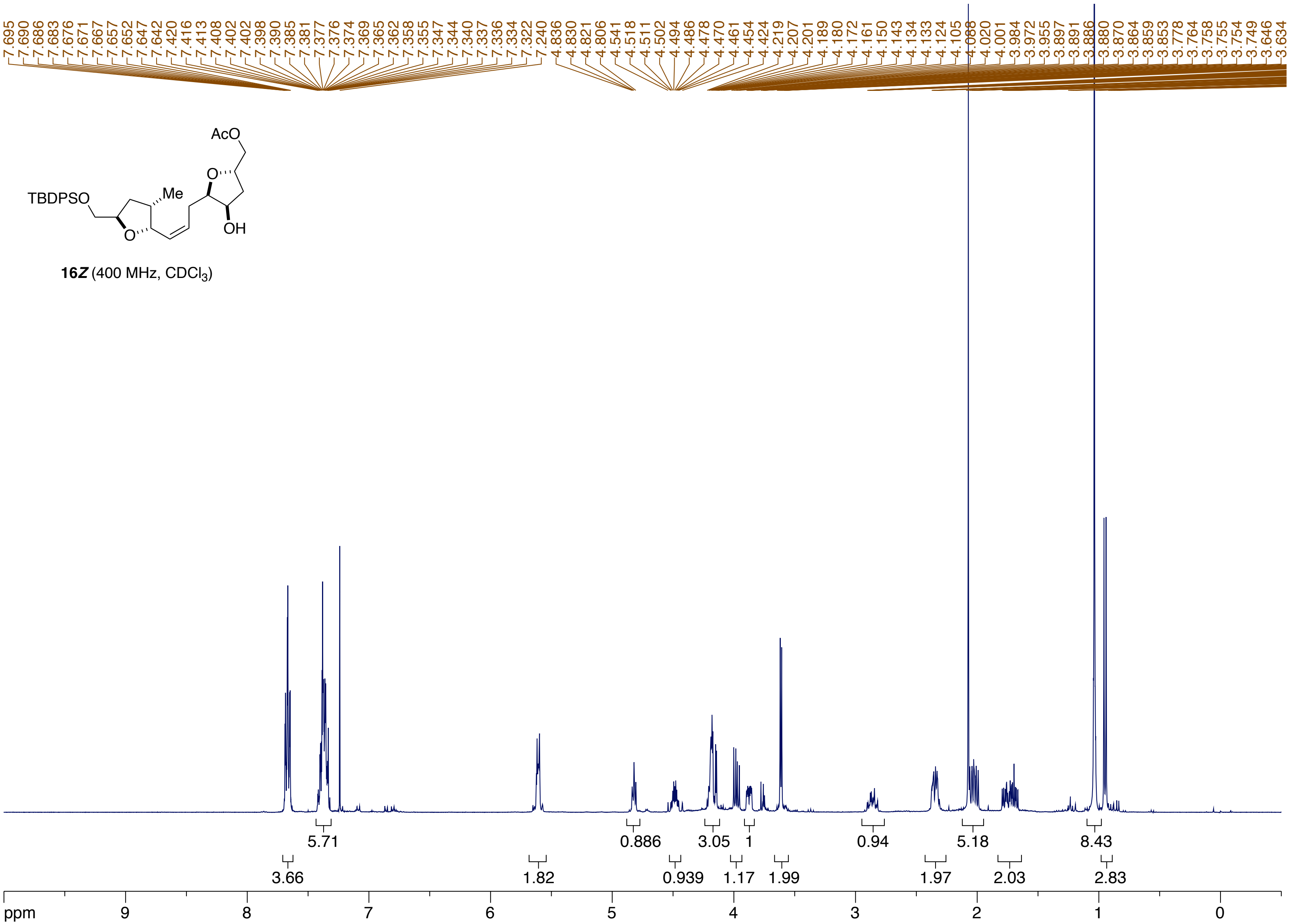


**16*Z*** (400 MHz, $CDCl_3$)

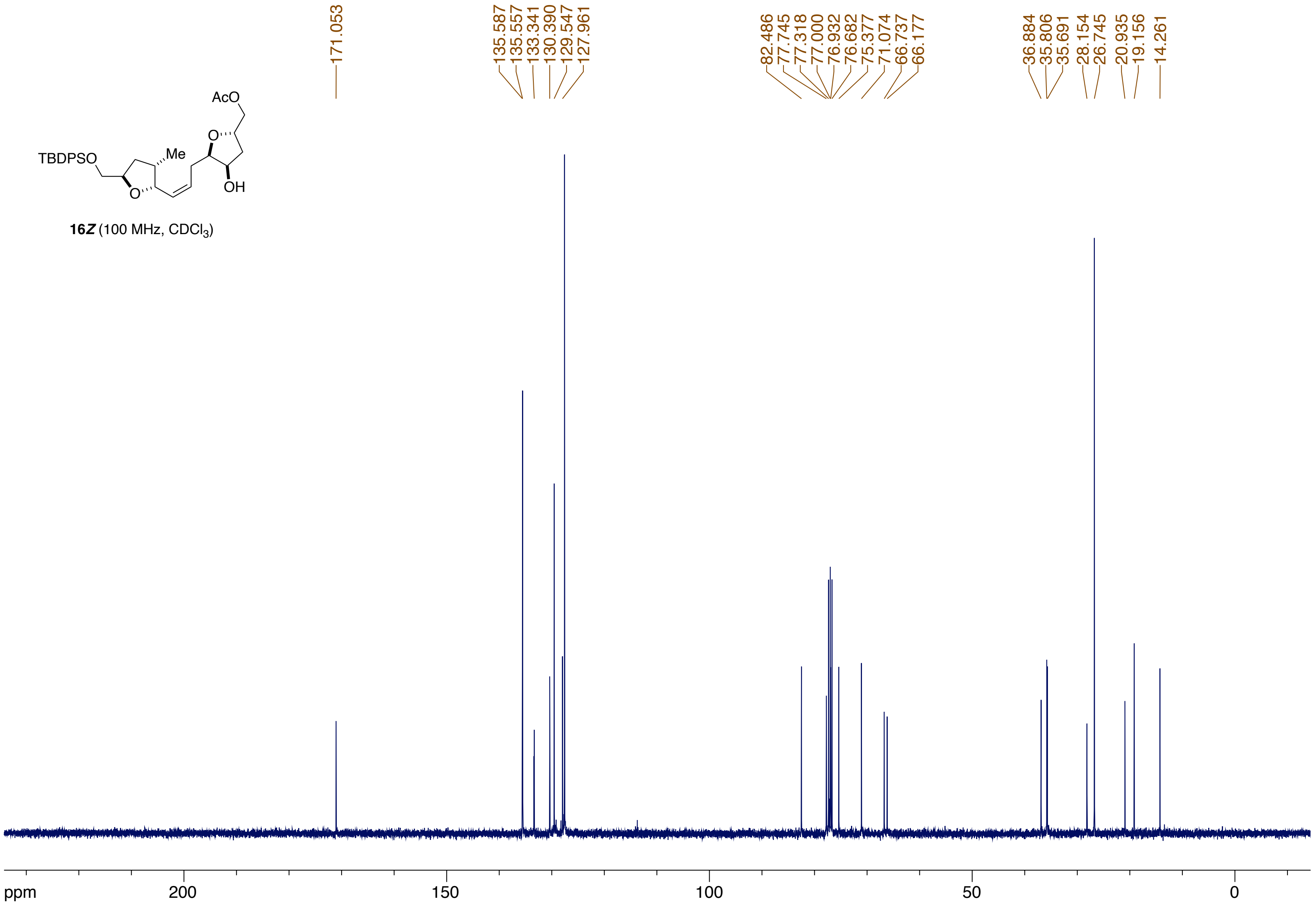
171.053
135.587
135.557
133.341
130.390
129.547
127.961
82.486
77.745
77.318
77.000
76.932
76.682
75.377
71.074
66.737
66.177
36.884
35.806
35.691
28.154
26.745
20.935
19.156
14.261
AcO
TBDPSO
Me
O
OH
**16*Z*** (100 MHz, $CDCl_3$)
ppm
200
150
100
50
0

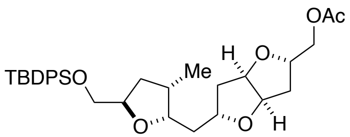


**17** (400 MHz, $C_6D_6$)

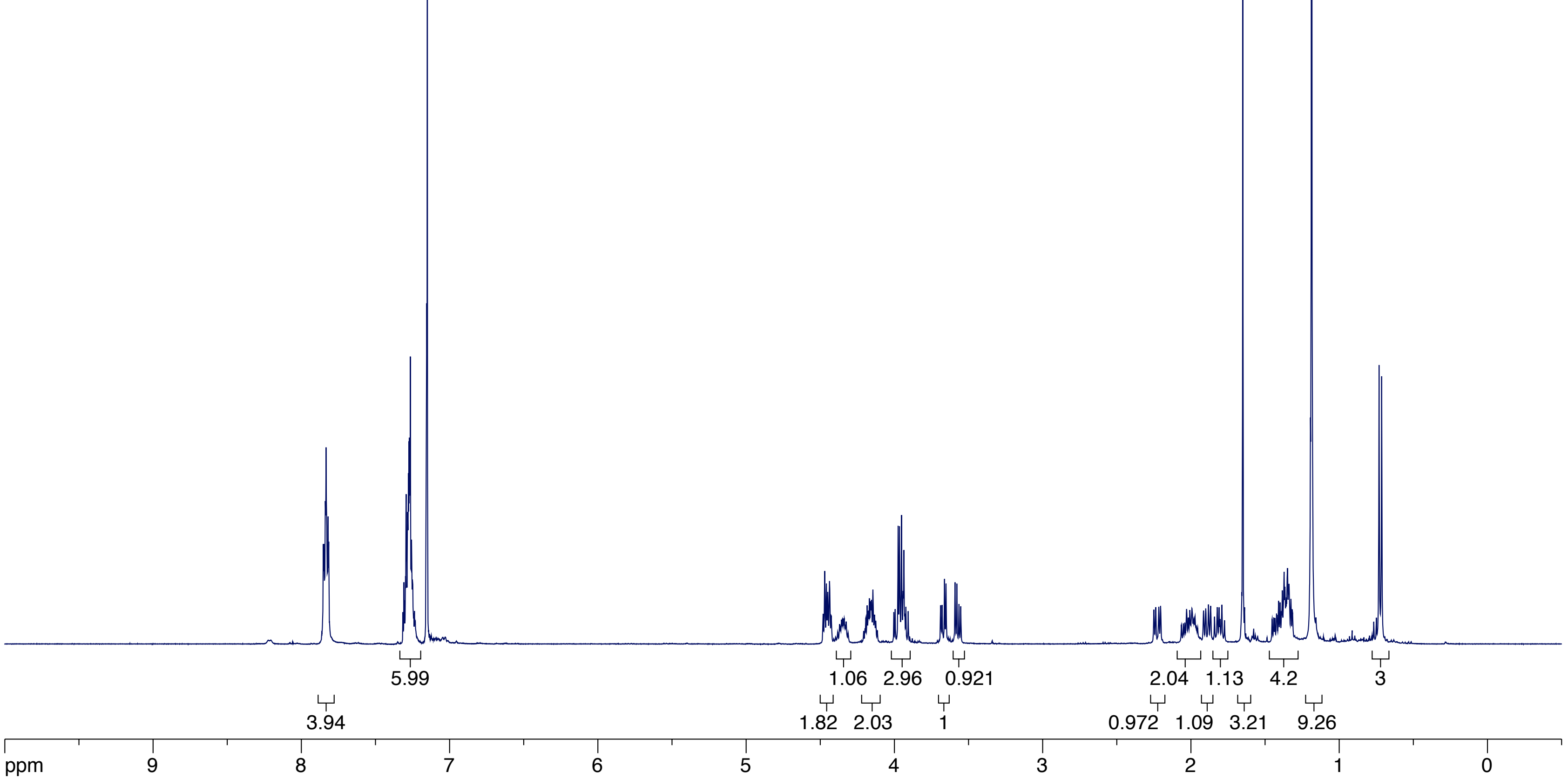

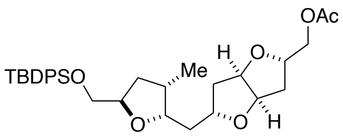


**17** (100 MHz, $C_6D_6$)

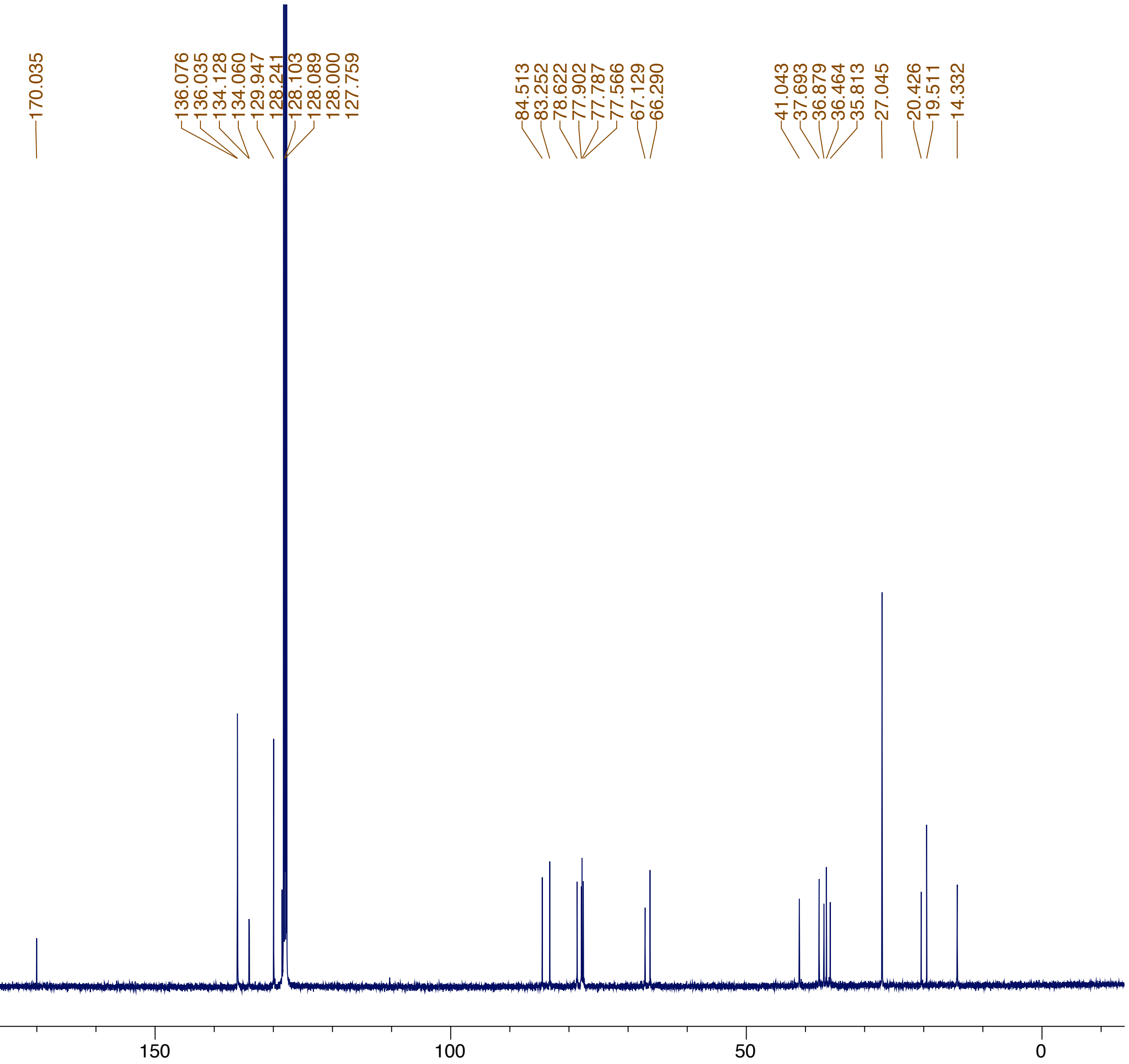

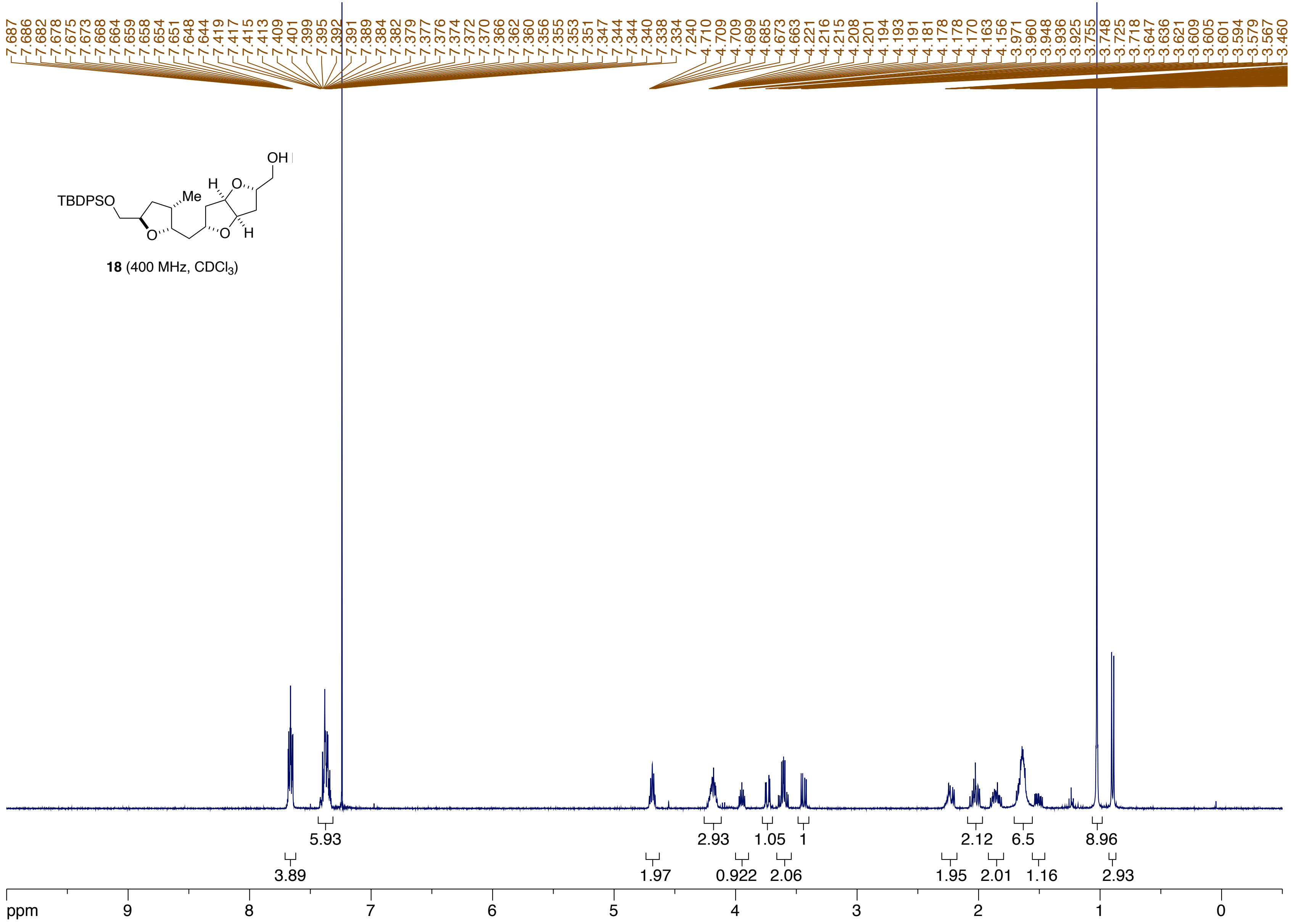
7.687 7.686 7.682 7.678 7.675 7.673 7.668 7.664 7.659 7.658 7.654 7.651 7.648 7.644 7.419 7.417 7.415 7.413 7.409 7.401 7.399 7.395 7.392 7.391 7.389 7.384 7.382 7.379 7.377 7.376 7.374 7.372 7.370 7.366 7.362 7.360 7.356 7.355 7.353 7.351 7.347 7.344 7.340 7.338 7.334 7.240 4.710 4.709 4.699 4.685 4.673 4.663 4.221 4.216 4.215 4.208 4.201 4.194 4.193 4.191 4.181 4.178 4.178 4.170 4.163 4.156 3.971 3.960 3.948 3.936 3.925 3.755 3.748 3.725 3.718 3.647 3.636 3.621 3.609 3.605 3.601 3.594 3.579 3.567 3.460
TBDPSO
Me
H
O
OH
O
O
H
18 (400 MHz, CDCl3)
5.93
3.89
1.97
2.93
0.922
1.05
2.06
1
1.95
2.12
2.01
6.5
1.16
8.96
2.93
ppm
9
8
7
6
5
4
3
2
1
0

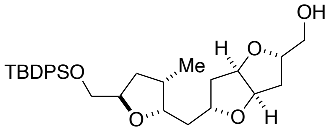


**18** (100 MHz, $CDCl_3$)

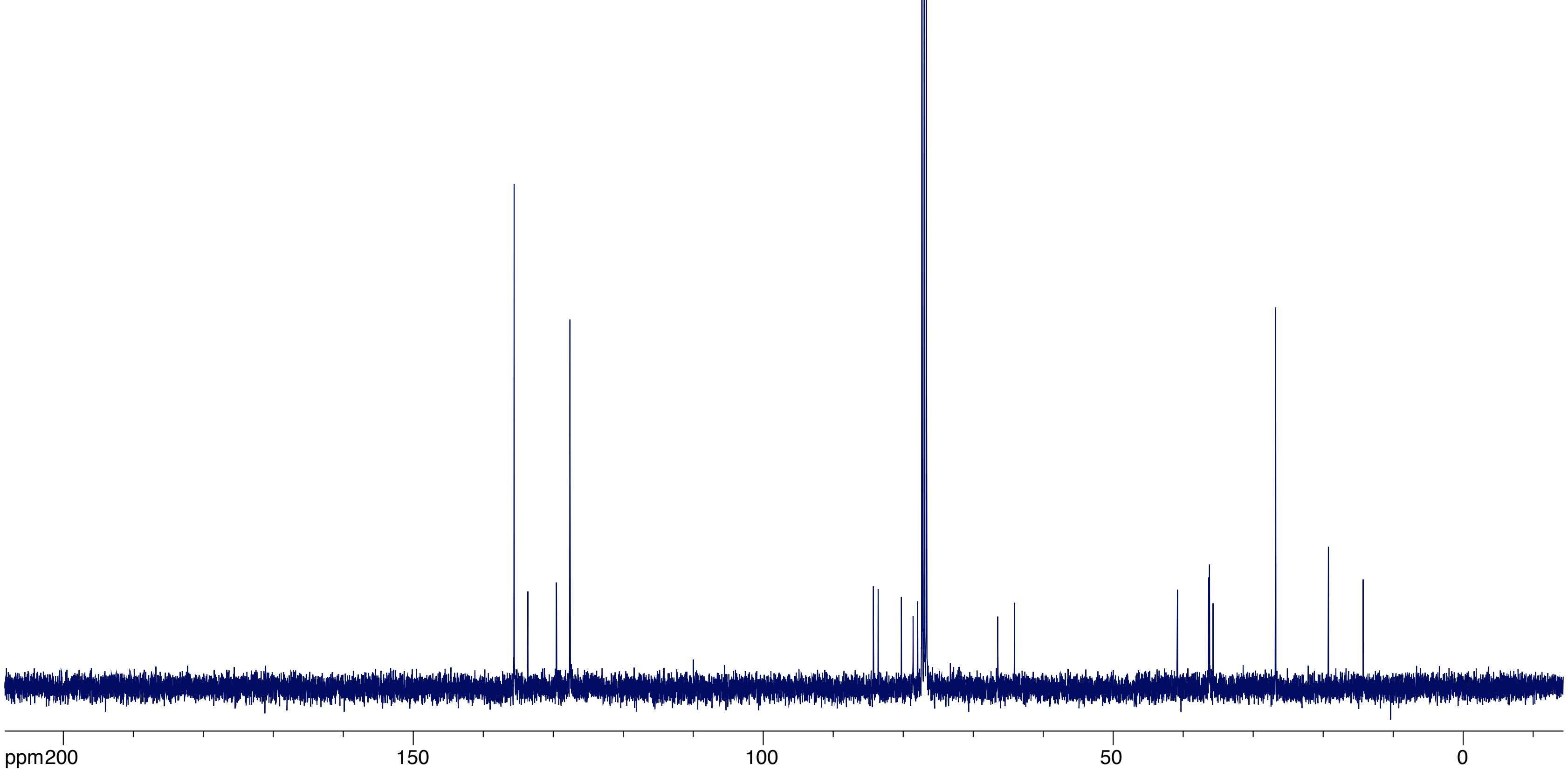

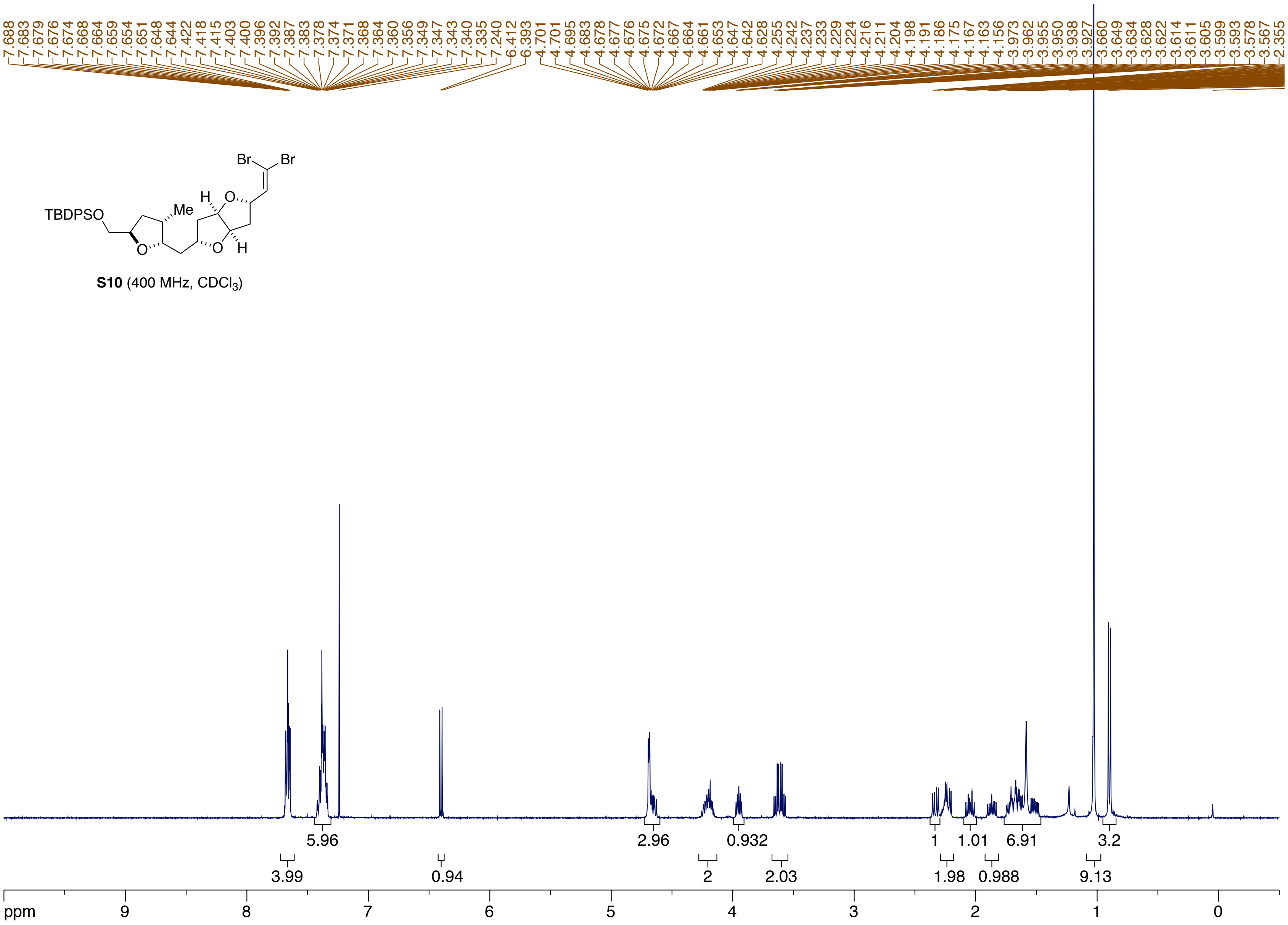

**S10** (400 MHz, $CDCl_3$)

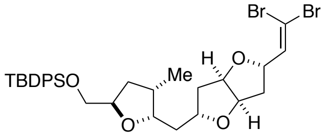


**S10** (100 MHz, $CDCl_3$)

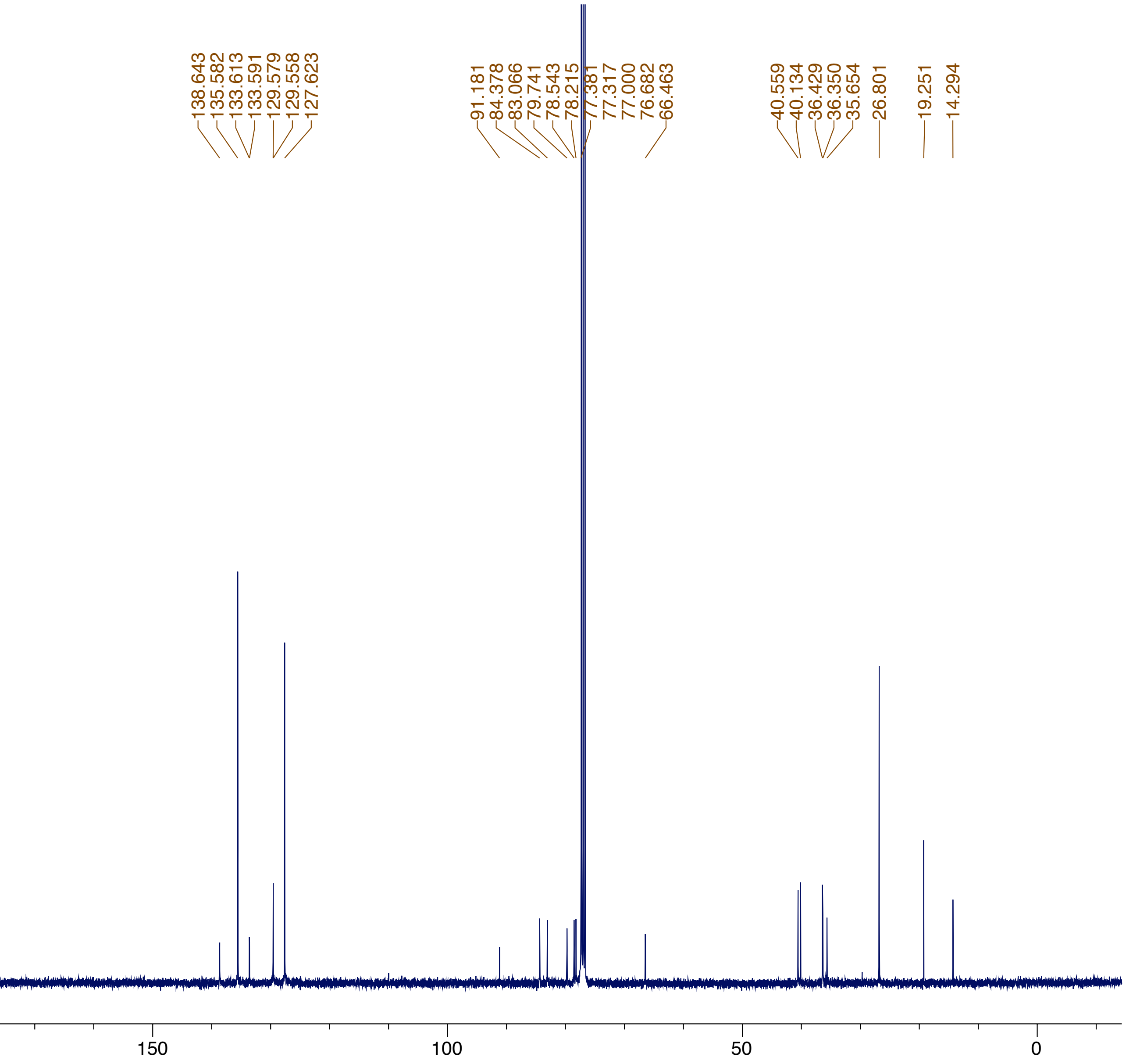

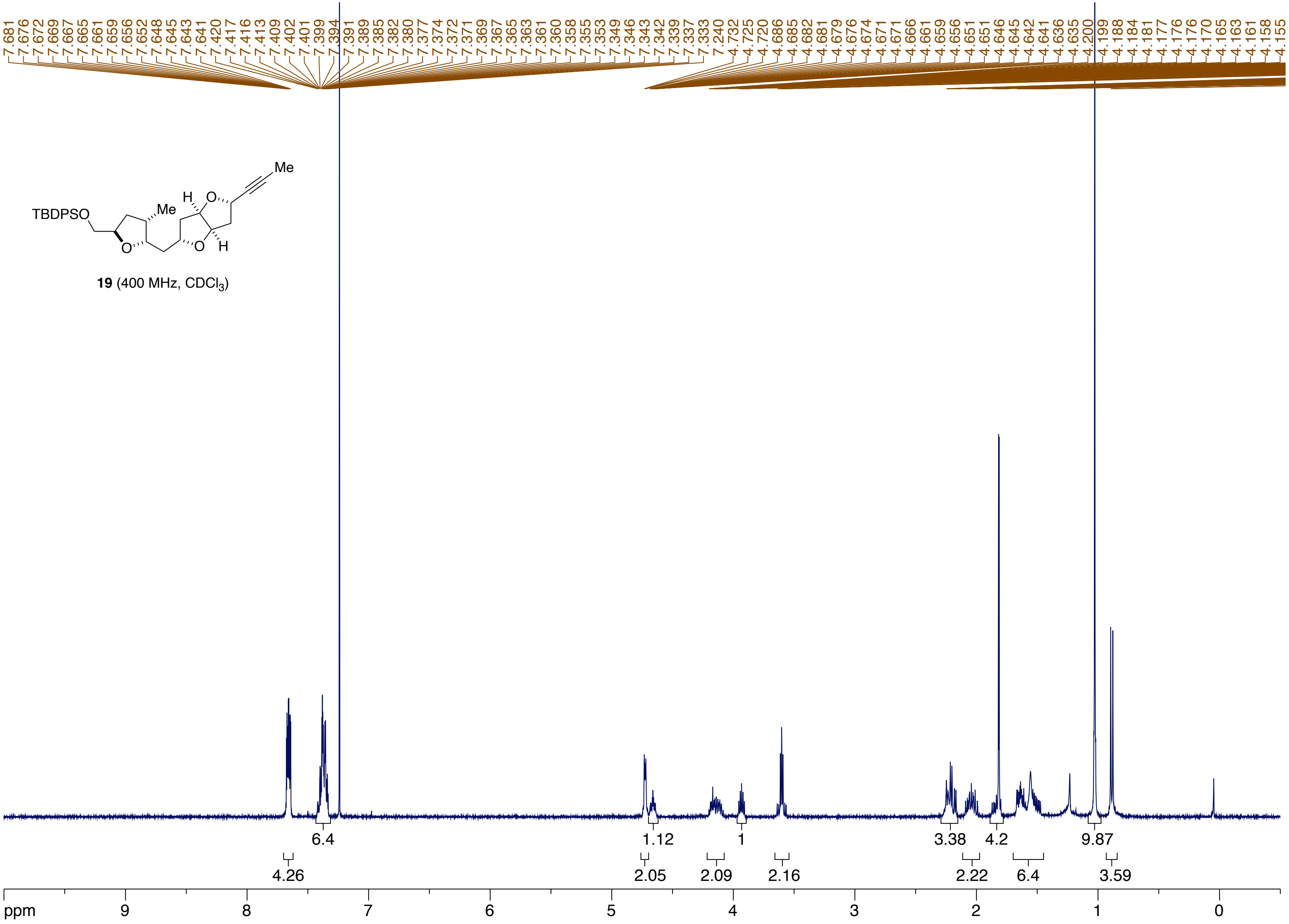
7.681 7.676 7.672 7.669 7.667 7.665 7.661 7.659 7.656 7.652 7.648 7.645 7.643 7.641 7.420 7.417 7.416 7.413 7.409 7.402 7.401 7.399 7.394 7.391 7.389 7.385 7.382 7.380 7.377 7.374 7.372 7.371 7.369 7.367 7.365 7.363 7.361 7.360 7.358 7.355 7.353 7.349 7.346 7.343 7.342 7.339 7.337 7.333 7.240 4.732 4.725 4.720 4.686 4.685 4.682 4.681 4.679 4.676 4.674 4.671 4.666 4.661 4.659 4.656 4.651 4.651 4.646 4.645 4.642 4.641 4.636 4.635 4.200 4.199 4.188 4.184 4.181 4.177 4.176 4.176 4.170 4.165 4.163 4.161 4.158 4.155
TBDPSO
Me
H
O
H
Me
**19** (400 MHz, $CDCl_3$)
6.4
1.12
1
3.38
4.2
9.87
4.26
2.05
2.09
2.16
2.22
6.4
3.59
ppm
9
8
7
6
5
4
3
2
1
0

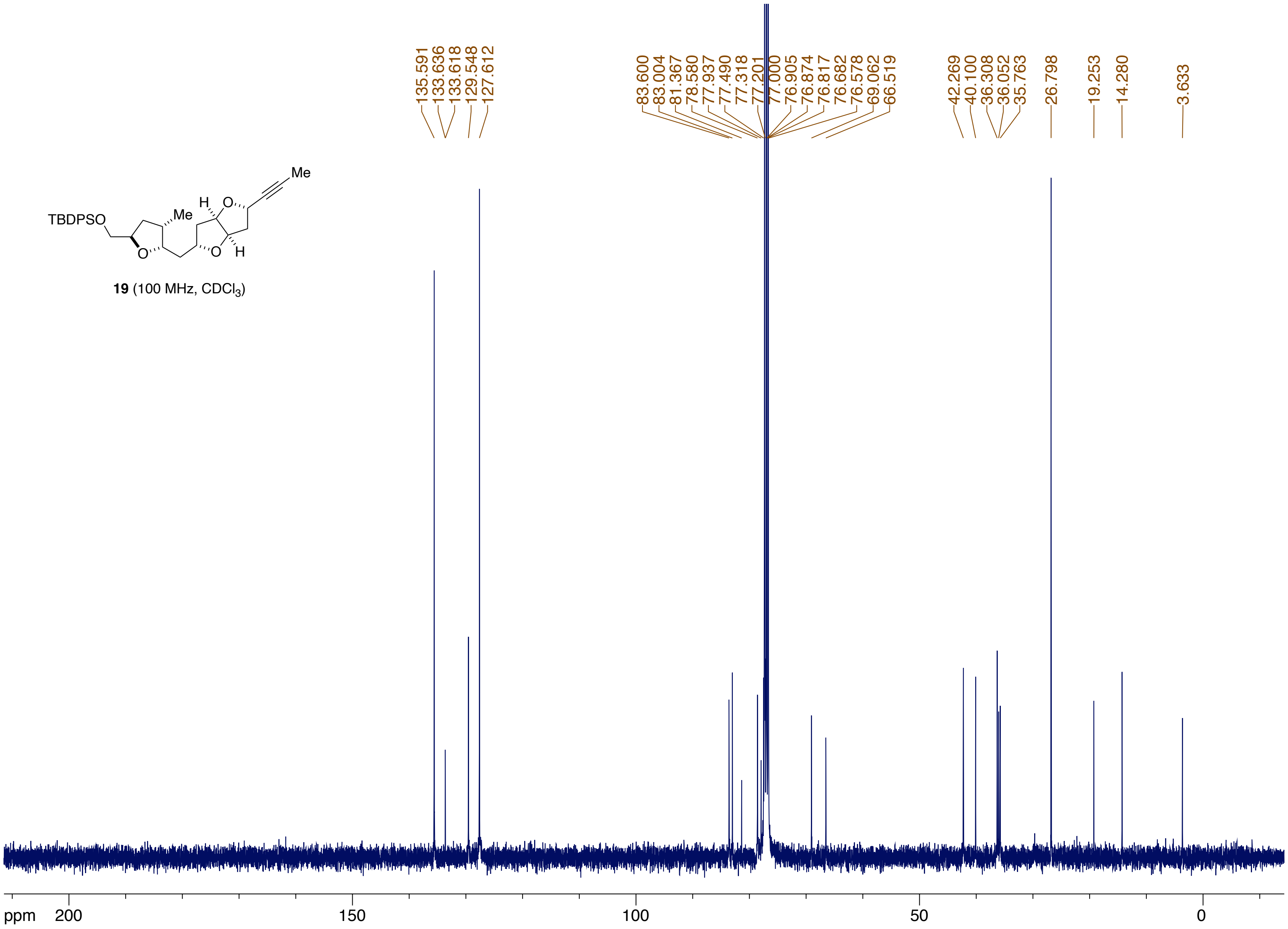


**19** (100 MHz, $CDCl_3$)

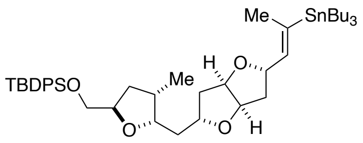


**4** (400 MHz, $C_6D_6$)

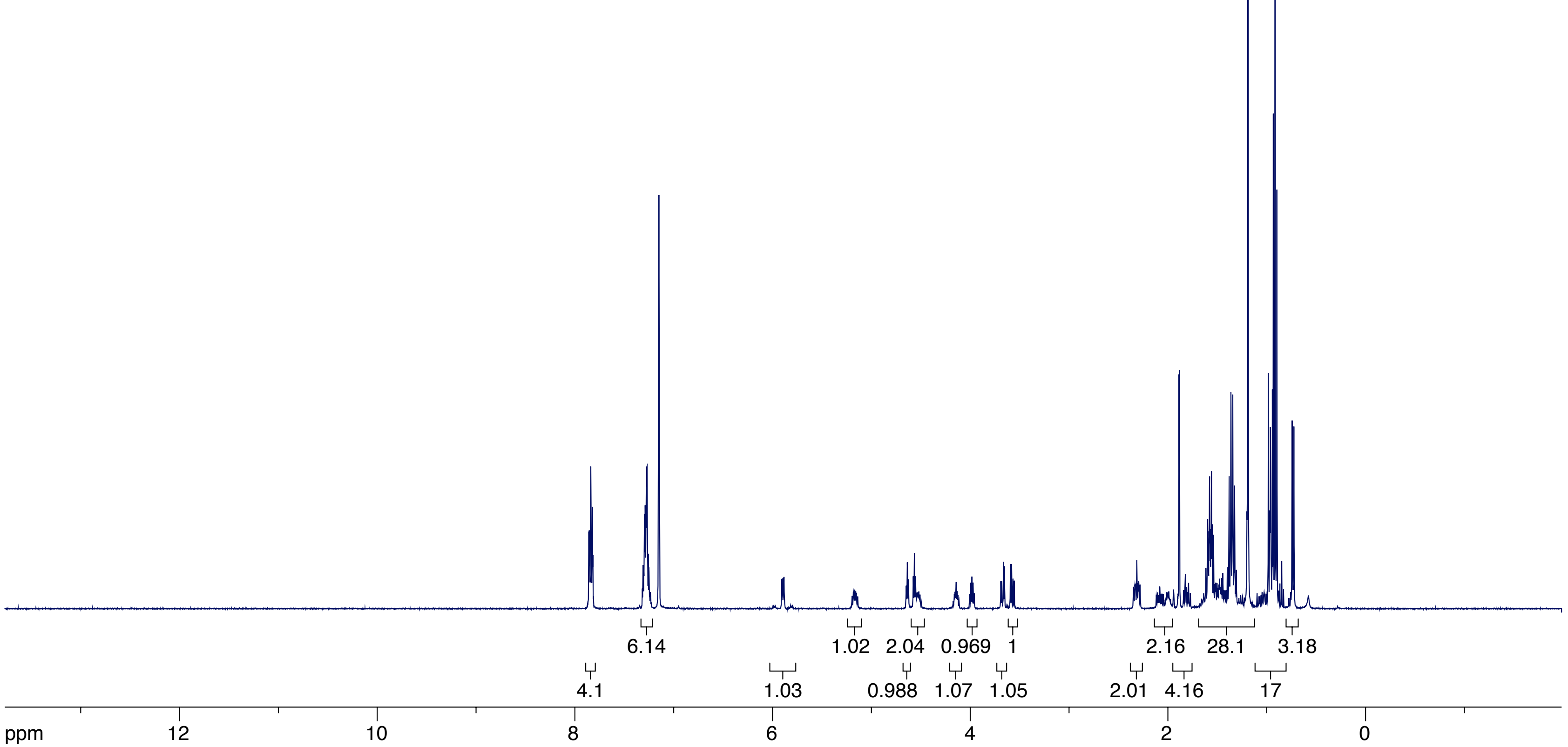

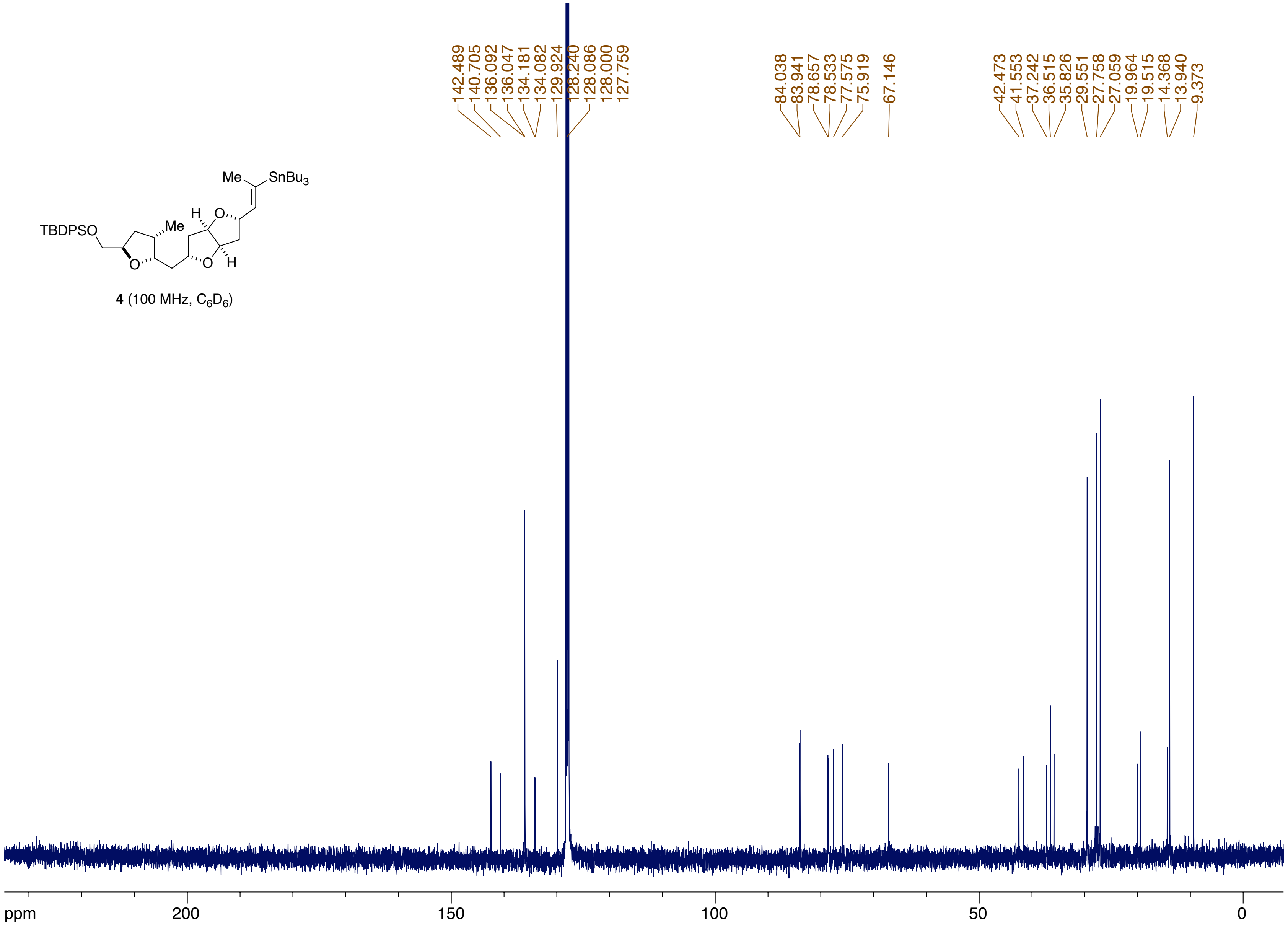


**4** (100 MHz, $C_6D_6$)

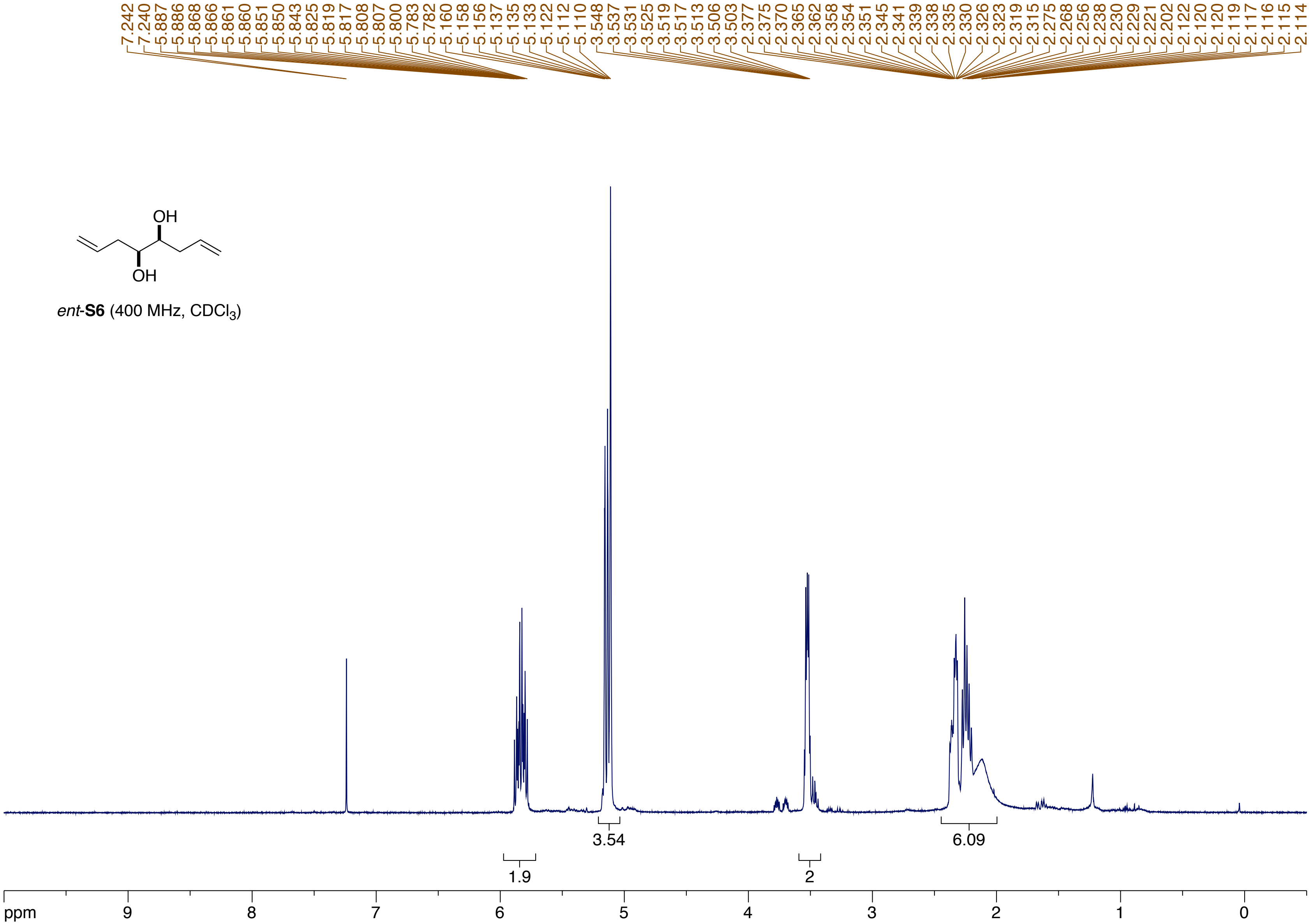

7.242
7.240
5.887
5.886
5.868
5.866
5.861
5.860
5.851
5.850
5.843
5.825
5.819
5.817
5.808
5.807
5.800
5.783
5.782
5.160
5.158
5.156
5.137
5.135
5.133
5.122
5.112
5.110
3.548
3.537
3.531
3.525
3.519
3.517
3.513
3.506
3.503
2.377
2.375
2.370
2.365
2.362
2.358
2.354
2.351
2.345
2.341
2.339
2.338
2.335
2.330
2.326
2.323
2.319
2.315
2.275
2.268
2.256
2.238
2.230
2.229
2.221
2.202
2.122
2.120
2.119
2.117
2.116
2.115
2.114
OH
OH
*ent*-**S6** (400 MHz, $CDCl_3$)
3.54
6.09
1.9
2
ppm
9
8
7
6
5
4
3
2
1
0

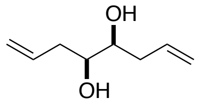


*ent*-**S6** (100 MHz, $CDCl_3$)

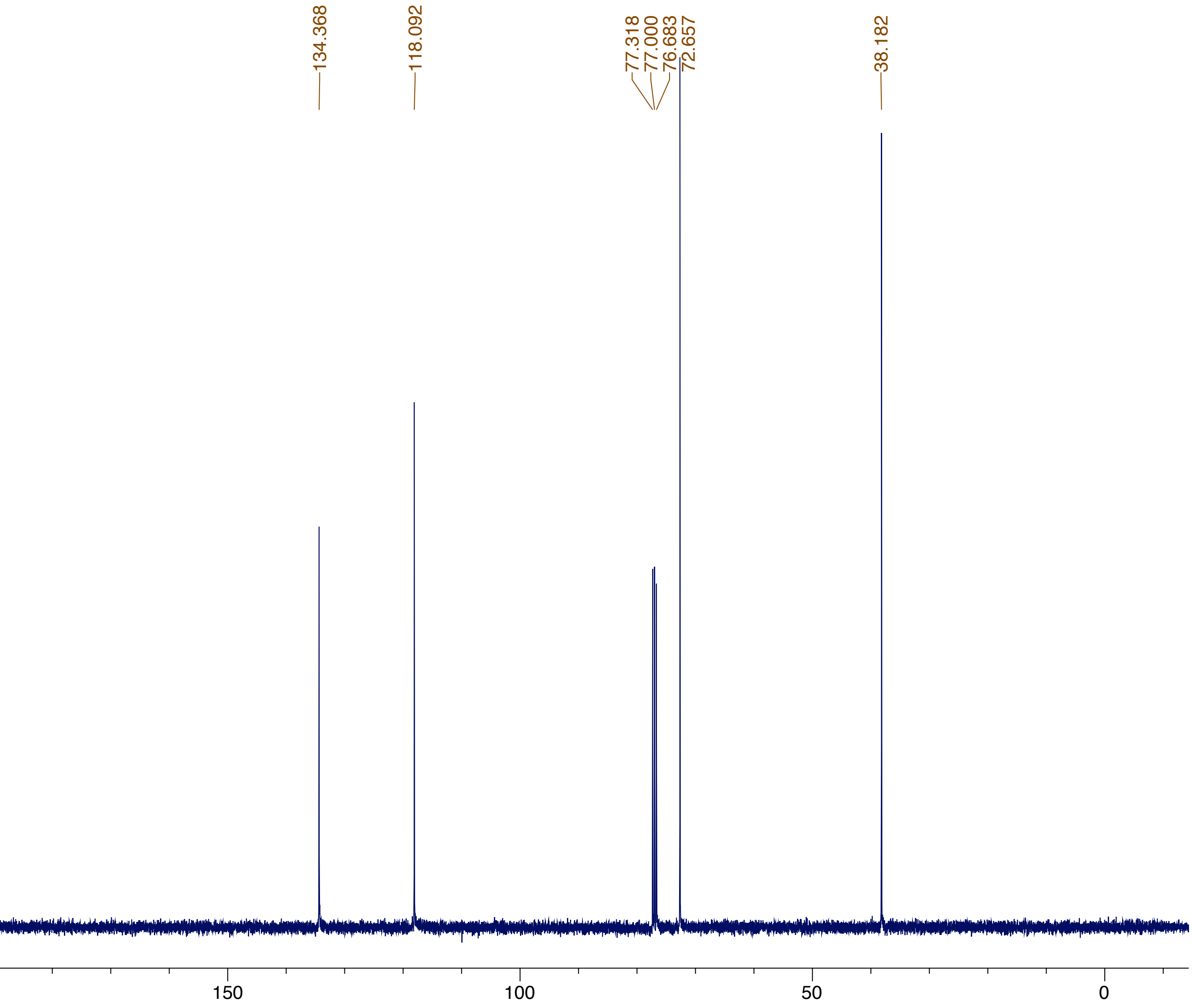

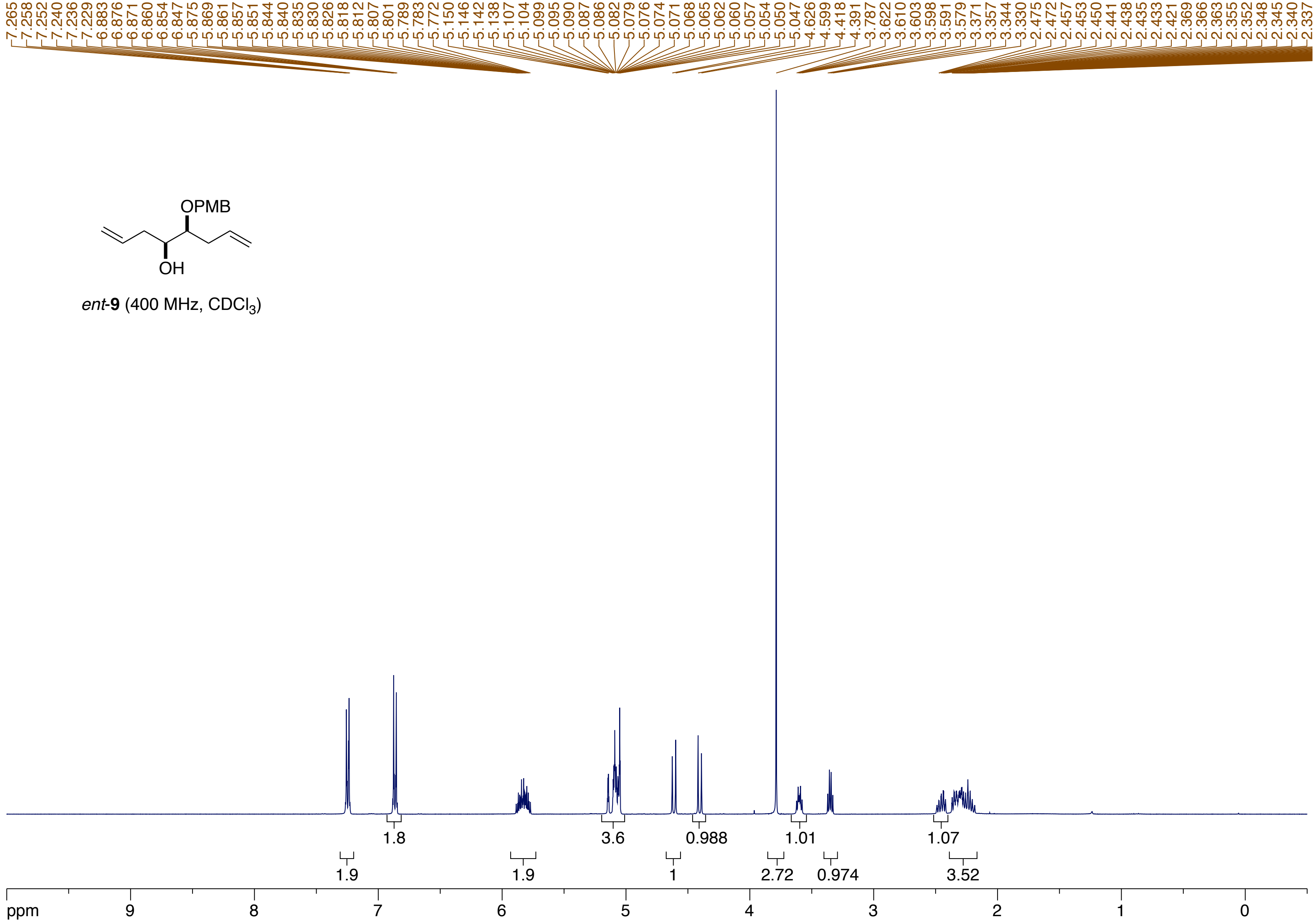


*ent*-**9** (400 MHz, $CDCl_3$)

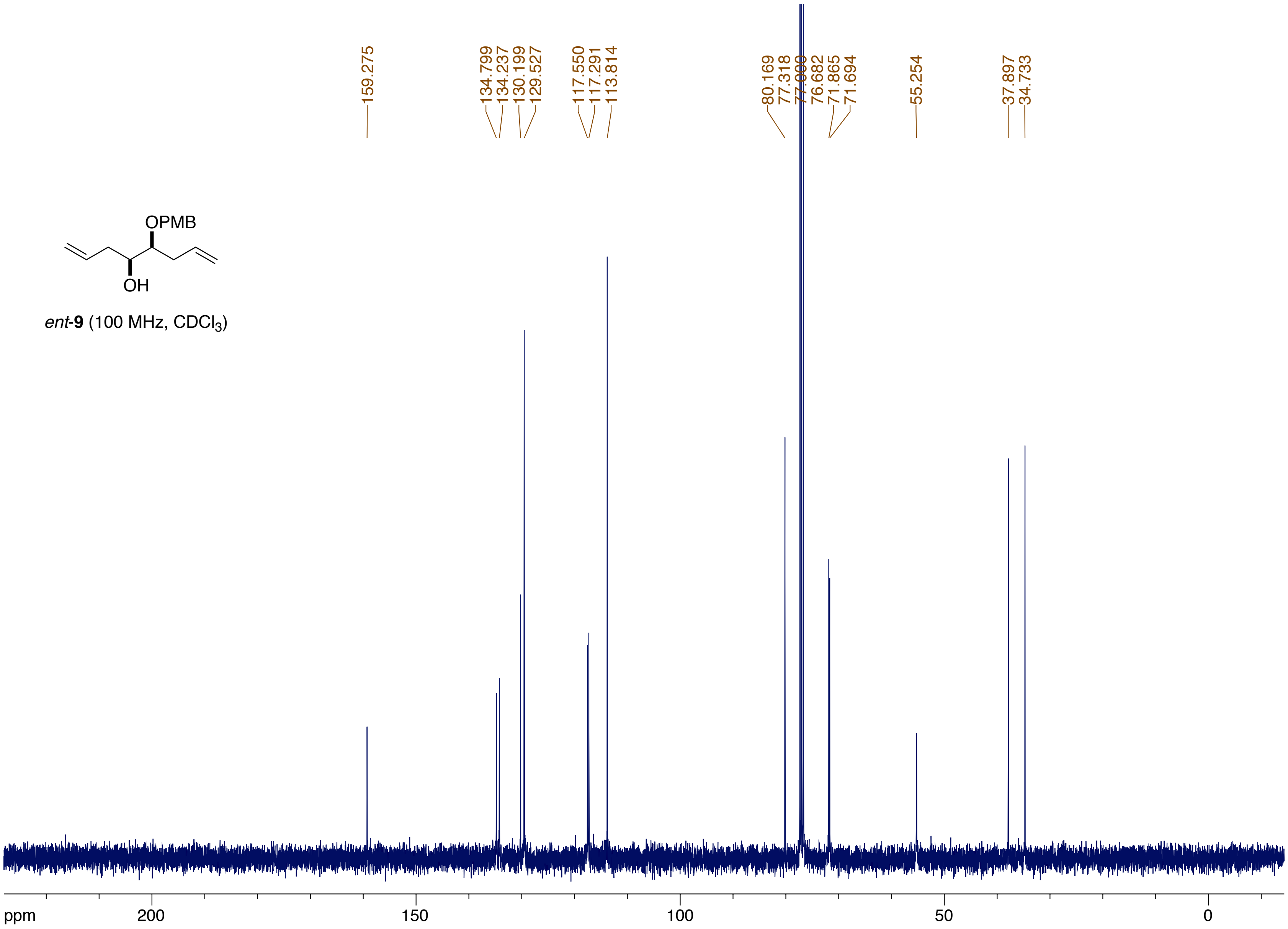


*ent*-**9** (100 MHz, $CDCl_3$)

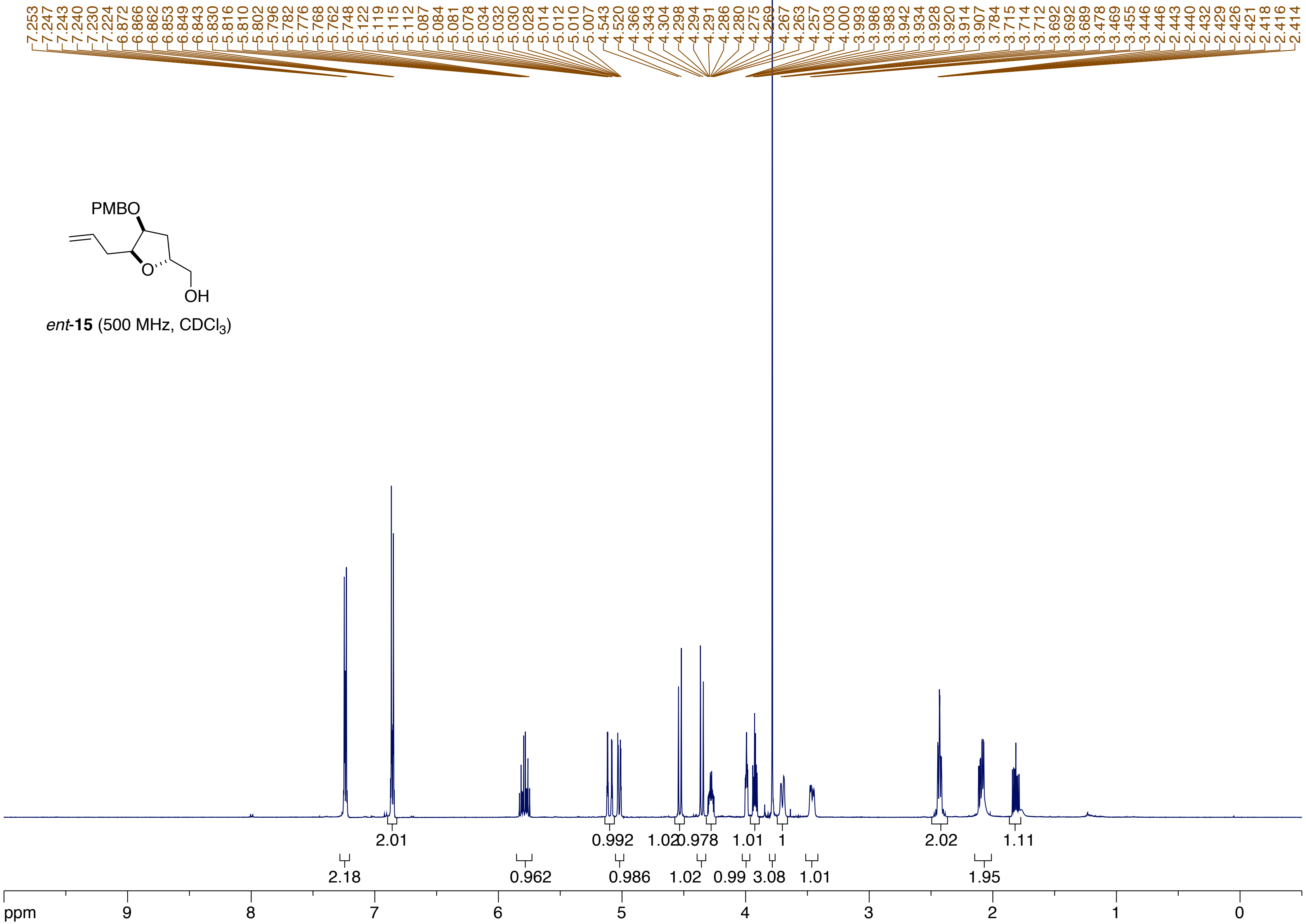
7.253 7.247 7.243 7.240 7.230 7.224 6.872 6.866 6.862 6.853 6.849 6.843 5.830 5.816 5.810 5.802 5.796 5.782 5.776 5.768 5.762 5.748 5.122 5.119 5.115 5.112 5.087 5.084 5.081 5.078 5.034 5.032 5.030 5.028 5.014 5.012 5.010 5.007 4.543 4.520 4.366 4.343 4.304 4.298 4.294 4.291 4.286 4.280 4.275 4.269 4.267 4.263 4.257 4.003 4.000 3.993 3.986 3.983 3.942 3.934 3.928 3.920 3.914 3.907 3.784 3.715 3.714 3.712 3.692 3.692 3.689 3.478 3.469 3.455 3.446 2.443 2.440 2.432 2.429 2.426 2.421 2.418 2.416 2.414
PMBO
O
OH
*ent*-**15** (500 MHz, $CDCl_3$)
2.01 0.992 1.02 0.978 1.01 1 2.02 1.11
2.18 0.962 0.986 1.02 0.99 3.08 1.01 1.95
ppm 9 8 7 6 5 4 3 2 1 0

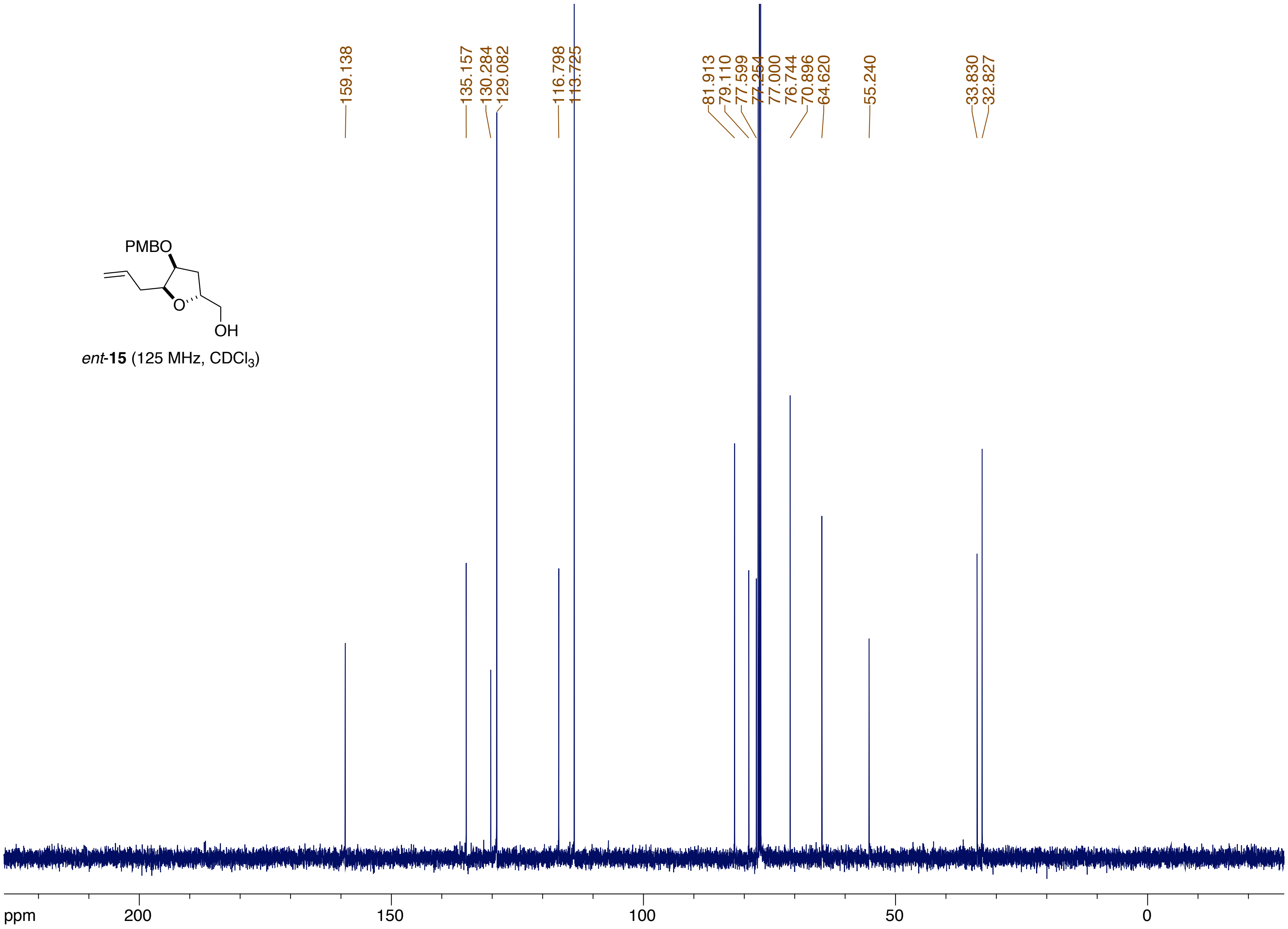
159.138
135.157
130.284
129.082
116.798
113.725
81.913
79.110
77.599
77.254
77.000
76.744
70.896
64.620
55.240
33.830
32.827
PMBO
O
OH
ent-15 (125 MHz, CDCl3)
ppm
200
150
100
50
0

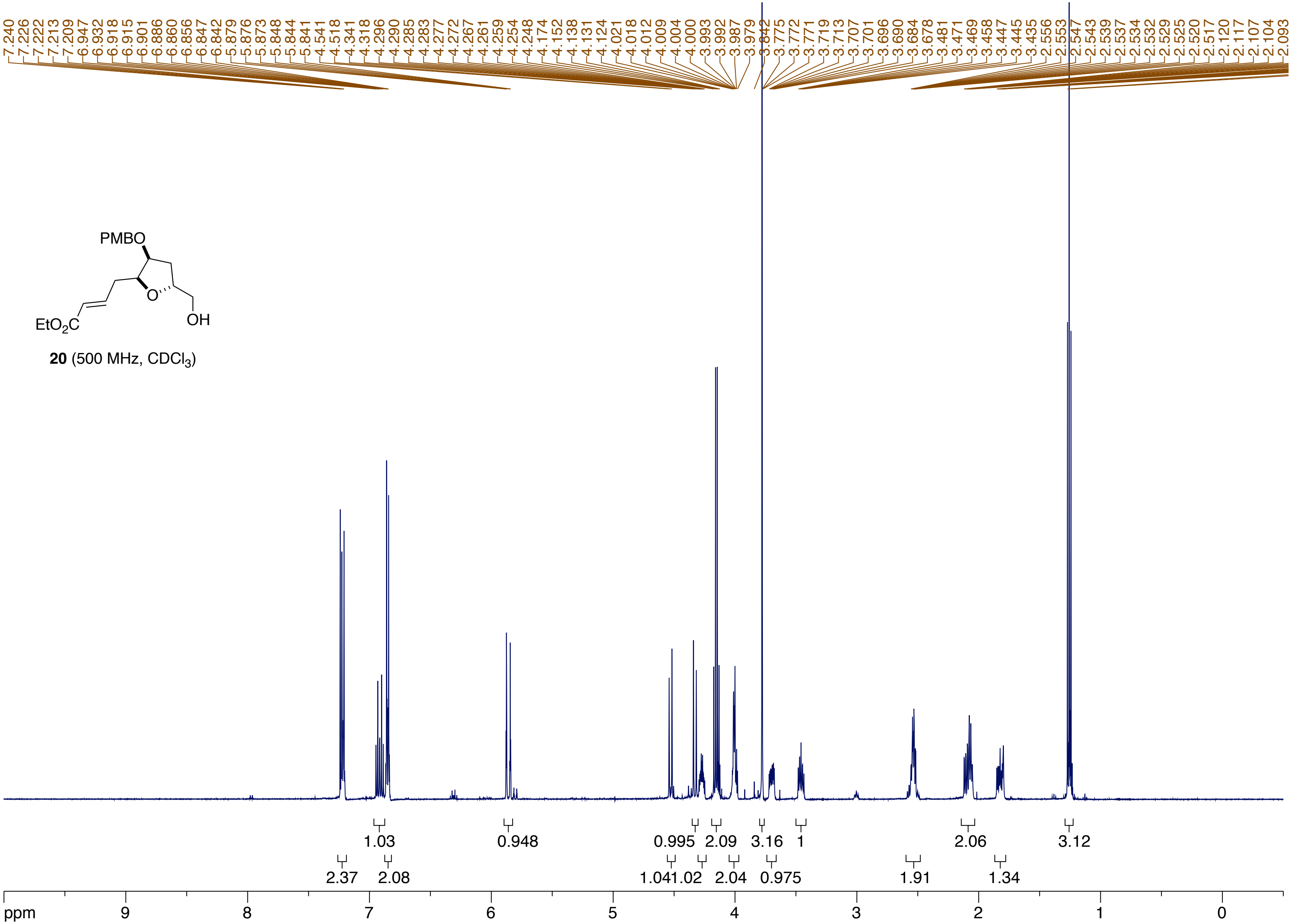
7.240 7.226 7.222 7.213 7.209 6.947 6.932 6.918 6.915 6.901 6.886 6.860 6.856 6.847 6.842 5.879 5.876 5.873 5.848 5.844 5.841 4.541 4.518 4.341 4.318 4.296 4.290 4.285 4.283 4.277 4.272 4.267 4.261 4.259 4.254 4.248 4.174 4.152 4.138 4.131 4.124 4.021 4.018 4.012 4.009 4.004 4.000 3.993 3.992 3.987 3.979 3.842 3.775 3.772 3.771 3.719 3.713 3.707 3.701 3.696 3.690 3.684 3.678 3.481 3.471 3.469 3.458 3.447 3.445 3.435 2.556 2.553 2.547 2.543 2.539 2.537 2.534 2.532 2.529 2.525 2.520 2.517 2.120 2.117 2.107 2.104 2.093
PMBO
O
EtO2C
OH
20 (500 MHz, CDCl3)
1.03 0.948 0.995 2.09 3.16 1 2.06 3.12
2.37 2.08 1.04 1.02 2.04 0.975 1.91 1.34
ppm 9 8 7 6 5 4 3 2 1 0

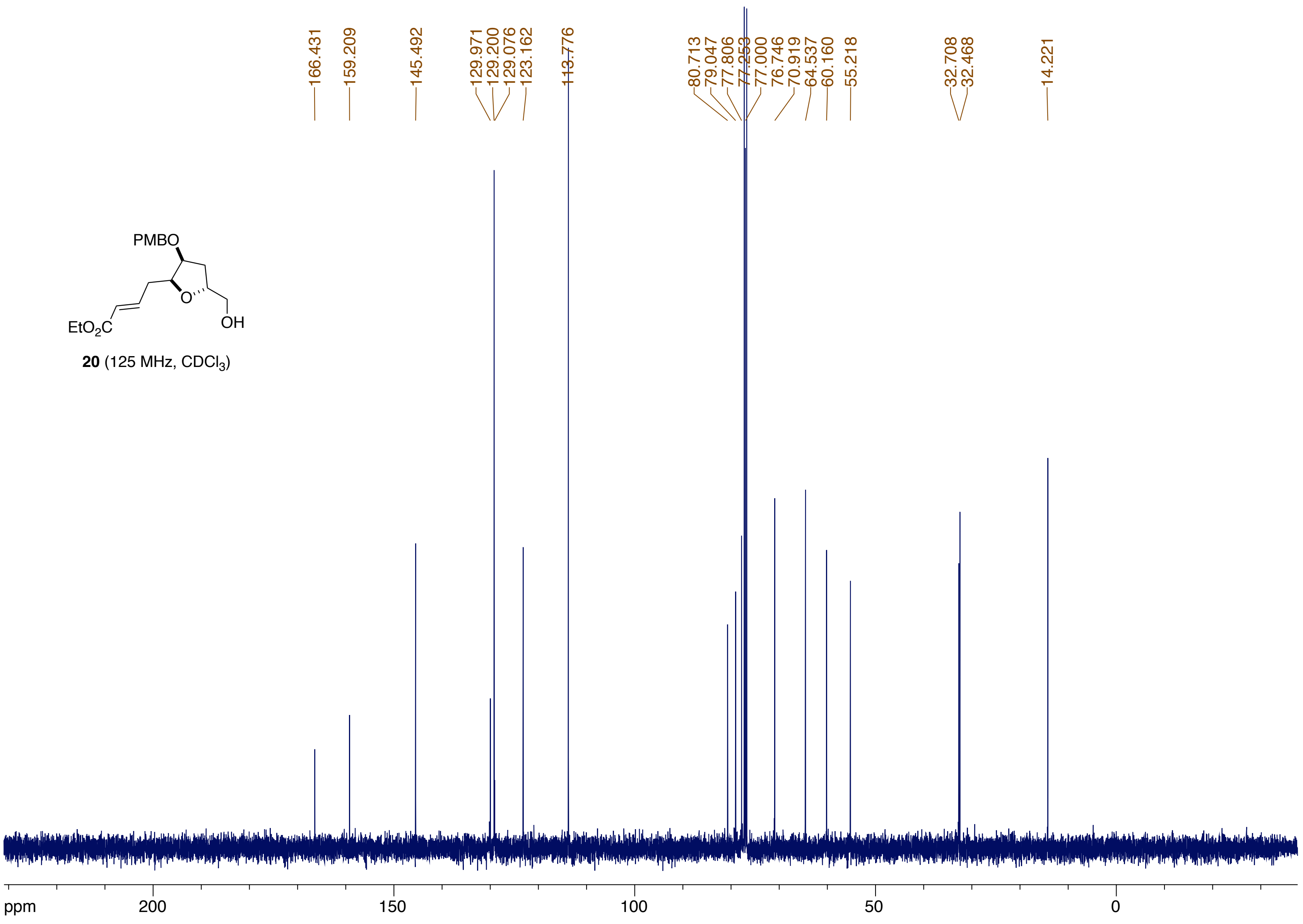


**20** (125 MHz, $CDCl_3$)

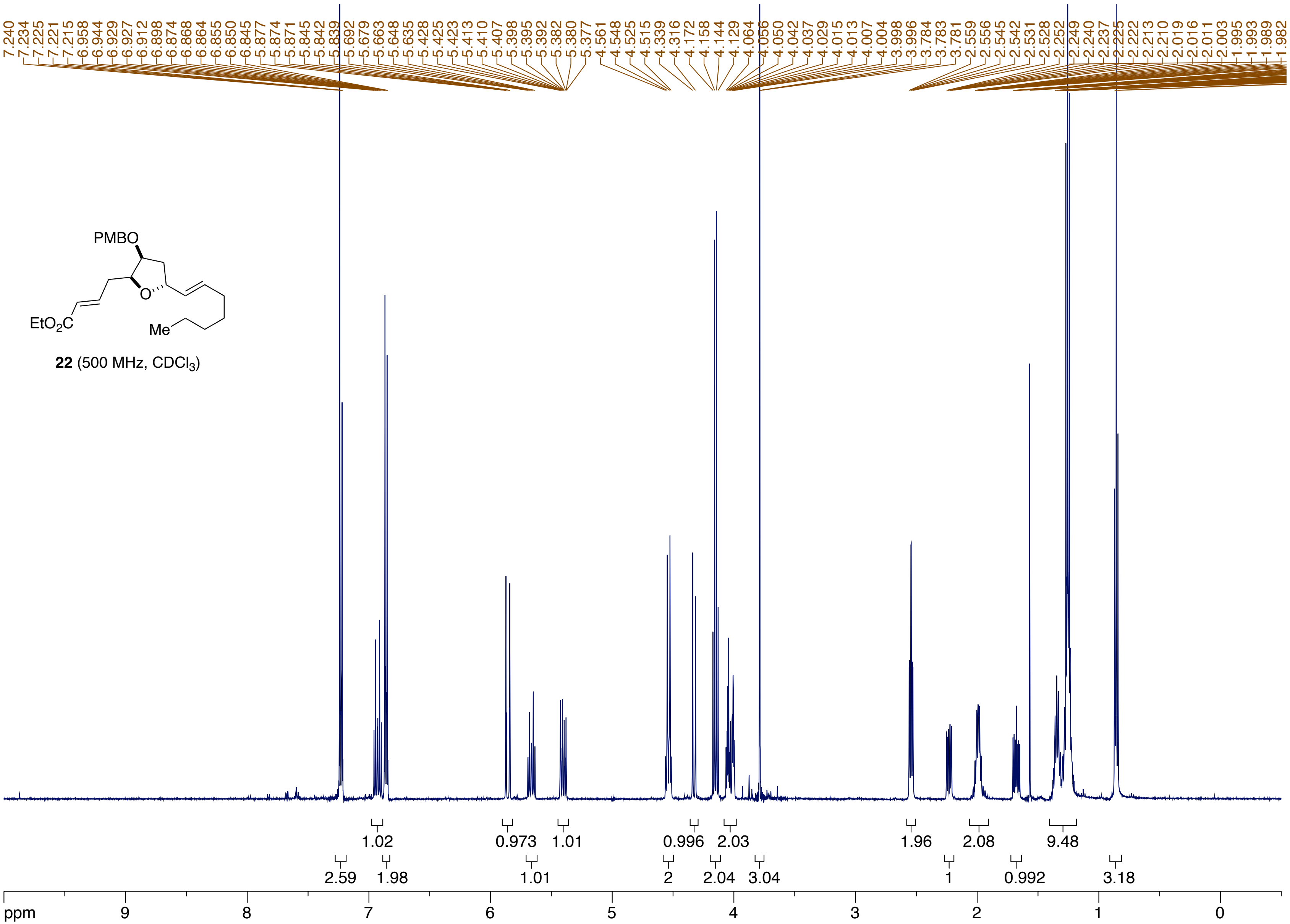


**22** (500 MHz, $CDCl_3$)

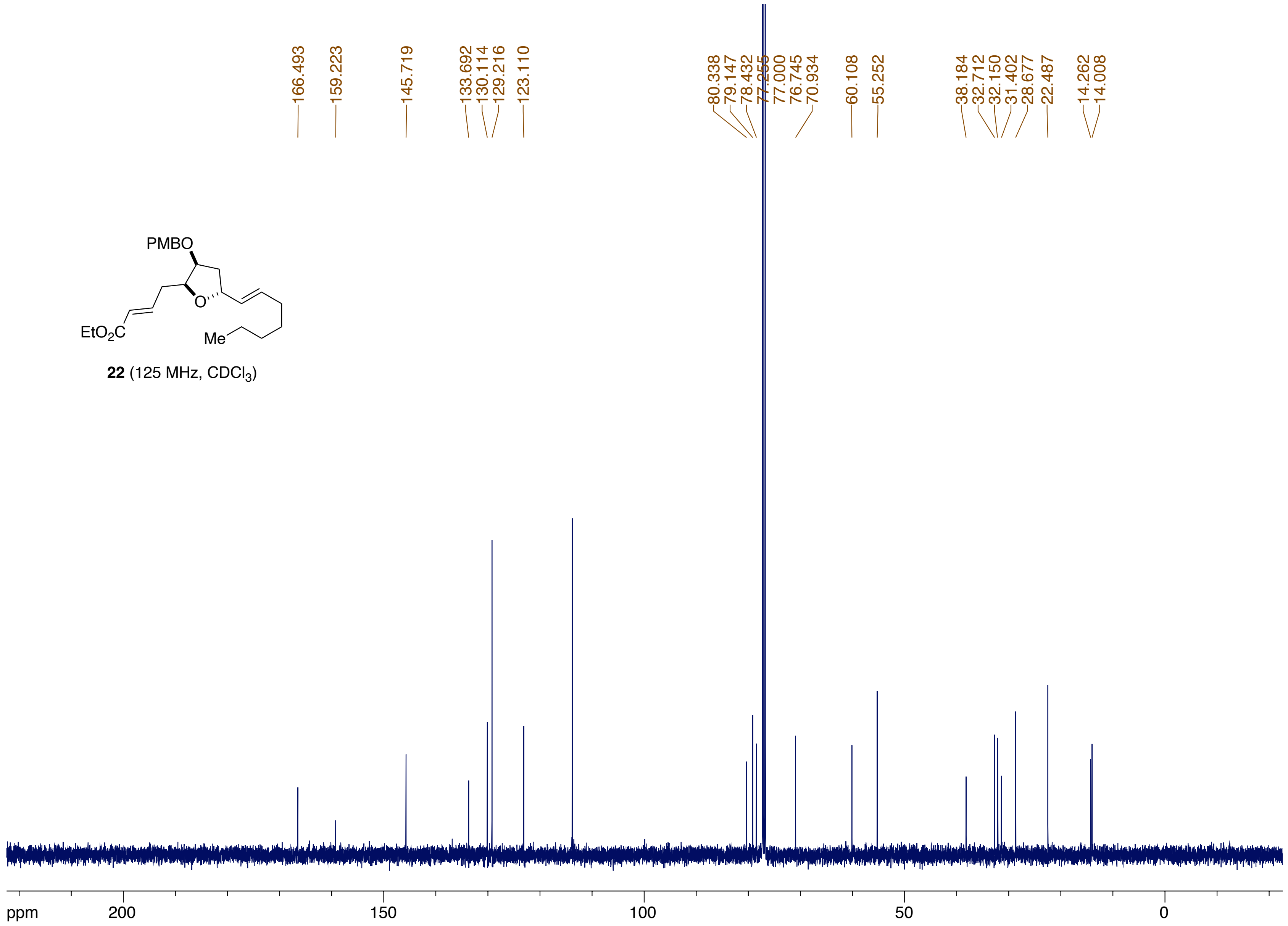
166.493
159.223
145.719
133.692
130.114
129.216
123.110
80.338
79.147
78.432
77.256
77.000
76.745
70.934
60.108
55.252
38.184
32.712
32.150
31.402
28.677
22.487
14.262
14.008
PMBO
O
EtO2C
Me
22 (125 MHz, CDCl3)
ppm
200
150
100
50
0

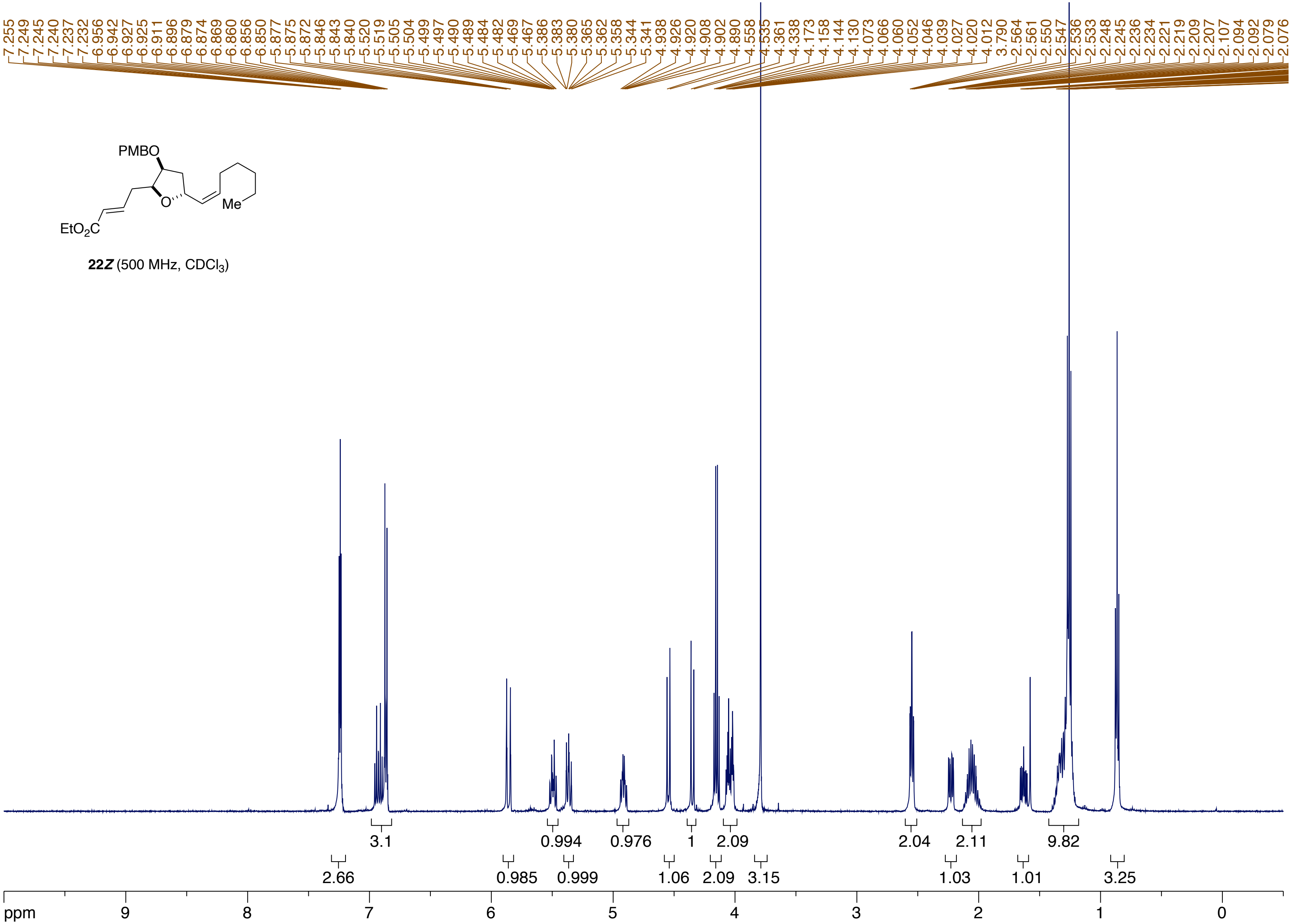
7.255 7.249 7.245 7.240 7.237 7.232 6.956 6.942 6.927 6.925 6.911 6.896 6.879 6.874 6.869 6.860 6.856 6.850 5.877 5.875 5.872 5.846 5.843 5.840 5.520 5.519 5.505 5.504 5.499 5.497 5.490 5.489 5.484 5.482 5.469 5.467 5.386 5.383 5.380 5.365 5.362 5.358 5.344 5.341 4.938 4.926 4.920 4.908 4.902 4.890 4.558 4.535 4.361 4.338 4.173 4.158 4.144 4.130 4.073 4.066 4.060 4.052 4.046 4.039 4.027 4.020 4.012 3.790 2.564 2.561 2.550 2.547 2.536 2.533 2.248 2.245 2.236 2.234 2.221 2.219 2.209 2.207 2.107 2.094 2.092 2.079 2.076
PMBO
Me
$EtO_2C$
O
**22*Z*** (500 MHz, $CDCl_3$)
3.1
0.994
0.976
1
2.09
2.04
2.11
9.82
2.66
0.985
0.999
1.06
2.09
3.15
1.03
1.01
3.25
ppm
9
8
7
6
5
4
3
2
1
0

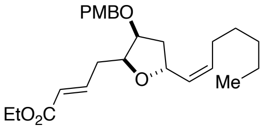

**22*Z*** (125 MHz, $CDCl_3$)

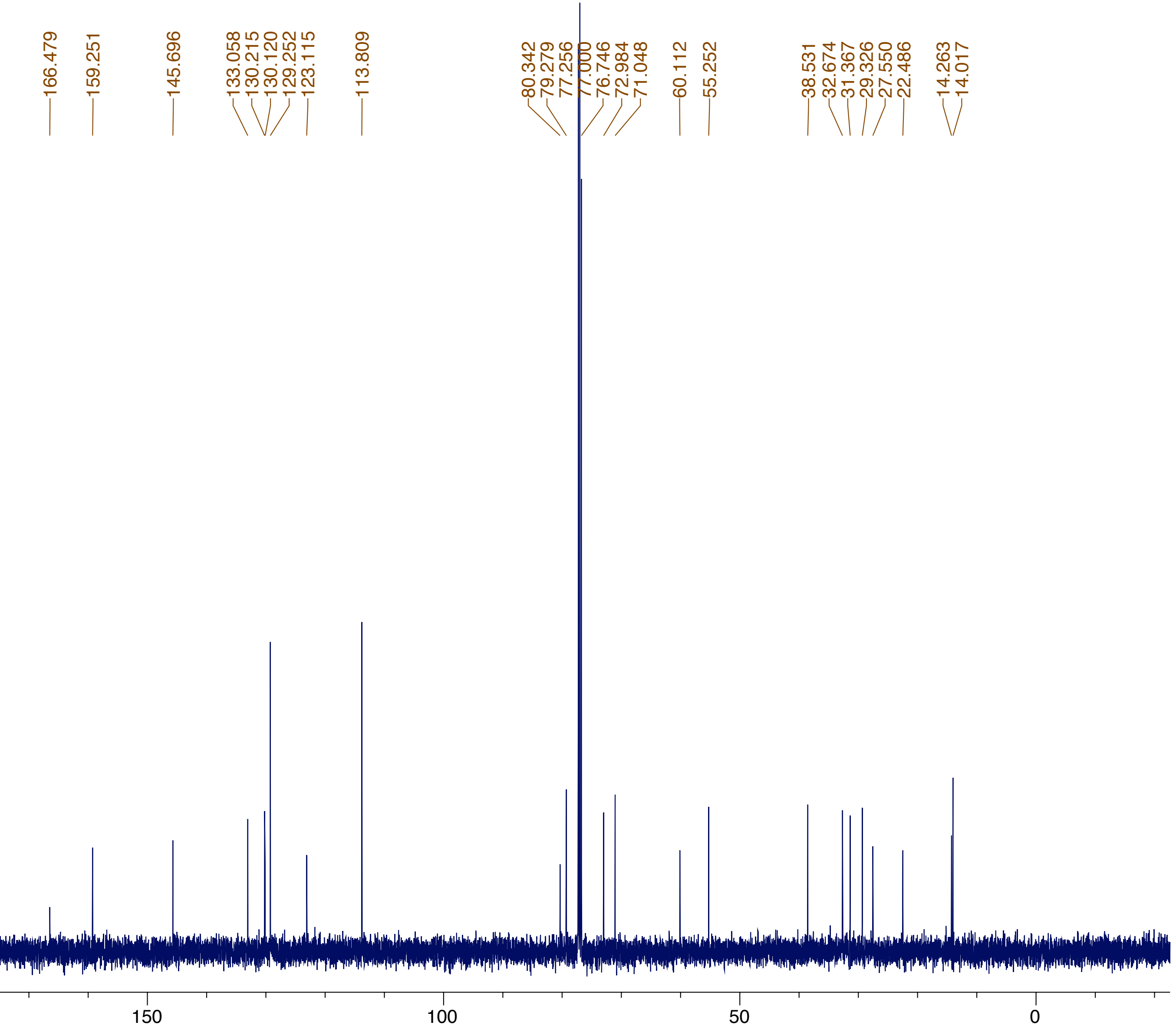

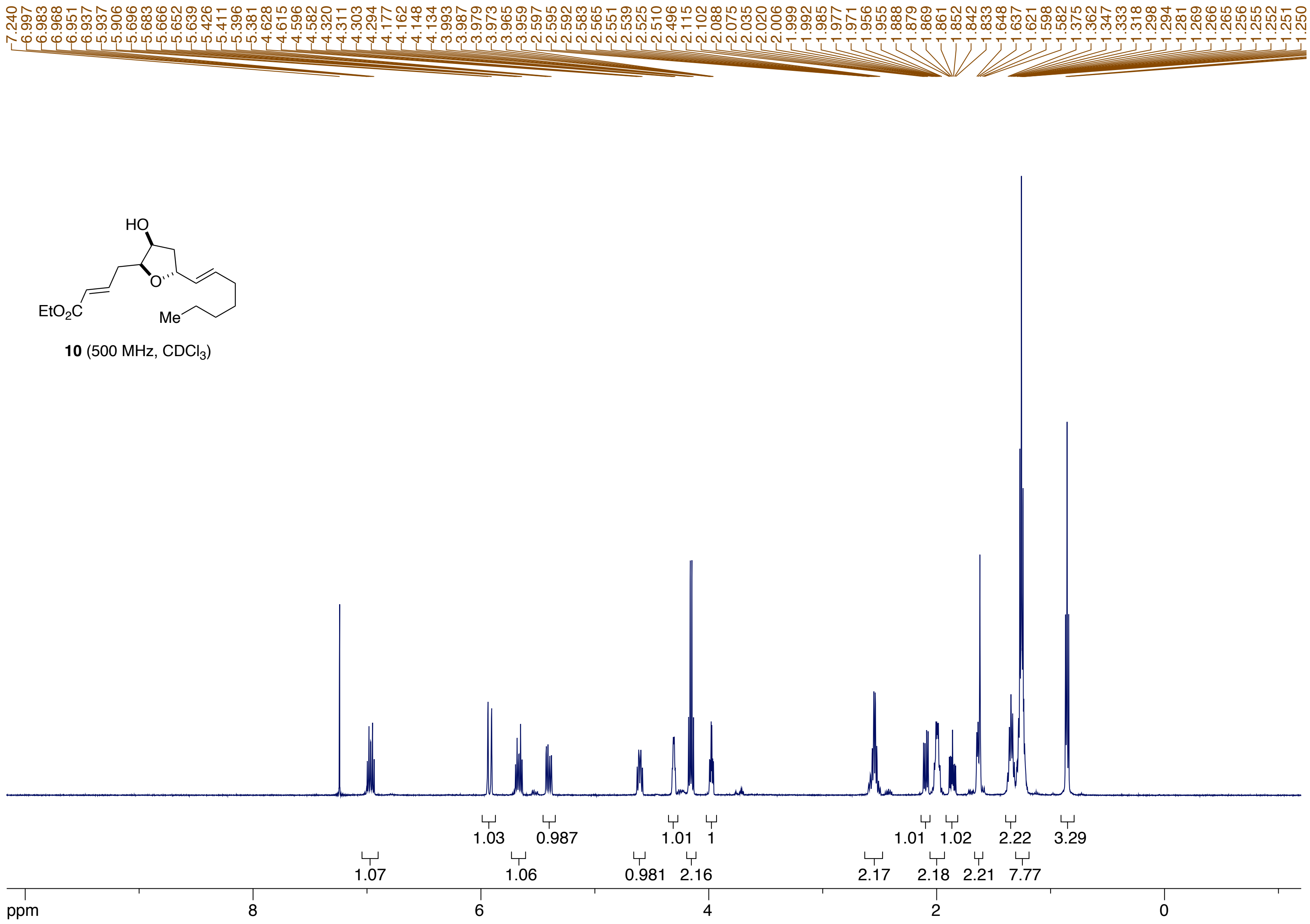

**10** (500 MHz, $CDCl_3$)

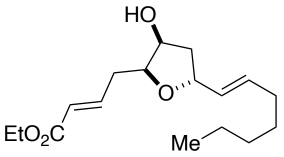


**10** (125 MHz, $CDCl_3$)

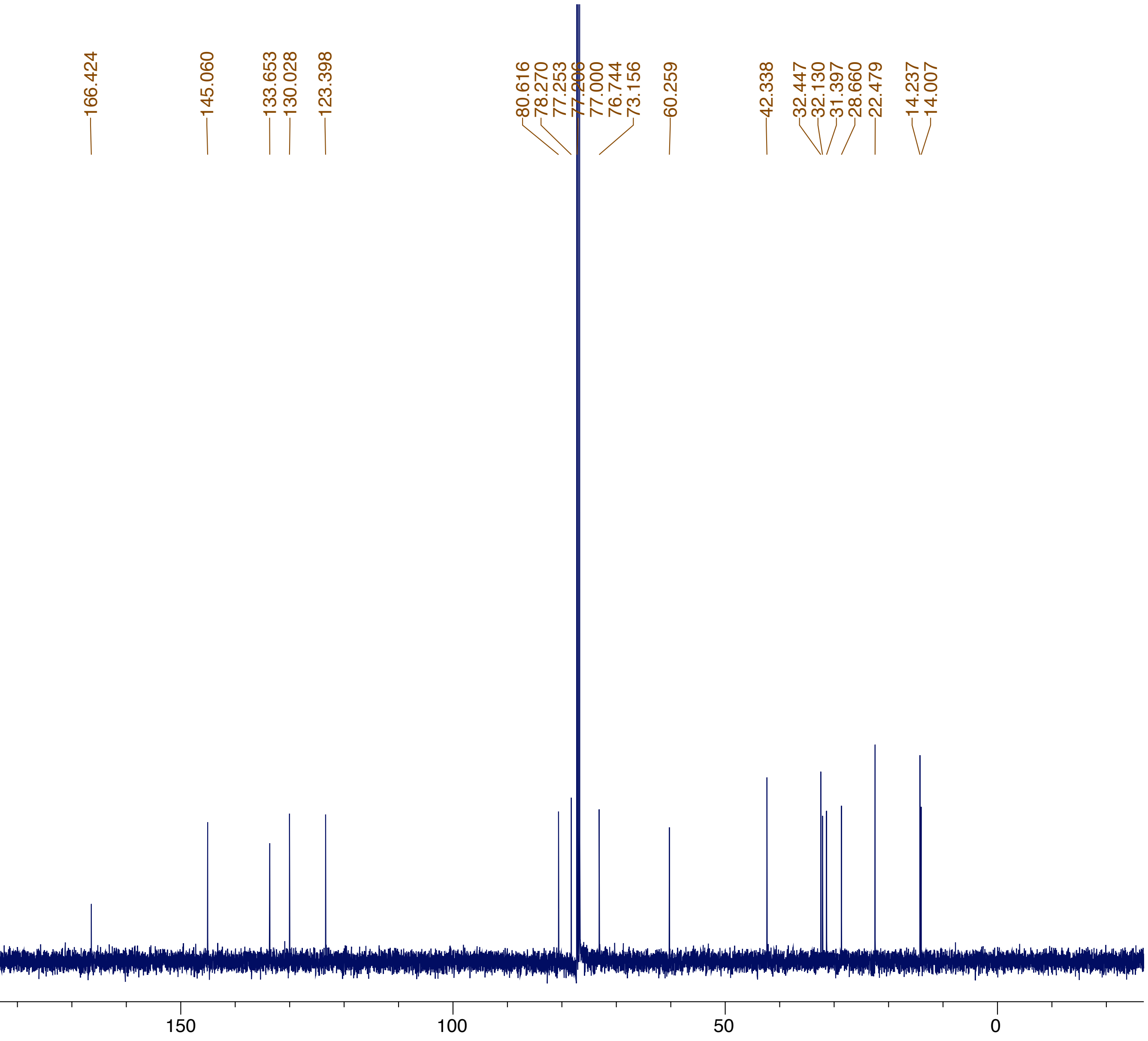

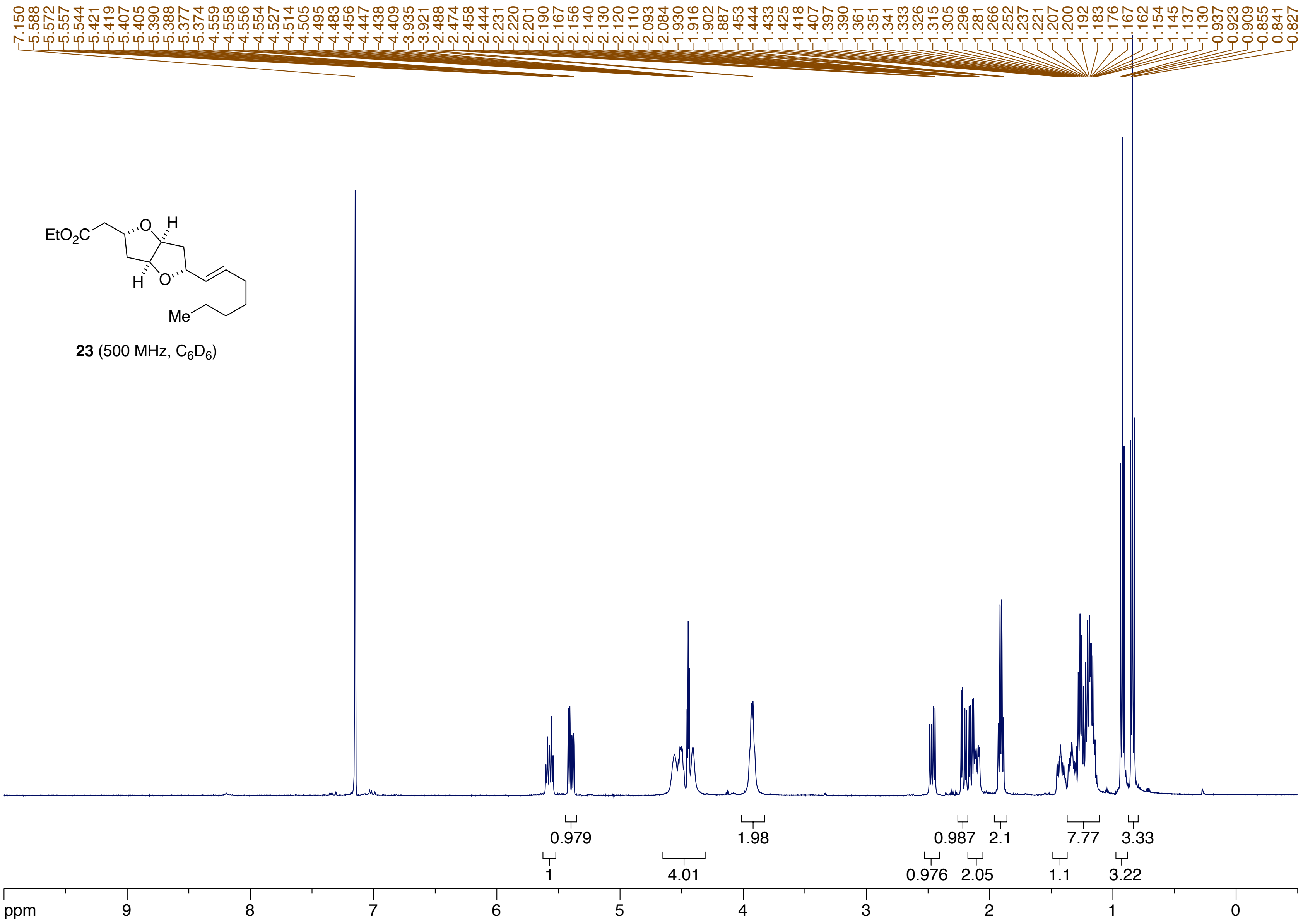
7.150 5.588 5.572 5.557 5.544 5.421 5.419 5.407 5.405 5.390 5.388 5.377 5.374 4.559 4.558 4.556 4.554 4.527 4.514 4.505 4.495 4.483 4.456 4.447 4.438 4.409 3.935 3.921 2.488 2.474 2.458 2.444 2.231 2.220 2.201 2.190 2.167 2.156 2.140 2.130 2.120 2.110 2.093 2.084 1.930 1.916 1.902 1.887 1.453 1.444 1.433 1.425 1.418 1.407 1.397 1.390 1.361 1.351 1.341 1.333 1.326 1.315 1.305 1.296 1.281 1.266 1.252 1.237 1.221 1.207 1.200 1.192 1.183 1.176 1.167 1.162 1.154 1.145 1.137 1.130 0.937 0.923 0.909 0.855 0.841 0.827
EtO2C
O
H
H
O
Me
23 (500 MHz, C6D6)
0.979
1.98
0.987
2.1
7.77
3.33
1
4.01
0.976
2.05
1.1
3.22
ppm 9 8 7 6 5 4 3 2 1 0

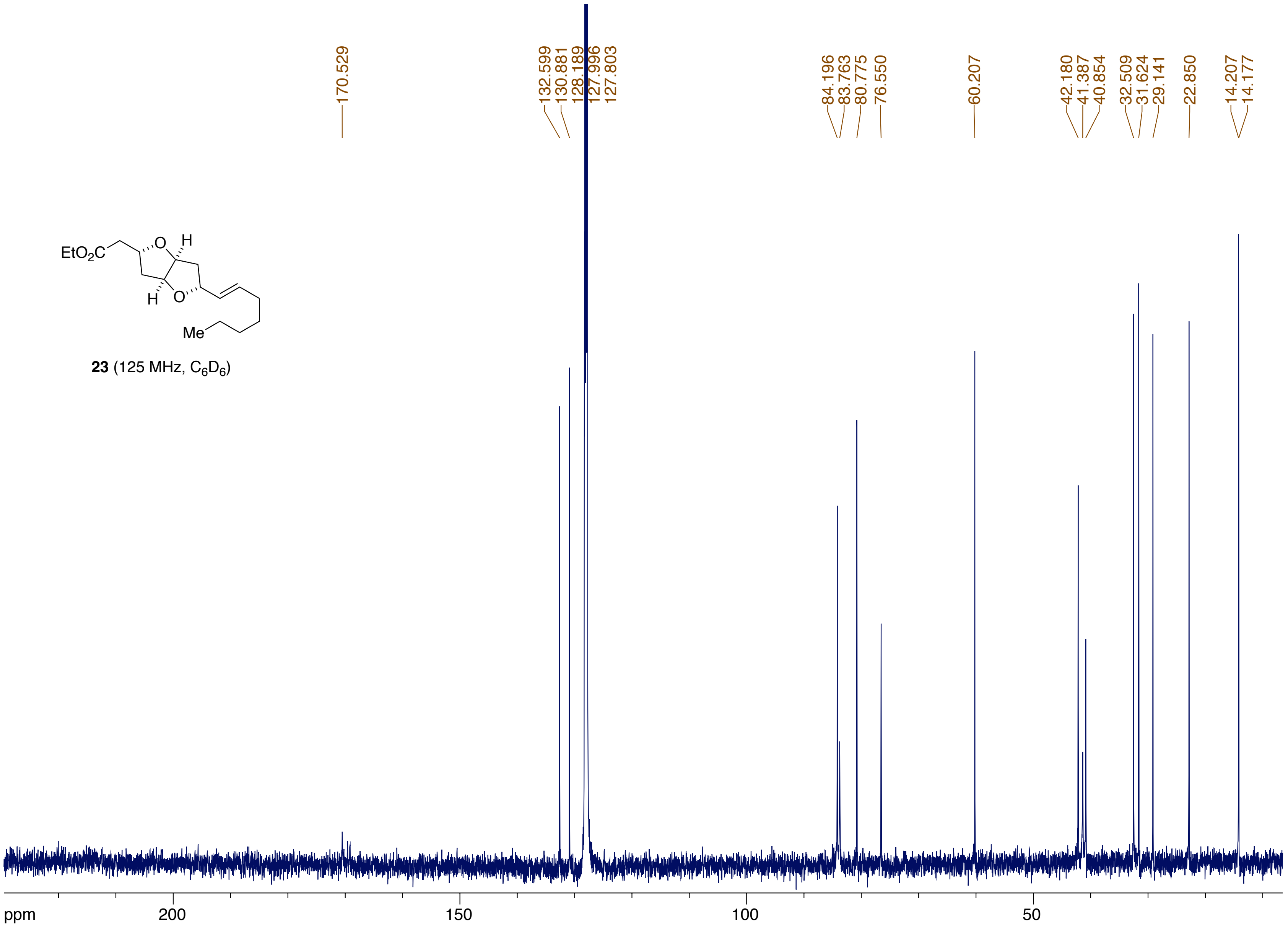


**23** (125 MHz, $C_6D_6$)

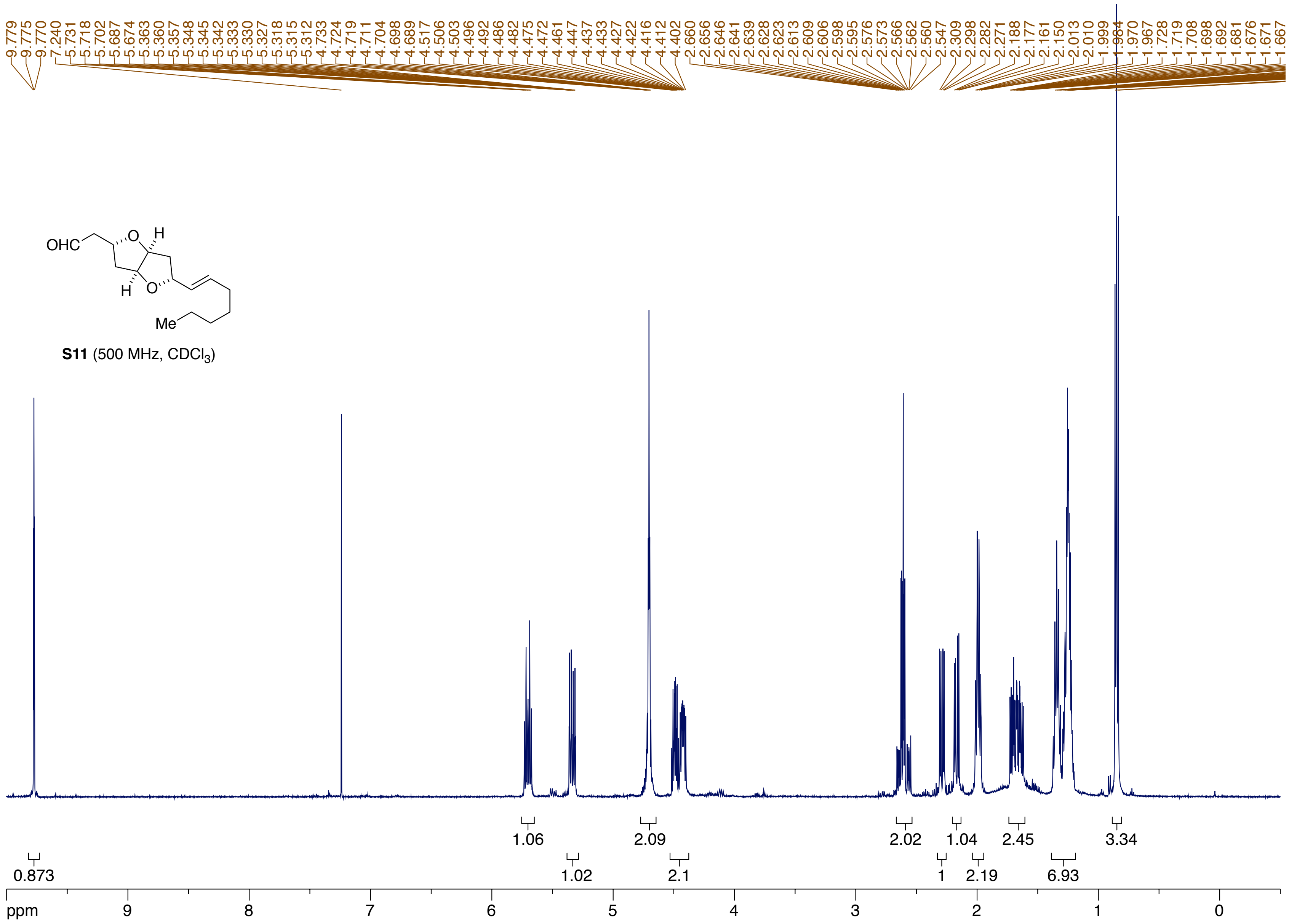

**S11** (500 MHz, $CDCl_3$)

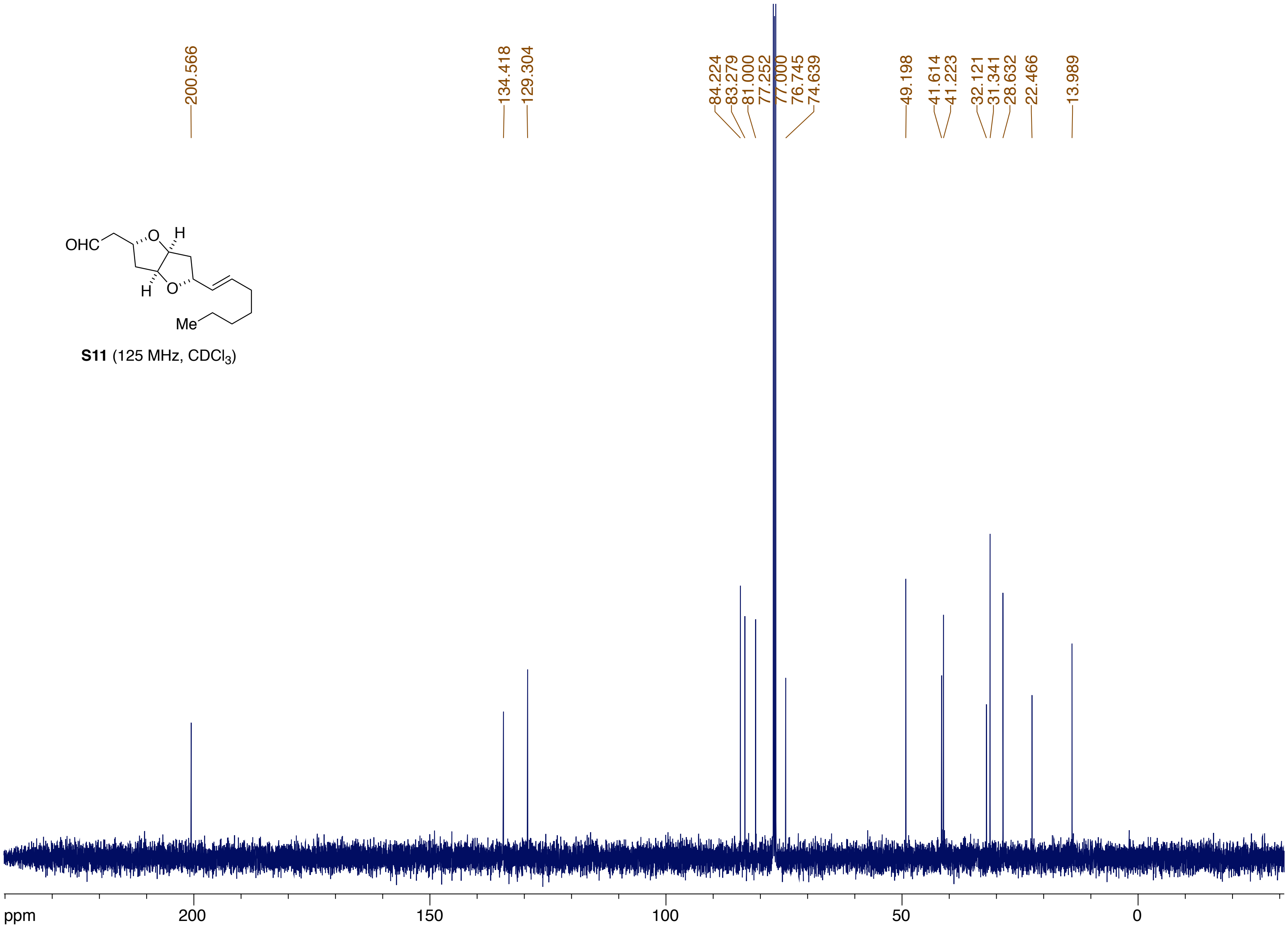
200.566
134.418
129.304
84.224
83.279
81.000
77.252
77.000
76.745
74.639
49.198
41.614
41.223
32.121
31.341
28.632
22.466
13.989
OHC
O
H
H
O
Me
S11 (125 MHz, $CDCl_3$)
ppm
200
150
100
50
0

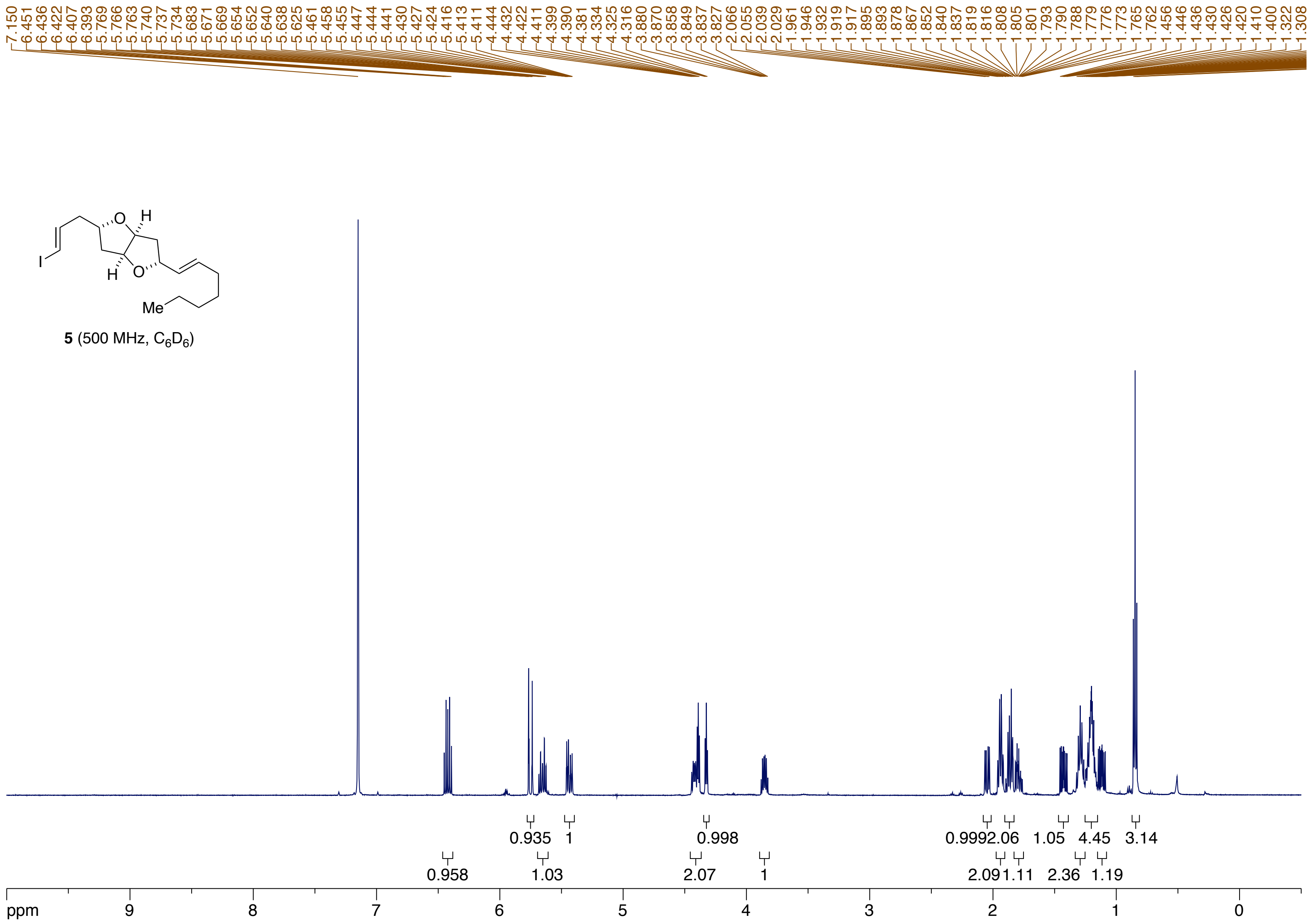

**5** (500 MHz, $C_6D_6$)

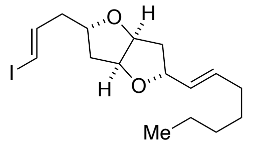

**5** (125 MHz, $C_6D_6$)

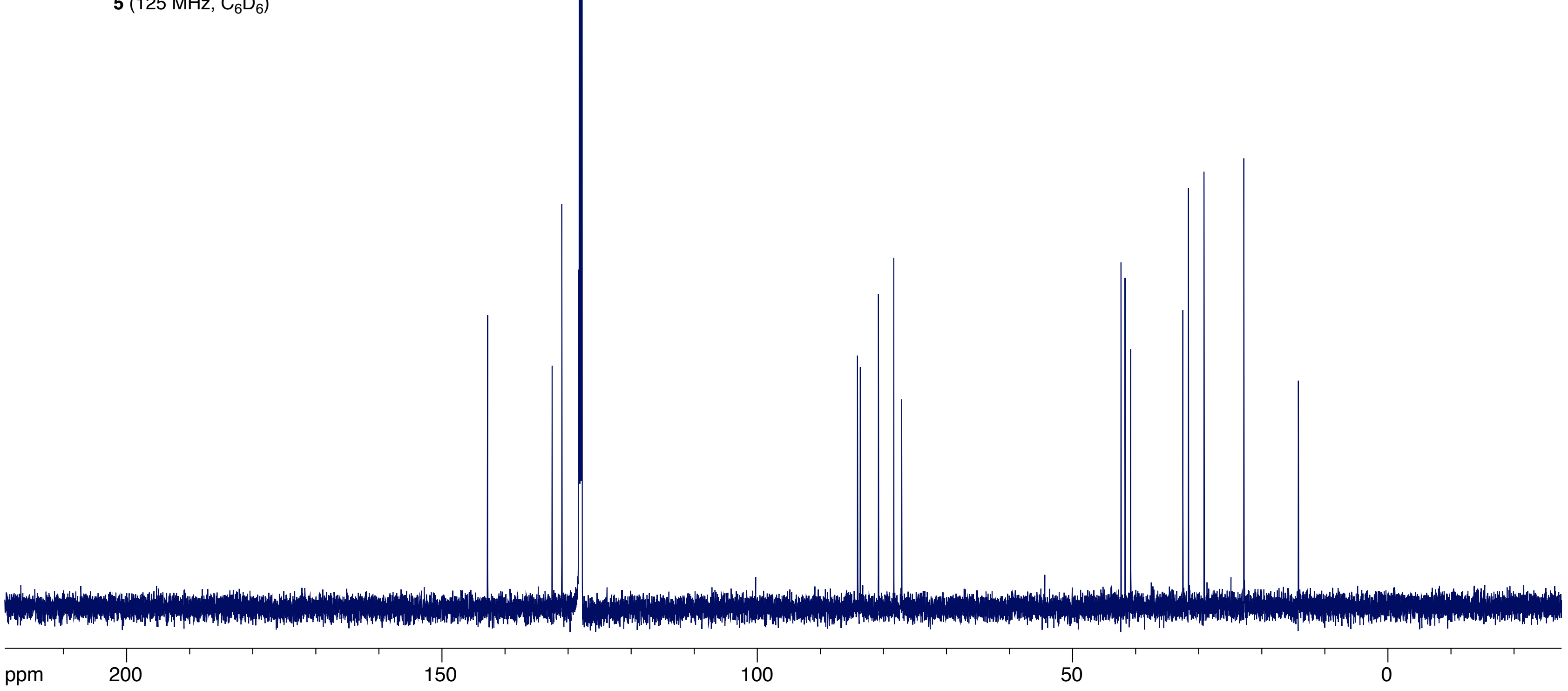

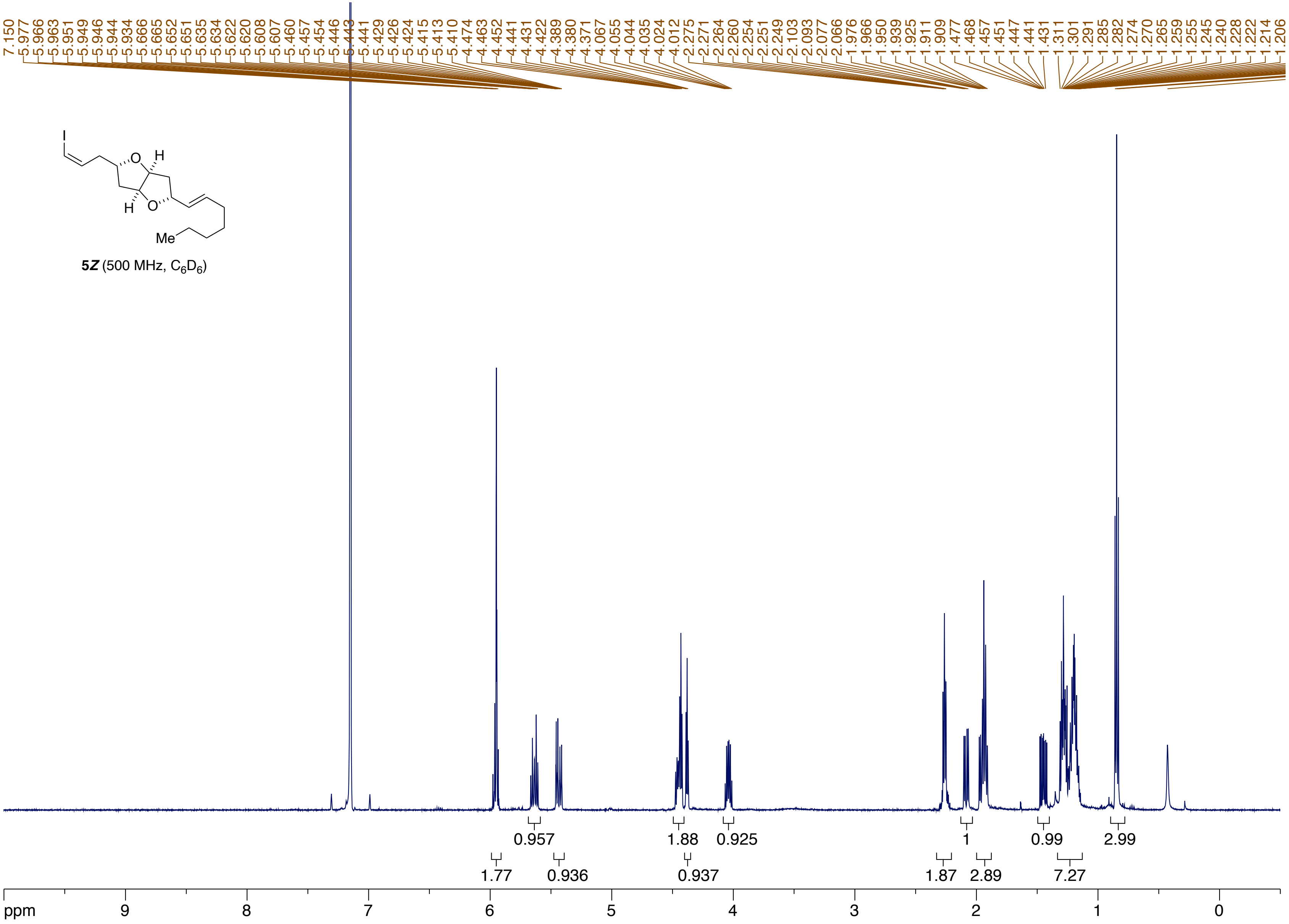
7.150
5.977
5.966
5.963
5.951
5.949
5.946
5.944
5.934
5.666
5.665
5.652
5.651
5.635
5.634
5.622
5.620
5.608
5.607
5.460
5.457
5.454
5.446
5.443
5.441
5.429
5.426
5.424
5.415
5.413
5.410
4.474
4.463
4.452
4.441
4.431
4.422
4.389
4.380
4.371
4.067
4.055
4.044
4.035
4.024
4.012
2.275
2.271
2.264
2.260
2.254
2.251
2.249
2.103
2.093
2.077
2.066
1.976
1.966
1.950
1.939
1.925
1.911
1.909
1.477
1.468
1.457
1.451
1.447
1.441
1.431
1.311
1.301
1.291
1.285
1.282
1.274
1.270
1.265
1.259
1.255
1.245
1.240
1.228
1.222
1.214
1.206
I
O
H
H
O
Me
5Z (500 MHz, C6D6)
0.957
1.88
0.925
1
0.99
2.99
1.77
0.936
0.937
1.87
2.89
7.27
ppm
9
8
7
6
5
4
3
2
1
0

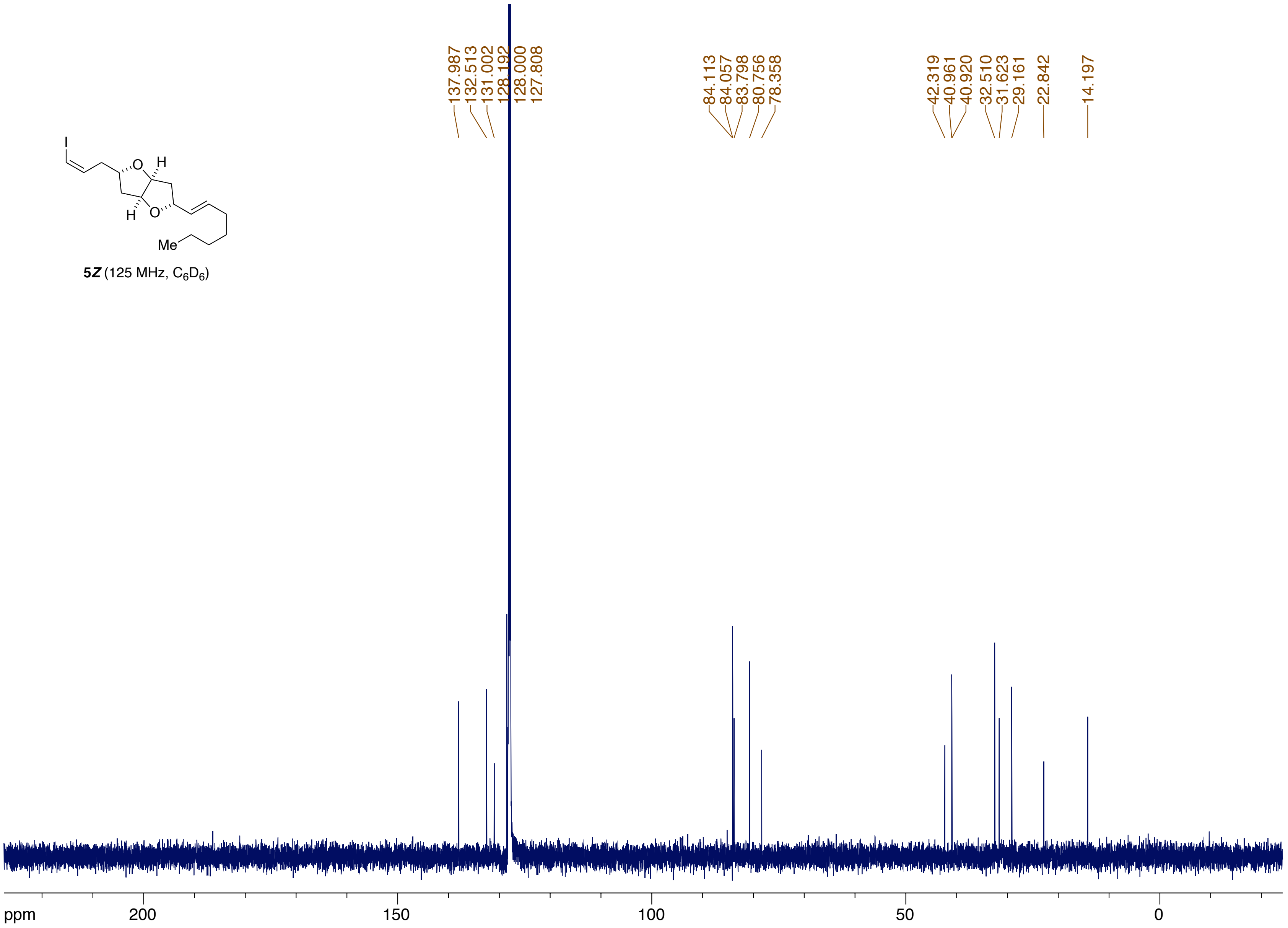


**5*Z*** (125 MHz, $C_6D_6$)

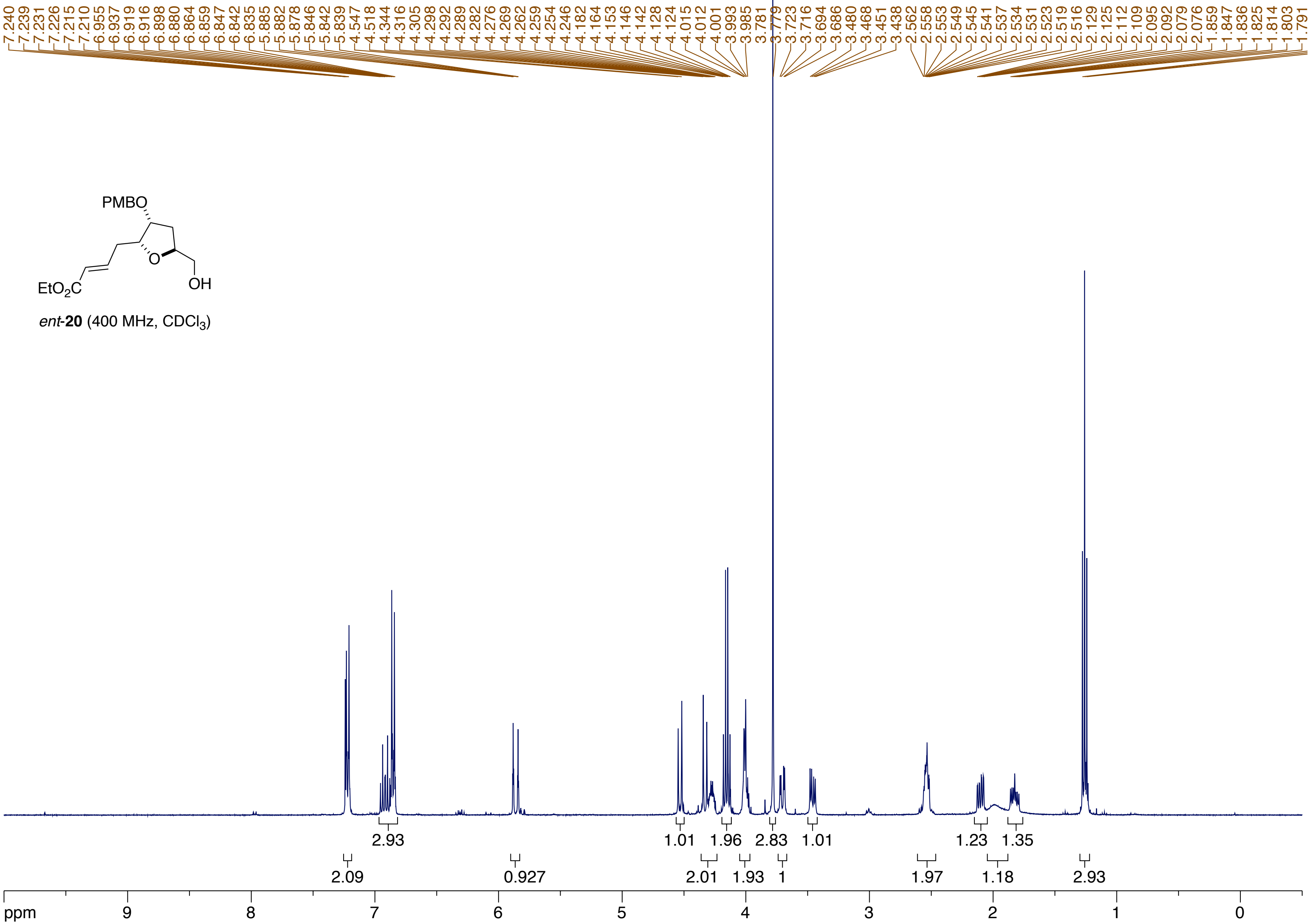
PMBO
O
OH
EtO$_2$C
*ent*-**20** (400 MHz, $CDCl_3$)
7.240
7.239
7.231
7.226
7.215
7.210
6.955
6.937
6.919
6.916
6.898
6.880
6.864
6.859
6.847
6.842
6.835
5.885
5.882
5.878
5.846
5.842
5.839
4.547
4.518
4.344
4.316
4.305
4.298
4.292
4.289
4.282
4.276
4.269
4.262
4.259
4.254
4.246
4.182
4.164
4.153
4.146
4.142
4.128
4.124
4.015
4.012
4.001
3.993
3.985
3.781
3.779
3.723
3.716
3.694
3.686
3.480
3.468
3.451
3.438
2.562
2.558
2.553
2.549
2.545
2.541
2.537
2.534
2.531
2.523
2.519
2.516
2.129
2.125
2.112
2.109
2.095
2.092
2.079
2.076
1.859
1.847
1.836
1.825
1.814
1.803
1.791
2.93
1.01
1.96
2.83
1.01
1.23
1.35
2.09
0.927
2.01
1.93
1
1.97
1.18
2.93
ppm
9
8
7
6
5
4
3
2
1
0

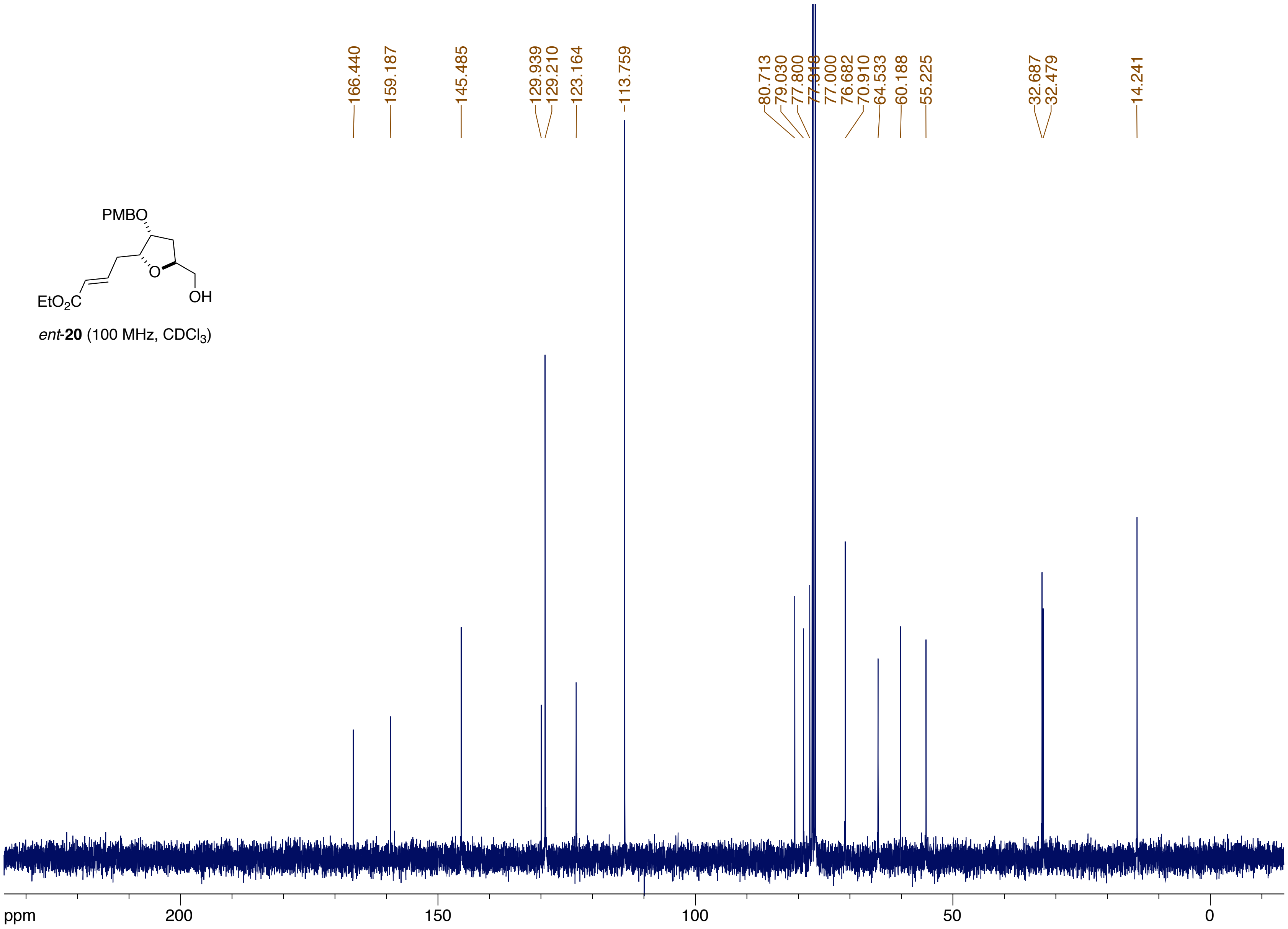


*ent*-**20** (100 MHz, $CDCl_3$)

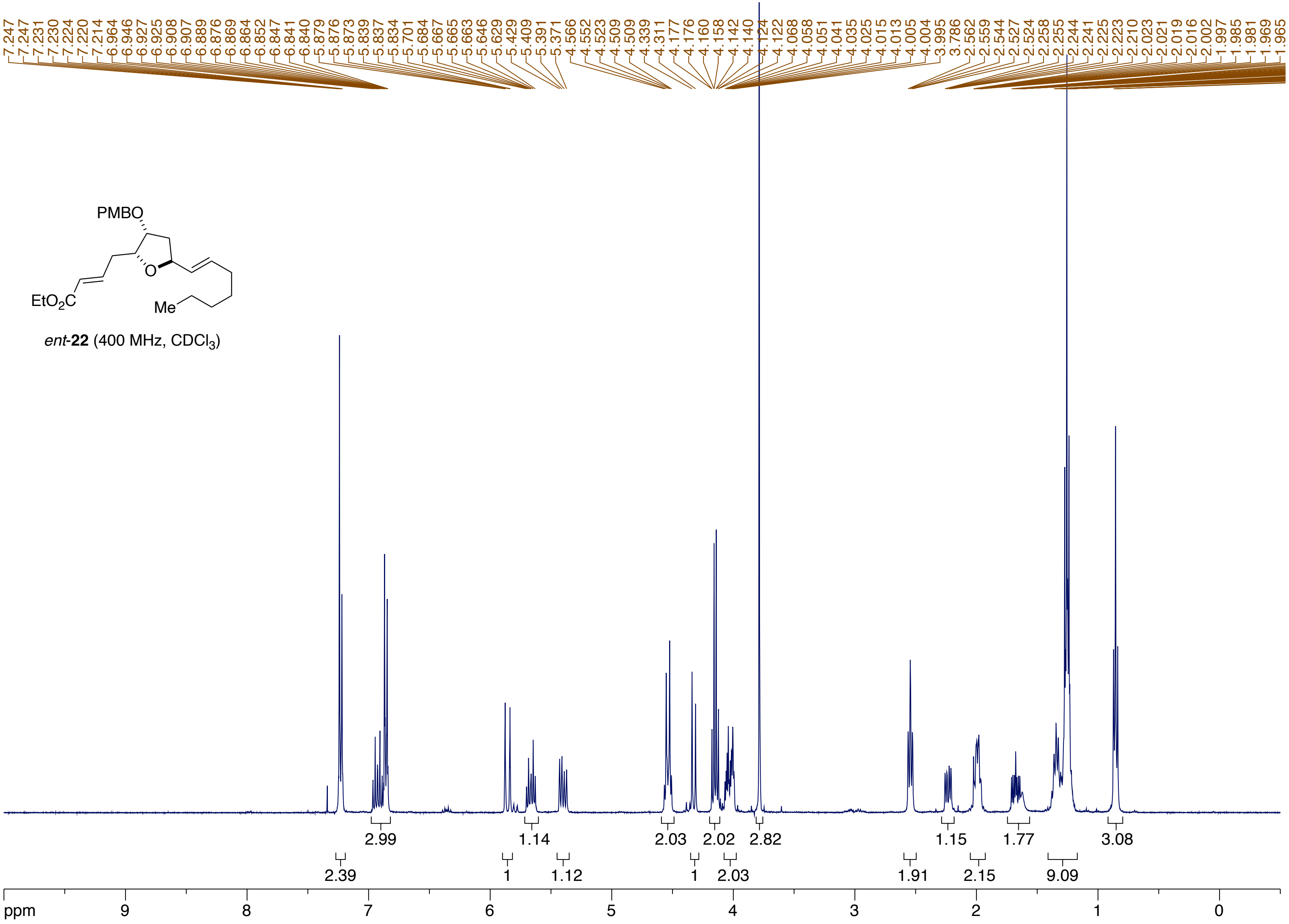
PMBO
EtO2C
Me
ent-22 (400 MHz, CDCl3)
ppm

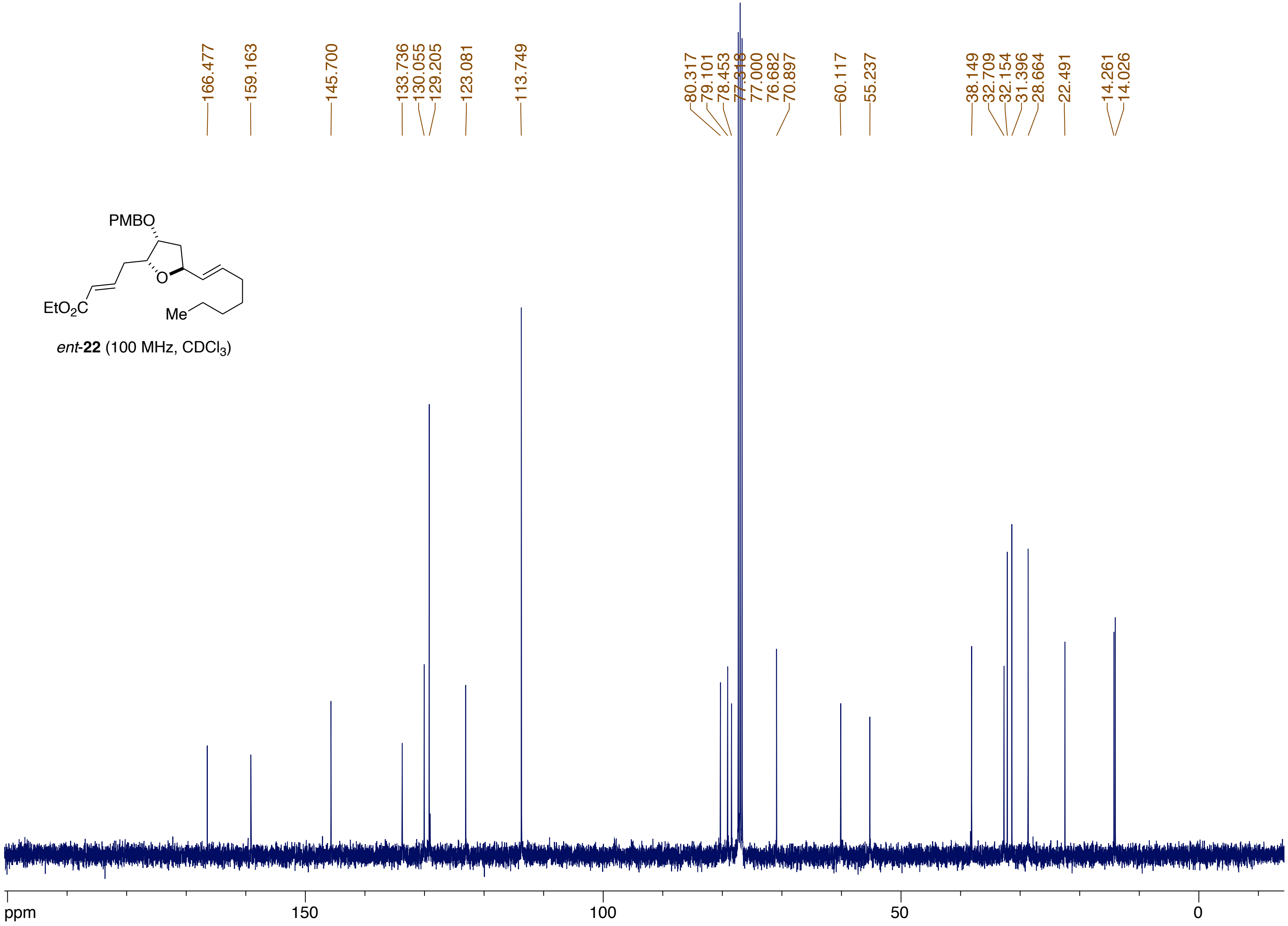
166.477
159.163
145.700
133.736
130.055
129.205
123.081
113.749
80.317
79.101
78.453
77.318
77.000
76.682
70.897
60.117
55.237
38.149
32.709
32.154
31.396
28.664
22.491
14.261
14.026
PMBO
O
$EtO_2C$
Me
*ent*-**22** (100 MHz, $CDCl_3$)
ppm
150
100
50
0

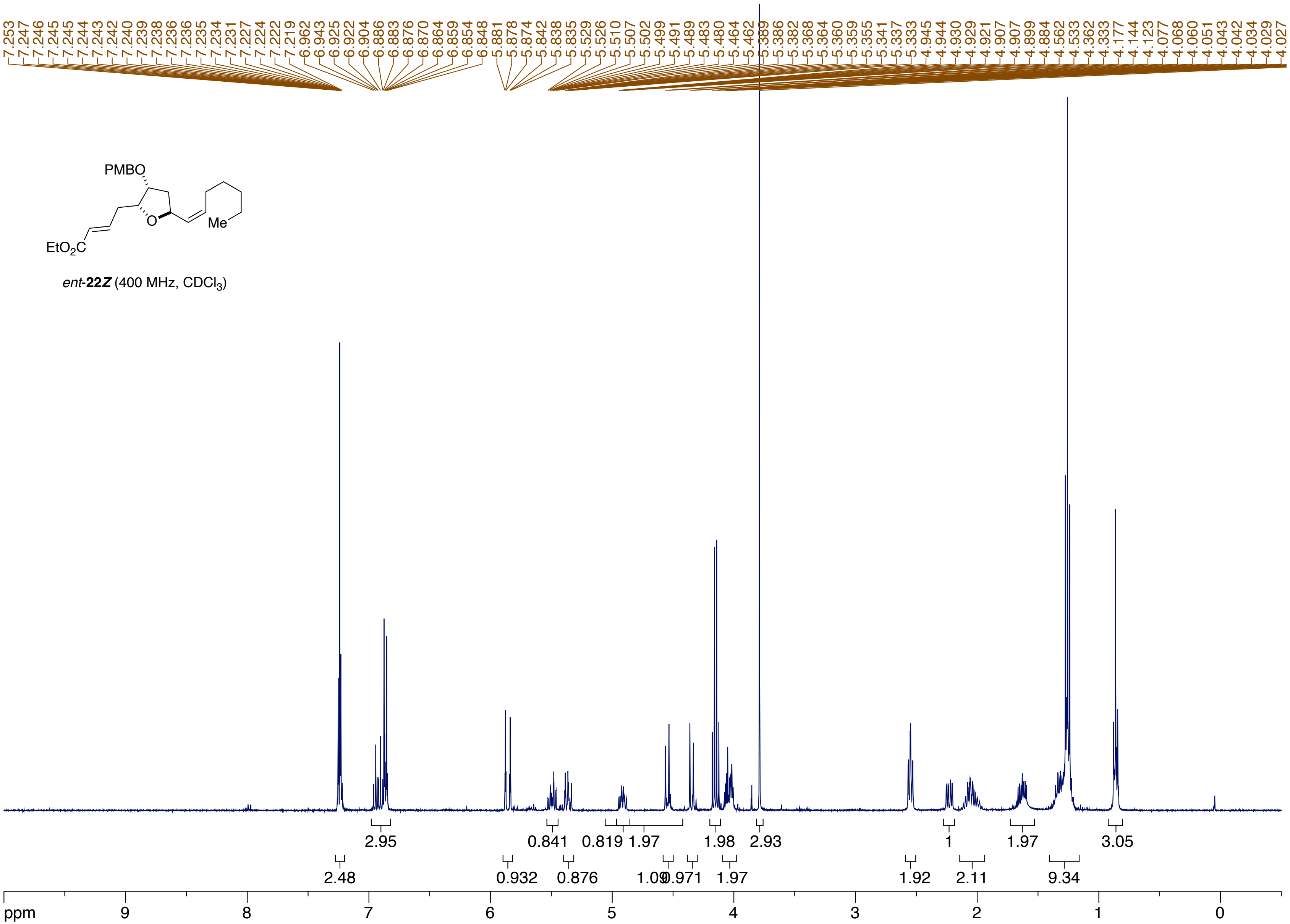


*ent*-**22*Z*** (400 MHz, $CDCl_3$)

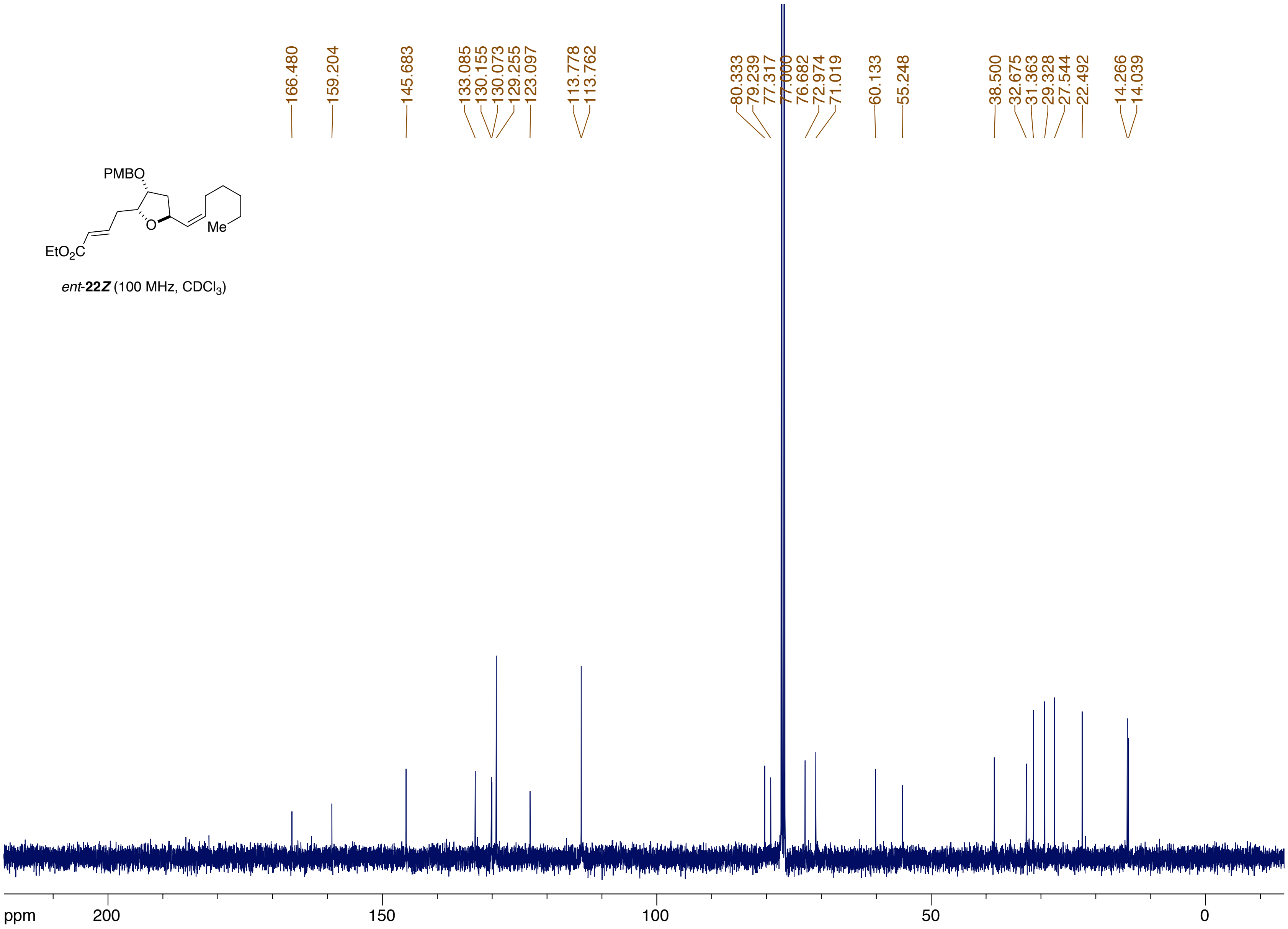


*ent*-**22*Z*** (100 MHz, $CDCl_3$)

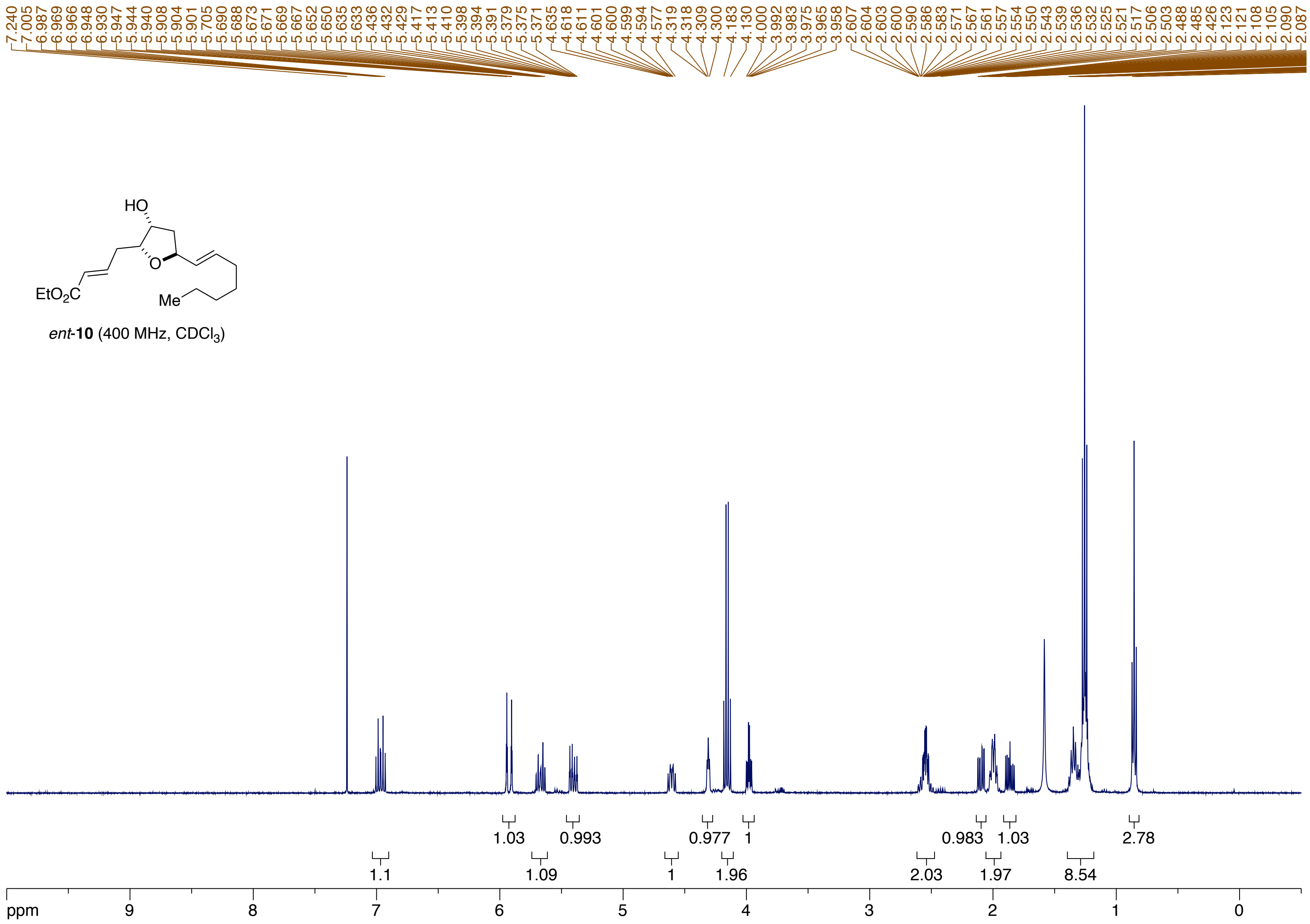


*ent*-**10** (400 MHz, $CDCl_3$)

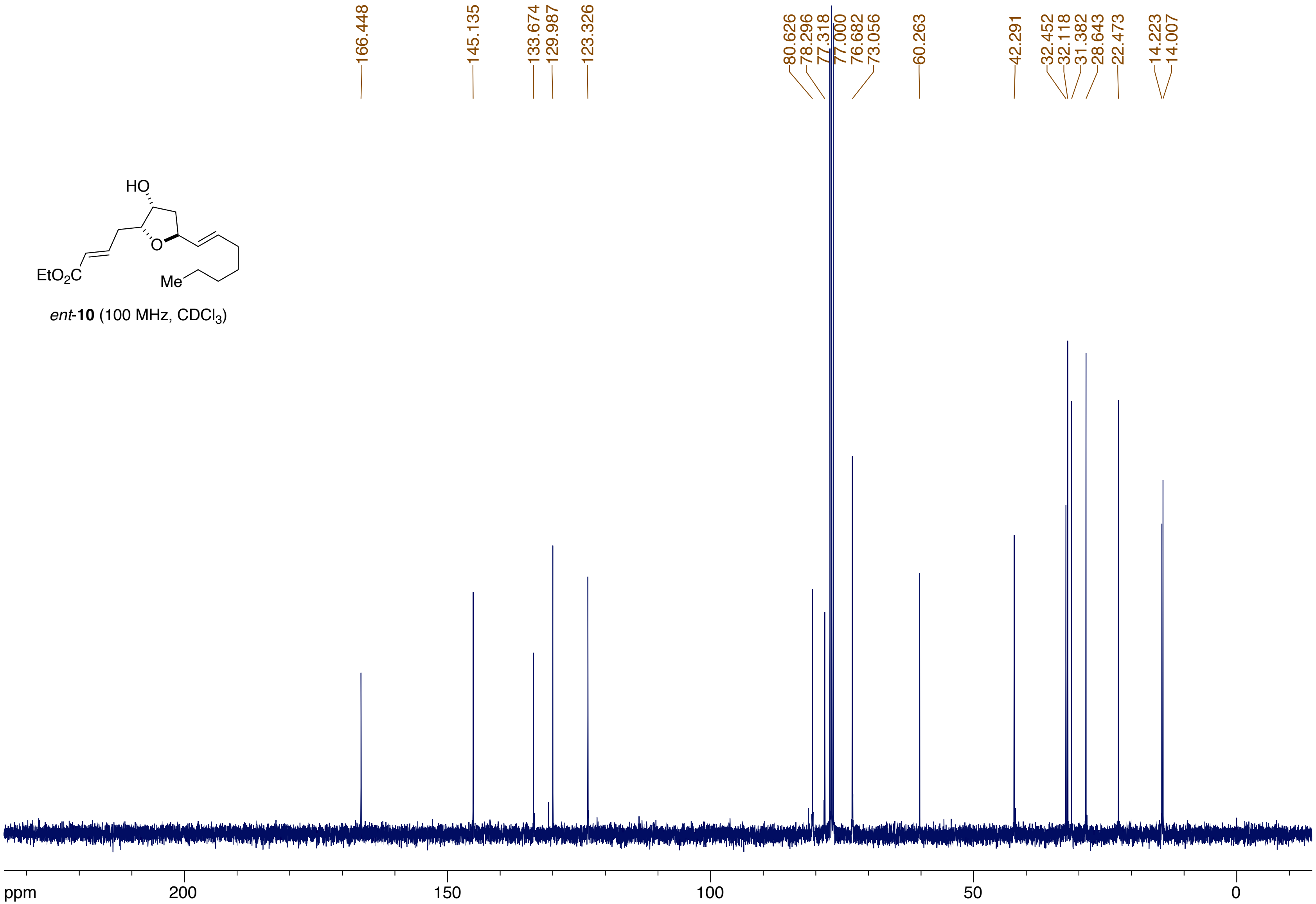


*ent*-**10** (100 MHz, $CDCl_3$)

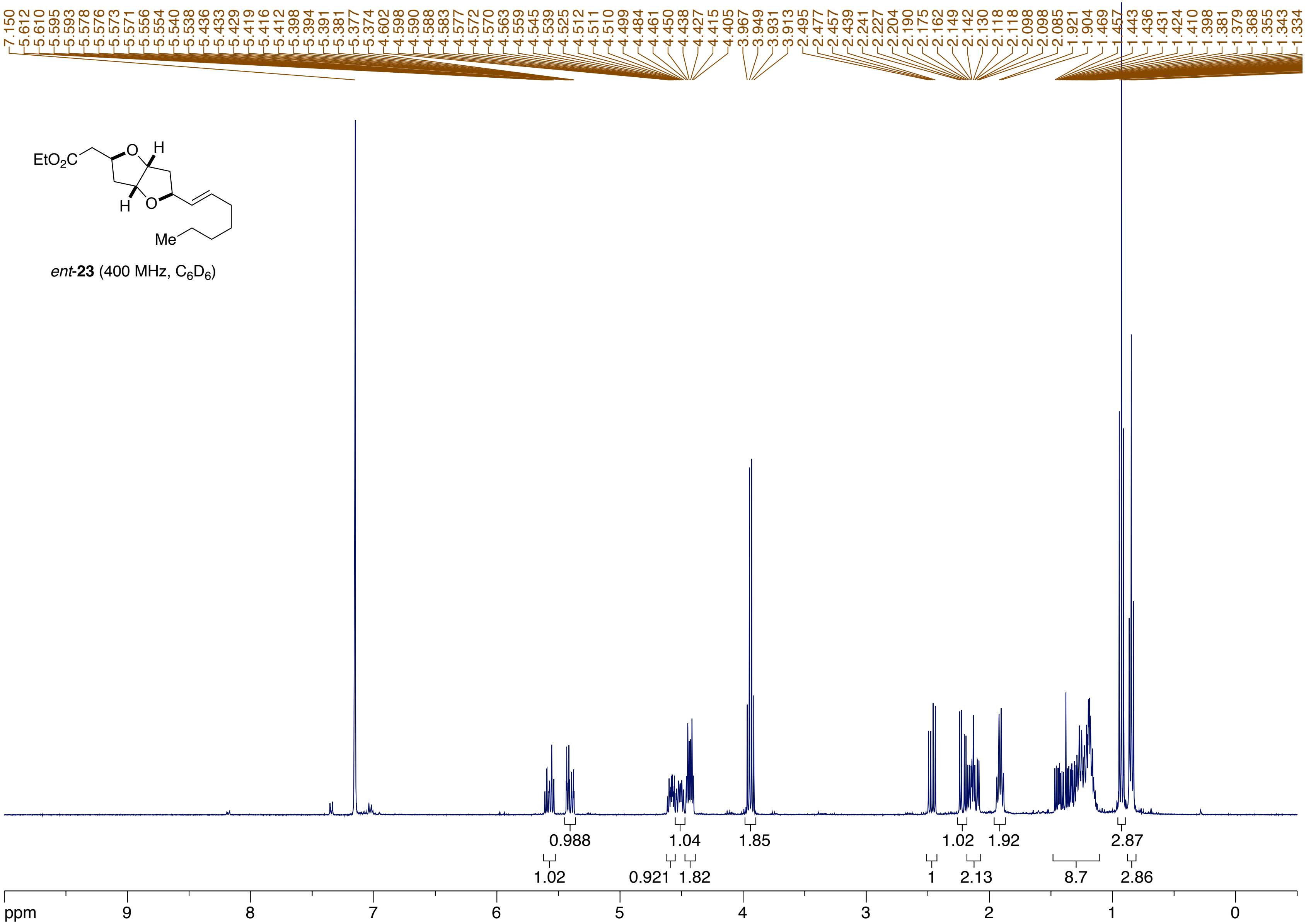
EtO2C
O
H
H
O
Me
*ent*-**23** (400 MHz, $C_6D_6$)
7.150
5.612
5.610
5.595
5.593
5.578
5.576
5.573
5.571
5.556
5.554
5.540
5.538
5.436
5.433
5.429
5.419
5.416
5.412
5.398
5.394
5.391
5.381
5.377
5.374
4.602
4.598
4.590
4.588
4.583
4.577
4.572
4.570
4.563
4.559
4.545
4.539
4.525
4.512
4.511
4.510
4.499
4.484
4.461
4.450
4.438
4.427
4.415
4.405
3.967
3.949
3.931
3.913
2.495
2.477
2.457
2.439
2.241
2.227
2.204
2.190
2.175
2.162
2.149
2.142
2.130
2.118
2.118
2.098
2.098
2.085
1.921
1.904
1.469
1.457
1.443
1.436
1.431
1.424
1.410
1.398
1.381
1.379
1.368
1.355
1.343
1.334
0.988
1.04
1.85
1.02
1.92
2.87
1.02
0.921
1.82
1
2.13
8.7
2.86
ppm
9
8
7
6
5
4
3
2
1
0

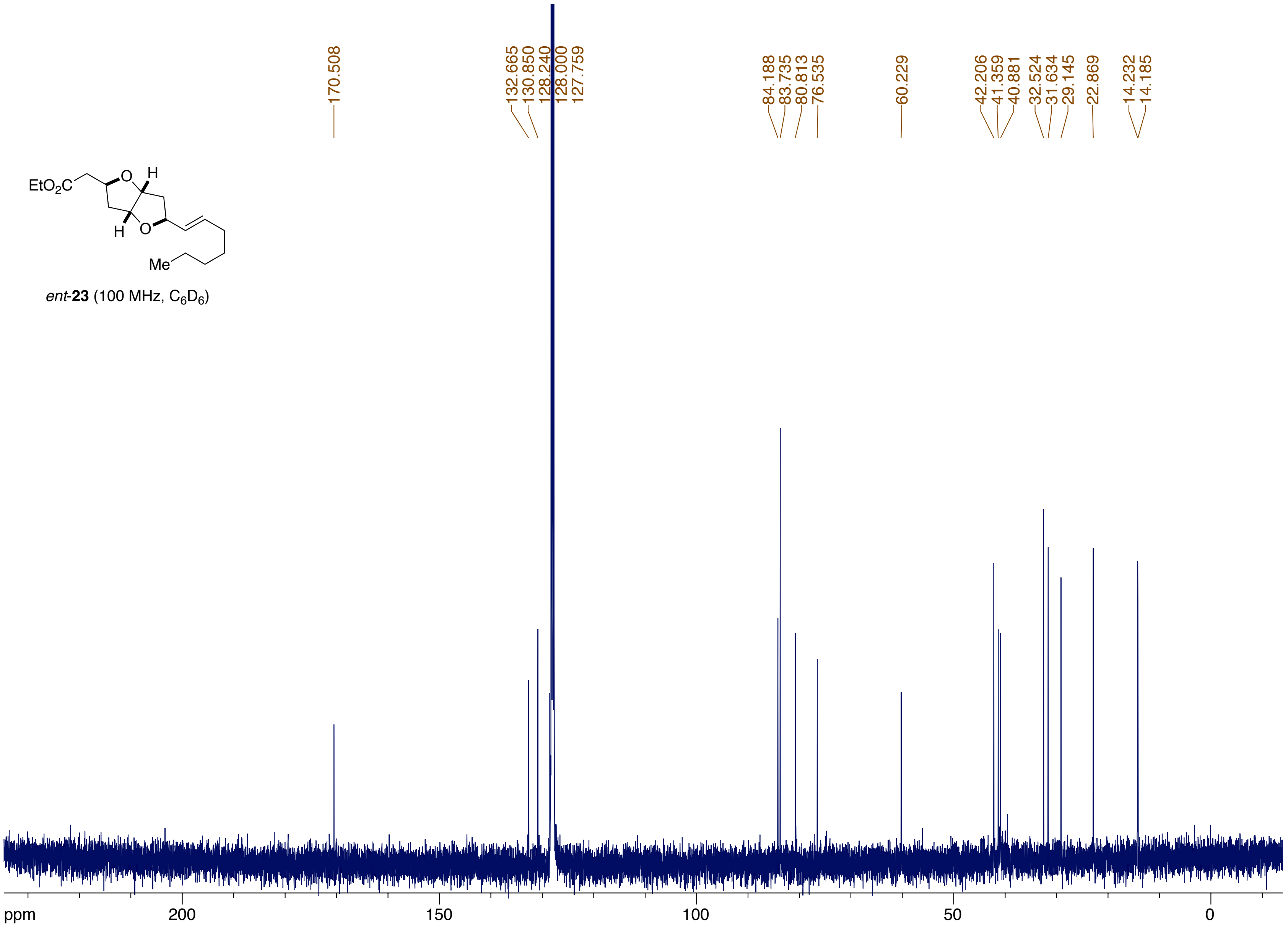


*ent*-**23** (100 MHz, $C_6D_6$)

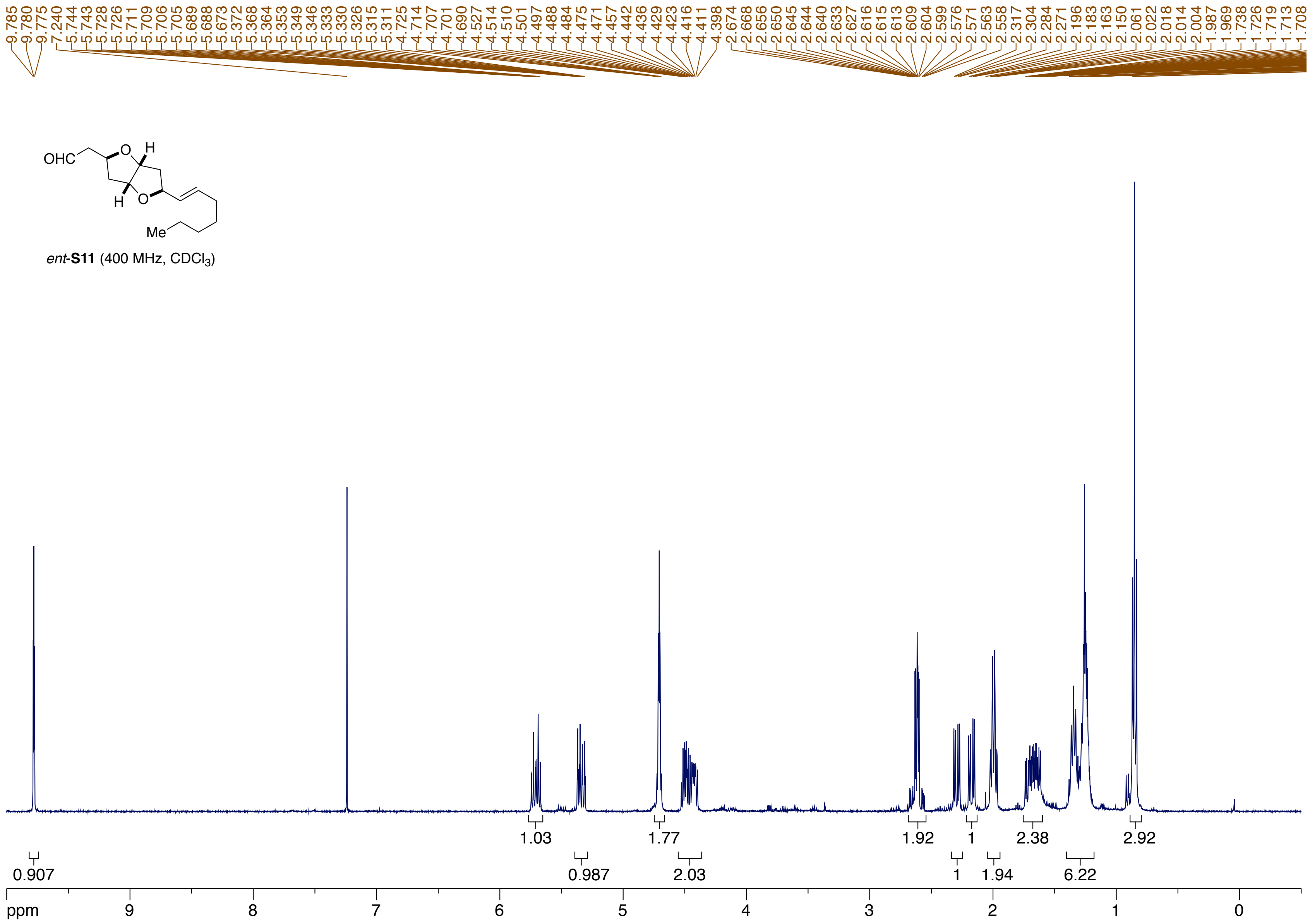
9.785 9.780 9.775 7.240 5.744 5.743 5.728 5.726 5.711 5.709 5.706 5.705 5.689 5.688 5.673 5.372 5.368 5.364 5.353 5.349 5.346 5.333 5.330 5.326 5.315 5.311 4.725 4.714 4.707 4.701 4.690 4.527 4.514 4.510 4.501 4.497 4.488 4.484 4.475 4.471 4.457 4.442 4.436 4.429 4.423 4.416 4.411 4.398 2.674 2.668 2.656 2.650 2.645 2.644 2.640 2.633 2.627 2.616 2.615 2.613 2.609 2.604 2.599 2.576 2.571 2.563 2.558 2.317 2.304 2.284 2.271 2.196 2.183 2.163 2.150 2.061 2.022 2.018 2.014 2.004 1.987 1.969 1.738 1.726 1.719 1.713 1.708
OHC
O
H
H
O
Me
*ent*-**S11** (400 MHz, $CDCl_3$)
1.03
1.77
1.92
1
2.38
2.92
0.907
0.987
2.03
1
1.94
6.22
ppm
9
8
7
6
5
4
3
2
1
0

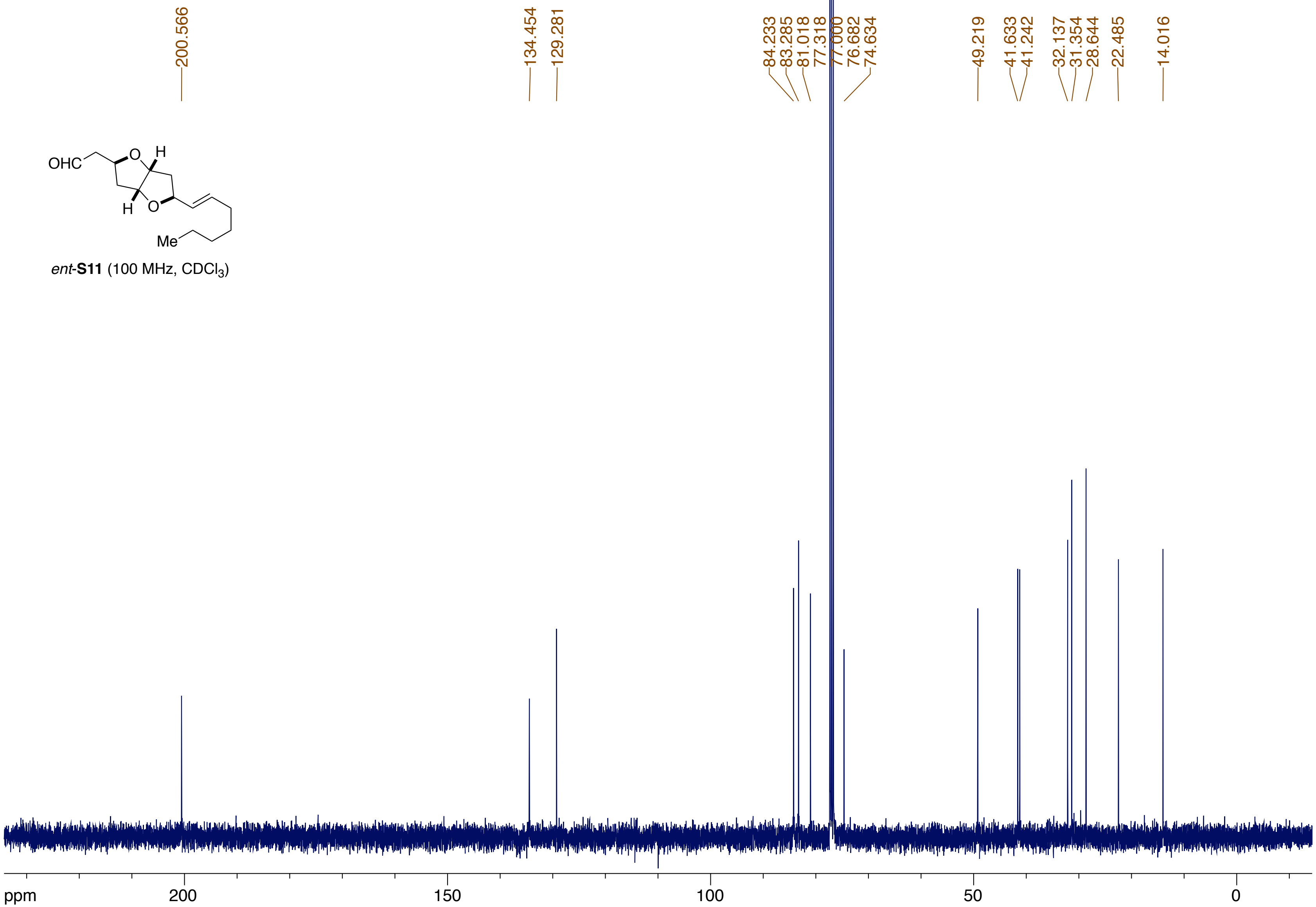


*ent*-**S11** (100 MHz, $CDCl_3$)

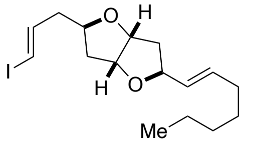

*ent*-**5** (400 MHz, $C_6D_6$)

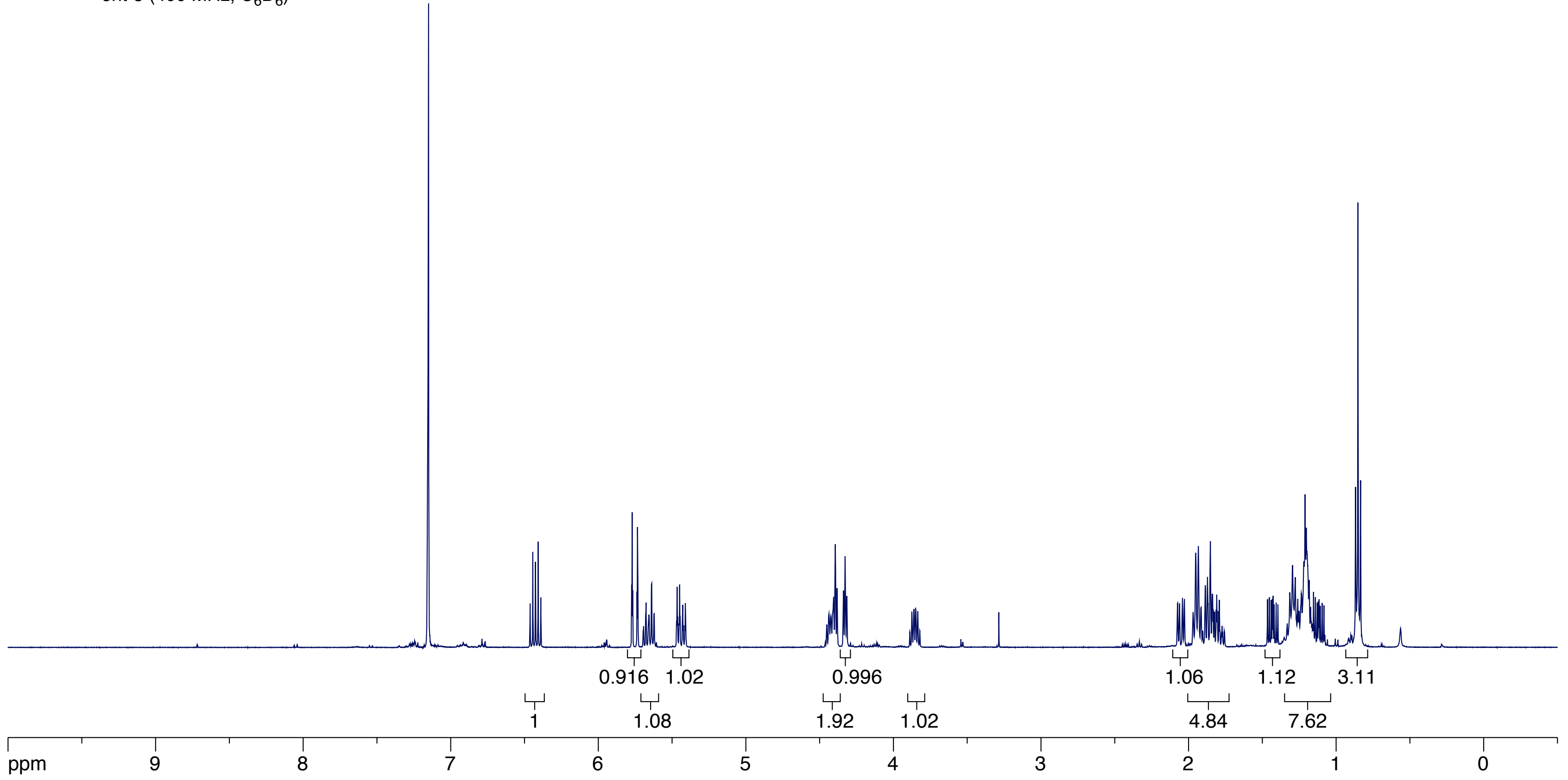

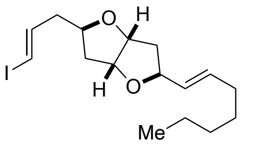


*ent*-**5** (100 MHz, $C_6D_6$)

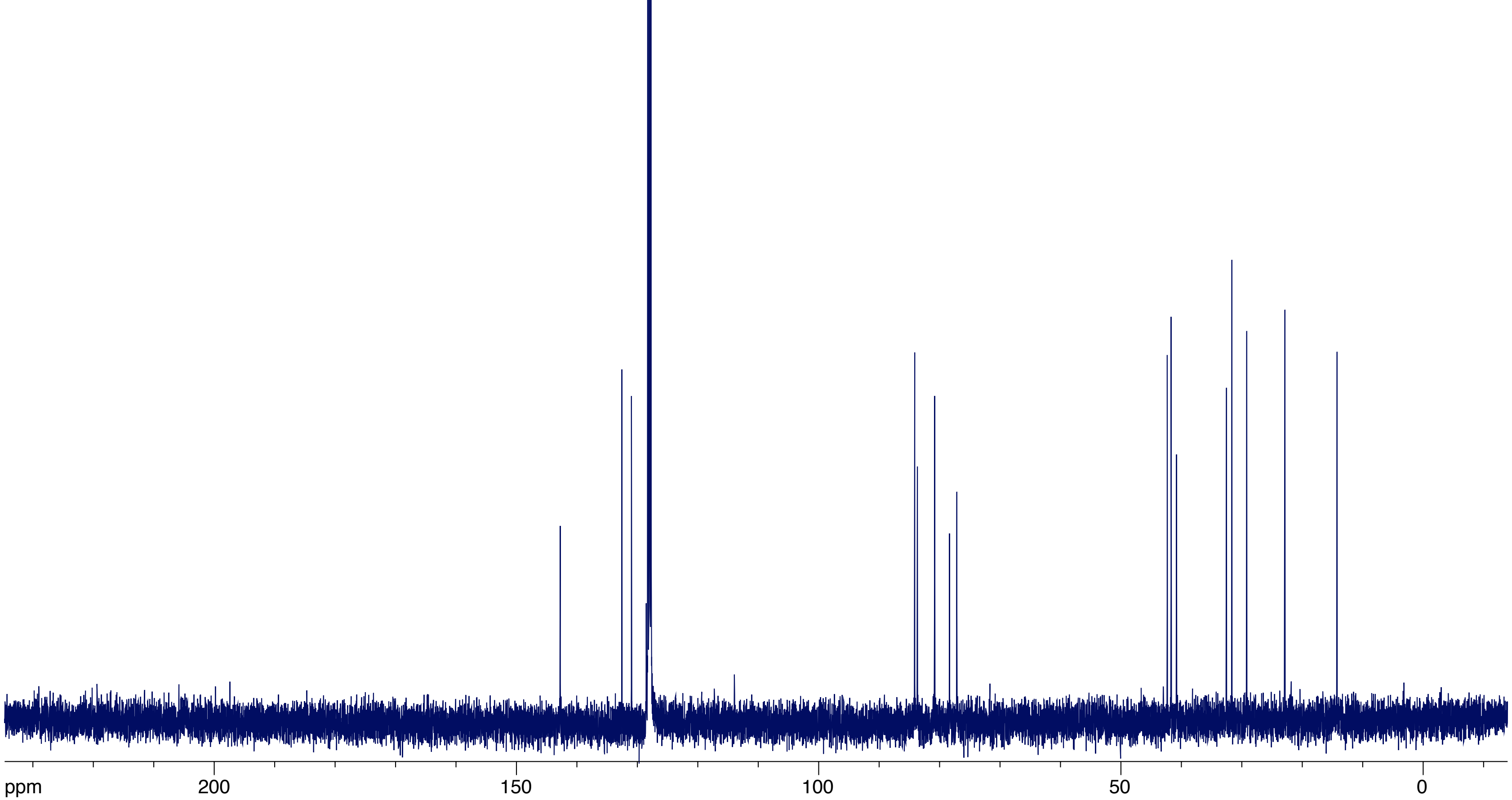

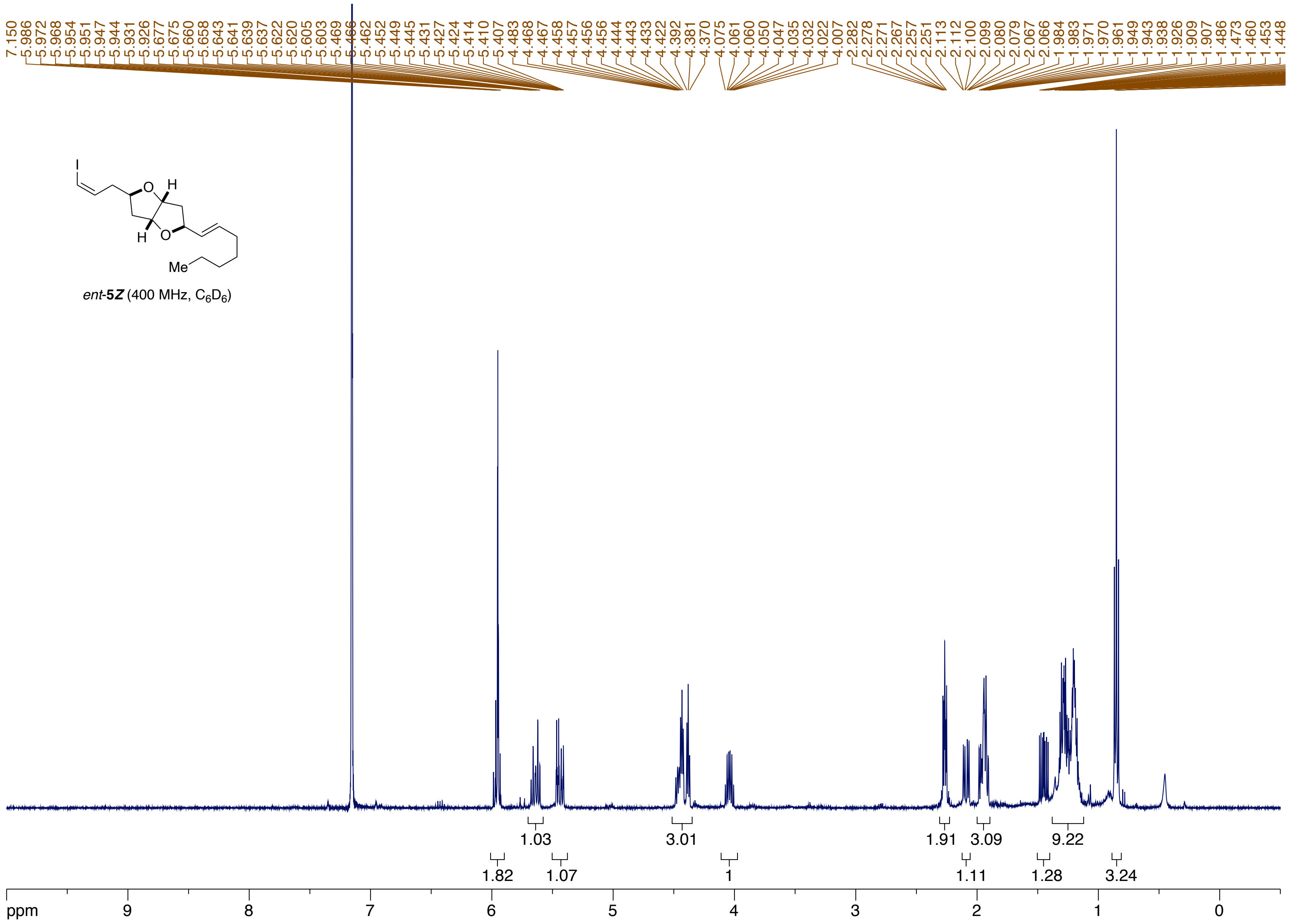

*ent*-**5Z** (400 MHz, $C_6D_6$)

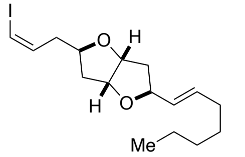

*ent*-**5*Z*** (100 MHz, $C_6D_6$)

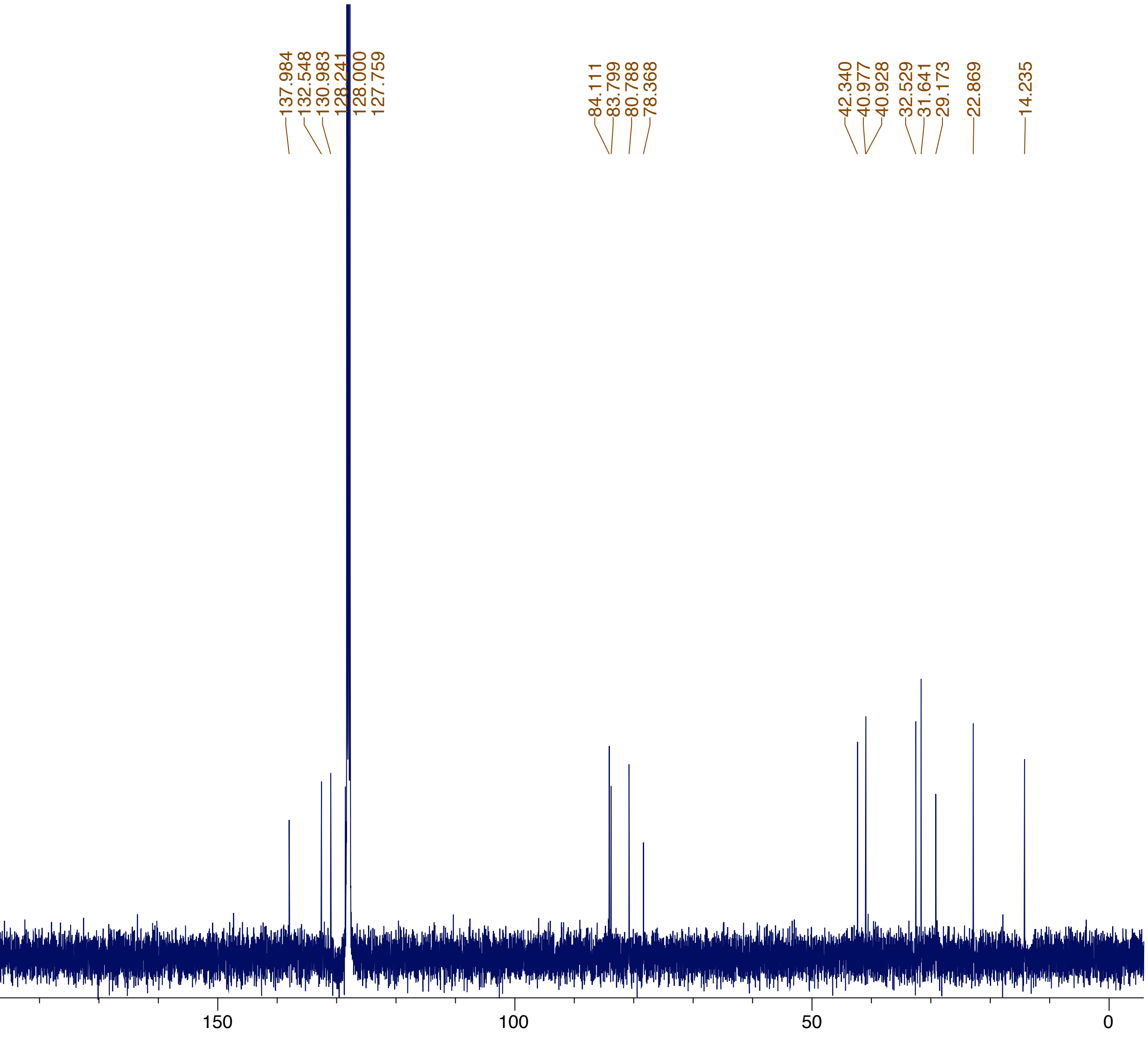

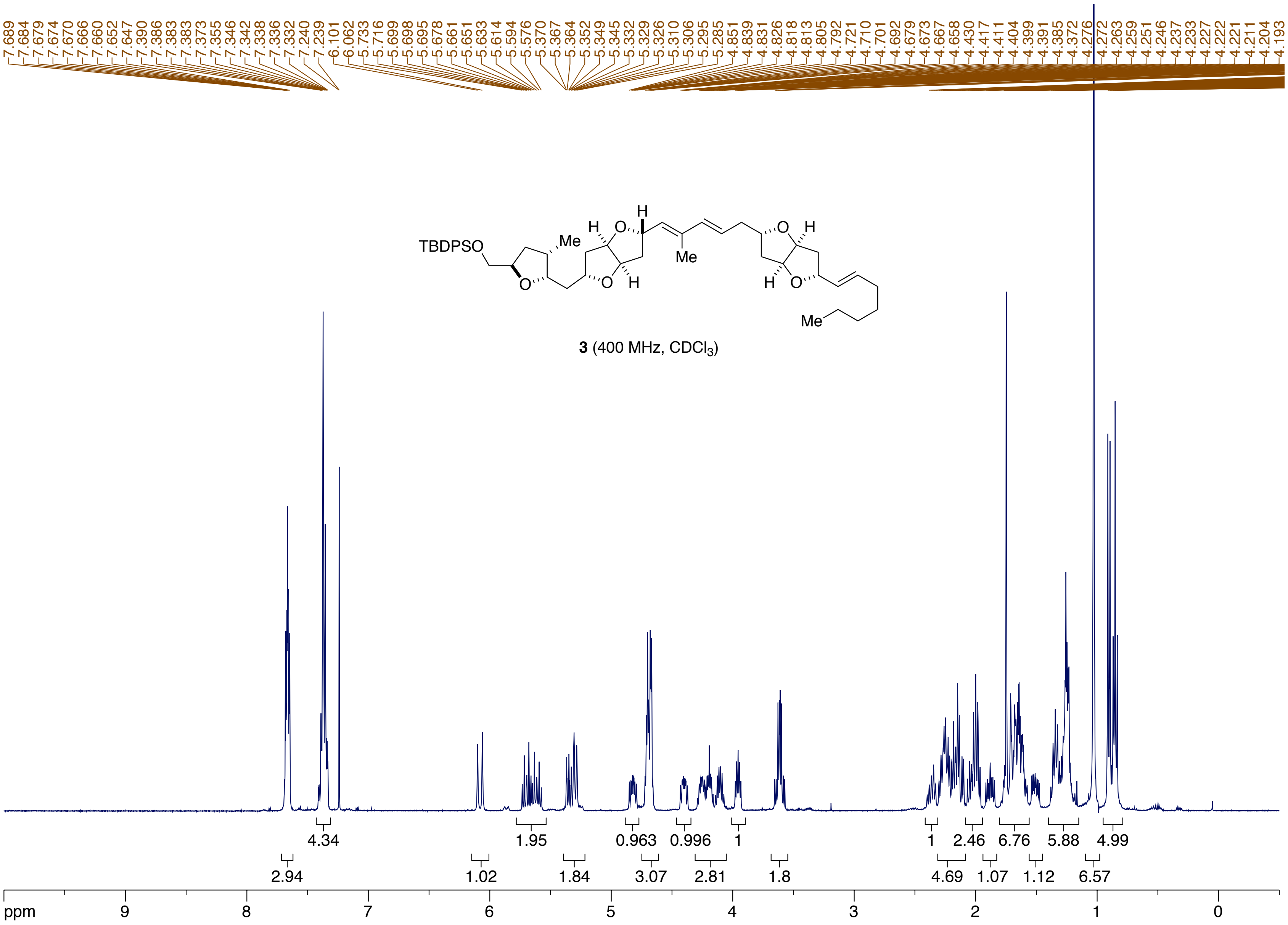

7.689 7.684 7.679 7.674 7.670 7.666 7.660 7.652 7.647 7.390 7.386 7.383 7.383 7.373 7.355 7.346 7.342 7.338 7.336 7.332 7.240 7.239 6.101 6.062 5.733 5.716 5.699 5.698 5.695 5.678 5.661 5.651 5.633 5.614 5.594 5.576 5.370 5.367 5.364 5.352 5.349 5.345 5.332 5.329 5.326 5.310 5.306 5.295 5.285 4.851 4.839 4.831 4.826 4.818 4.813 4.805 4.792 4.721 4.710 4.701 4.692 4.679 4.673 4.667 4.658 4.430 4.417 4.411 4.404 4.399 4.391 4.385 4.372 4.276 4.272 4.263 4.259 4.251 4.246 4.237 4.233 4.227 4.222 4.221 4.211 4.204 4.193
TBDPSO
Me
H
O
H
Me
O
H
H
O
H
O
H
Me
3 (400 MHz, CDCl$_3$)
4.34
1.95
0.963
0.996
1
1
2.46
6.76
5.88
4.99
2.94
1.02
1.84
3.07
2.81
1.8
4.69
1.07
1.12
6.57
ppm
9
8
7
6
5
4
3
2
1
0

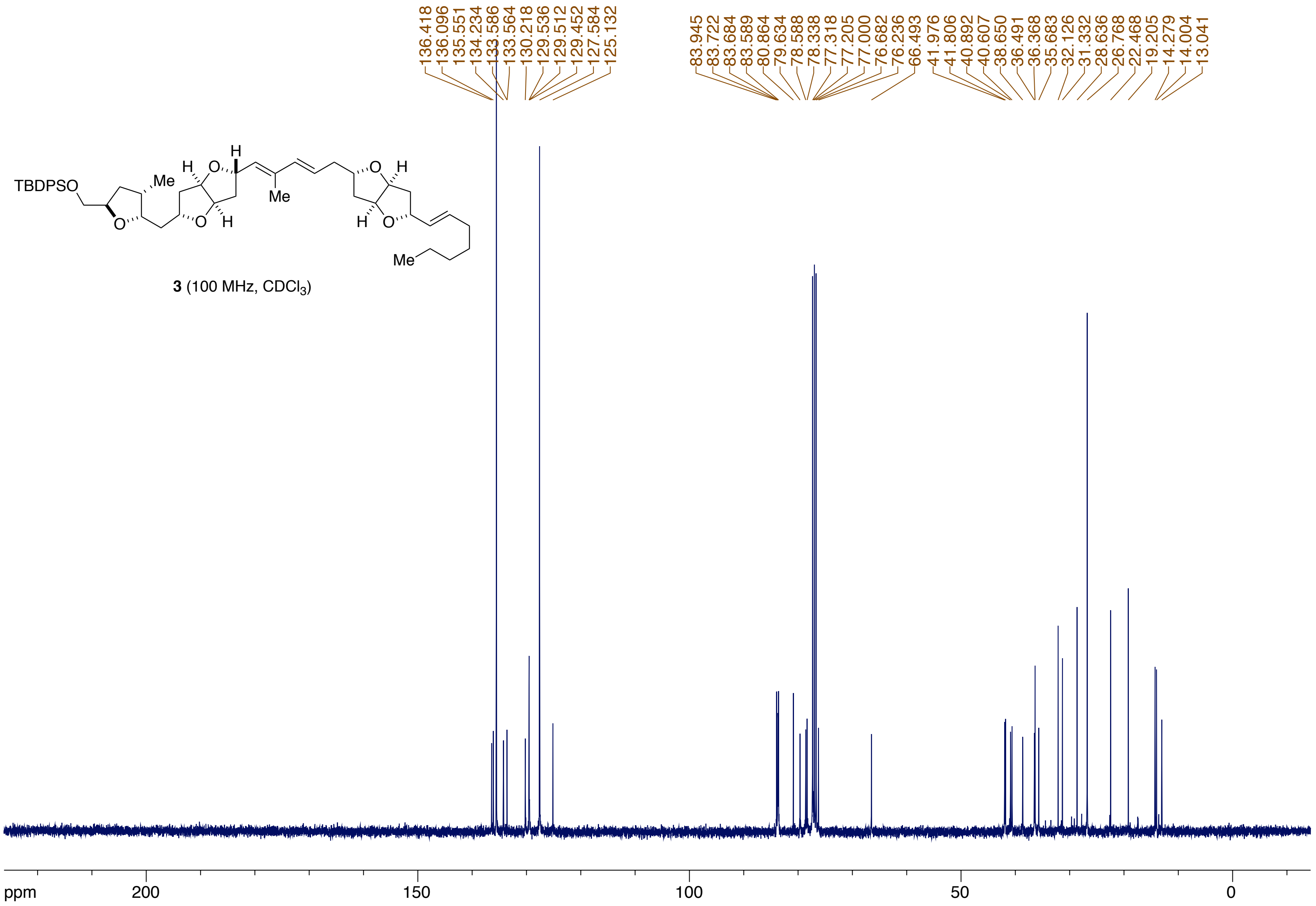


**3** (100 MHz, $CDCl_3$)

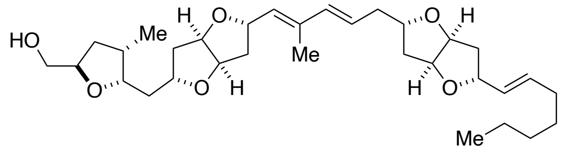


**24** (400 MHz, $CDCl_3$)

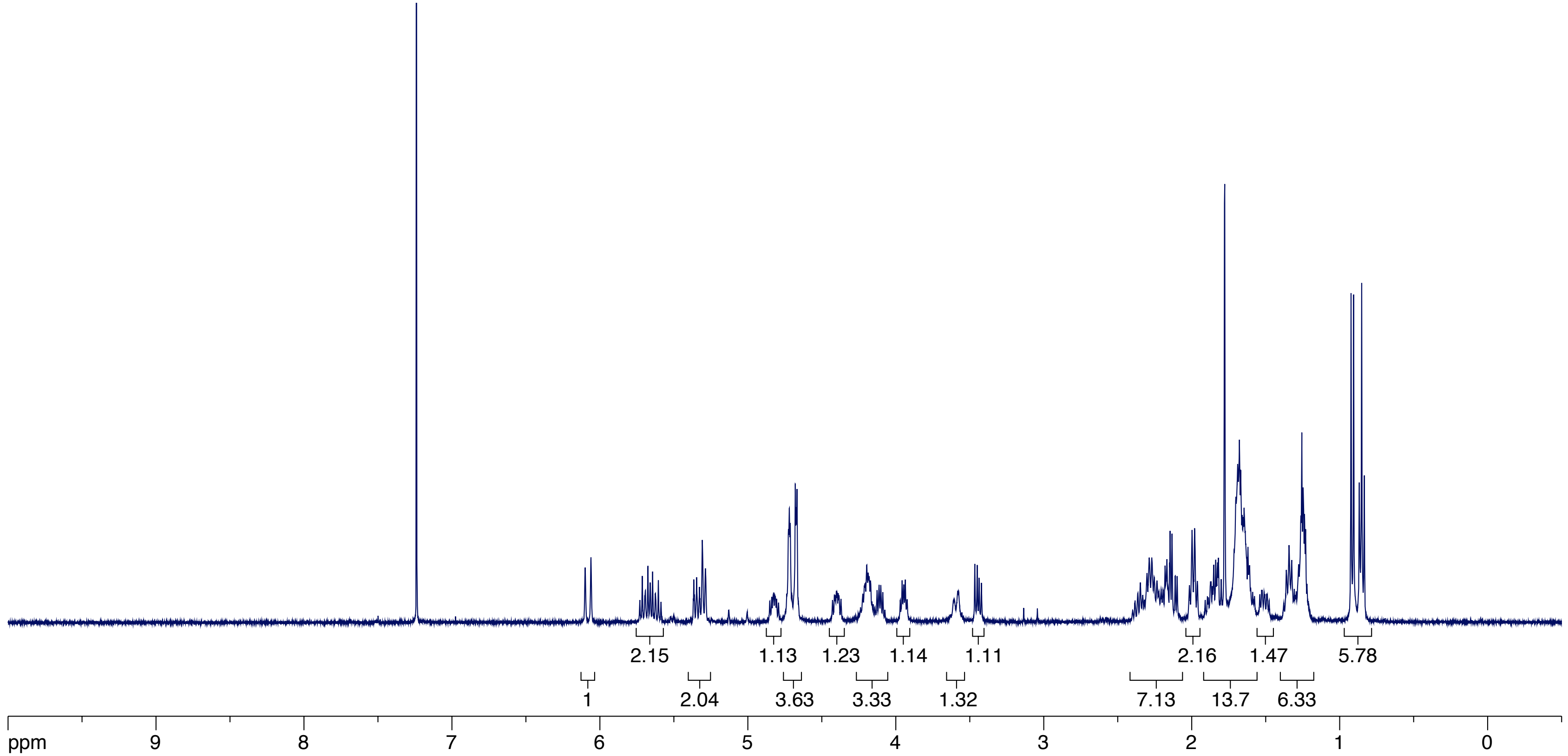

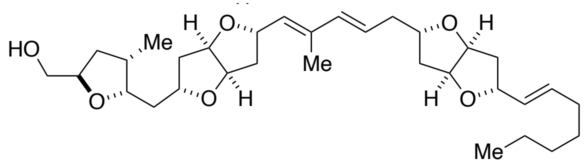

**24** (100 MHz, $CDCl_3$)

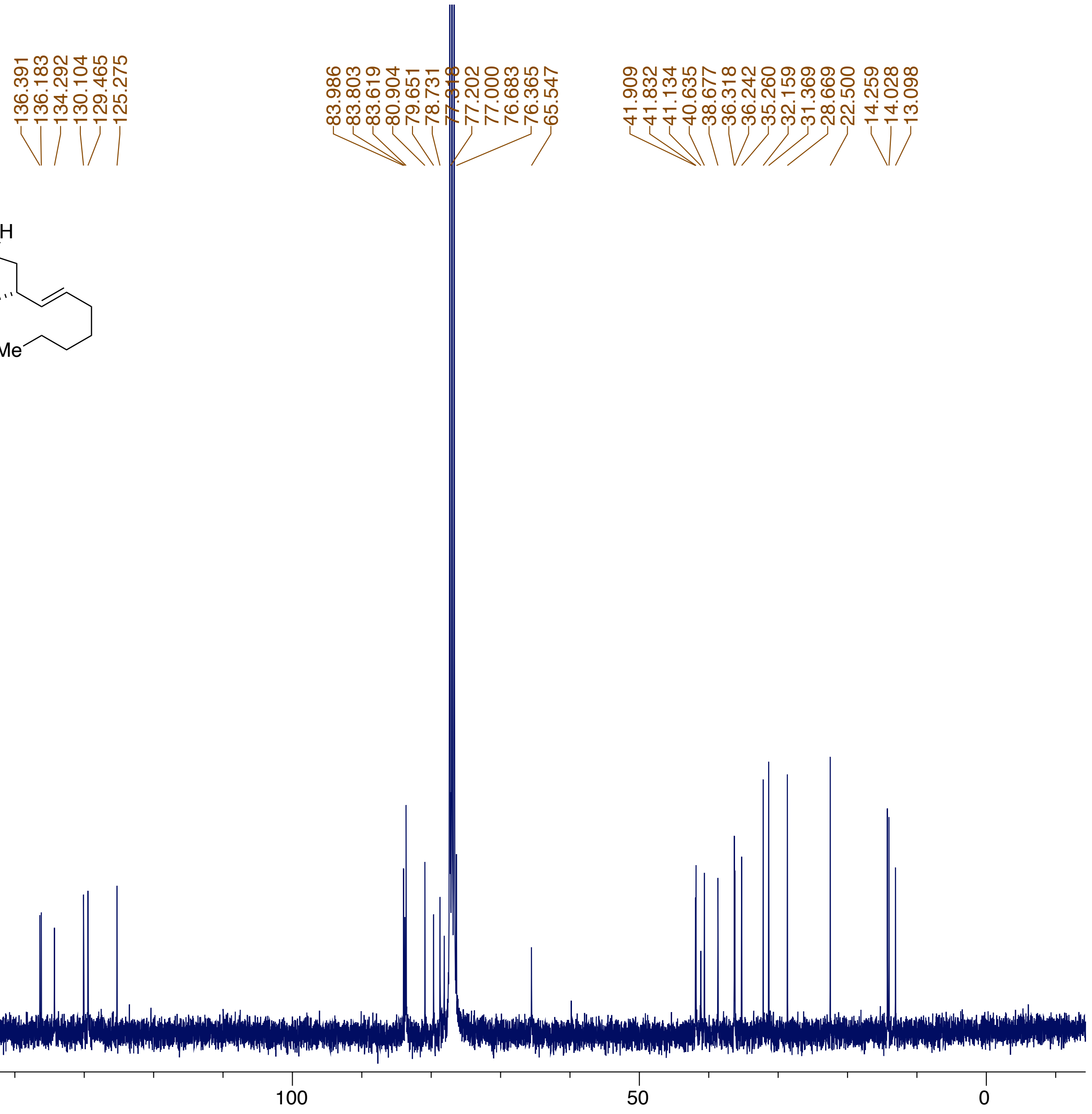

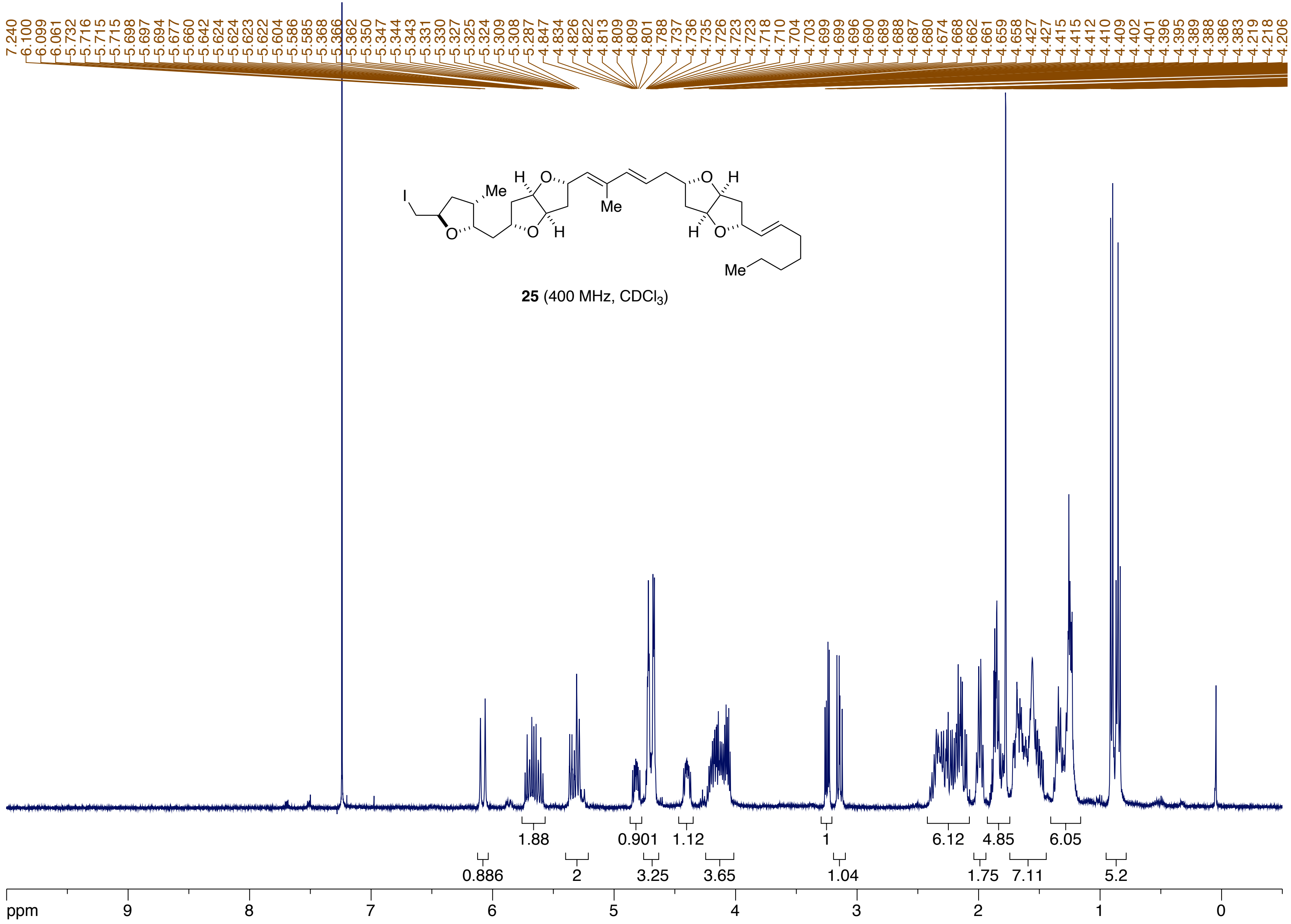

7.240 6.100 6.099 6.061 5.732 5.716 5.715 5.698 5.697 5.694 5.677 5.660 5.642 5.624 5.624 5.623 5.622 5.604 5.586 5.585 5.368 5.366 5.362 5.350 5.347 5.344 5.343 5.331 5.330 5.327 5.325 5.324 5.309 5.308 5.287 4.847 4.834 4.826 4.822 4.813 4.809 4.809 4.801 4.788 4.737 4.736 4.735 4.726 4.723 4.723 4.718 4.710 4.704 4.703 4.699 4.699 4.696 4.690 4.689 4.688 4.687 4.680 4.674 4.668 4.662 4.661 4.659 4.658 4.427 4.427 4.415 4.412 4.410 4.409 4.402 4.401 4.396 4.395 4.389 4.388 4.386 4.383 4.219 4.218 4.206
I
Me
H
O
O
H
Me
O
H
H
O
Me
25 (400 MHz, CDCl3)
1.88
0.901
1.12
1
6.12
4.85
6.05
0.886
2
3.25
3.65
1.04
1.75
7.11
5.2
ppm
9
8
7
6
5
4
3
2
1
0

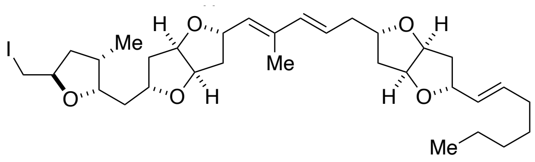

**25** (100 MHz, CDCl$_3$)

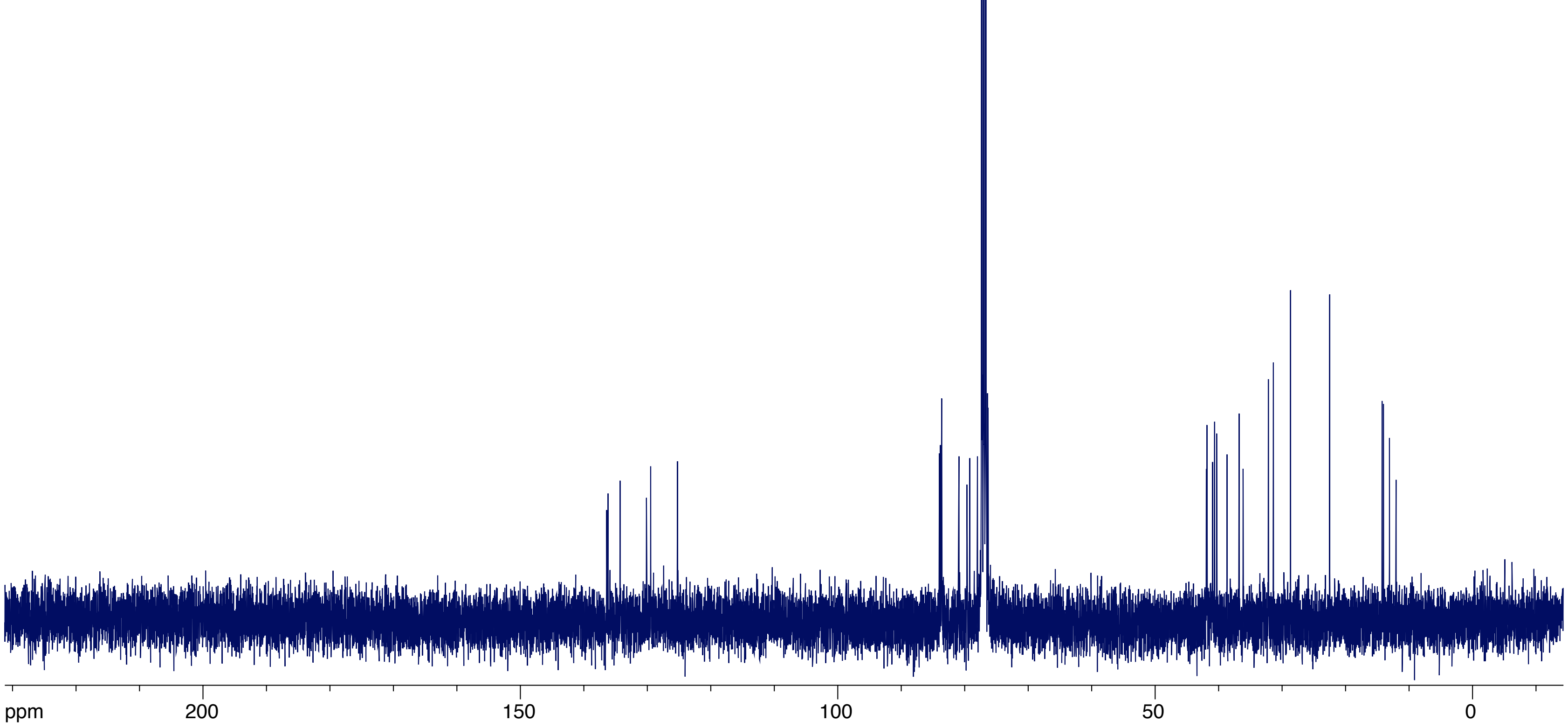

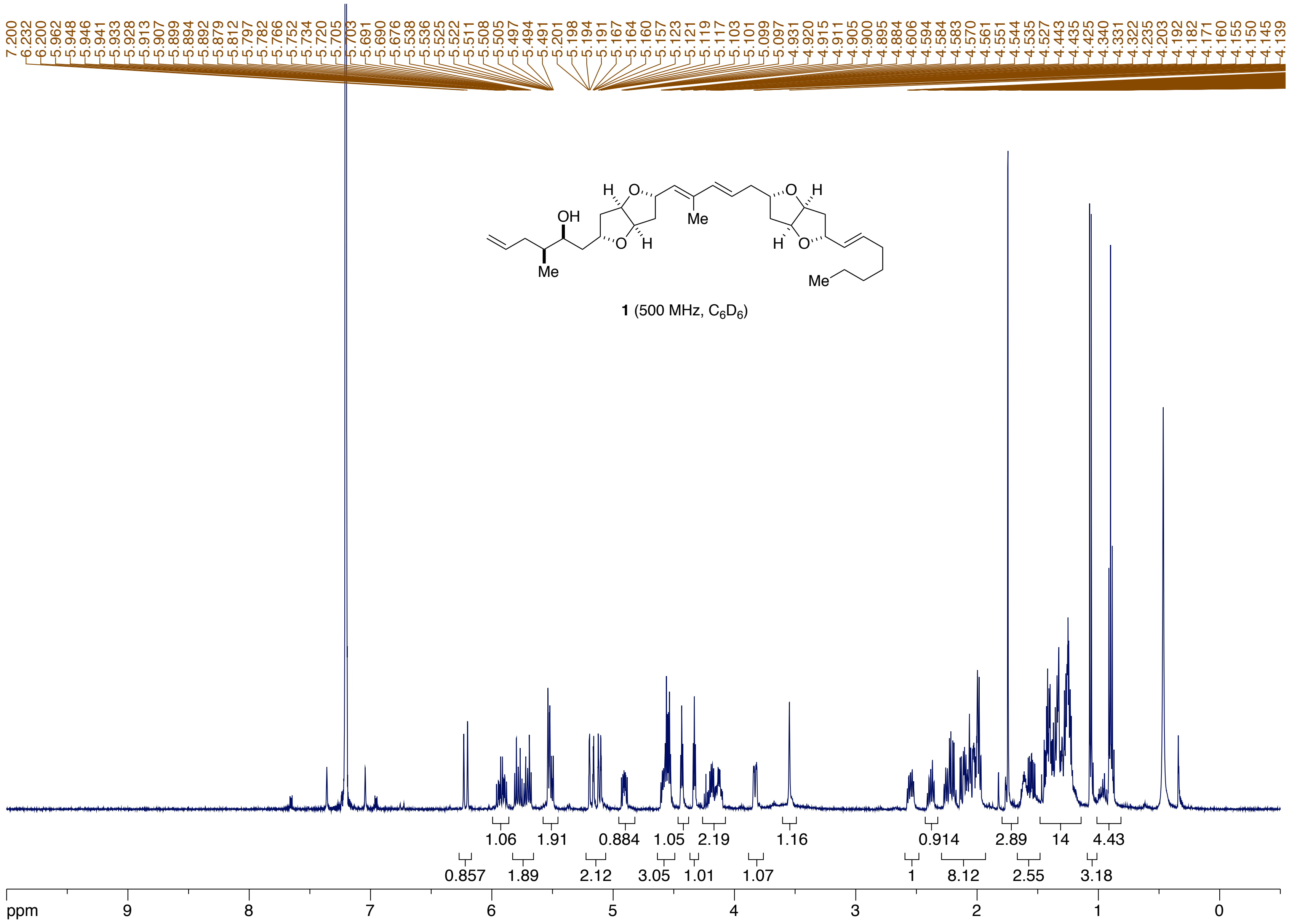
1 (500 MHz, C6D6)
ppm

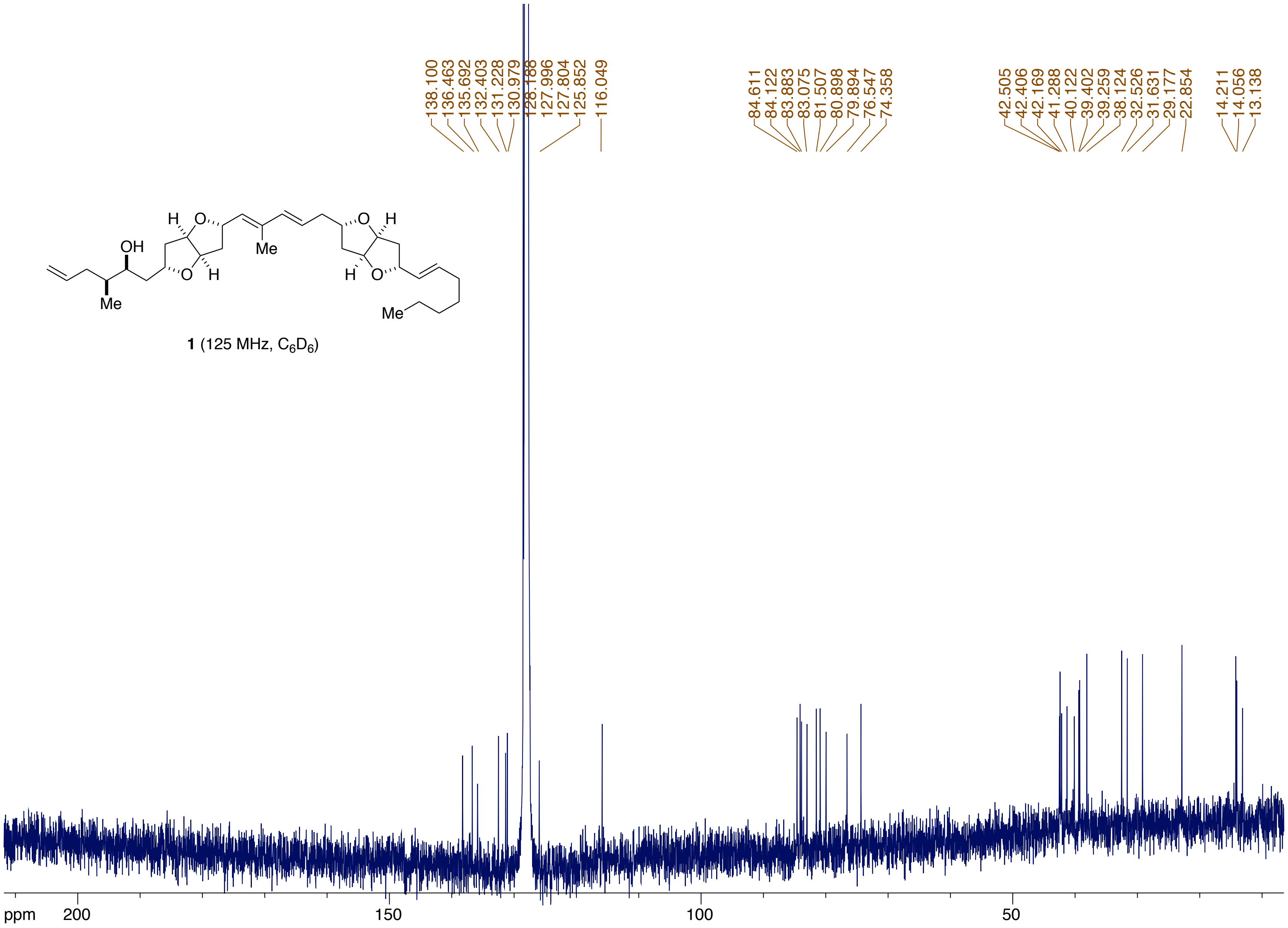
138.100
136.463
135.692
132.403
131.228
130.979
128.188
127.996
127.804
125.852
116.049
84.611
84.122
83.883
83.075
81.507
80.898
79.894
76.547
74.358
42.505
42.406
42.169
41.288
40.122
39.402
39.259
38.124
32.526
31.631
29.177
22.854
14.211
14.056
13.138
OH
Me
H
O
H
Me
O
H
H
O
Me
1 (125 MHz, $C_6D_6$)
ppm
200
150
100
50

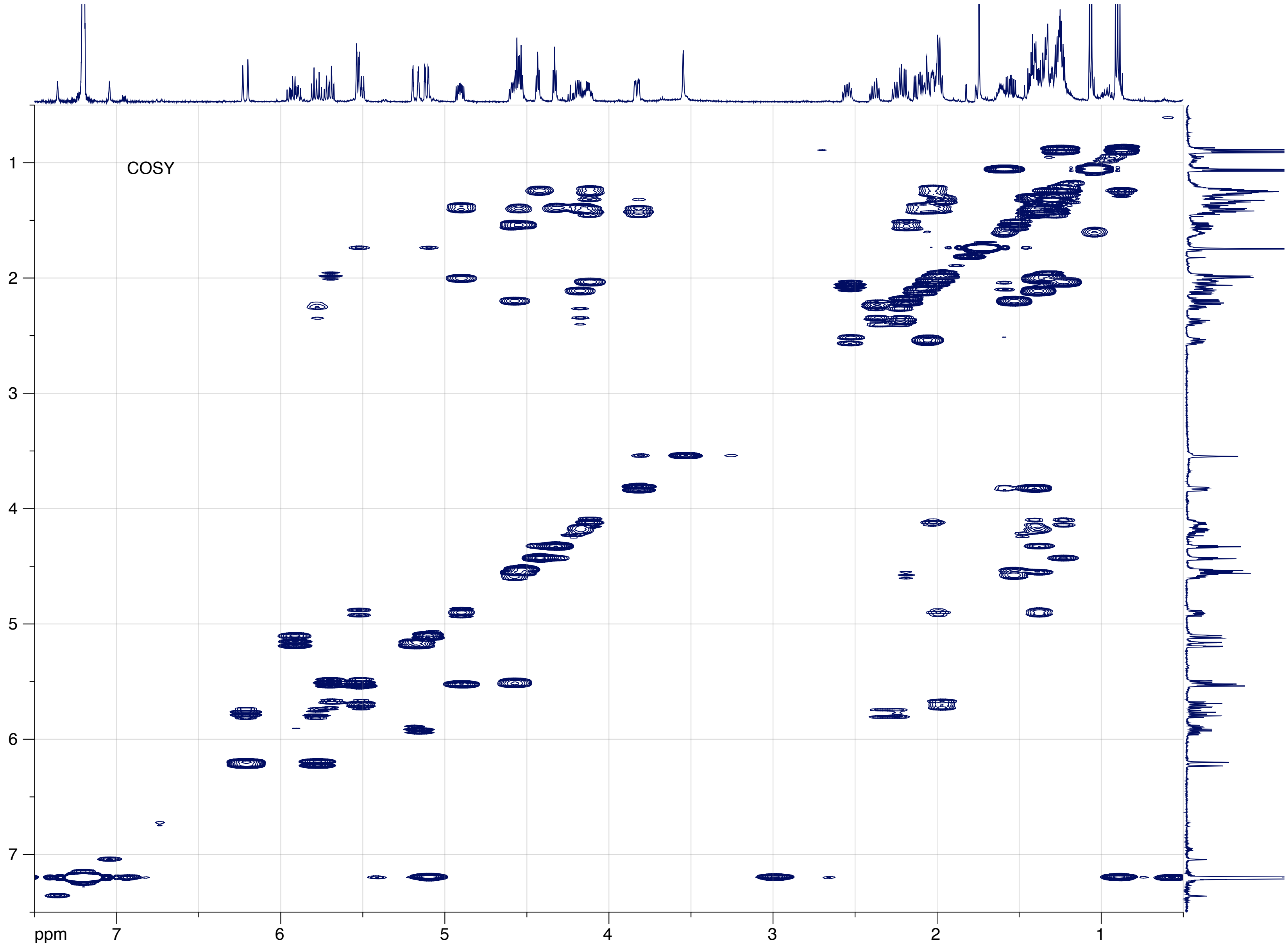
COSY
ppm
7
6
5
4
3
2
1

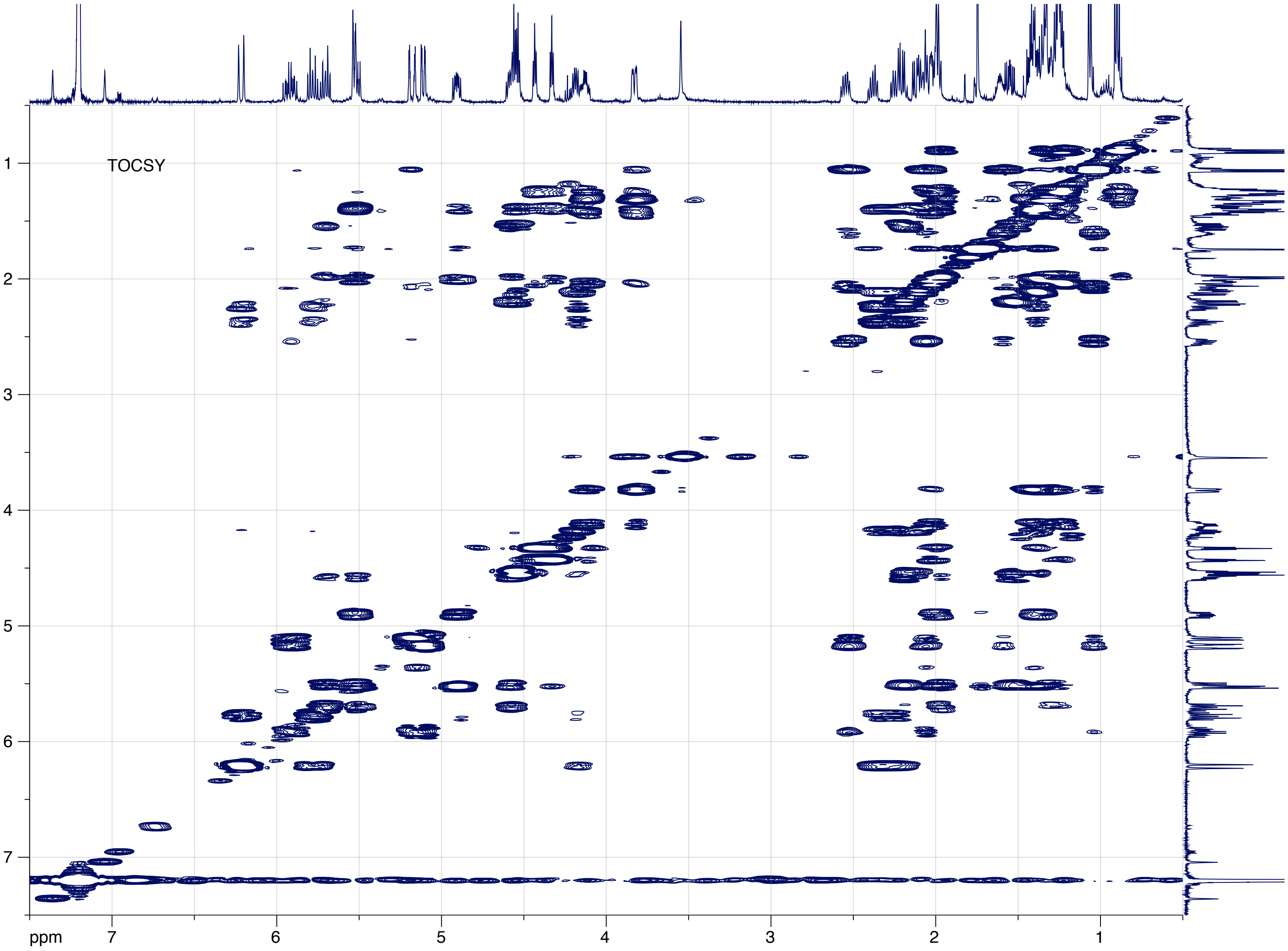
TOCSY
1
2
3
4
5
6
7
ppm
7
6
5
4
3
2
1

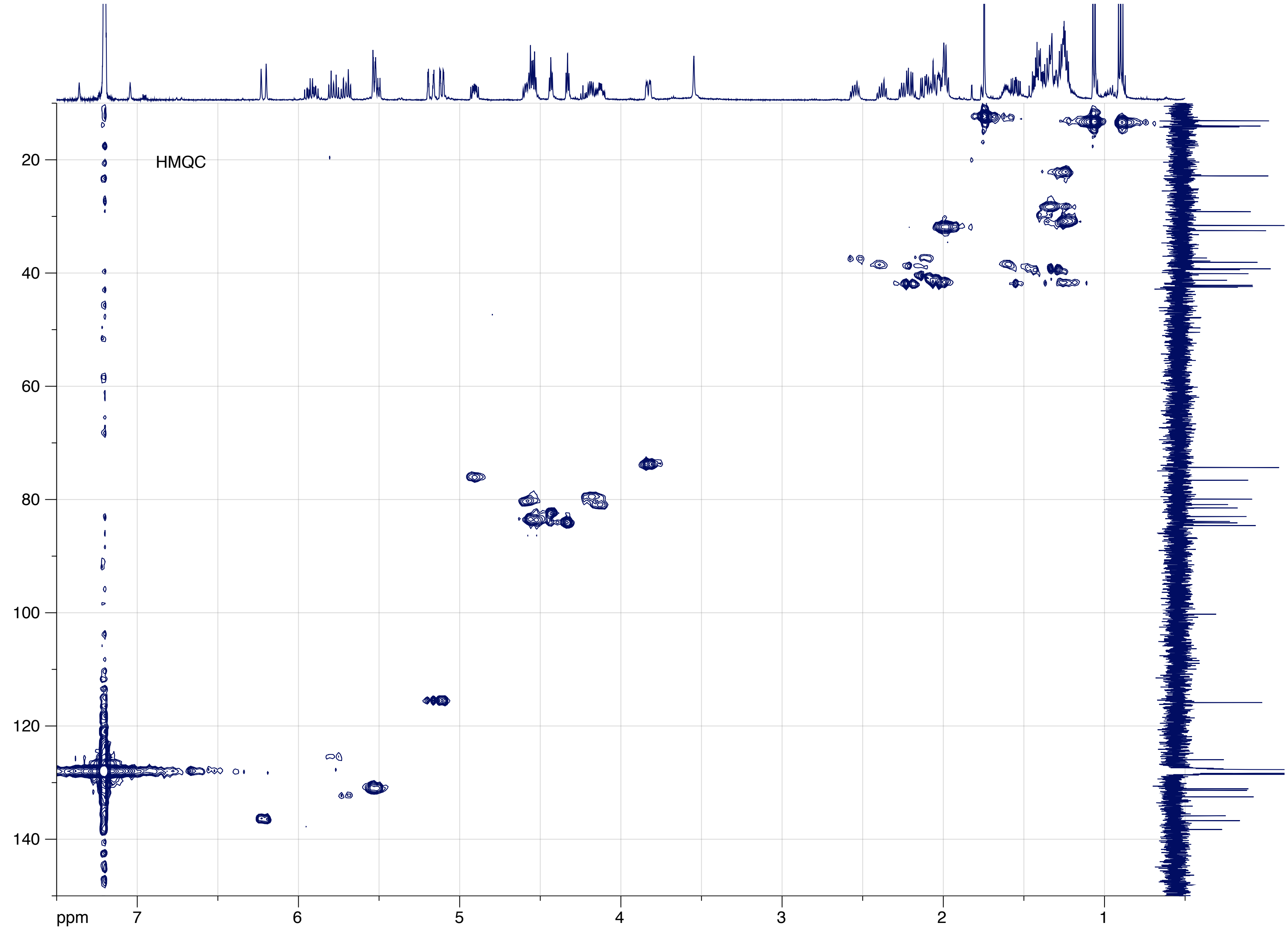
HMQC
ppm
20
40
60
80
100
120
140
7
6
5
4
3
2
1

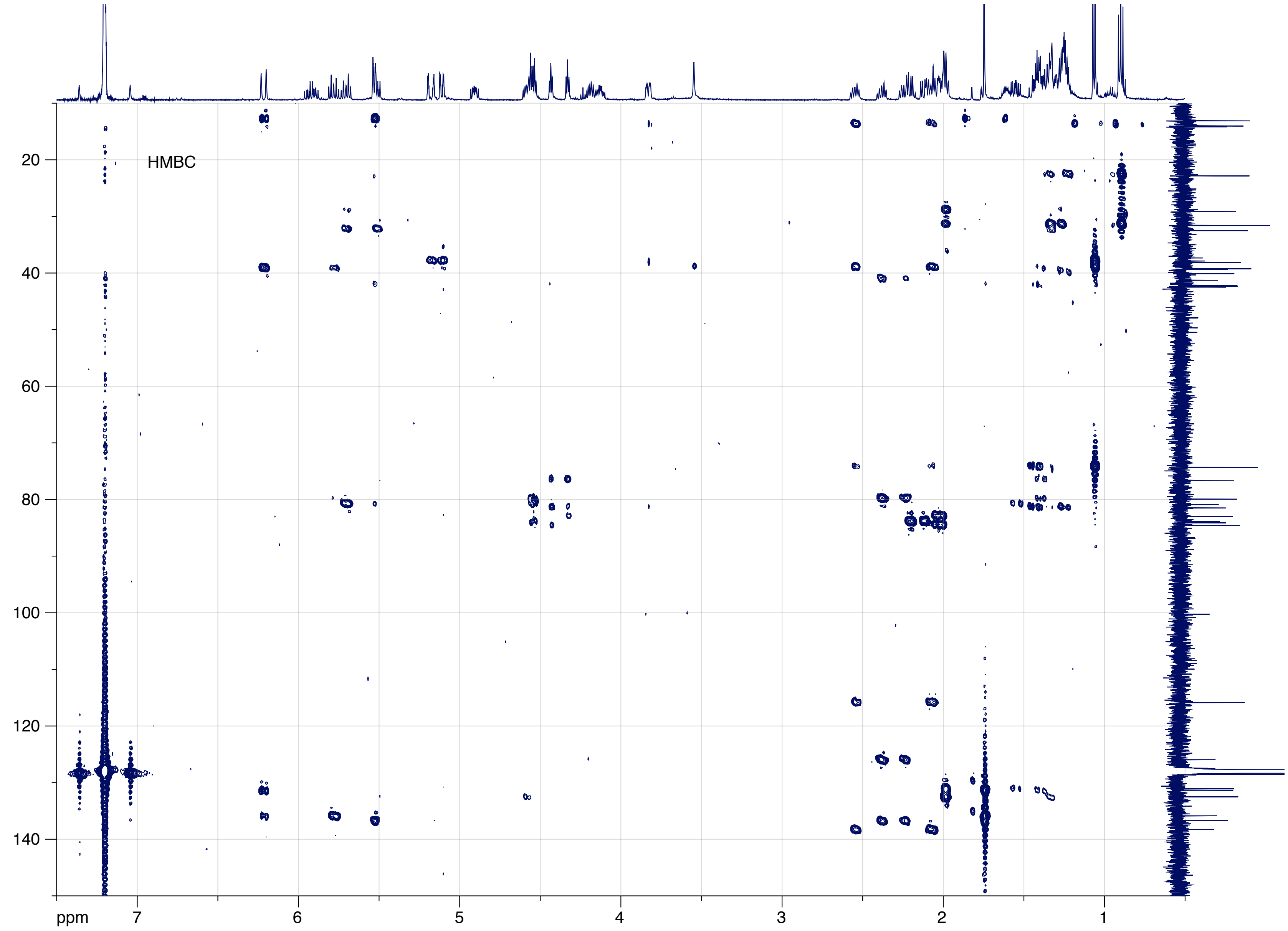
HMBC
ppm
7
6
5
4
3
2
1
20
40
60
80
100
120
140

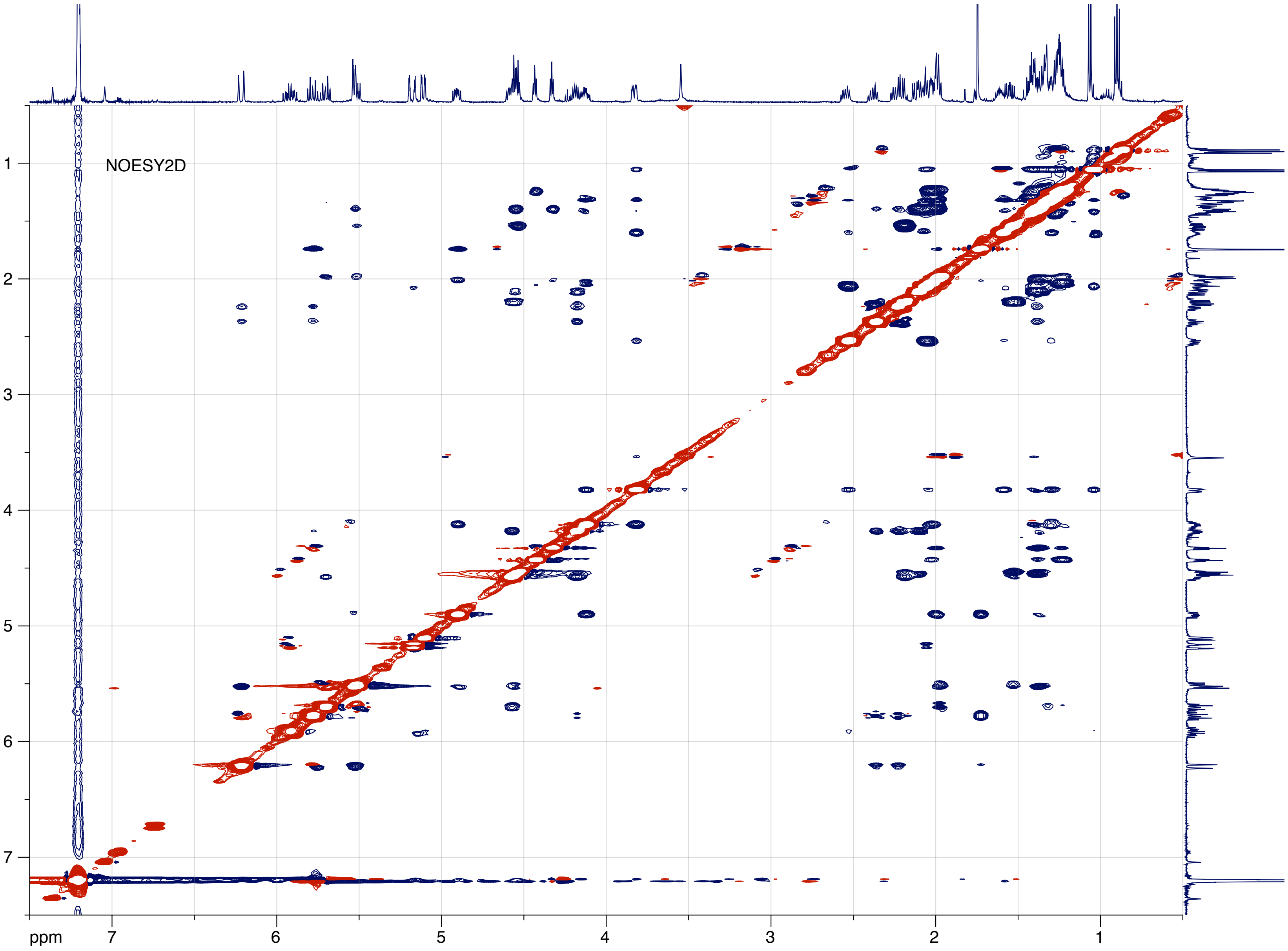
NOESY2D
ppm
7
6
5
4
3
2
1

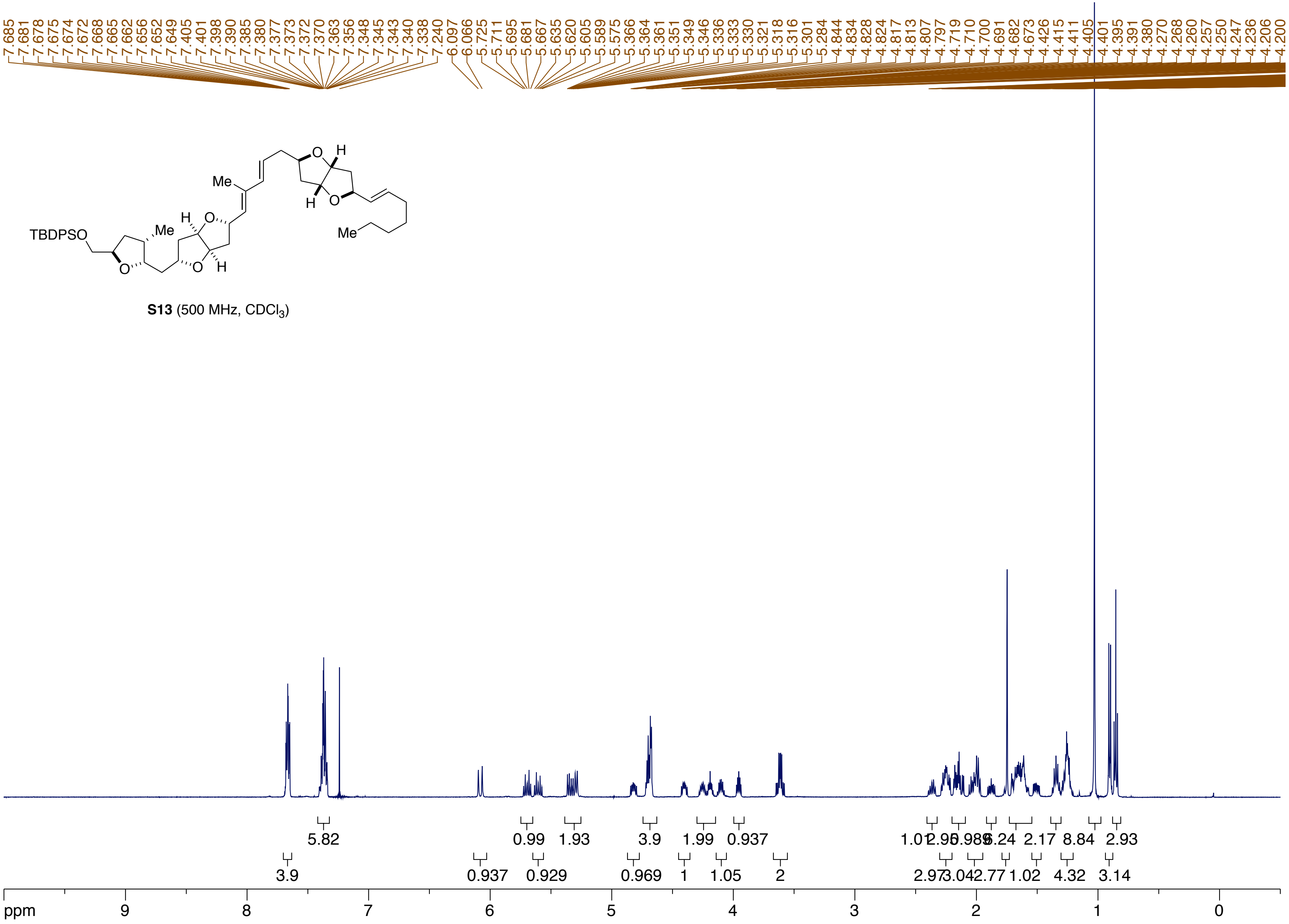
7.685 7.681 7.678 7.675 7.674 7.672 7.668 7.665 7.662 7.656 7.652 7.649 7.405 7.401 7.398 7.390 7.385 7.380 7.377 7.373 7.372 7.370 7.363 7.356 7.348 7.345 7.343 7.340 7.338 7.240 6.097 6.066 5.725 5.711 5.695 5.681 5.667 5.635 5.620 5.605 5.589 5.575 5.366 5.364 5.361 5.351 5.349 5.346 5.336 5.333 5.330 5.321 5.318 5.316 5.301 5.284 4.844 4.834 4.828 4.824 4.817 4.813 4.807 4.797 4.719 4.710 4.700 4.691 4.682 4.673 4.426 4.415 4.411 4.405 4.401 4.395 4.391 4.380 4.270 4.268 4.260 4.257 4.250 4.247 4.236 4.206 4.200
TBDPSO
Me
Me
Me
H
H
H
H
O
**S13** (500 MHz, $CDCl_3$)
5.82 0.99 1.93 3.9 1.99 0.937 1.01 2.96 0.98 6.24 2.17 8.84 2.93
3.9 0.937 0.929 0.969 1 1.05 2 2.97 3.04 2.77 1.02 4.32 3.14
ppm 9 8 7 6 5 4 3 2 1 0

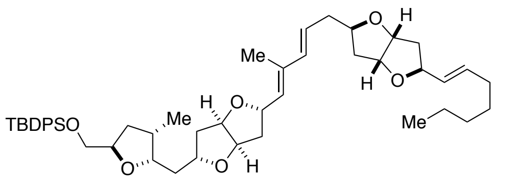


**S13** (125 MHz, $CDCl_3$)

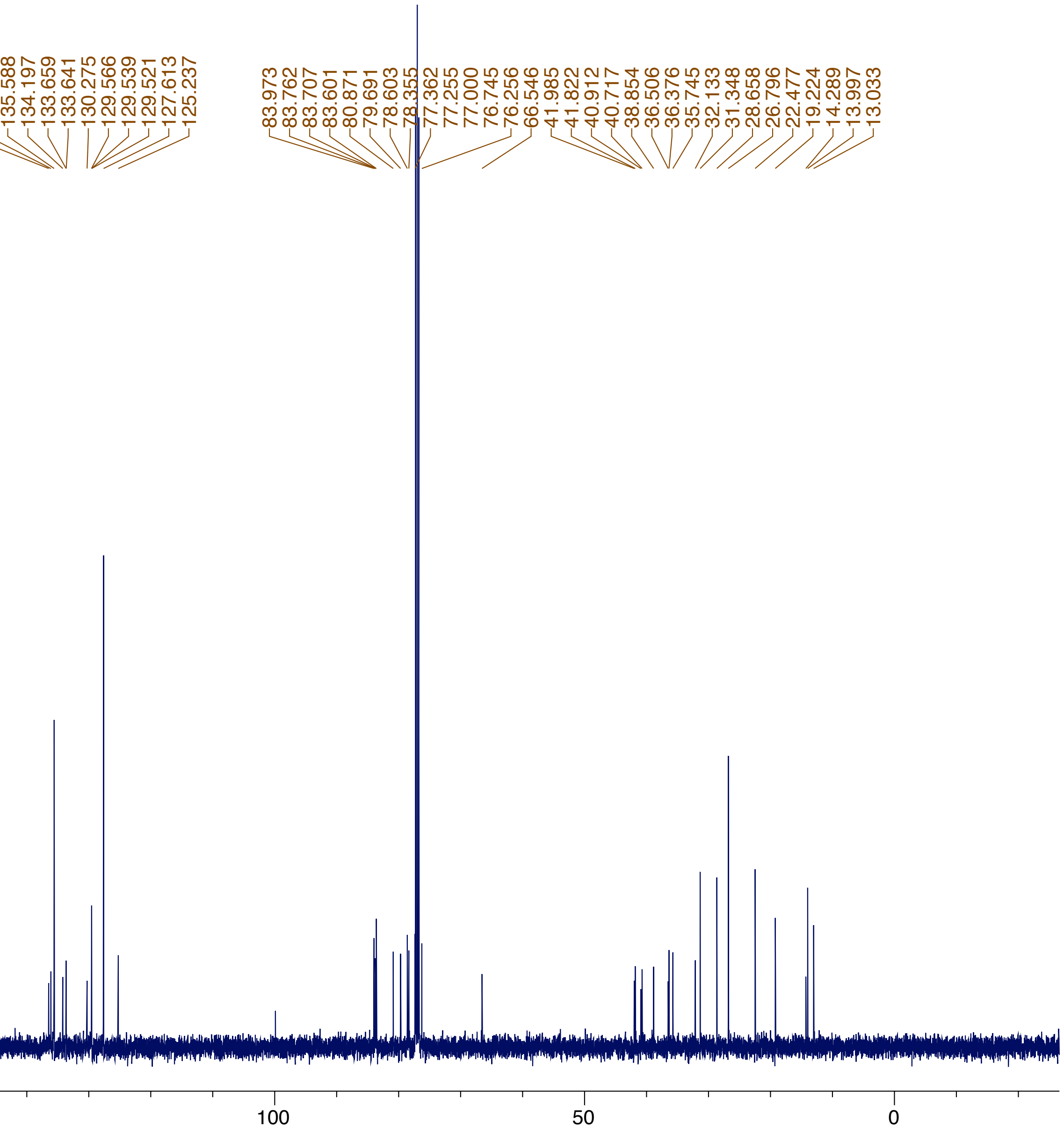

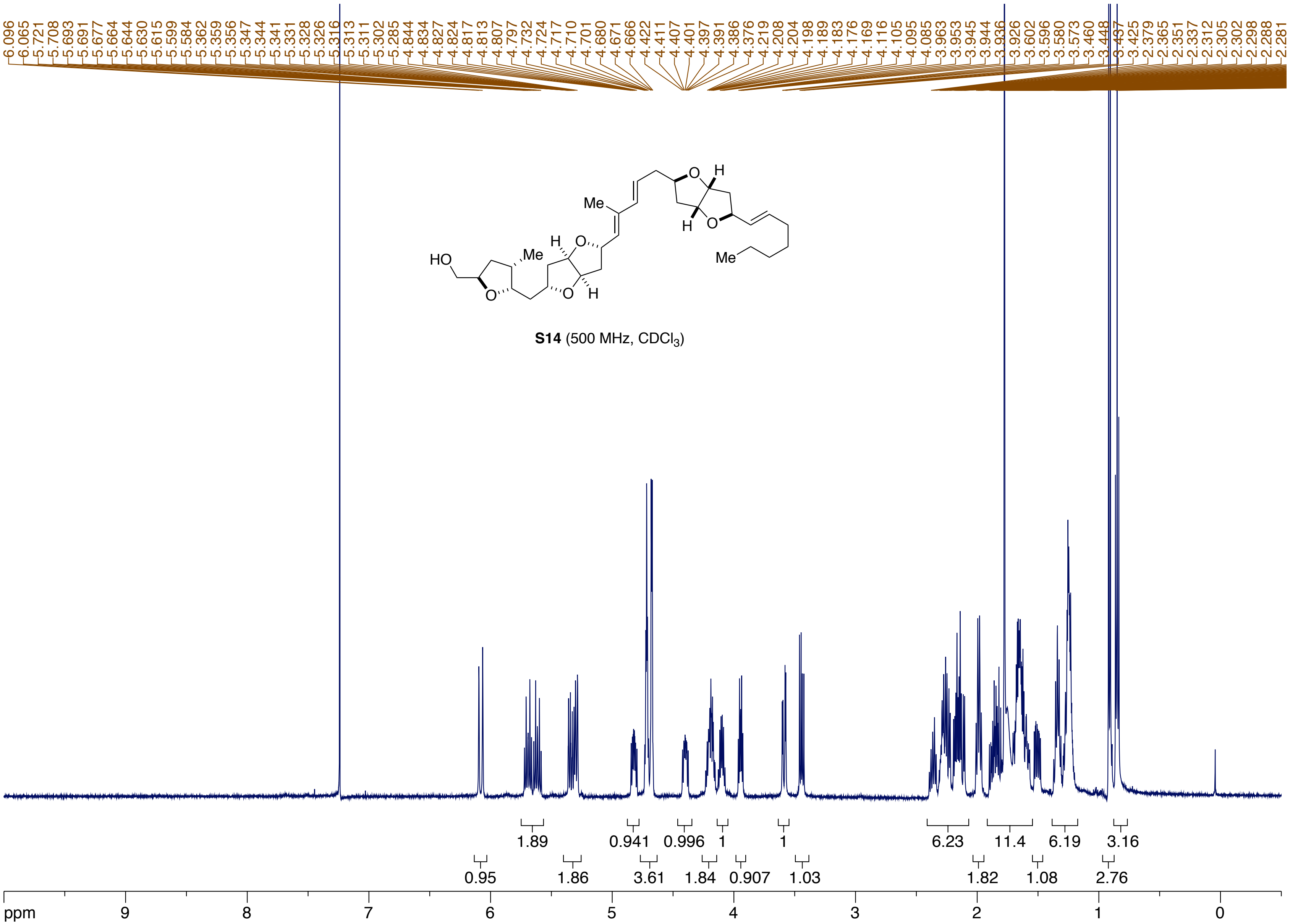

6.096
6.065
5.721
5.708
5.693
5.691
5.677
5.664
5.644
5.630
5.615
5.599
5.584
5.362
5.359
5.356
5.347
5.344
5.341
5.331
5.328
5.326
5.316
5.313
5.311
5.302
5.285
4.844
4.834
4.827
4.824
4.817
4.813
4.807
4.797
4.732
4.724
4.717
4.710
4.701
4.680
4.671
4.666
4.422
4.411
4.407
4.401
4.397
4.391
4.386
4.376
4.219
4.208
4.204
4.198
4.189
4.183
4.176
4.169
4.116
4.105
4.095
4.085
3.963
3.953
3.945
3.944
3.936
3.926
3.602
3.596
3.580
3.573
3.460
3.448
3.437
3.425
2.379
2.365
2.351
2.337
2.312
2.305
2.302
2.298
2.288
2.281
HO
Me
H
O
O
H
Me
O
H
H
O
Me
S14 (500 MHz, CDCl3)
1.89
0.941
0.996
1
1
6.23
11.4
6.19
3.16
0.95
1.86
3.61
1.84
0.907
1.03
1.82
1.08
2.76
ppm
9
8
7
6
5
4
3
2
1
0

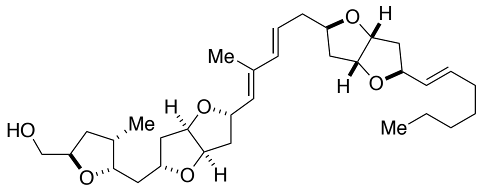

**S14** (125 MHz, $CDCl_3$)

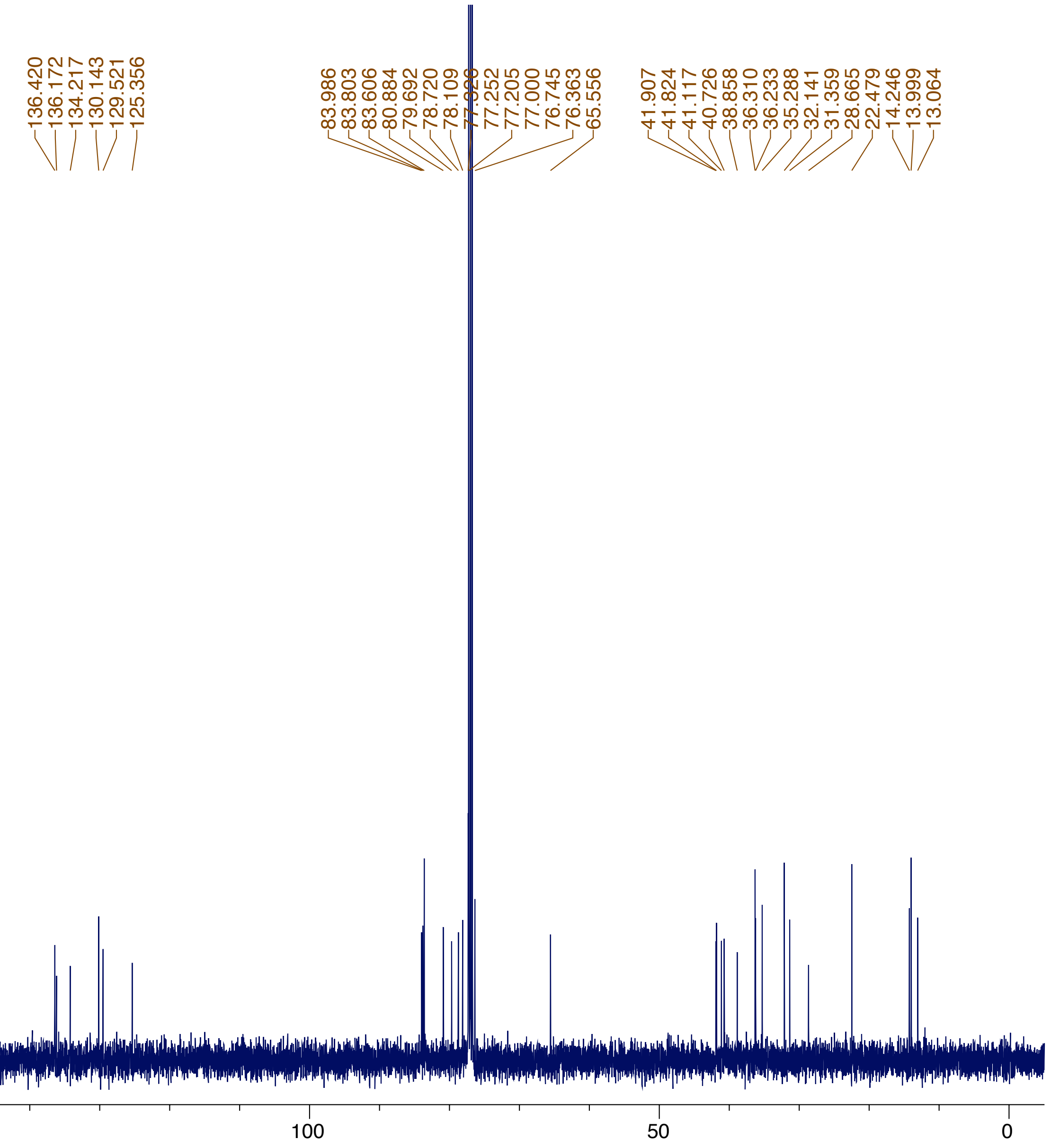

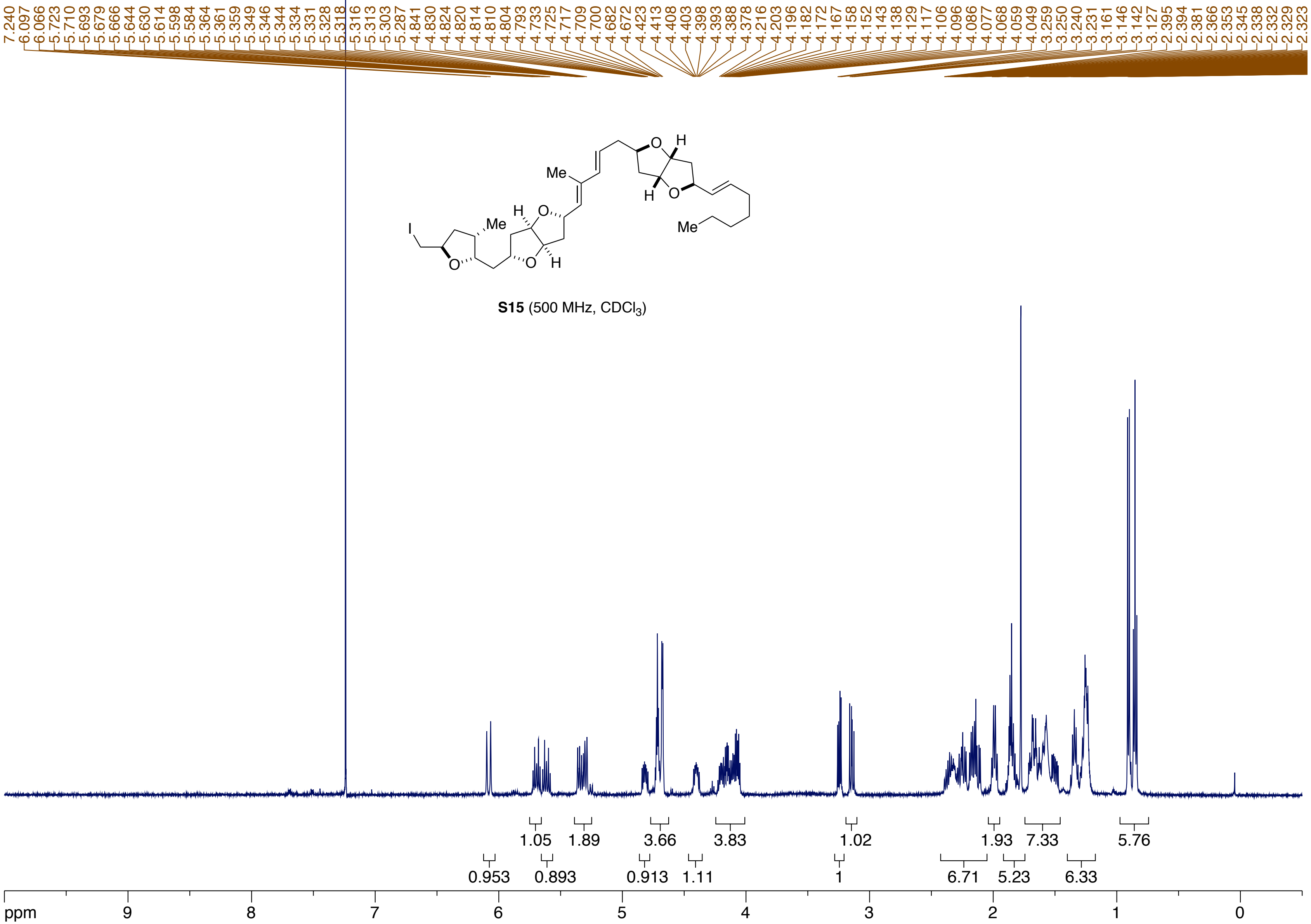


**S15** (500 MHz, $CDCl_3$)

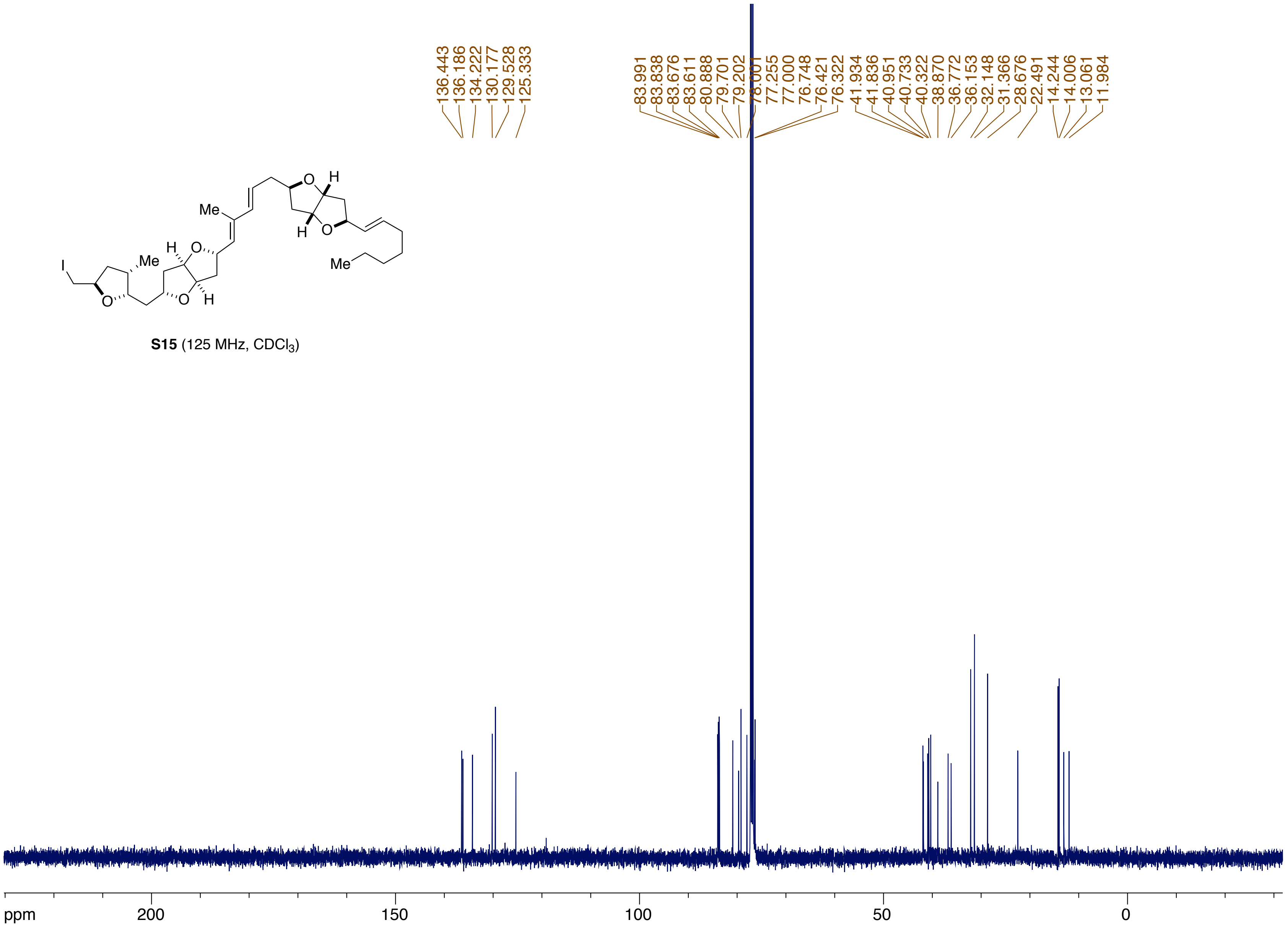


**S15** (125 MHz, $CDCl_3$)

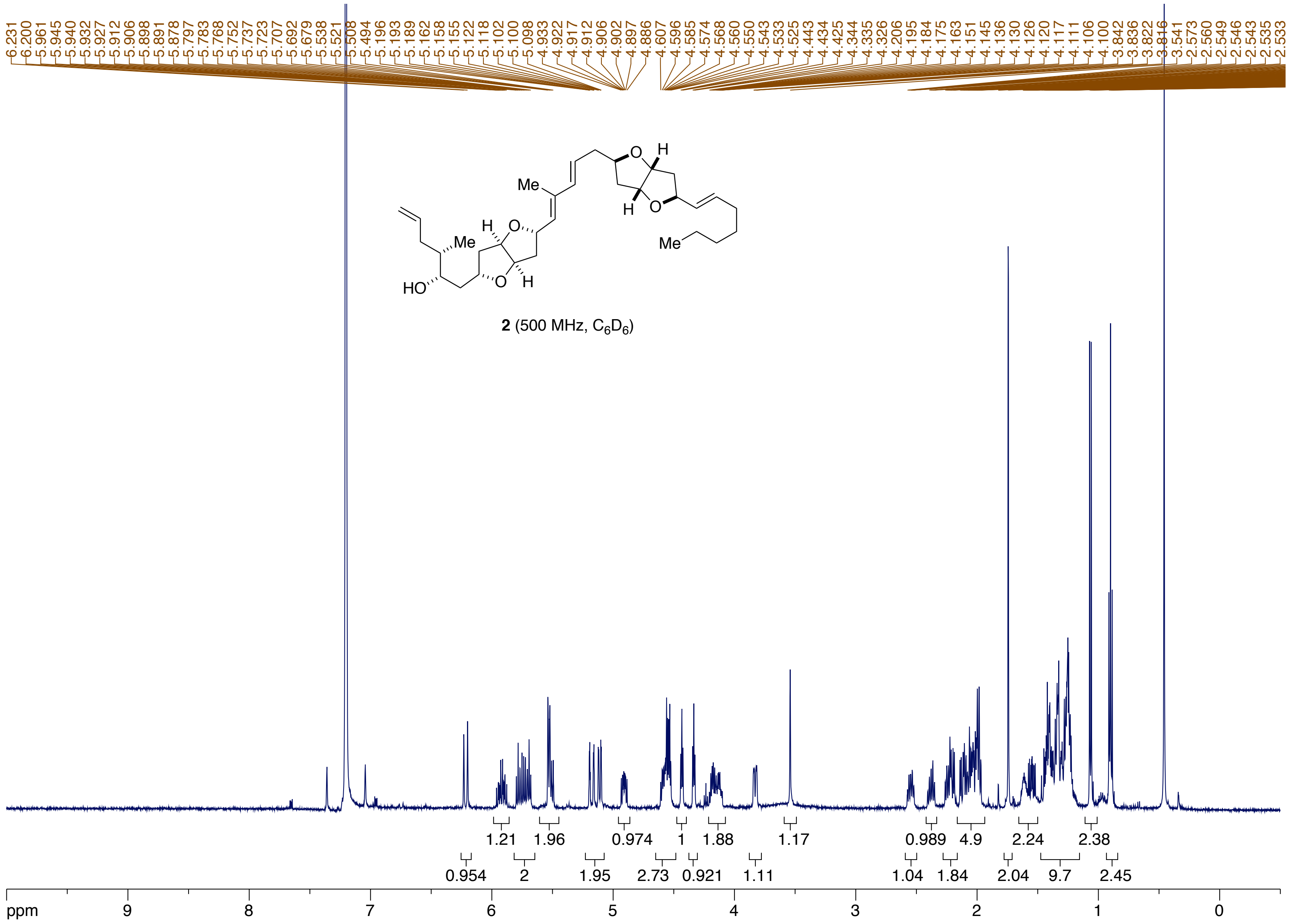

6.231
6.200
5.961
5.945
5.940
5.932
5.927
5.912
5.906
5.898
5.891
5.878
5.797
5.783
5.768
5.752
5.737
5.723
5.707
5.692
5.679
5.538
5.521
5.508
5.494
5.196
5.193
5.189
5.162
5.158
5.155
5.122
5.118
5.102
5.100
5.098
4.933
4.922
4.917
4.912
4.906
4.902
4.897
4.886
4.607
4.596
4.585
4.574
4.568
4.560
4.550
4.543
4.533
4.525
4.443
4.434
4.425
4.344
4.335
4.326
4.206
4.195
4.184
4.175
4.163
4.151
4.145
4.136
4.130
4.126
4.120
4.117
4.111
4.106
4.100
3.842
3.836
3.822
3.816
3.541
2.573
2.560
2.549
2.546
2.543
2.535
2.533
Me
O
H
H
O
Me
H
O
Me
HO
O
H
2 (500 MHz, $C_6D_6$)
1.21
1.96
0.974
1
1.88
1.17
0.989
4.9
2.24
2.38
0.954
2
1.95
2.73
0.921
1.11
1.04
1.84
2.04
9.7
2.45
ppm
9
8
7
6
5
4
3
2
1
0

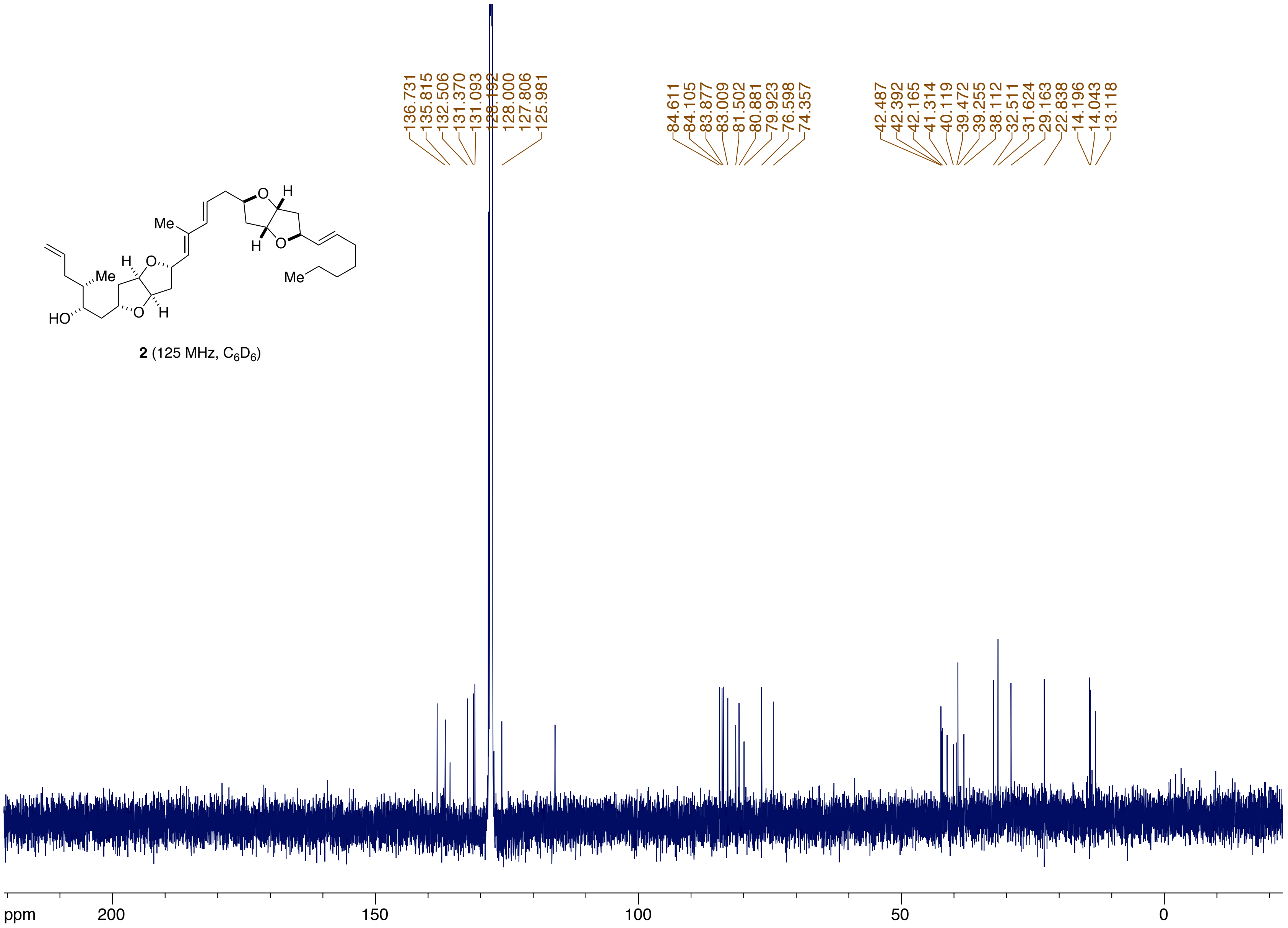


**2** (125 MHz, $C_6D_6$)

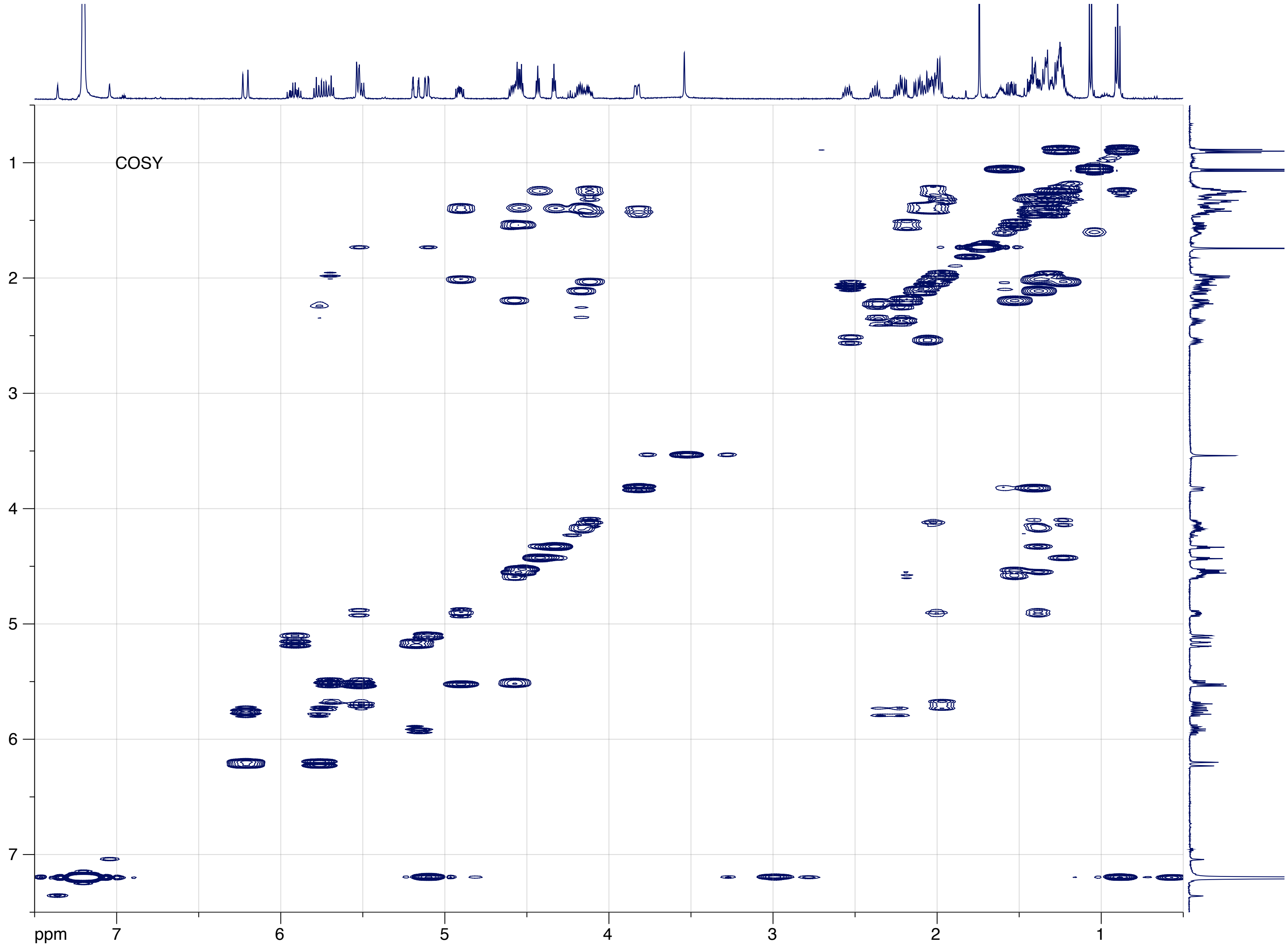
COSY
ppm
7
6
5
4
3
2
1

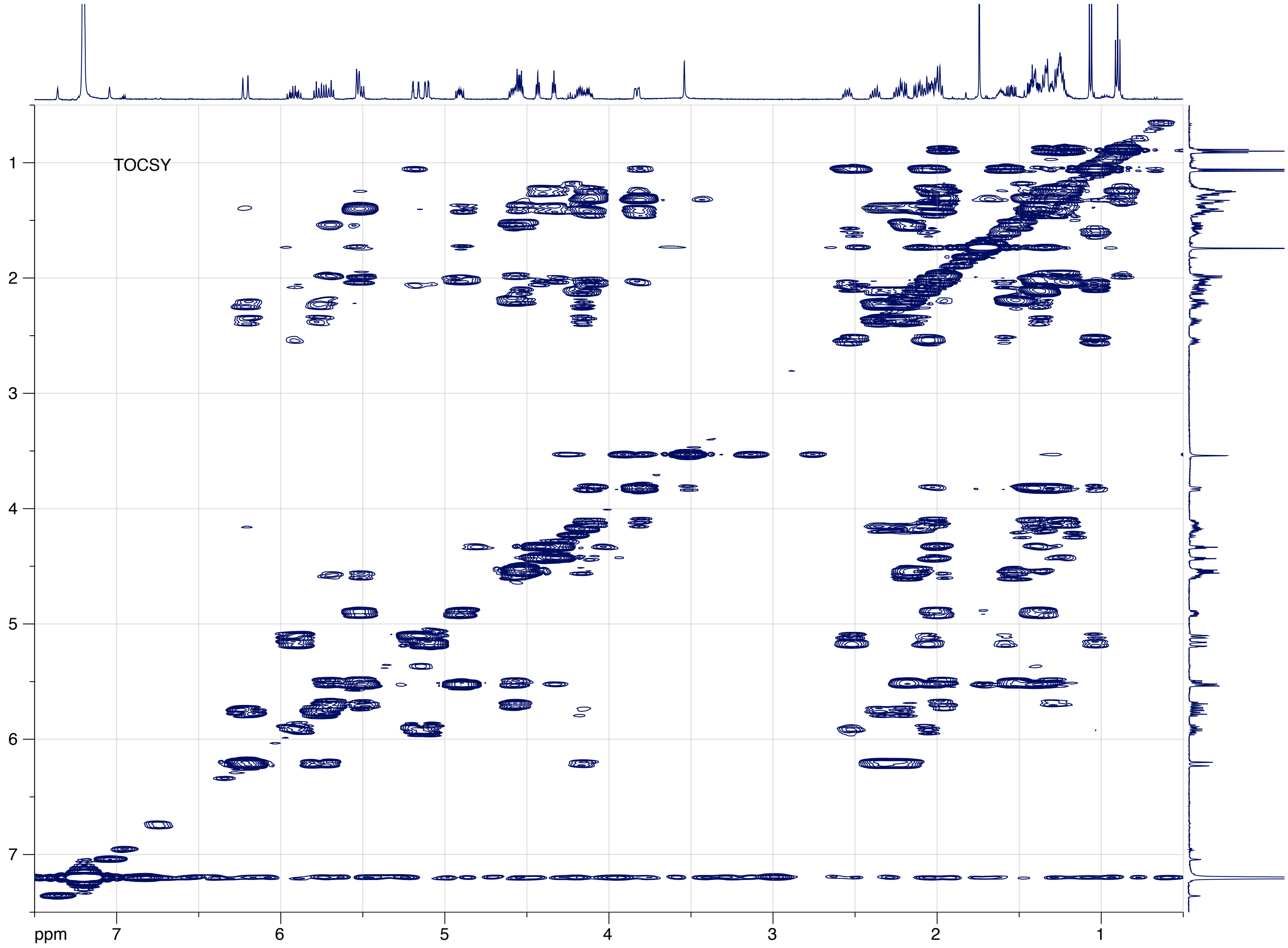
TOCSY
ppm
7
6
5
4
3
2
1

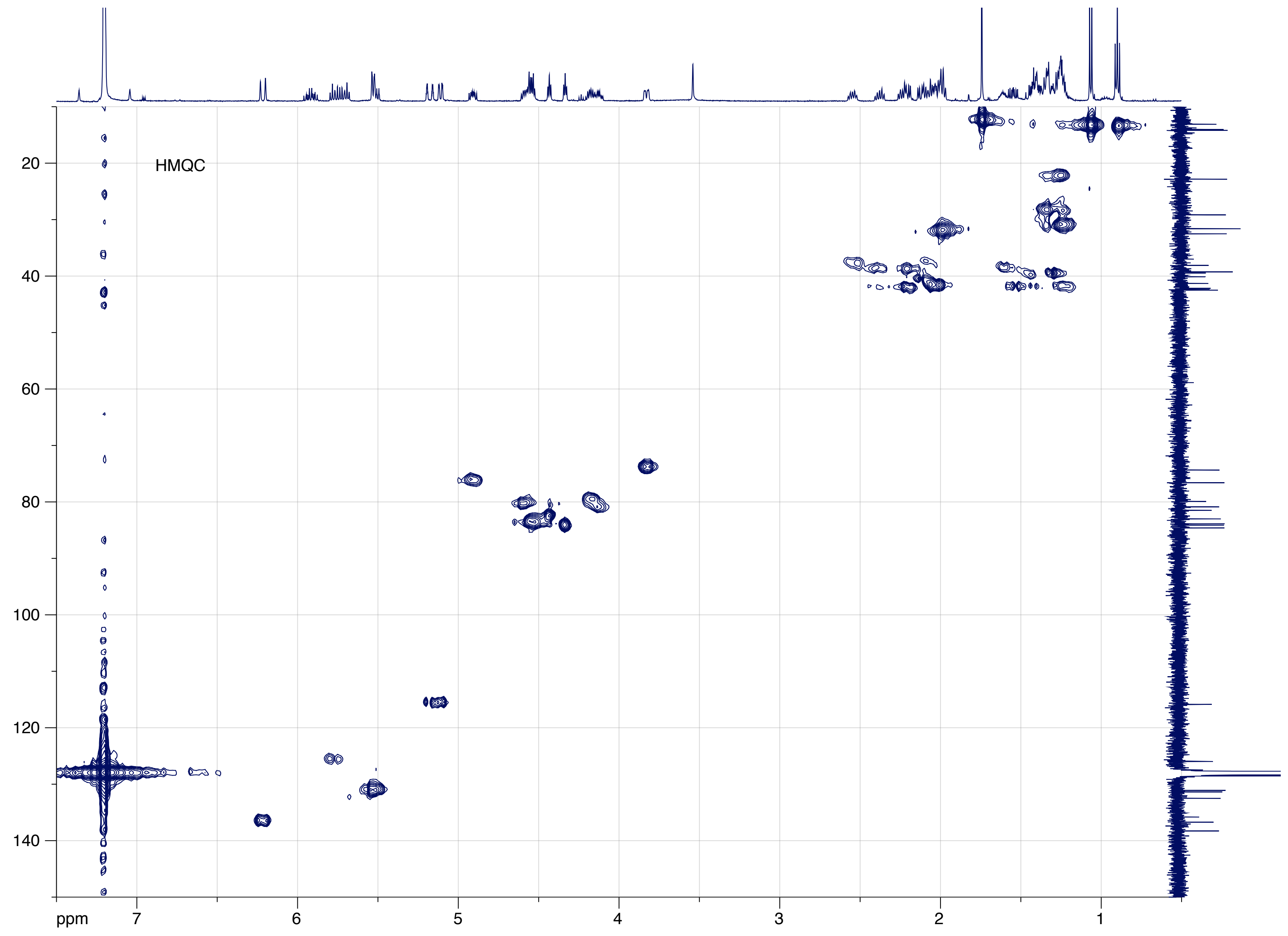


S170

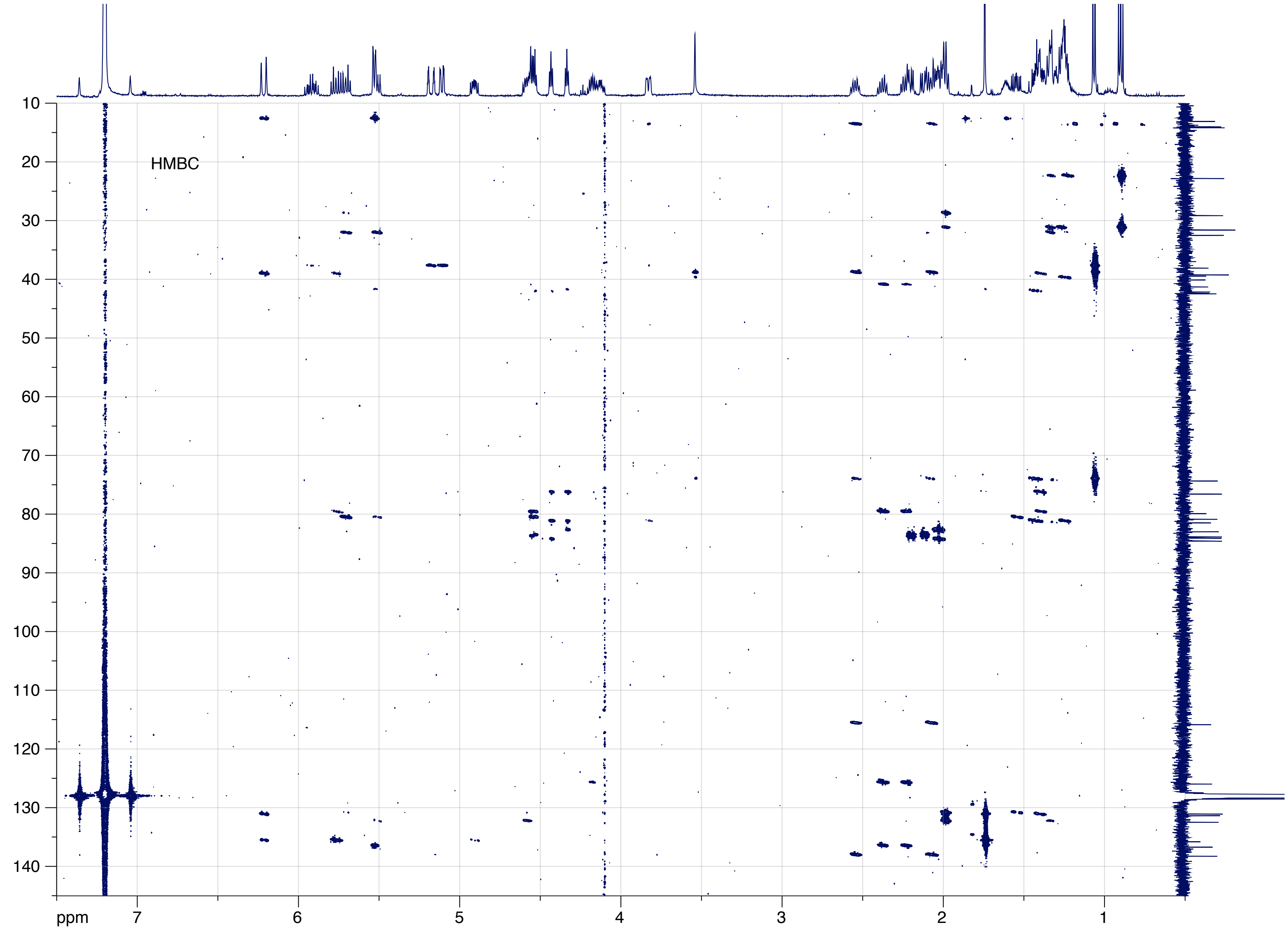
HMBC
ppm
7
6
5
4
3
2
1
10
20
30
40
50
60
70
80
90
100
110
120
130
140

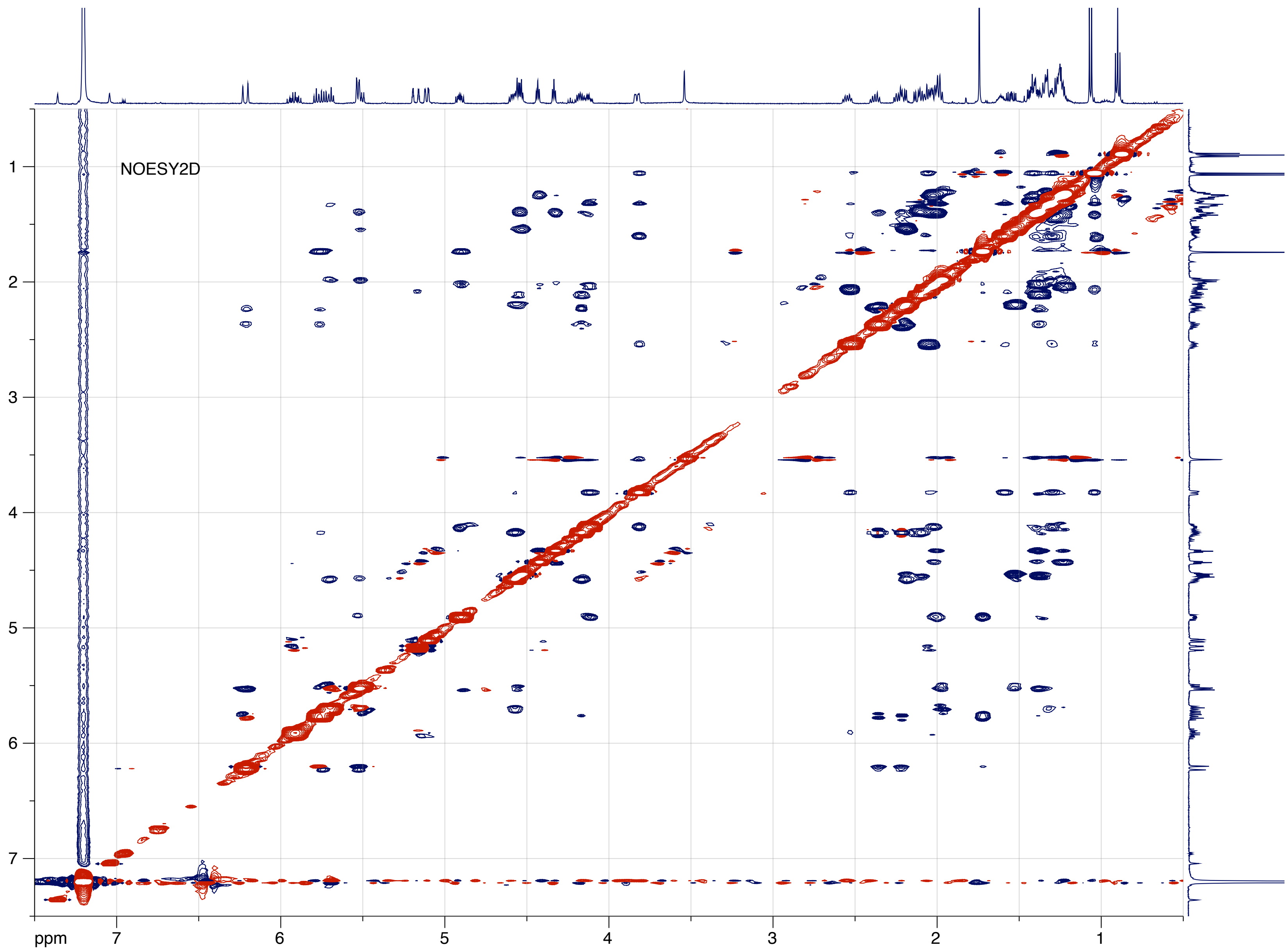


S172

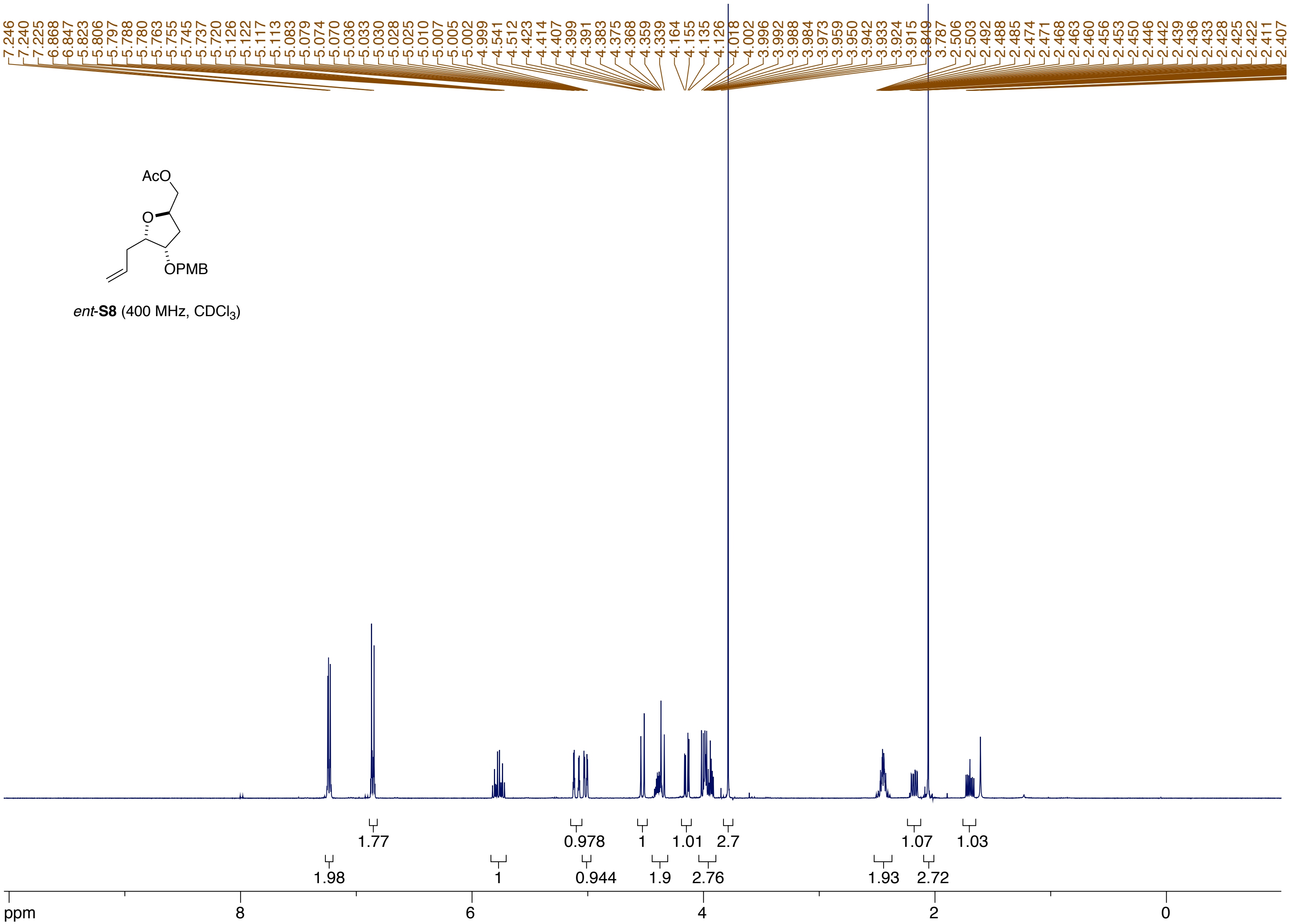

*ent*-**S8** (400 MHz, $CDCl_3$)

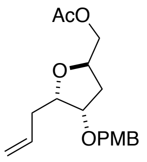

*ent*-**S8** (100 MHz, $CDCl_3$)

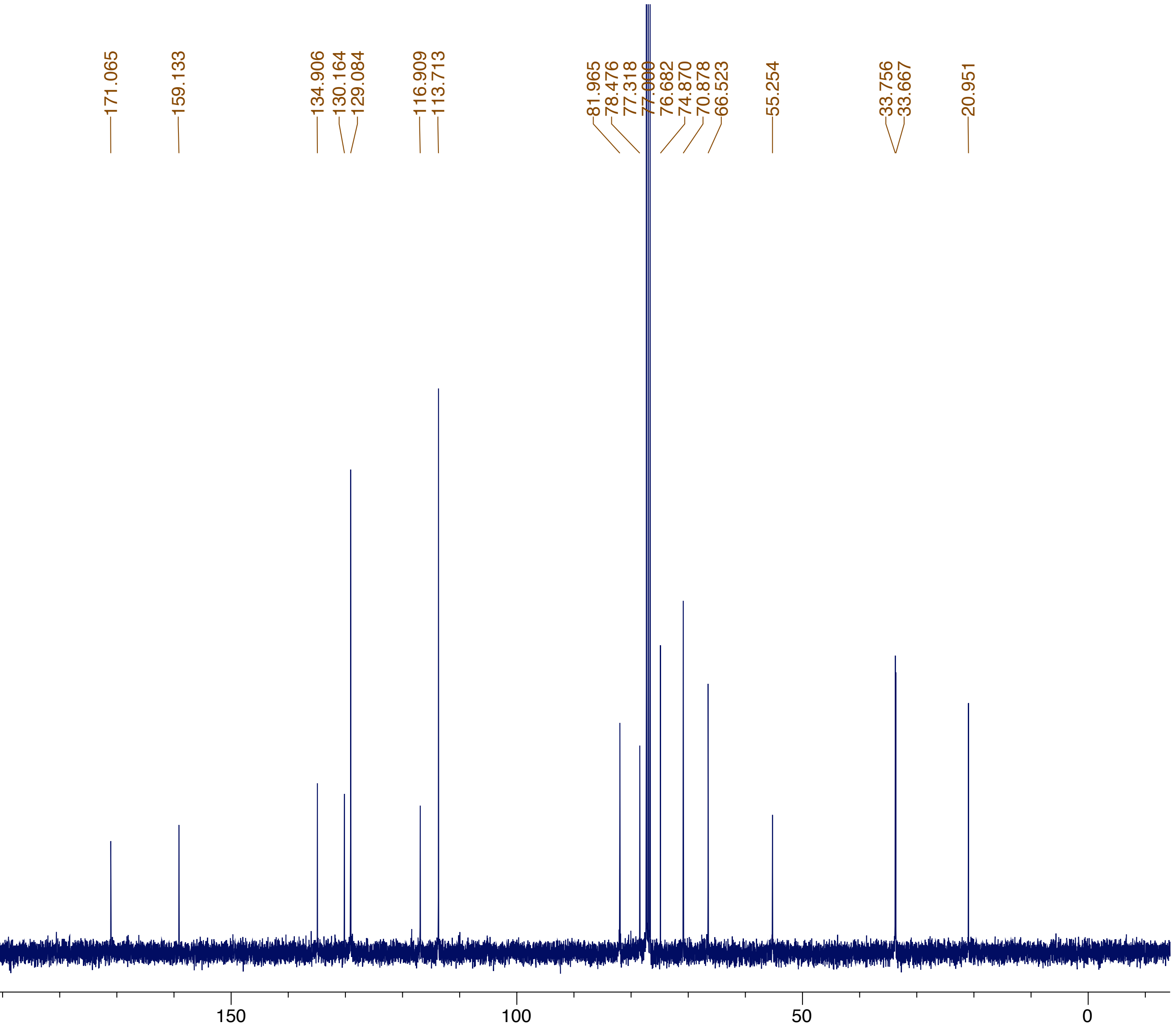

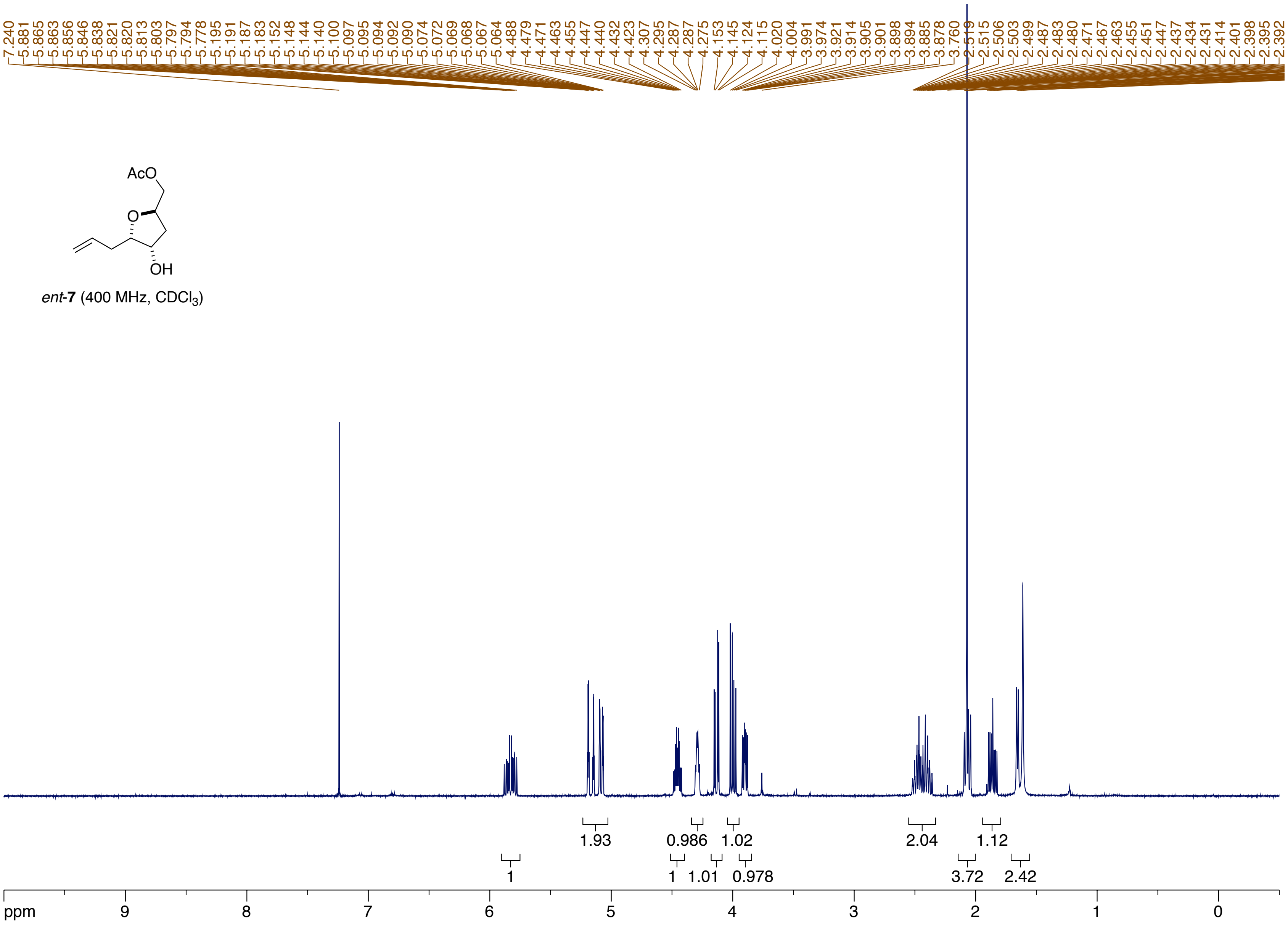

AcO
O
OH
*ent*-**7** (400 MHz, $CDCl_3$)
7.240
5.881
5.865
5.863
5.856
5.846
5.838
5.821
5.820
5.813
5.803
5.797
5.794
5.778
5.195
5.191
5.187
5.183
5.152
5.148
5.144
5.140
5.100
5.097
5.095
5.094
5.092
5.090
5.074
5.072
5.069
5.068
5.067
5.064
4.488
4.479
4.471
4.463
4.455
4.447
4.440
4.432
4.423
4.307
4.295
4.287
4.287
4.275
4.153
4.145
4.124
4.115
4.020
4.004
3.991
3.974
3.921
3.914
3.905
3.901
3.898
3.894
3.885
3.878
3.760
2.519
2.515
2.506
2.503
2.499
2.487
2.483
2.480
2.471
2.467
2.463
2.455
2.451
2.447
2.437
2.434
2.431
2.414
2.401
2.398
2.395
2.392
1.93
0.986
1.02
2.04
1.12
1
1
1.01
0.978
3.72
2.42
ppm
9
8
7
6
5
4
3
2
1
0

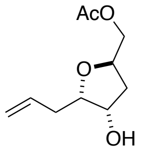

*ent*-**7** (100 MHz, $CDCl_3$)

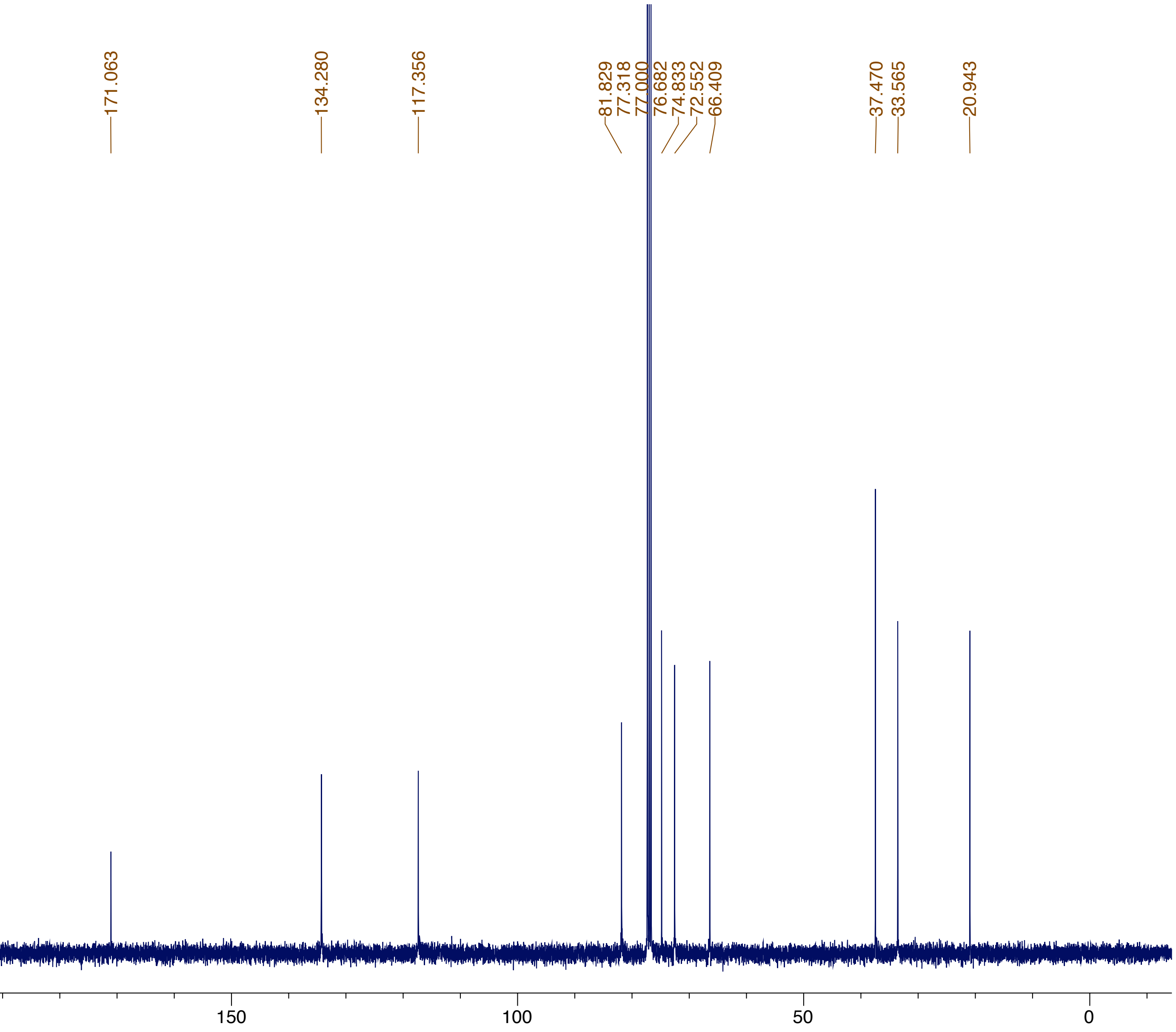

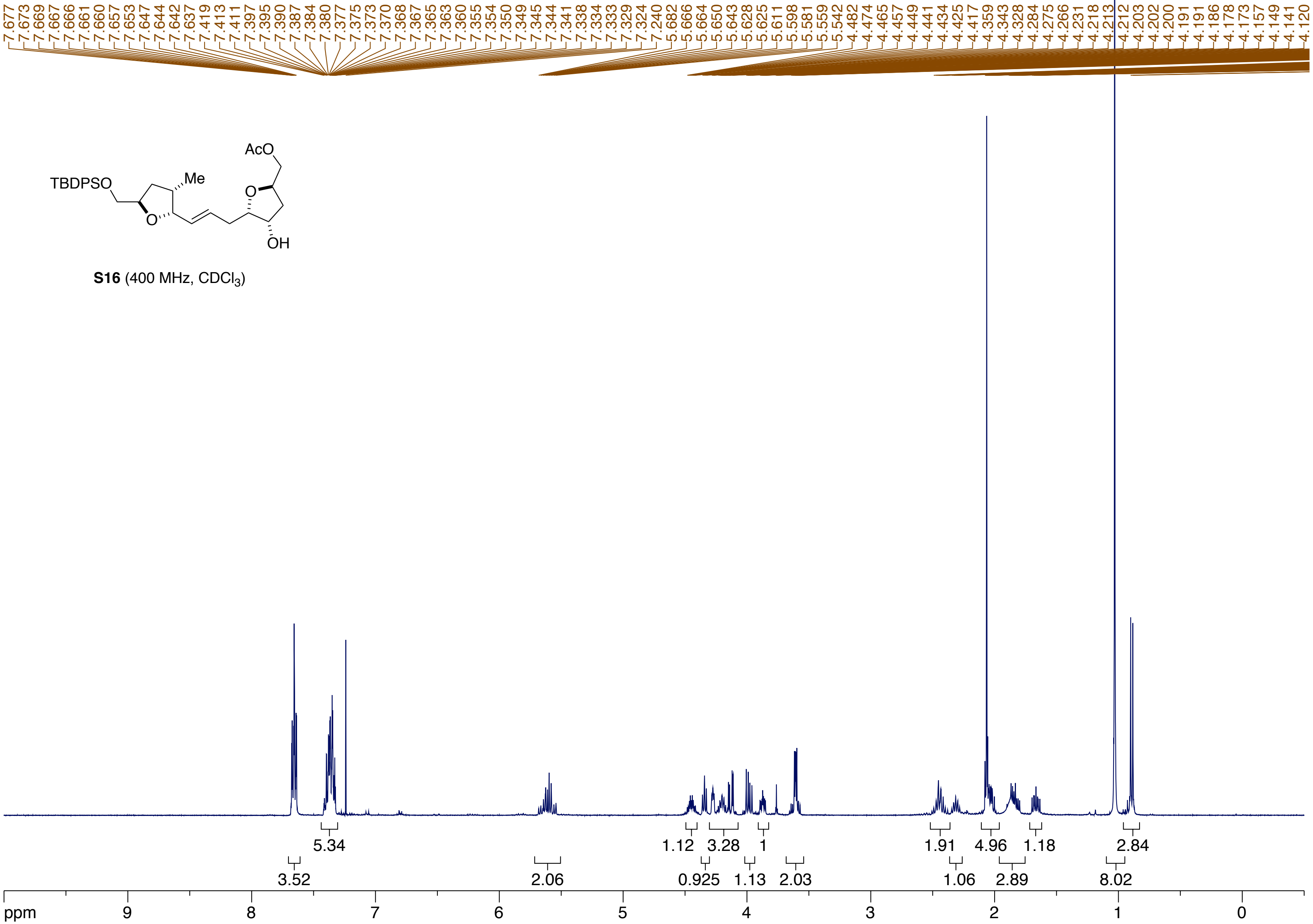


**S16** (400 MHz, $CDCl_3$)

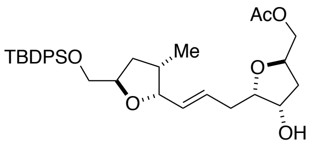

**S16** (100 MHz, $CDCl_3$)

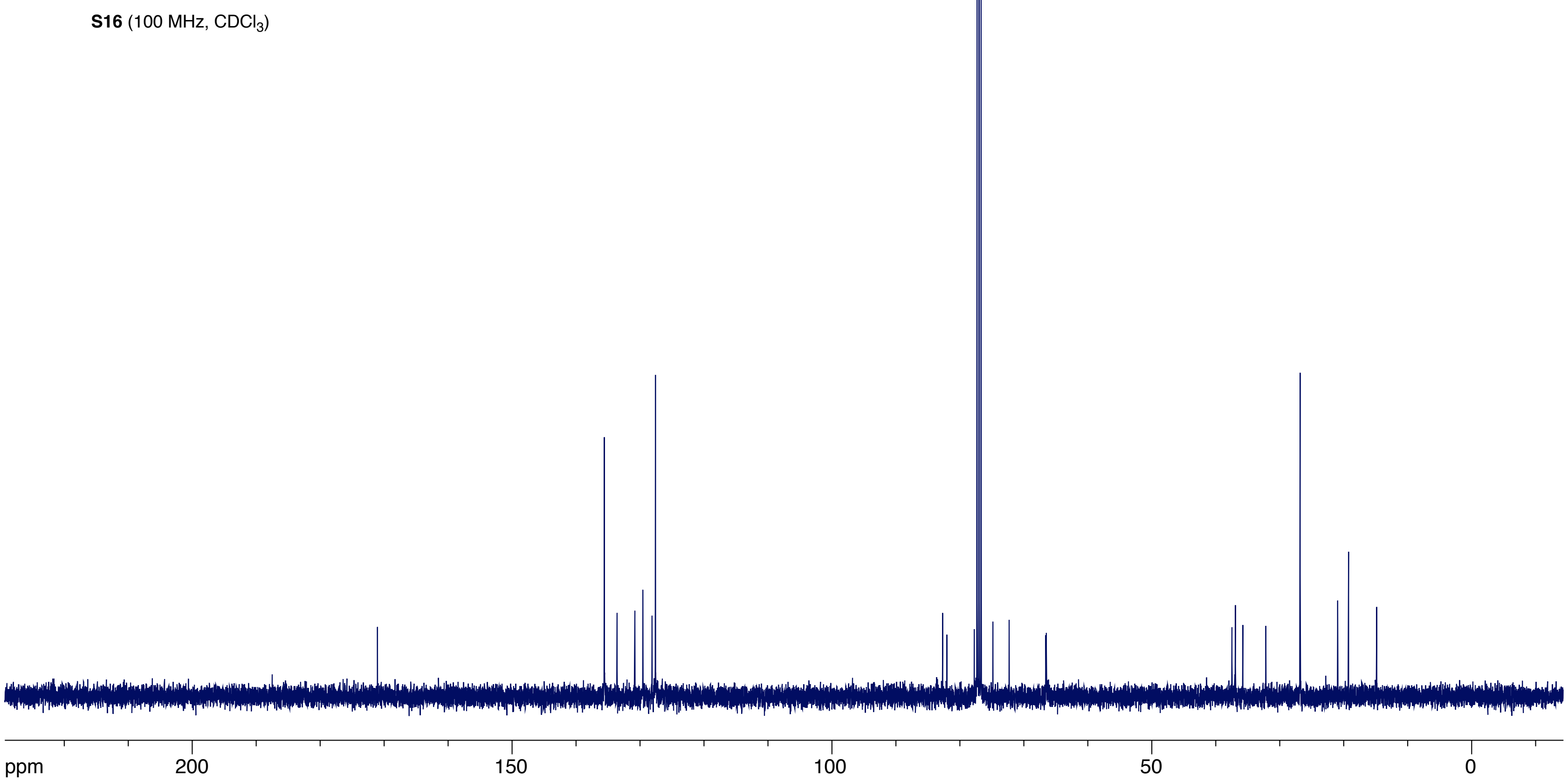

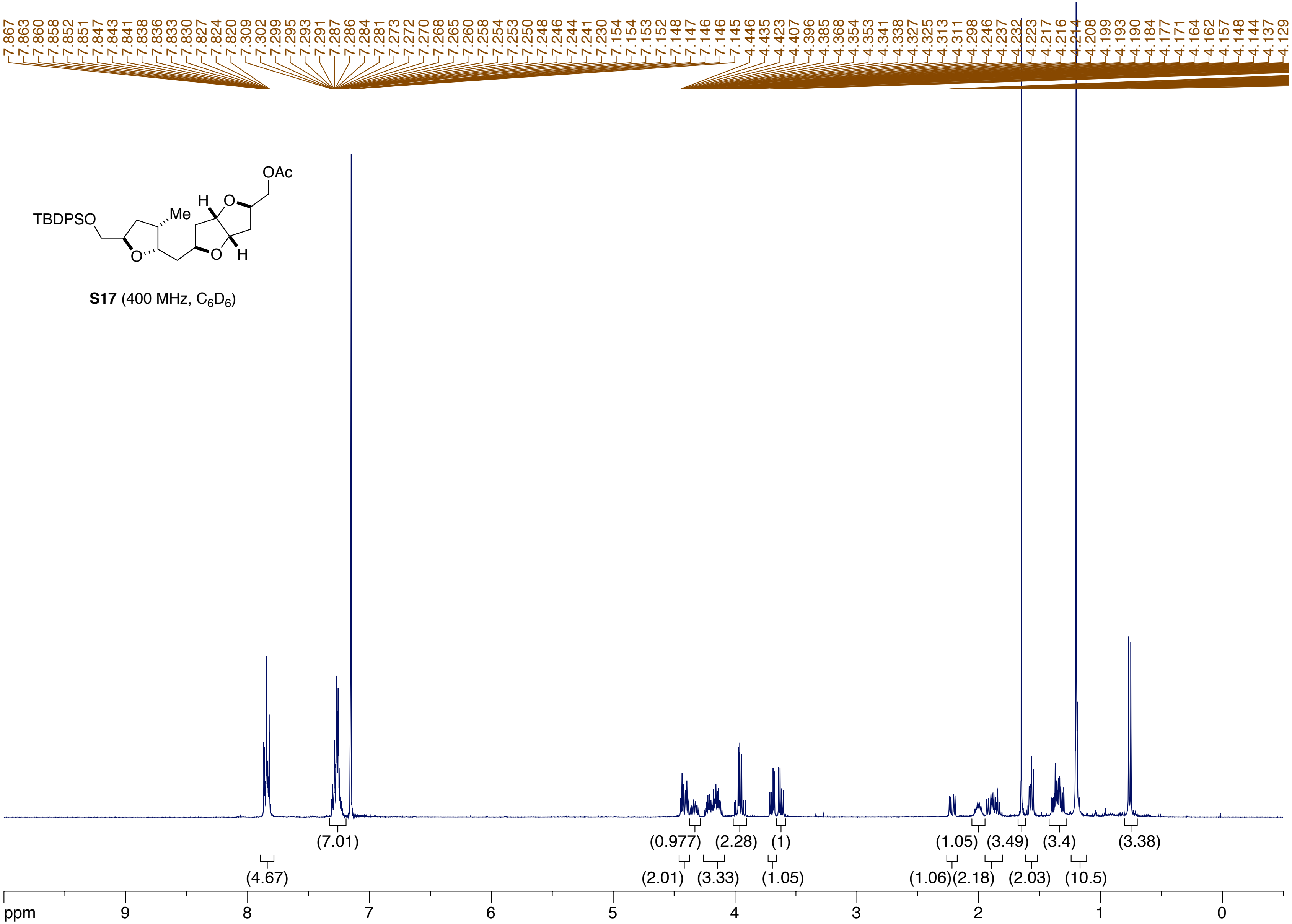


**S17** (400 MHz, $C_6D_6$)

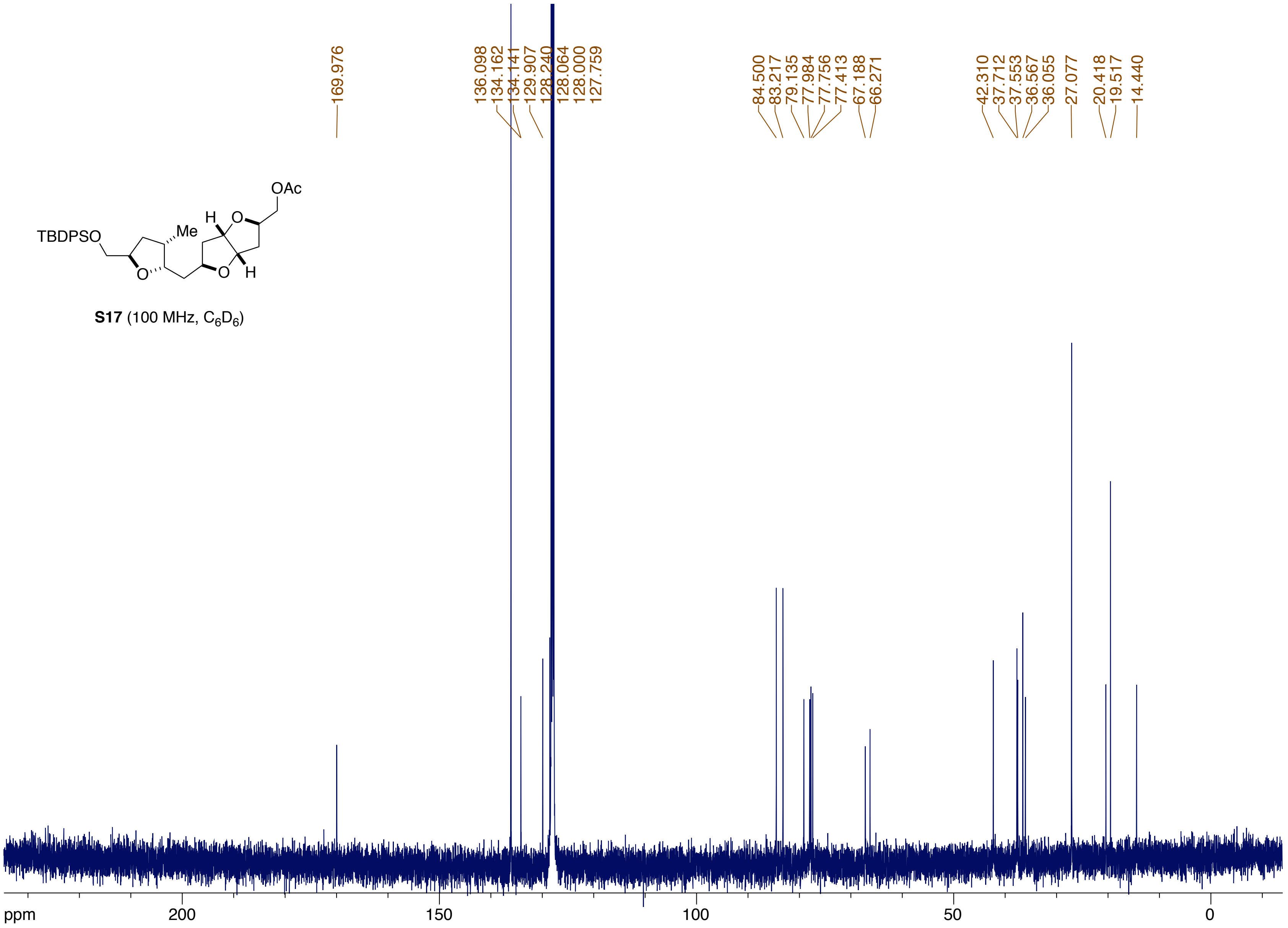


**S17** (100 MHz, $C_6D_6$)

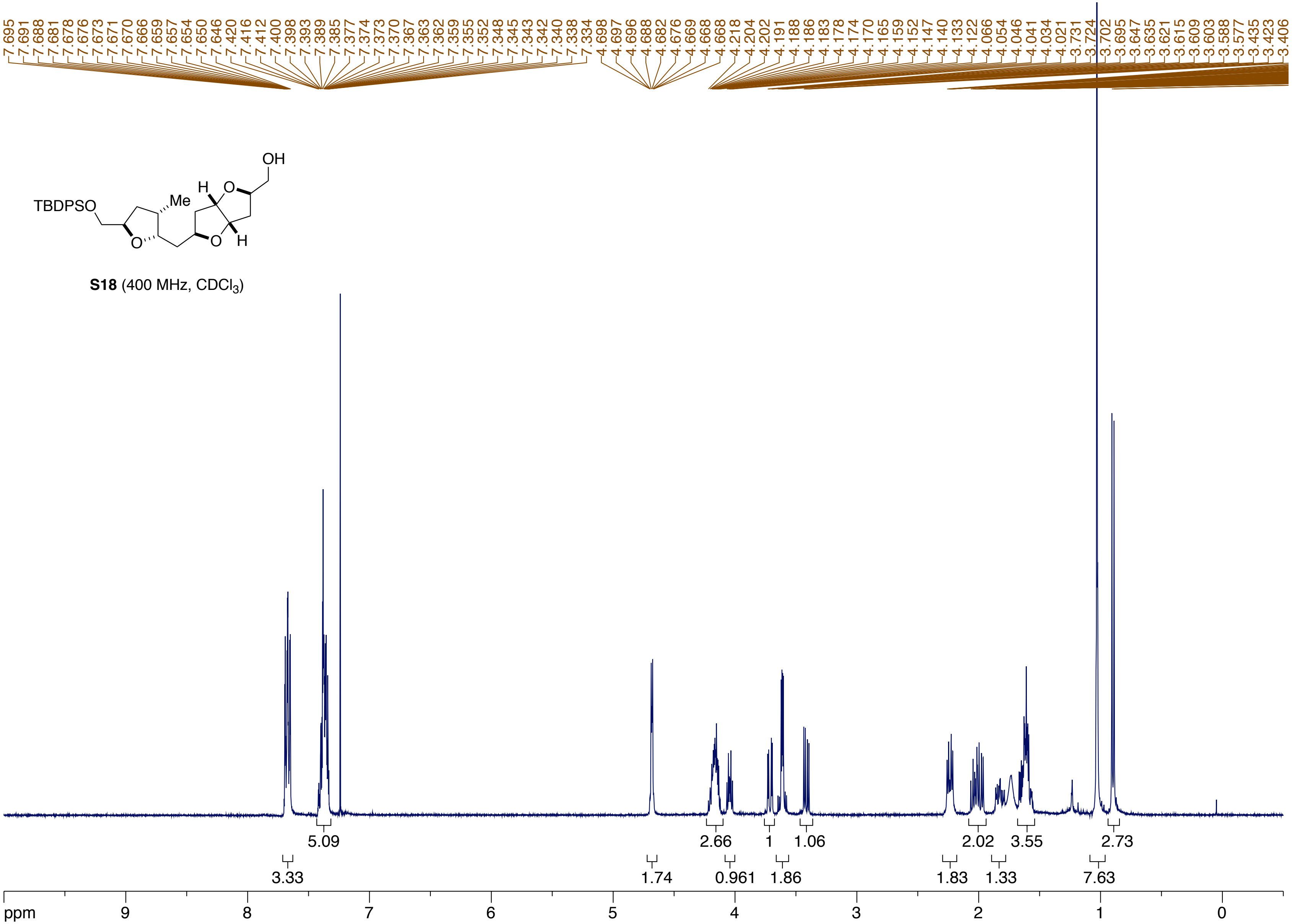

TBDPSO
Me
H
O
OH
O
O
H
S18 (400 MHz, CDCl3)
7.695 7.691 7.688 7.681 7.678 7.676 7.673 7.671 7.670 7.666 7.659 7.657 7.654 7.650 7.646 7.420 7.416 7.412 7.400 7.398 7.393 7.389 7.385 7.377 7.374 7.373 7.370 7.367 7.363 7.362 7.359 7.355 7.352 7.348 7.345 7.343 7.342 7.340 7.338 7.334 4.698 4.697 4.696 4.688 4.682 4.676 4.669 4.668 4.218 4.204 4.202 4.191 4.188 4.186 4.183 4.178 4.174 4.170 4.165 4.159 4.152 4.147 4.140 4.133 4.122 4.066 4.054 4.046 4.041 4.034 4.021 3.731 3.724 3.702 3.695 3.647 3.635 3.621 3.615 3.609 3.603 3.588 3.577 3.435 3.423 3.406
3.33
5.09
1.74
2.66
0.961
1
1.86
1.06
1.83
2.02
1.33
3.55
7.63
2.73
ppm
9
8
7
6
5
4
3
2
1
0

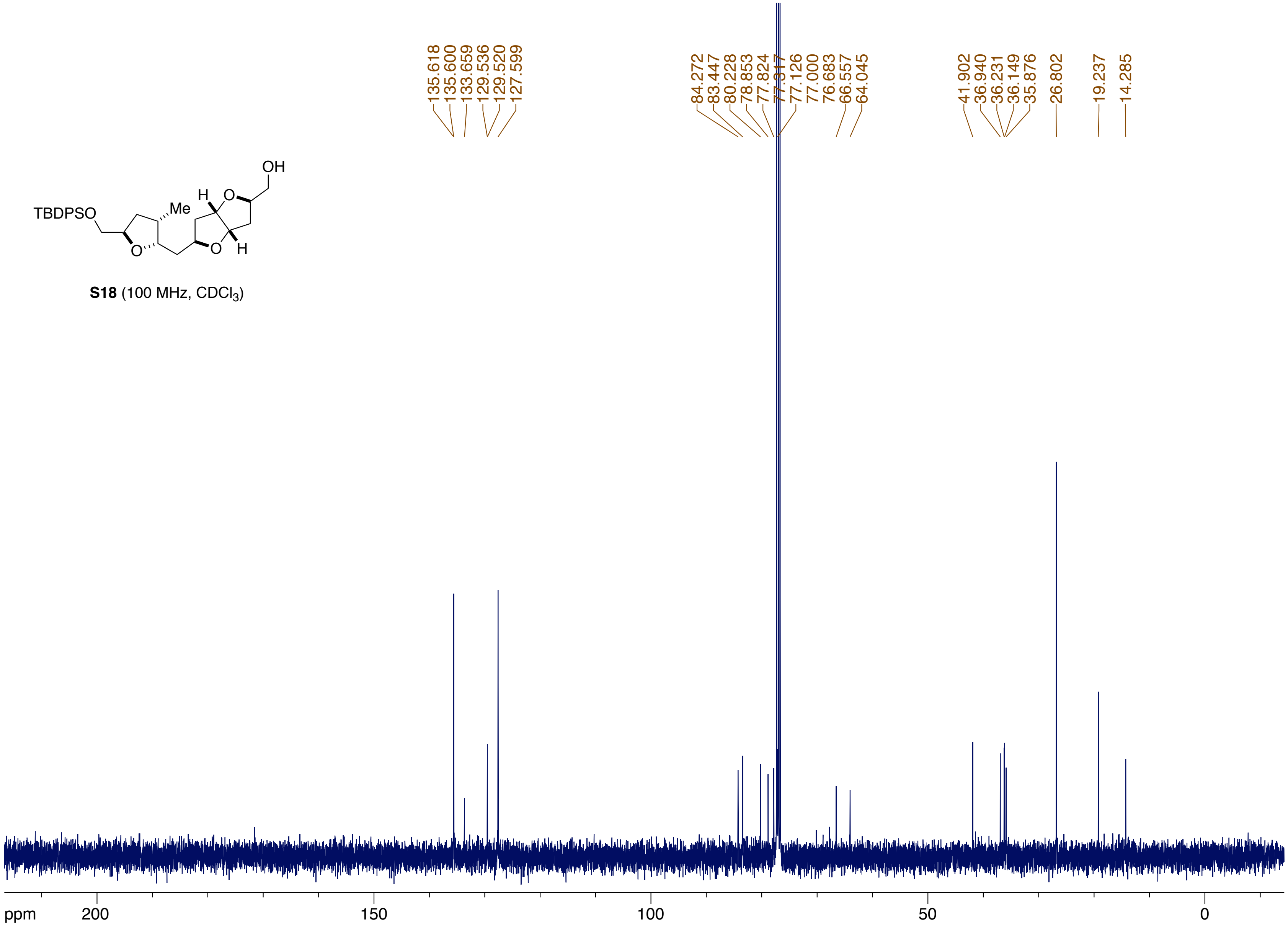


**S18** (100 MHz, $CDCl_3$)

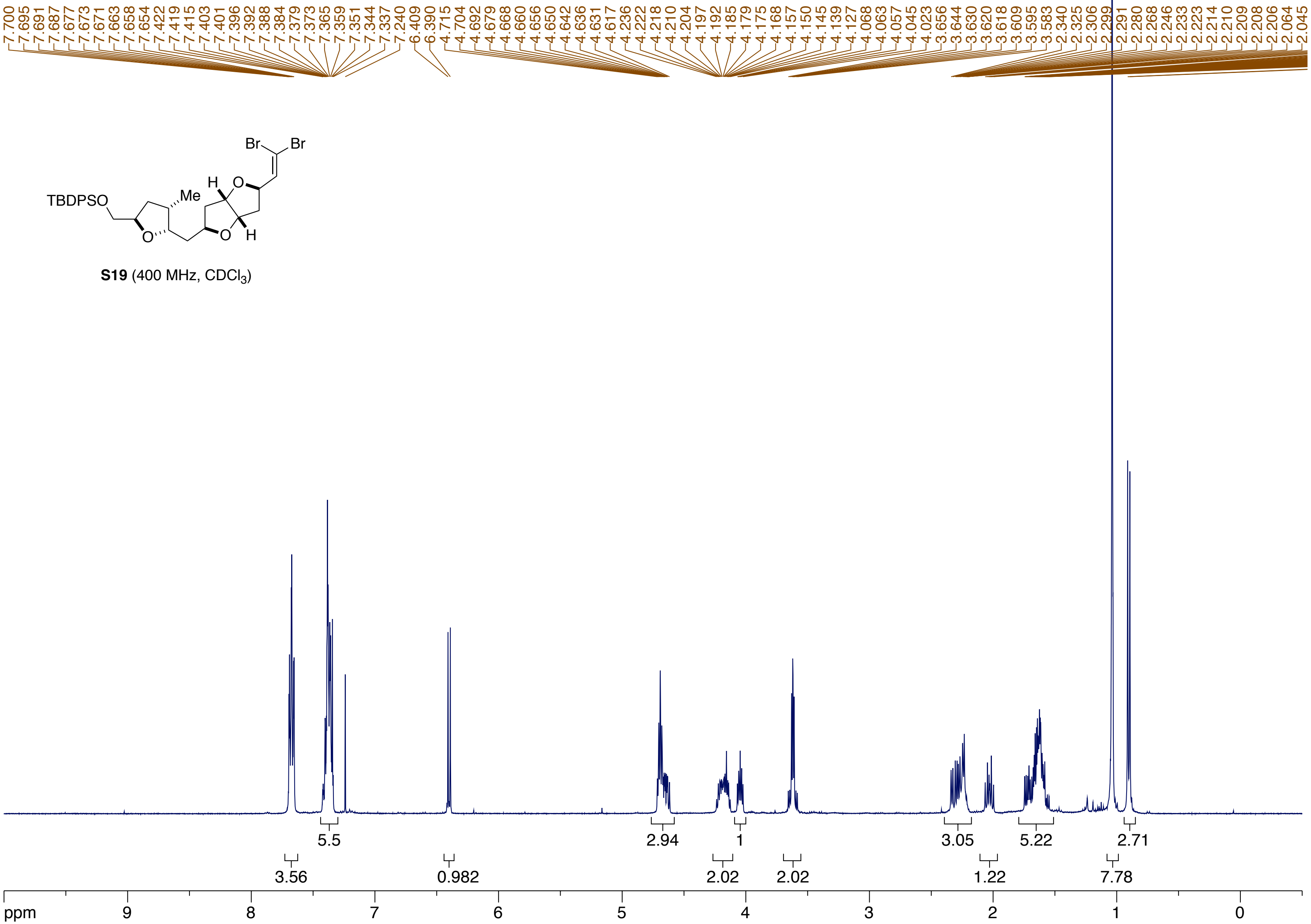
7.700 7.695 7.691 7.687 7.677 7.673 7.671 7.663 7.658 7.654 7.422 7.419 7.415 7.403 7.401 7.396 7.392 7.388 7.384 7.379 7.373 7.365 7.359 7.351 7.344 7.337 7.240 6.409 6.390 4.715 4.704 4.692 4.679 4.668 4.660 4.656 4.650 4.642 4.636 4.631 4.617 4.236 4.222 4.218 4.210 4.204 4.197 4.192 4.185 4.179 4.175 4.168 4.157 4.150 4.145 4.139 4.127 4.068 4.063 4.057 4.045 4.023 3.656 3.644 3.630 3.620 3.618 3.609 3.595 3.583 2.340 2.325 2.306 2.299 2.291 2.280 2.268 2.246 2.233 2.223 2.214 2.210 2.209 2.208 2.206 2.064 2.045
Br
Br
H
O
TBDPSO
Me
O
O
H
**S19** (400 MHz, $CDCl_3$)
5.5
2.94
1
3.05
5.22
2.71
3.56
0.982
2.02
2.02
1.22
7.78
ppm
9
8
7
6
5
4
3
2
1
0

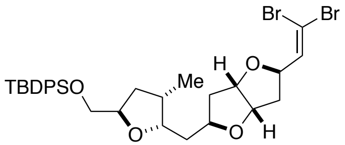


**S19** (100 MHz, $CDCl_3$)

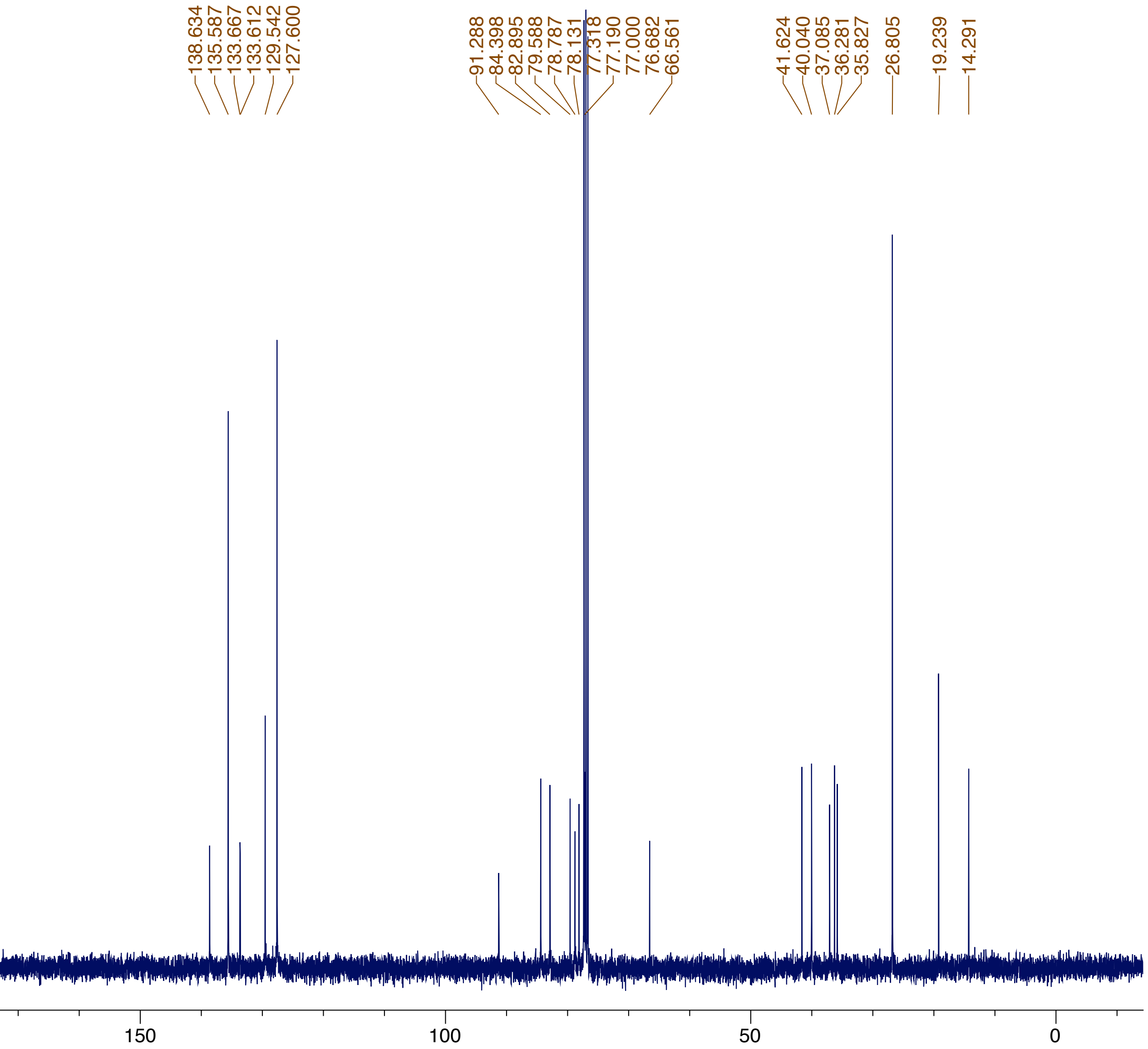

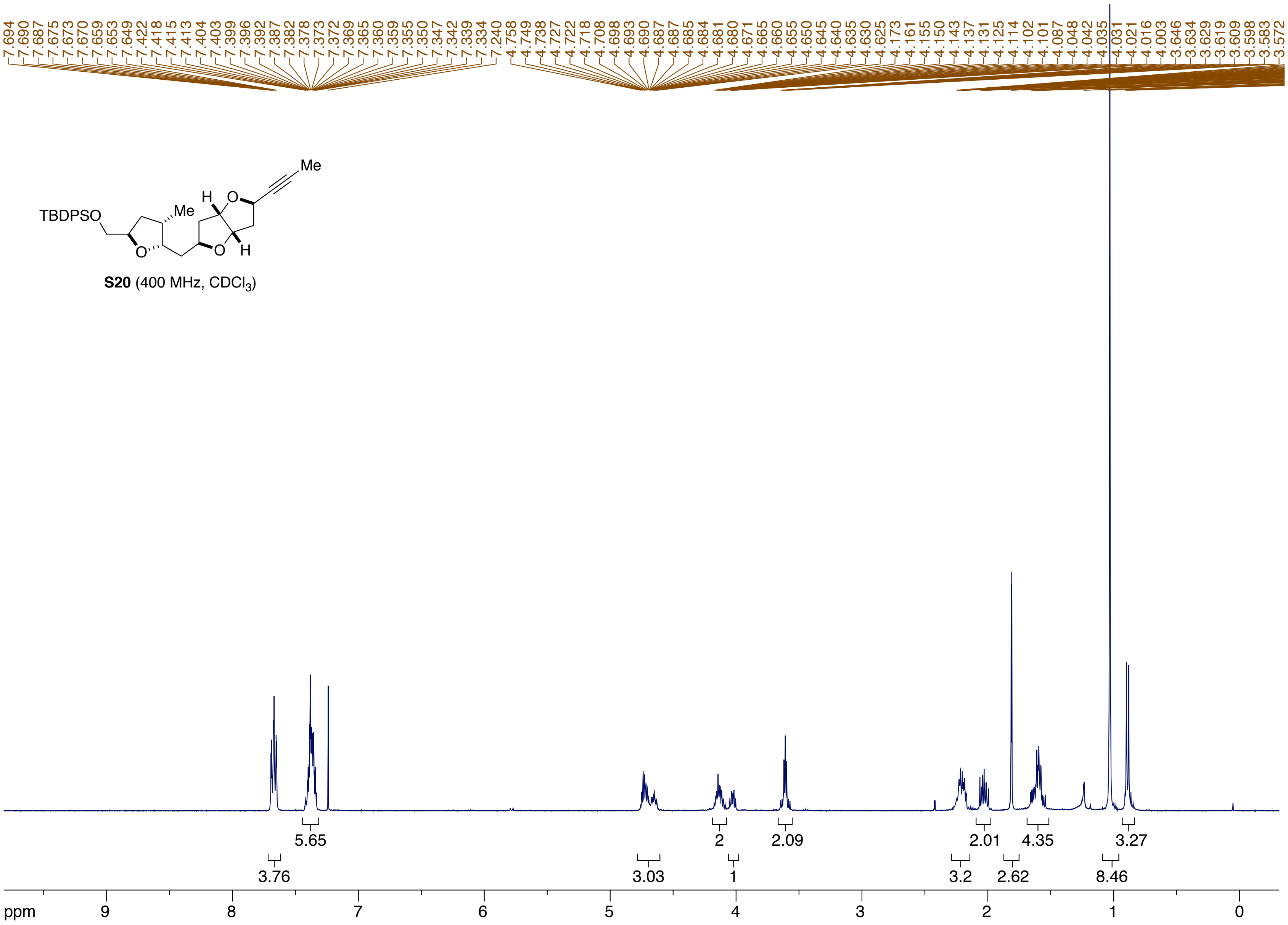


**S20** (400 MHz, $CDCl_3$)

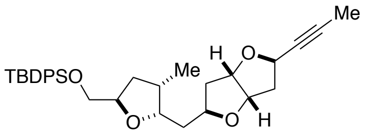


**S20** (100 MHz, CDCl$_3$)

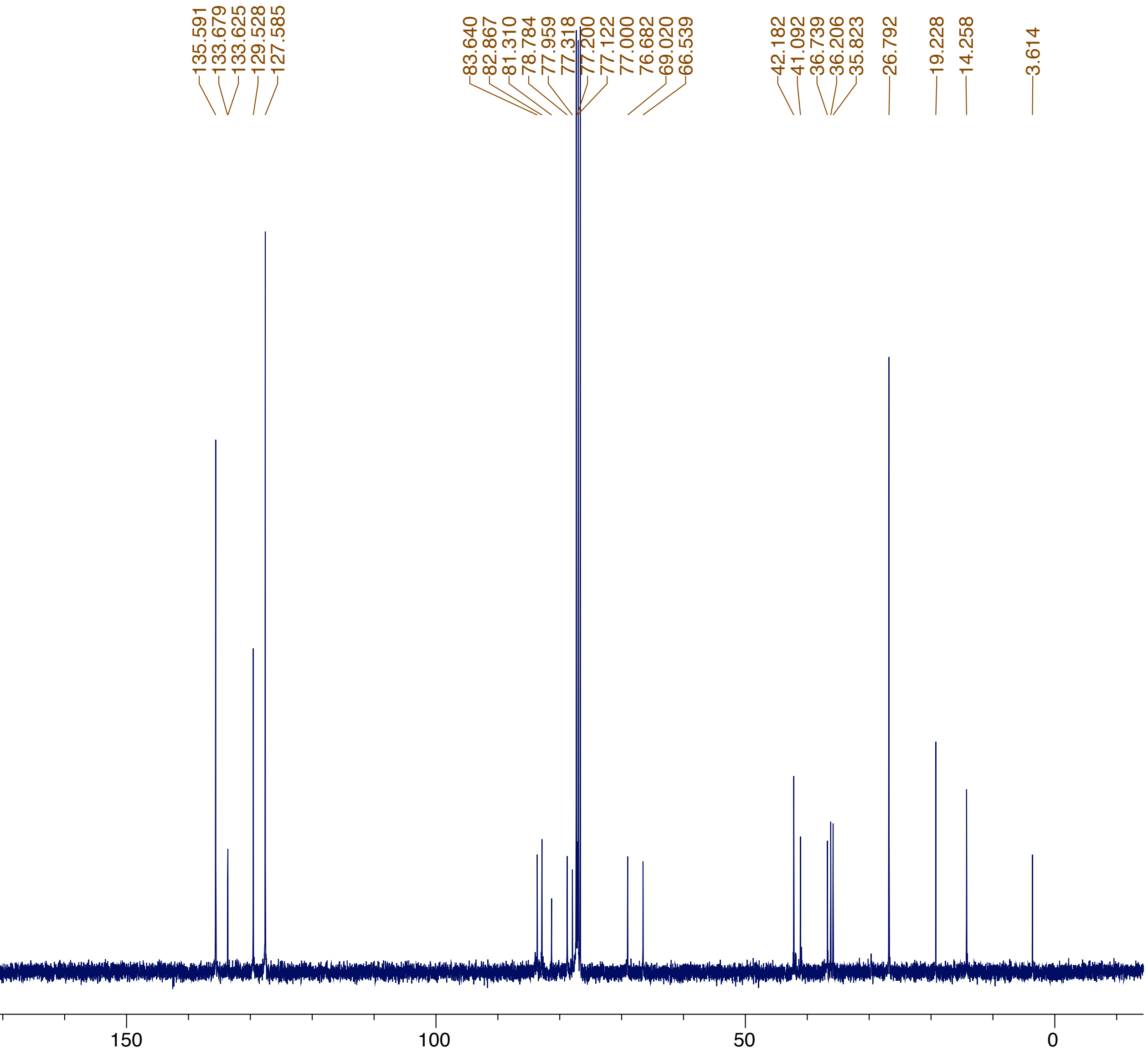

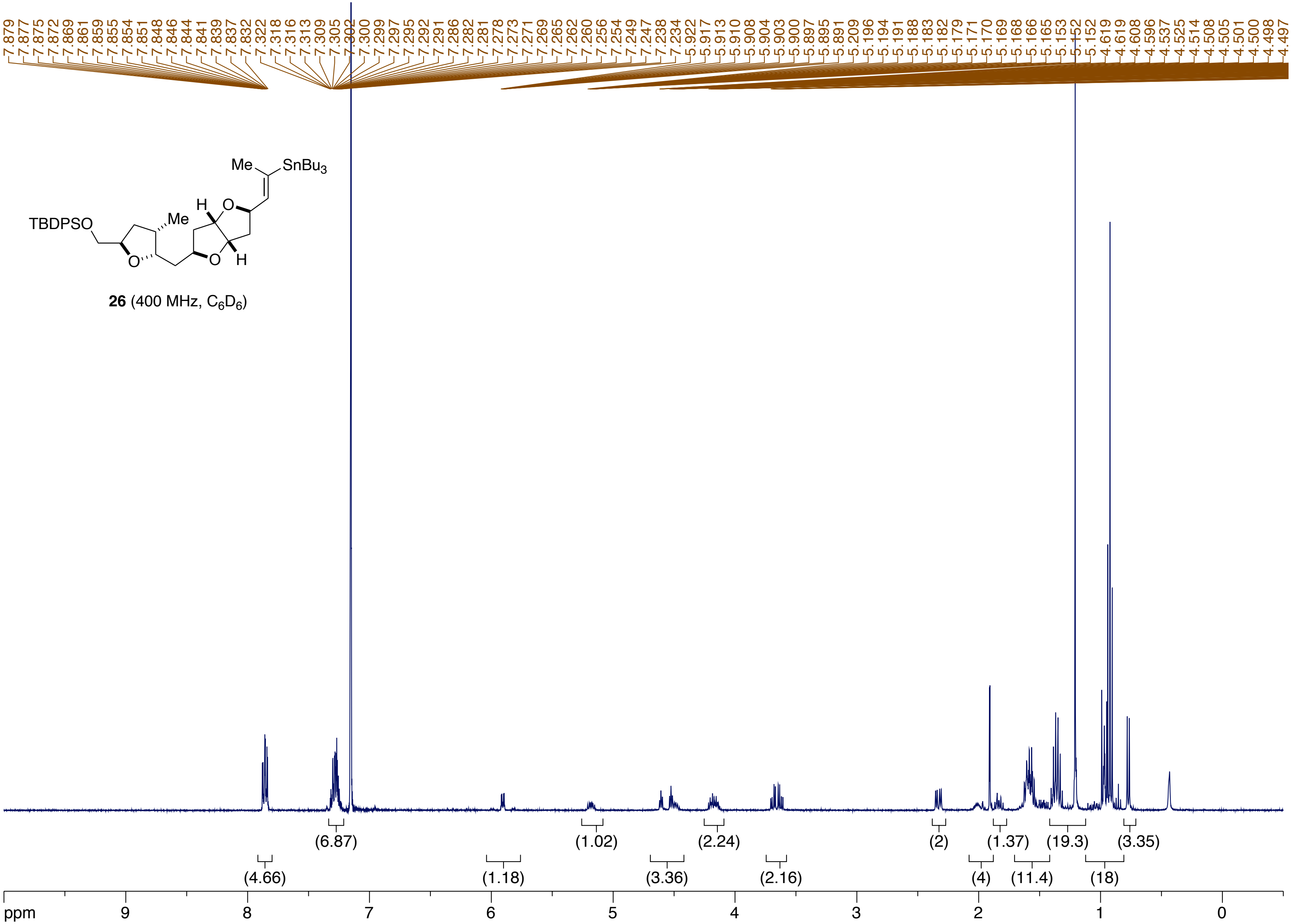
7.879 7.877 7.875 7.872 7.869 7.861 7.859 7.855 7.854 7.851 7.848 7.846 7.844 7.841 7.839 7.837 7.832 7.322 7.318 7.316 7.313 7.309 7.305 7.302 7.300 7.299 7.297 7.295 7.292 7.291 7.286 7.282 7.281 7.278 7.273 7.271 7.269 7.265 7.262 7.260 7.256 7.254 7.249 7.247 7.238 7.234 5.922 5.917 5.913 5.910 5.908 5.904 5.903 5.900 5.897 5.895 5.891 5.209 5.196 5.194 5.191 5.188 5.183 5.182 5.179 5.171 5.170 5.169 5.168 5.166 5.165 5.153 5.152 5.152 4.619 4.619 4.608 4.596 4.537 4.525 4.514 4.508 4.505 4.501 4.500 4.498 4.497
Me
SnBu3
TBDPSO
Me
H
O
O
O
H
26 (400 MHz, C6D6)
(6.87) (1.02) (2.24) (2) (1.37) (19.3) (3.35)
(4.66) (1.18) (3.36) (2.16) (4) (11.4) (18)
ppm
9
8
7
6
5
4
3
2
1
0

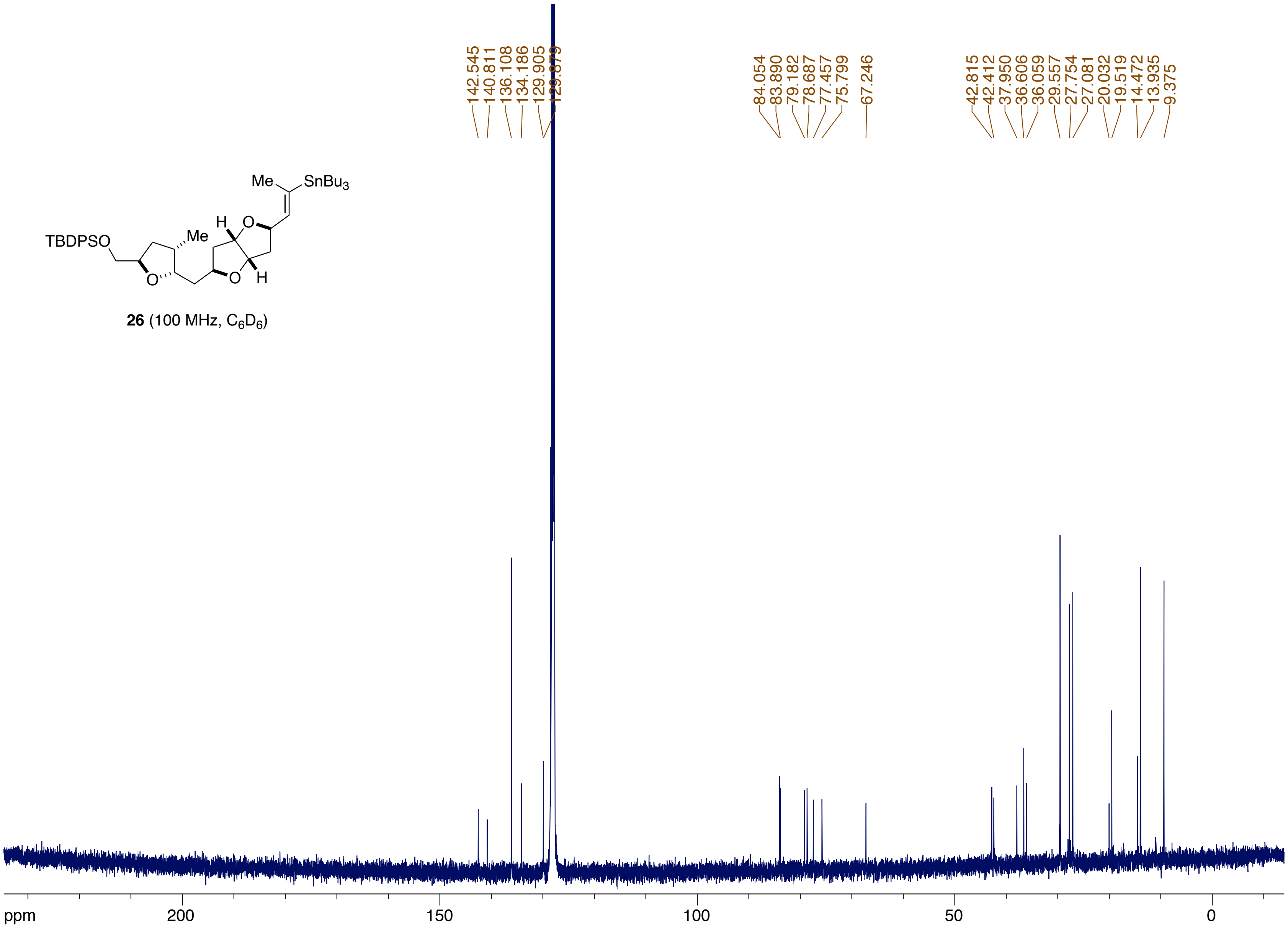

**26** (100 MHz, $C_6D_6$)

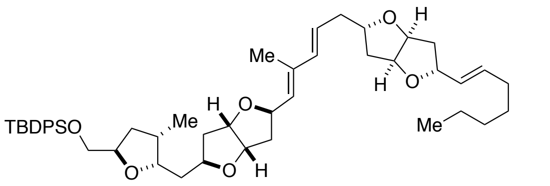

**S21** (500 MHz, $CDCl_3$)

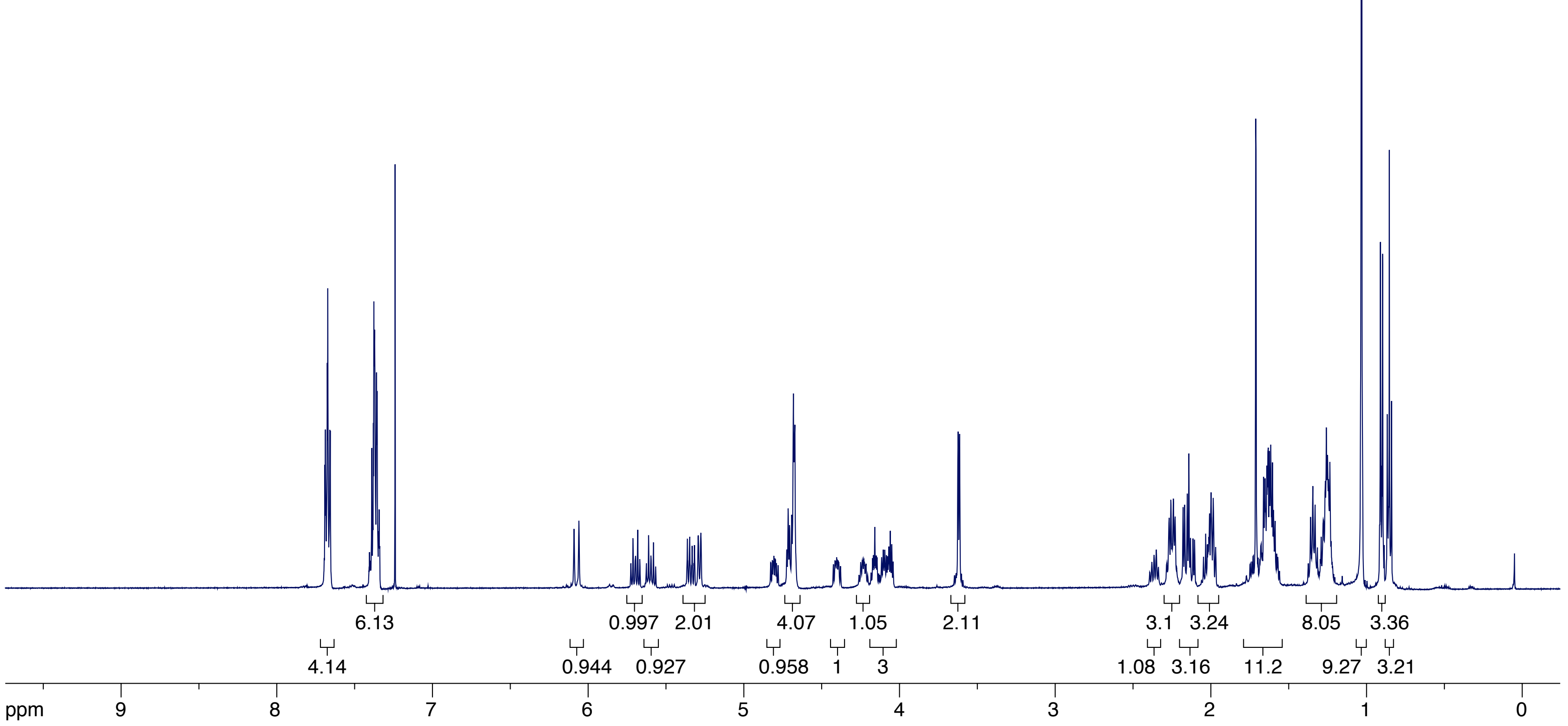

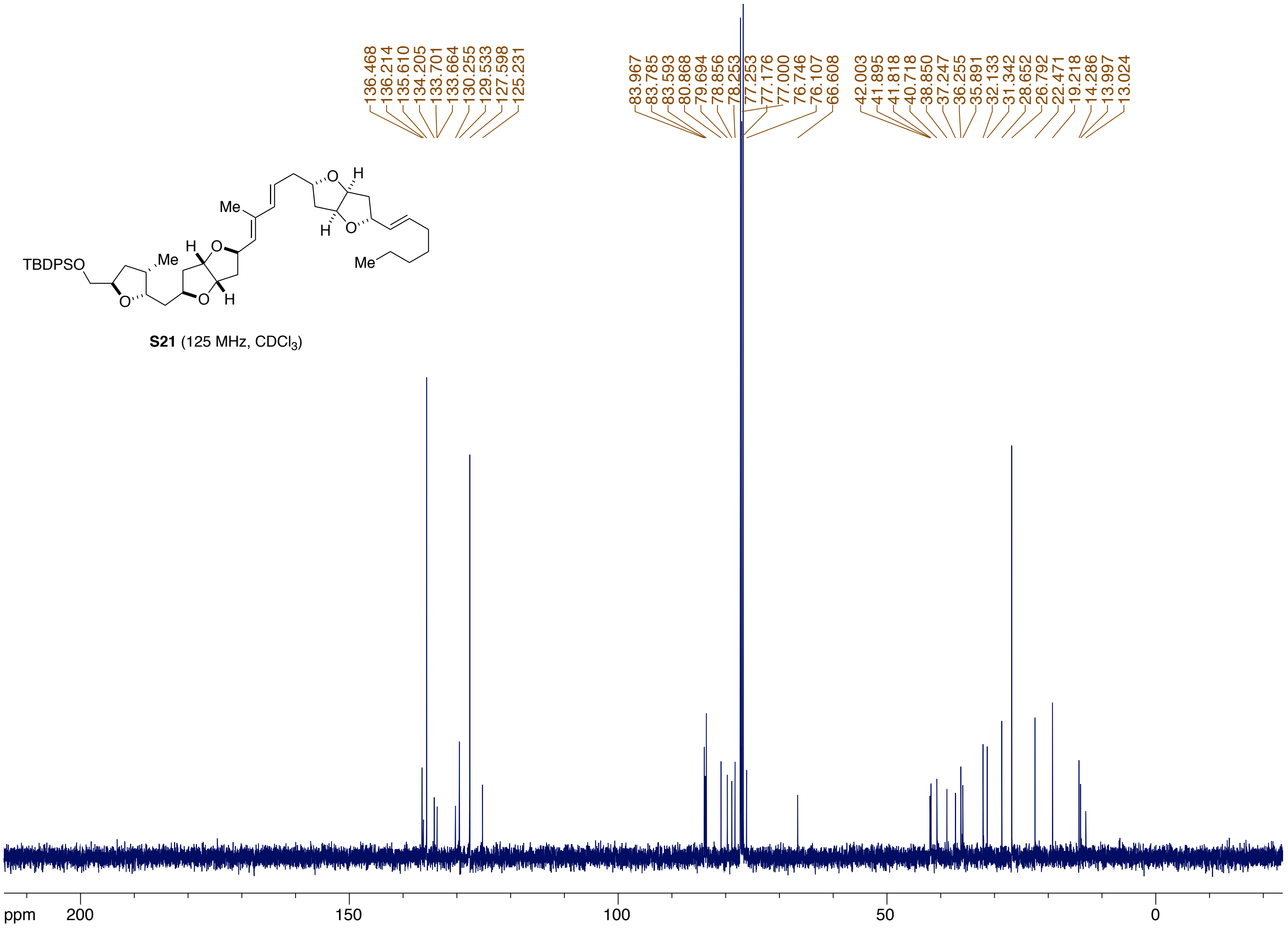


**S21** (125 MHz, $CDCl_3$)

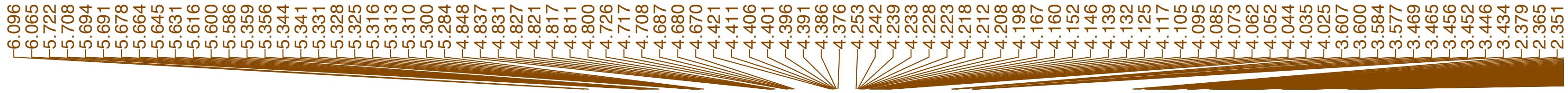


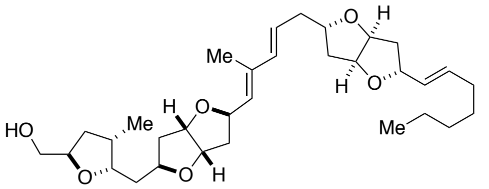


**S22** (500 MHz, $CDCl_3$)

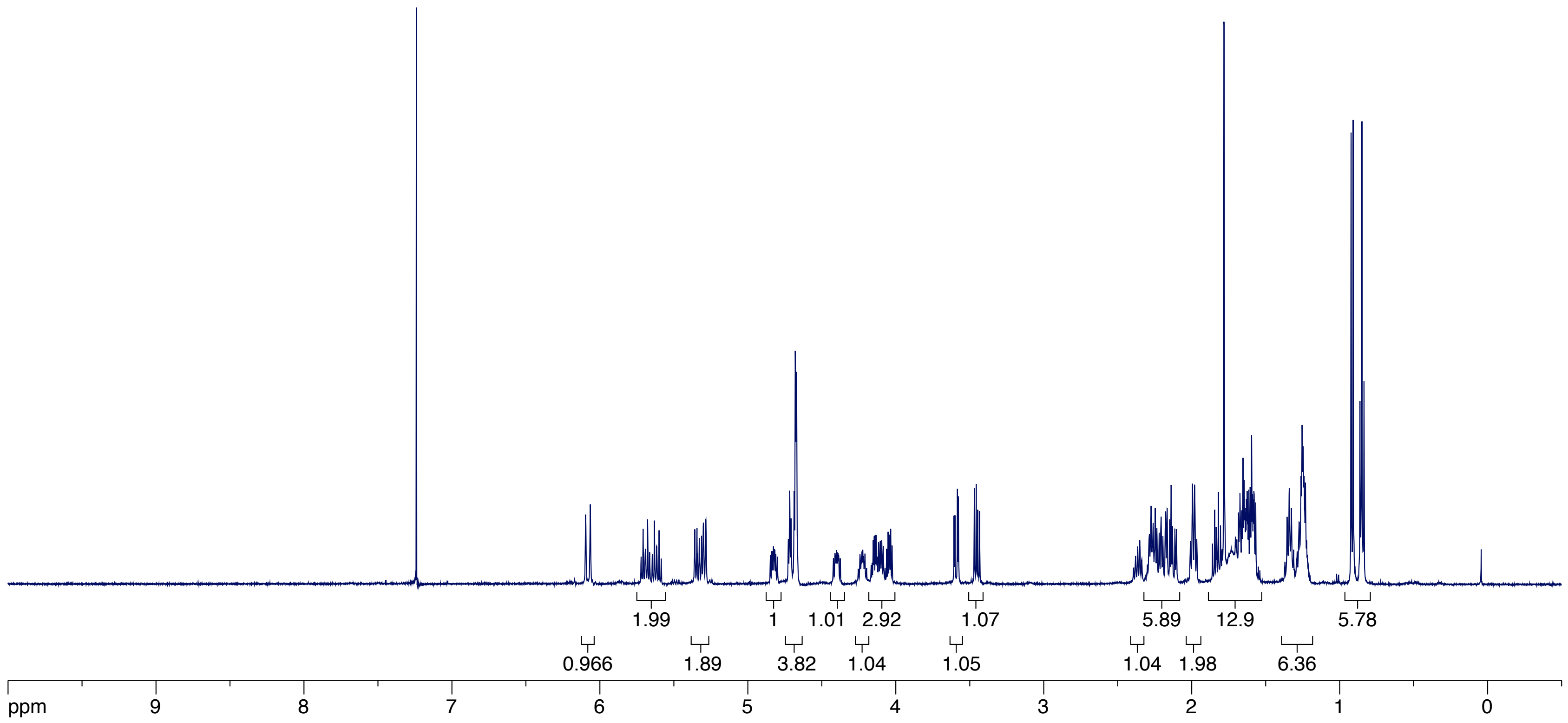

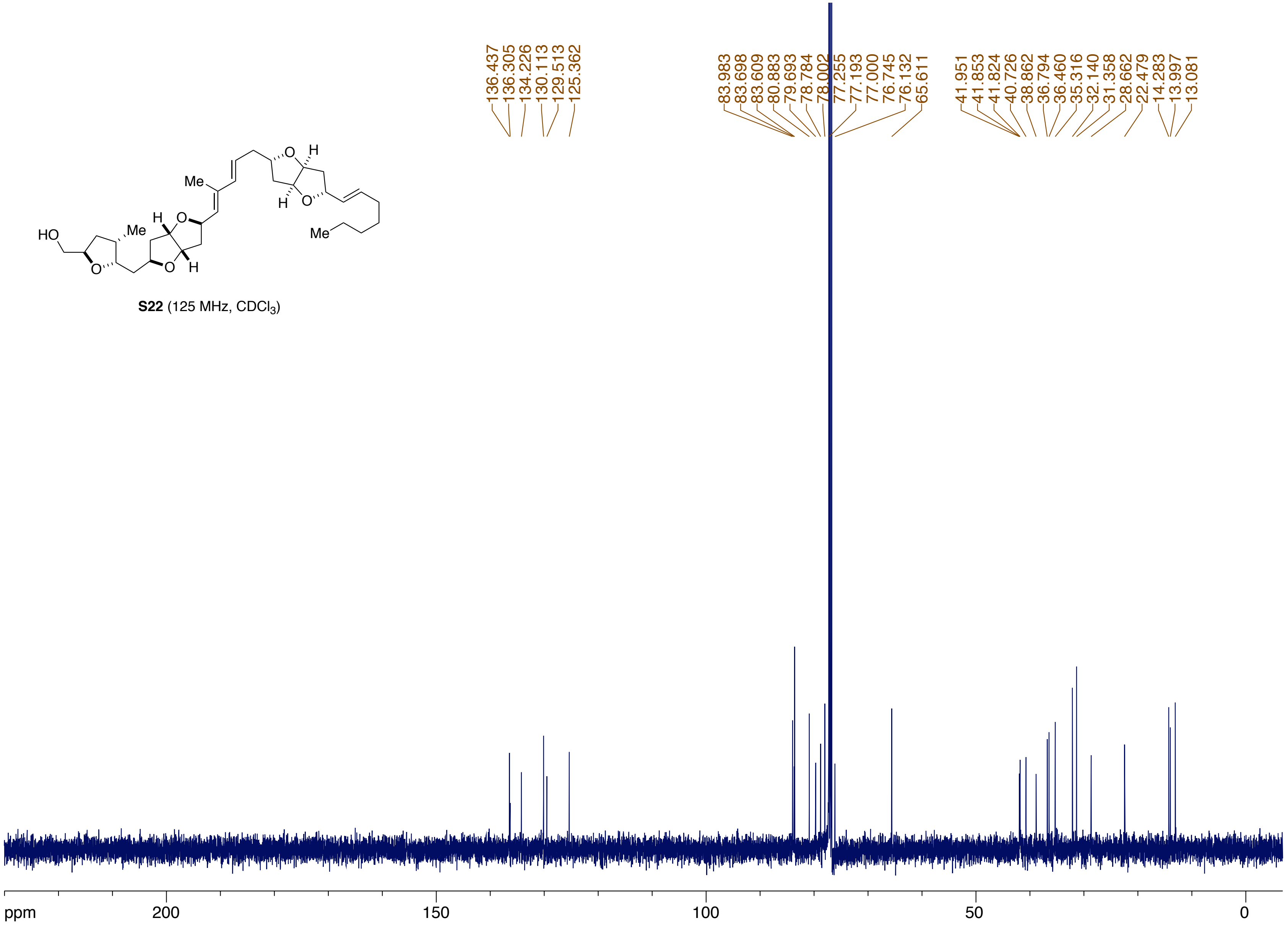


**S22** (125 MHz, $CDCl_3$)

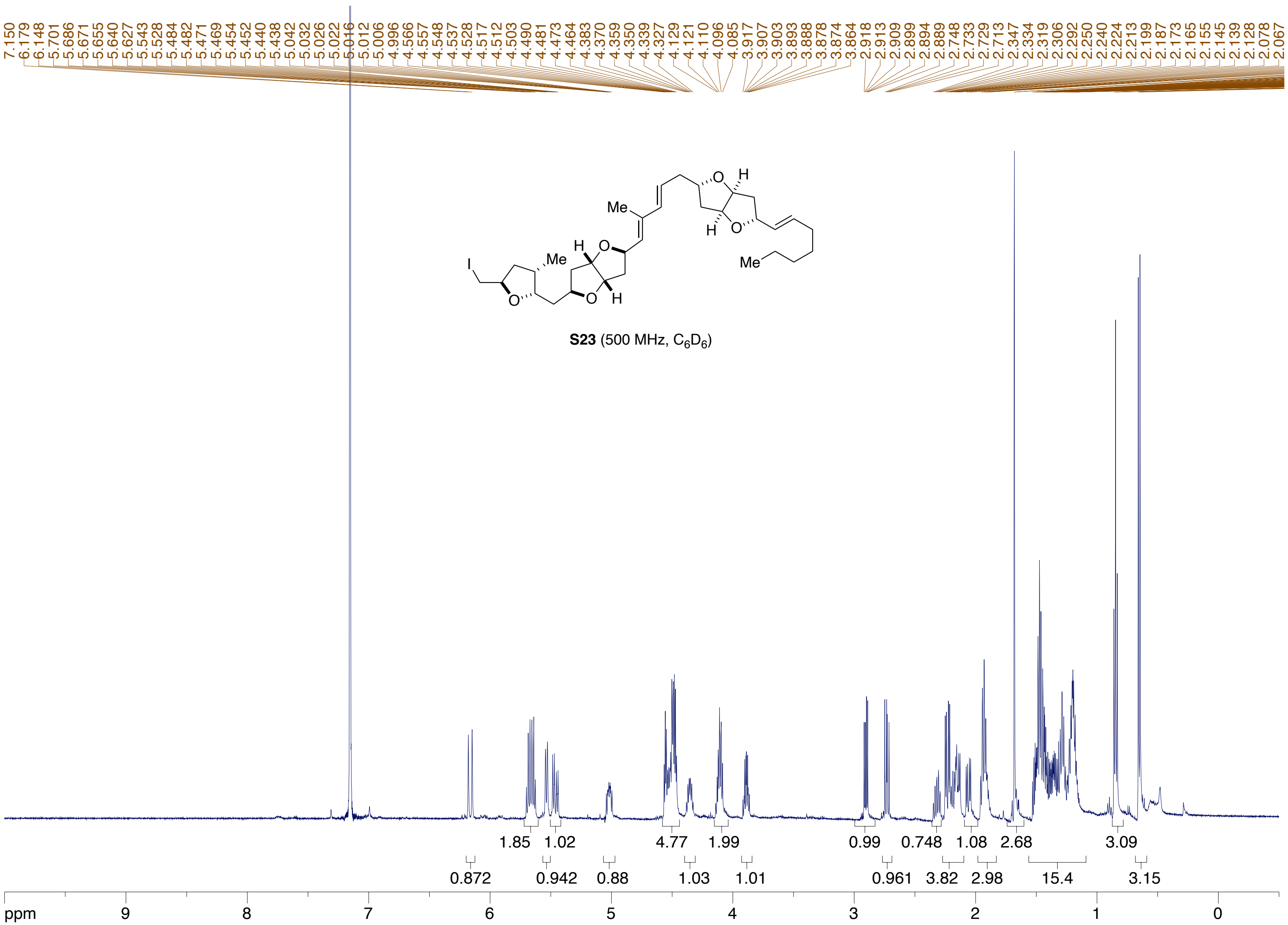


**S23** (500 MHz, $C_6D_6$)

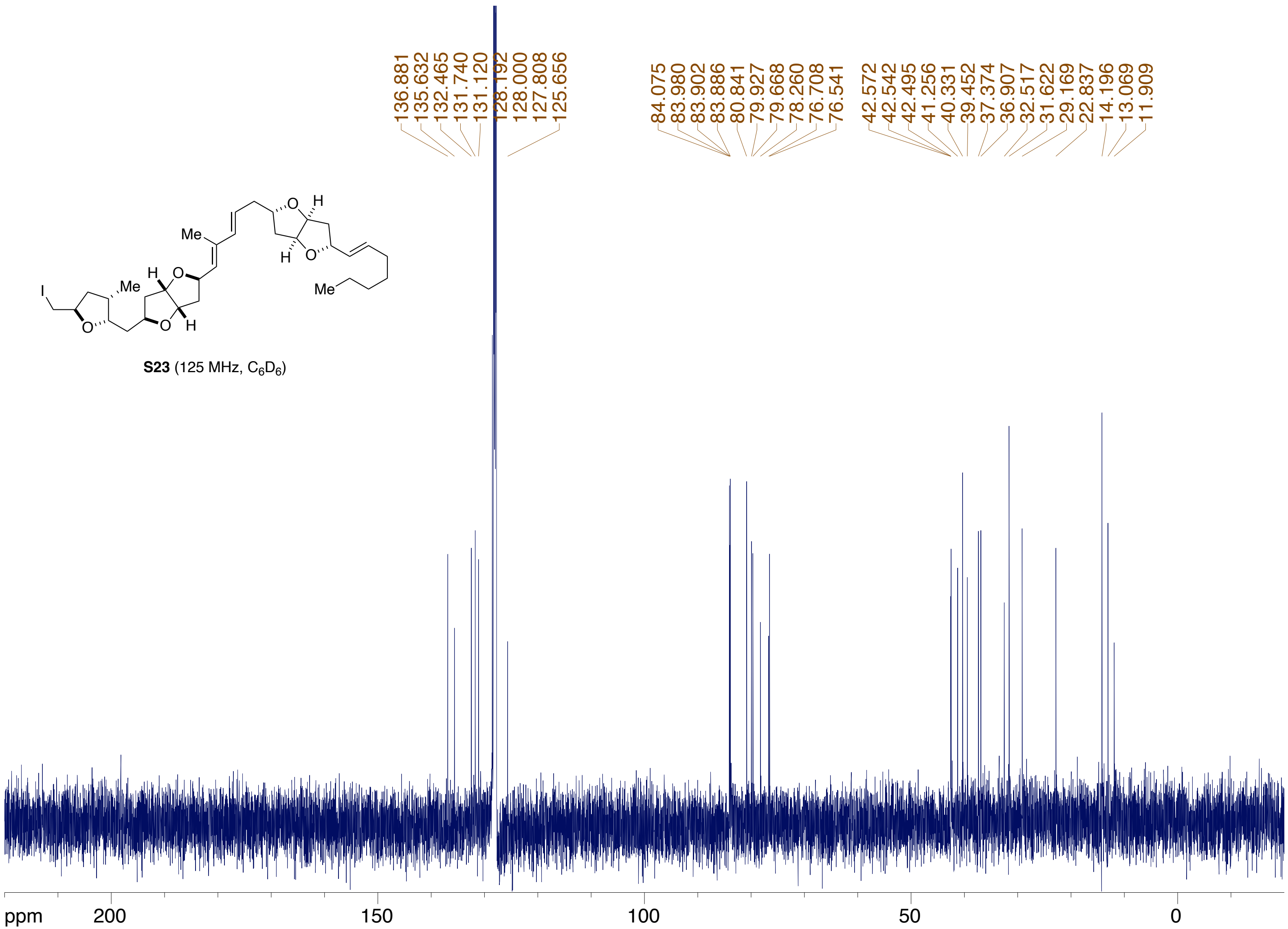
136.881
135.632
132.465
131.740
131.120
128.192
128.000
127.808
125.656
84.075
83.980
83.902
83.886
80.841
79.927
79.668
78.260
76.708
76.541
42.572
42.542
42.495
41.256
40.331
39.452
37.374
36.907
32.517
31.622
29.169
22.837
14.196
13.069
11.909
Me
I
Me
H
O
O
H
H
O
O
H
Me
**S23** (125 MHz, $C_6D_6$)
ppm
200
150
100
50
0

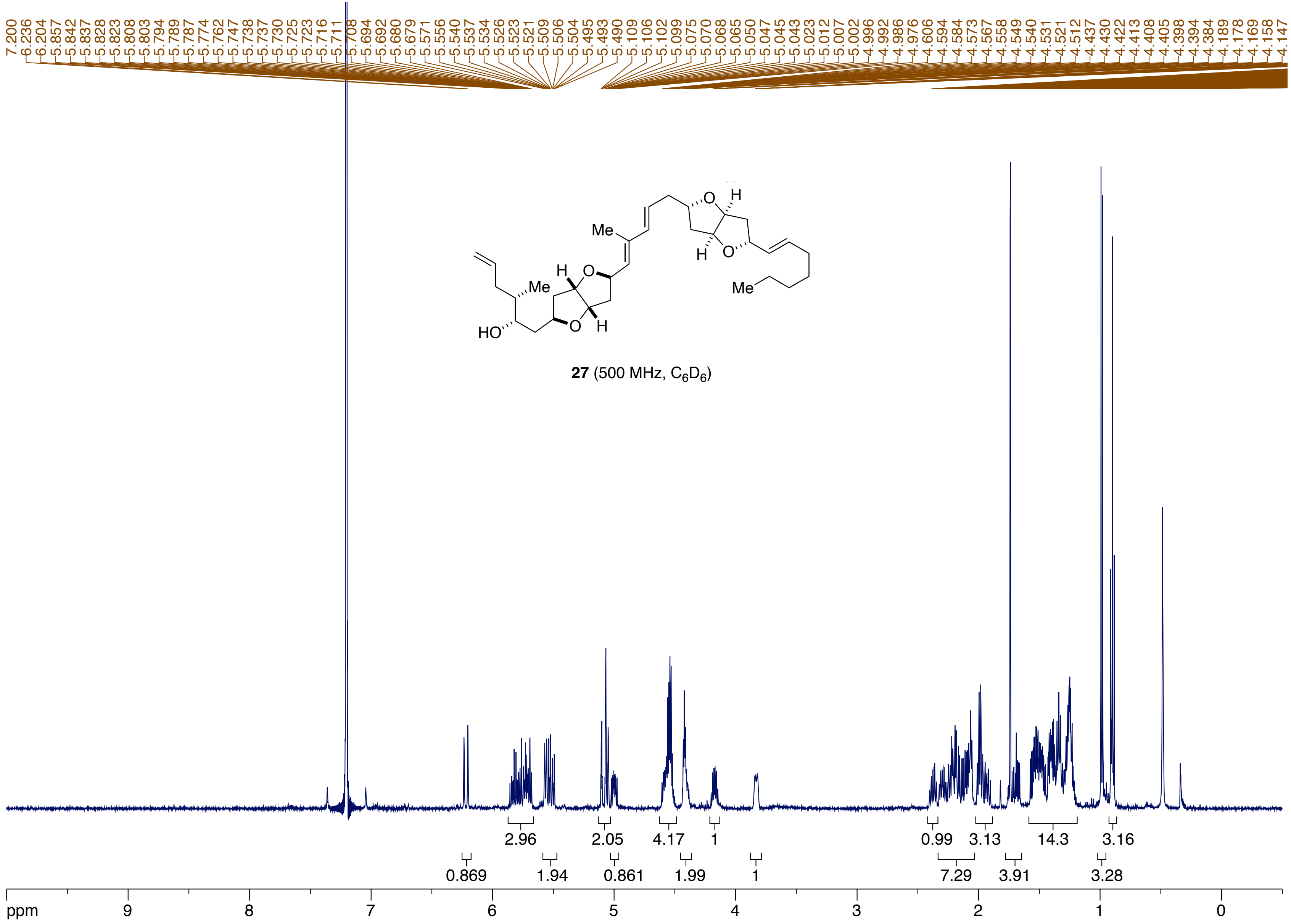


**27** (500 MHz, $C_6D_6$)

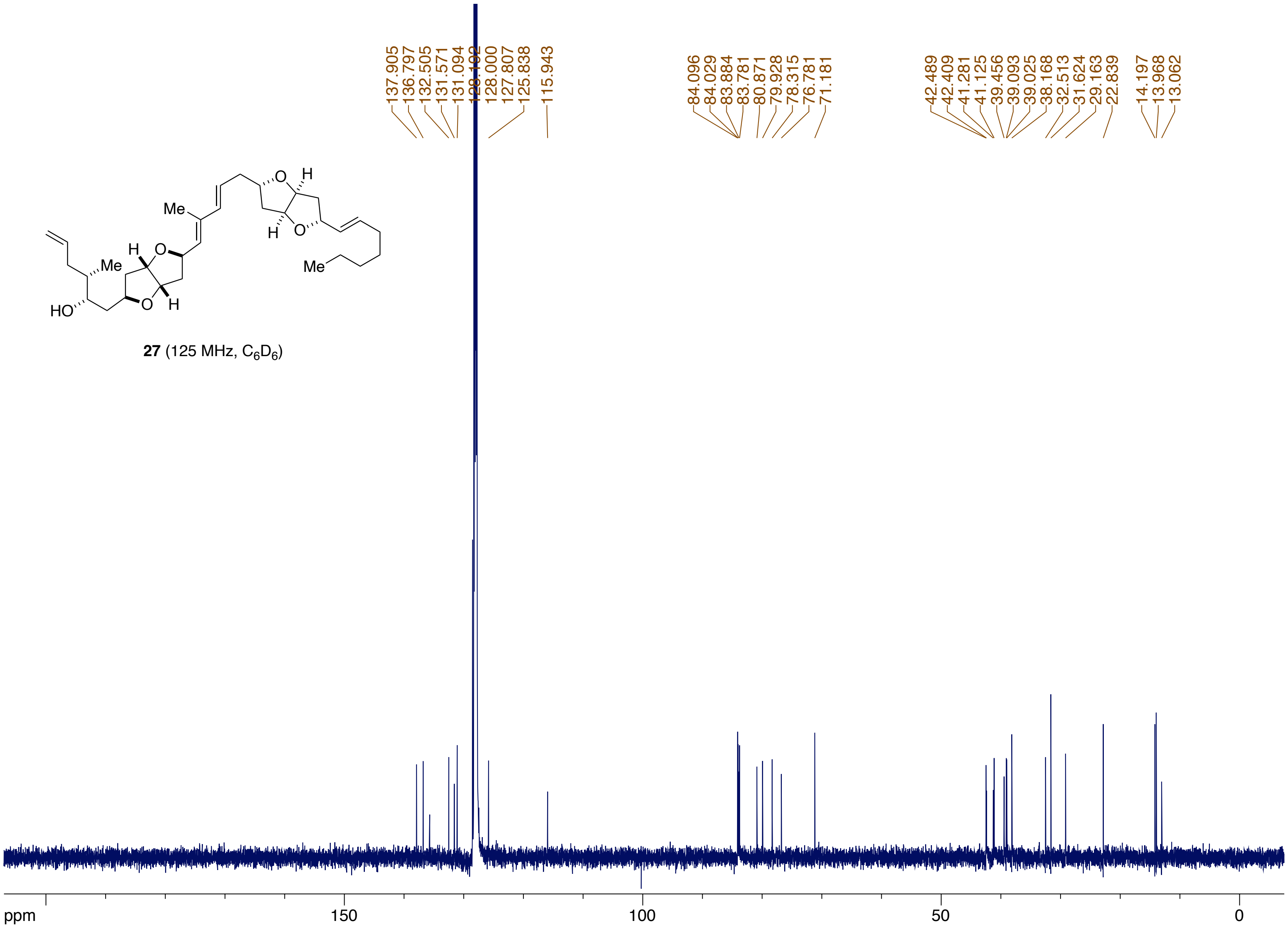


**27** (125 MHz, $C_6D_6$)

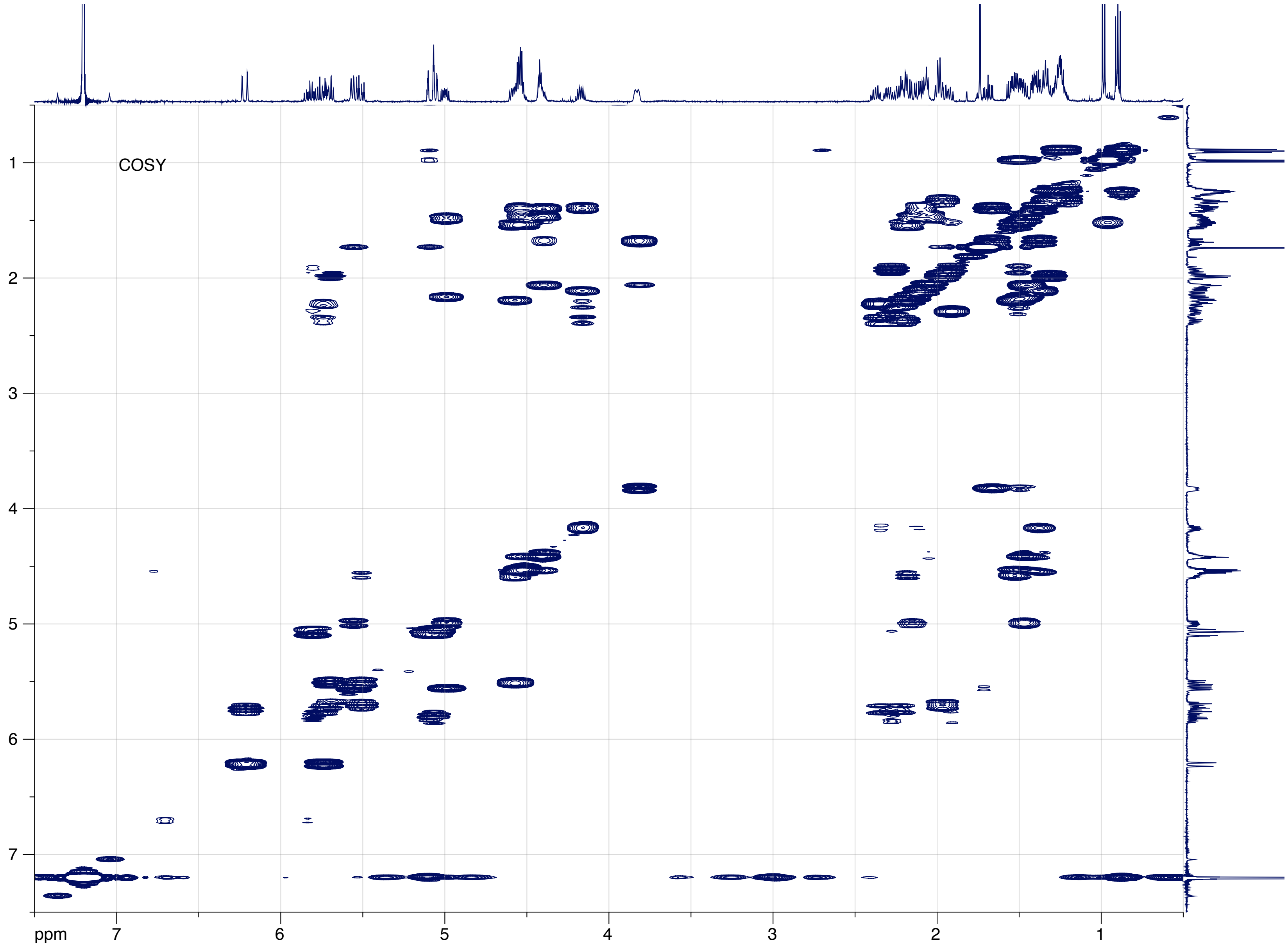
COSY
ppm
7
6
5
4
3
2
1

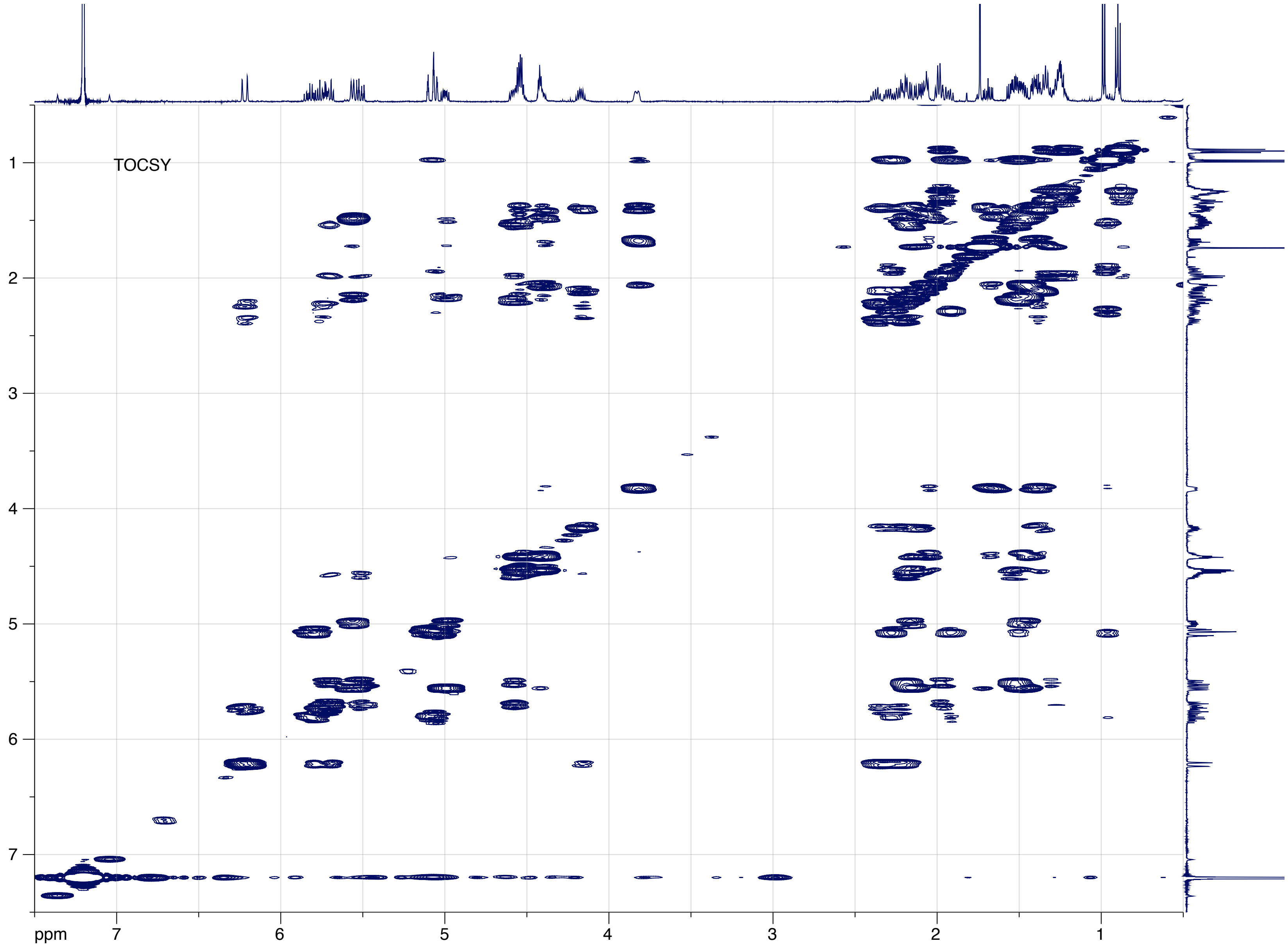
TOCSY
ppm
7
6
5
4
3
2
1

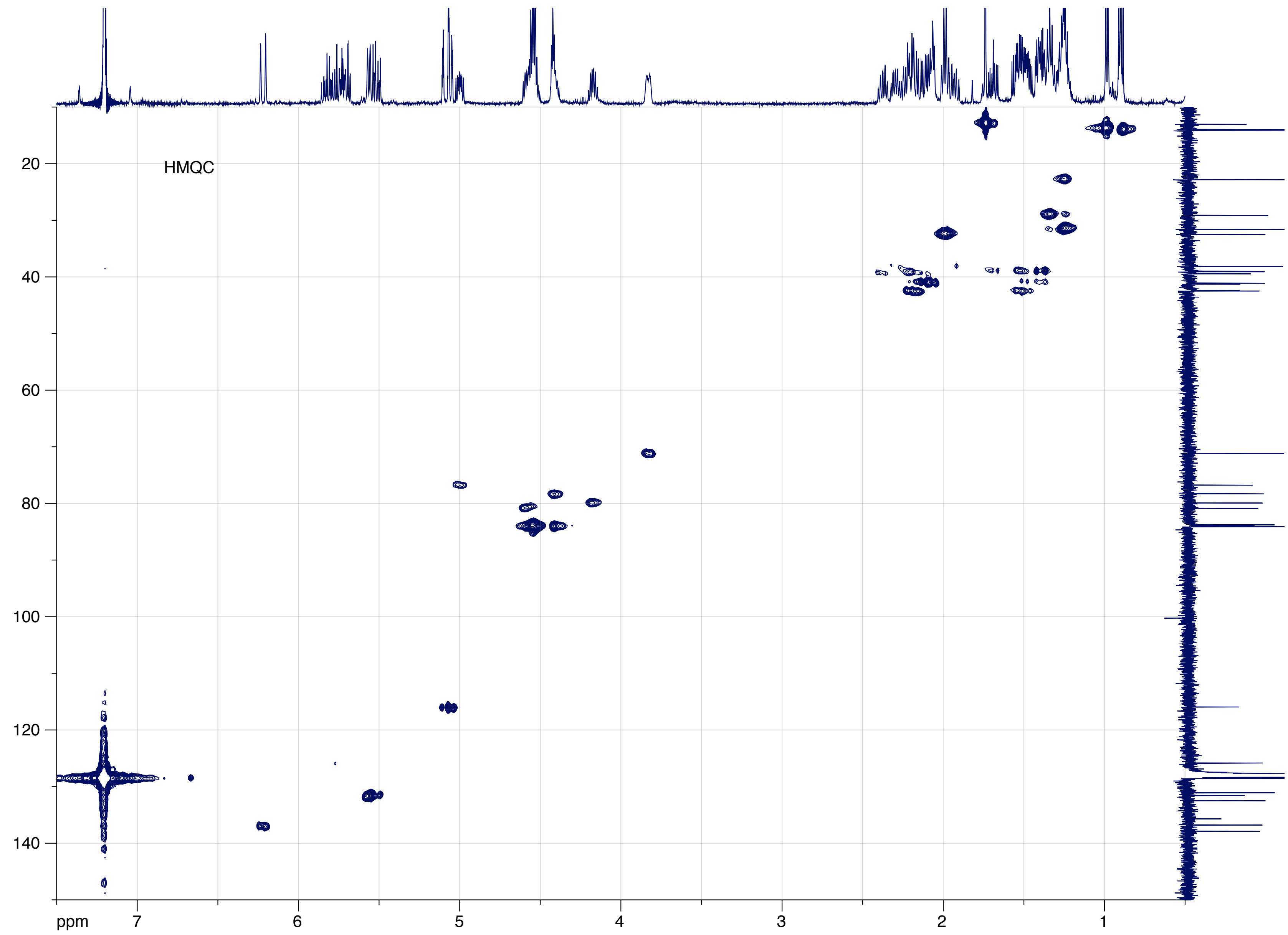
HMQC
20
40
60
80
100
120
140
ppm
7
6
5
4
3
2
1

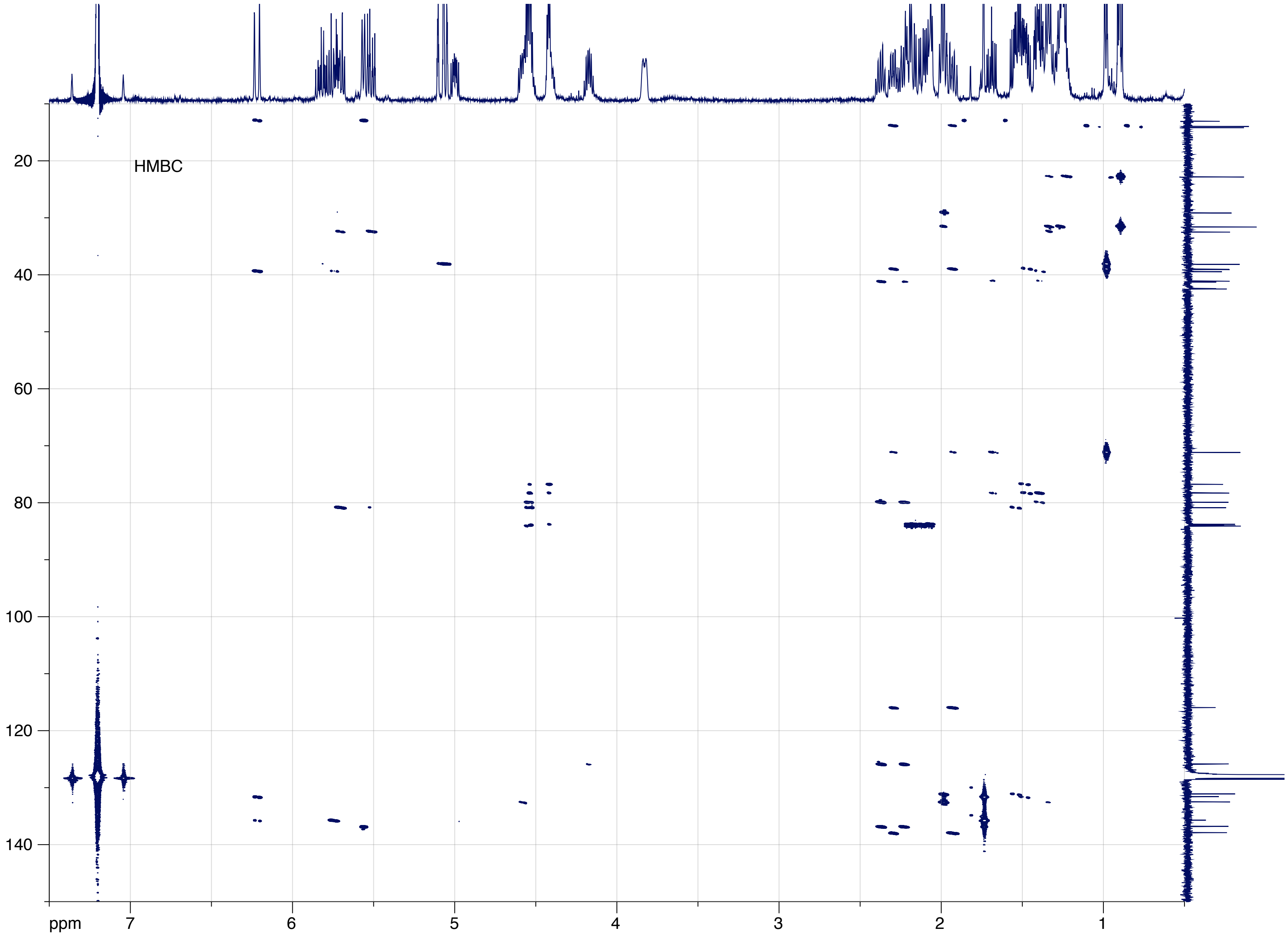
HMBC
ppm
20
40
60
80
100
120
140
7
6
5
4
3
2
1

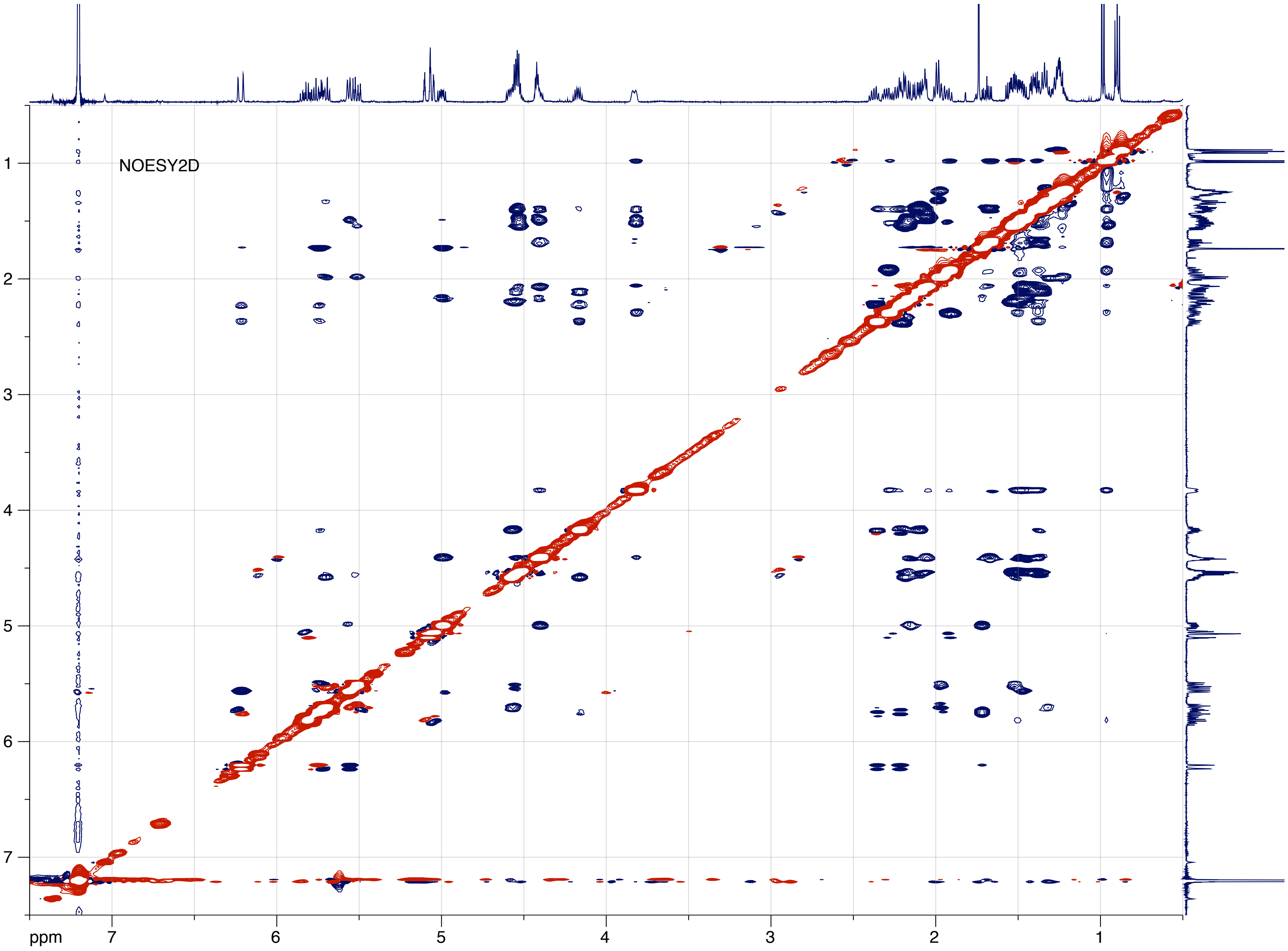


S201

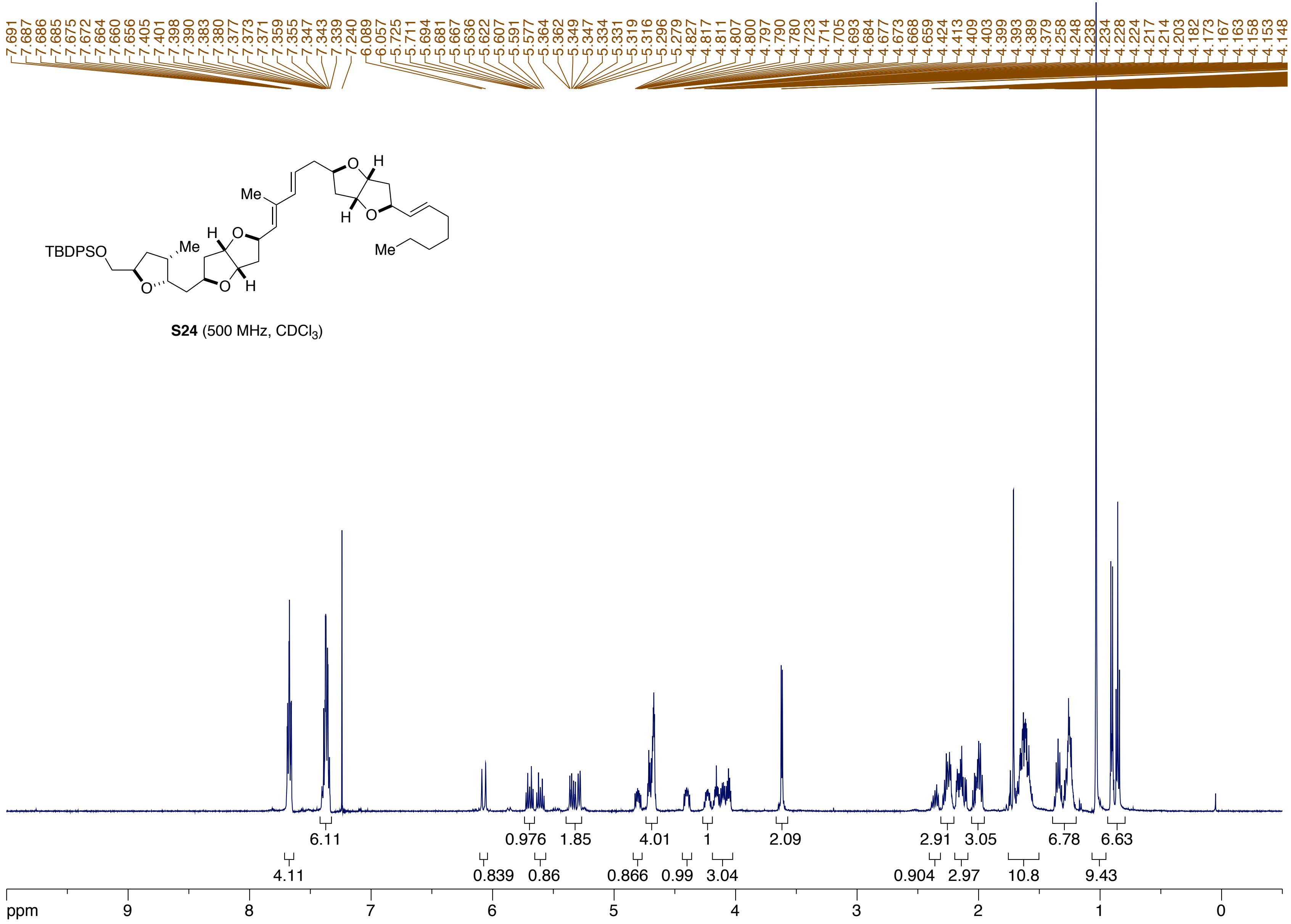

**S24** (500 MHz, $CDCl_3$)

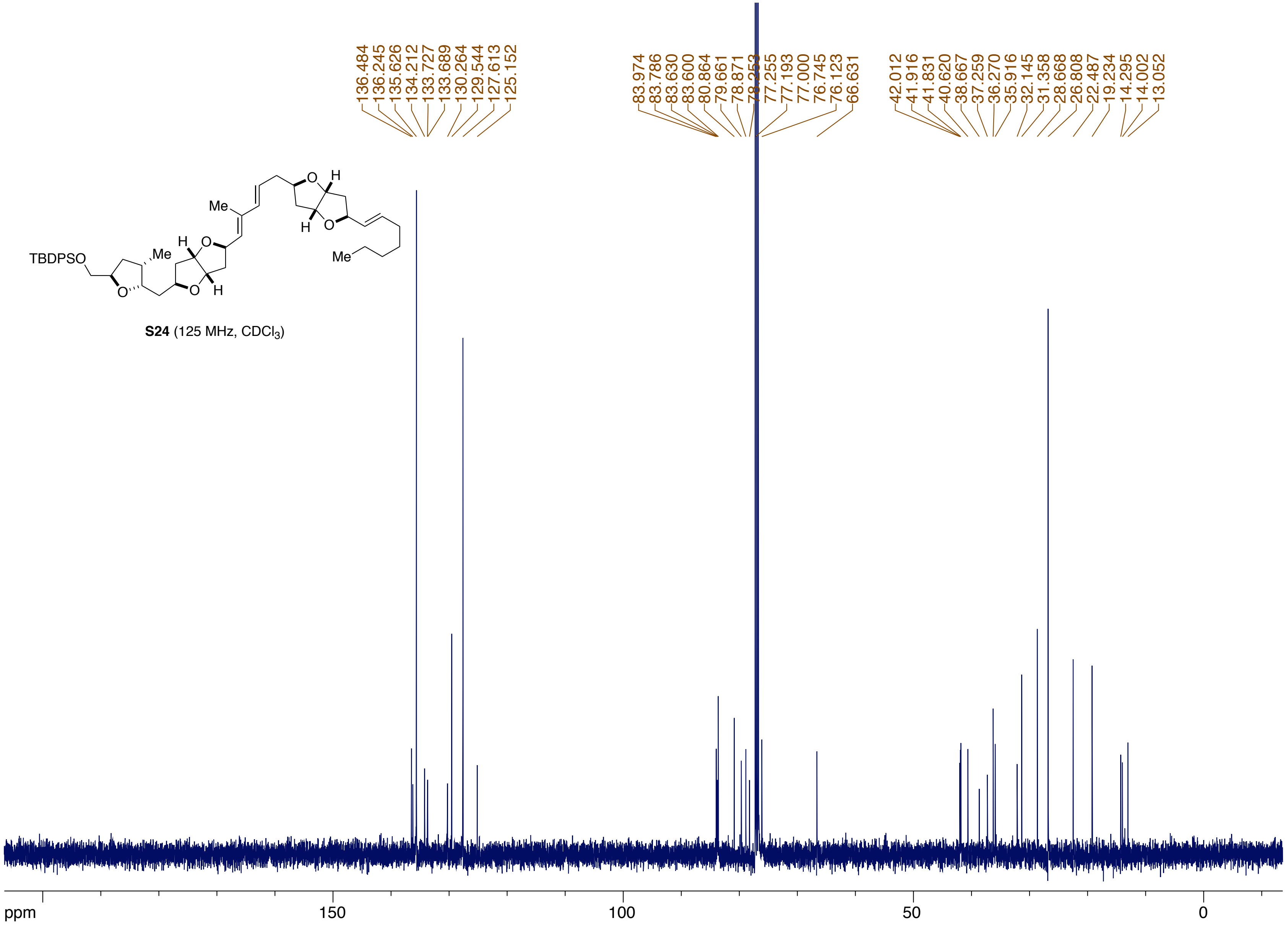
136.484
136.245
135.626
134.212
133.727
133.689
130.264
129.544
127.613
125.152
83.974
83.786
83.630
83.600
80.864
79.661
78.871
78.253
77.255
77.193
77.000
76.745
76.123
66.631
42.012
41.916
41.831
40.620
38.667
37.259
36.270
35.916
32.145
31.358
28.668
26.808
22.487
19.234
14.295
14.002
13.052
TBDPSO
Me
H
O
H
Me
**S24** (125 MHz, $CDCl_3$)
ppm
150
100
50
0

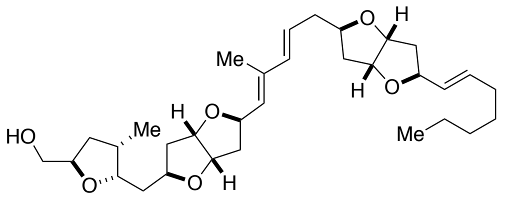


**S25** (500 MHz, $CDCl_3$)

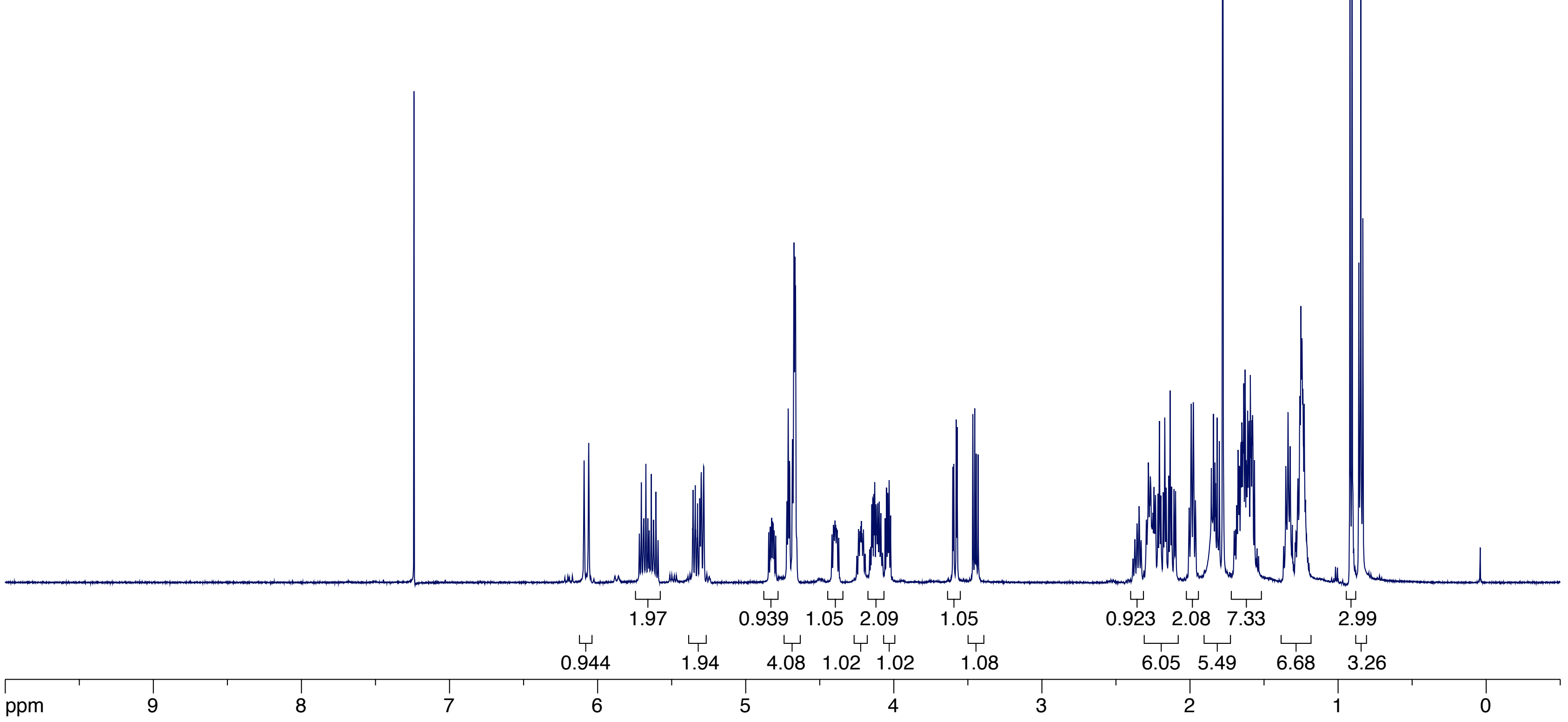

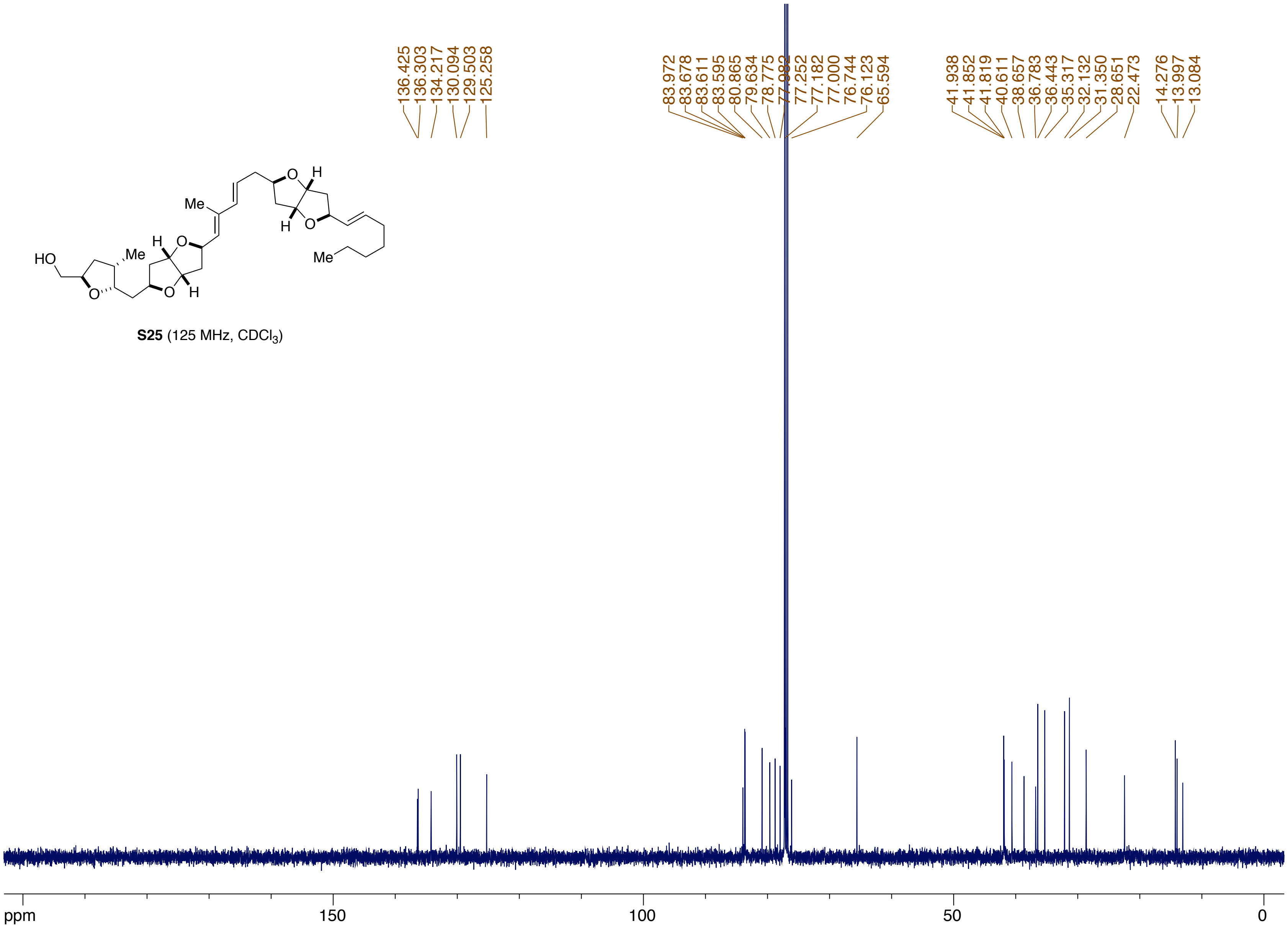


**S25** (125 MHz, $CDCl_3$)

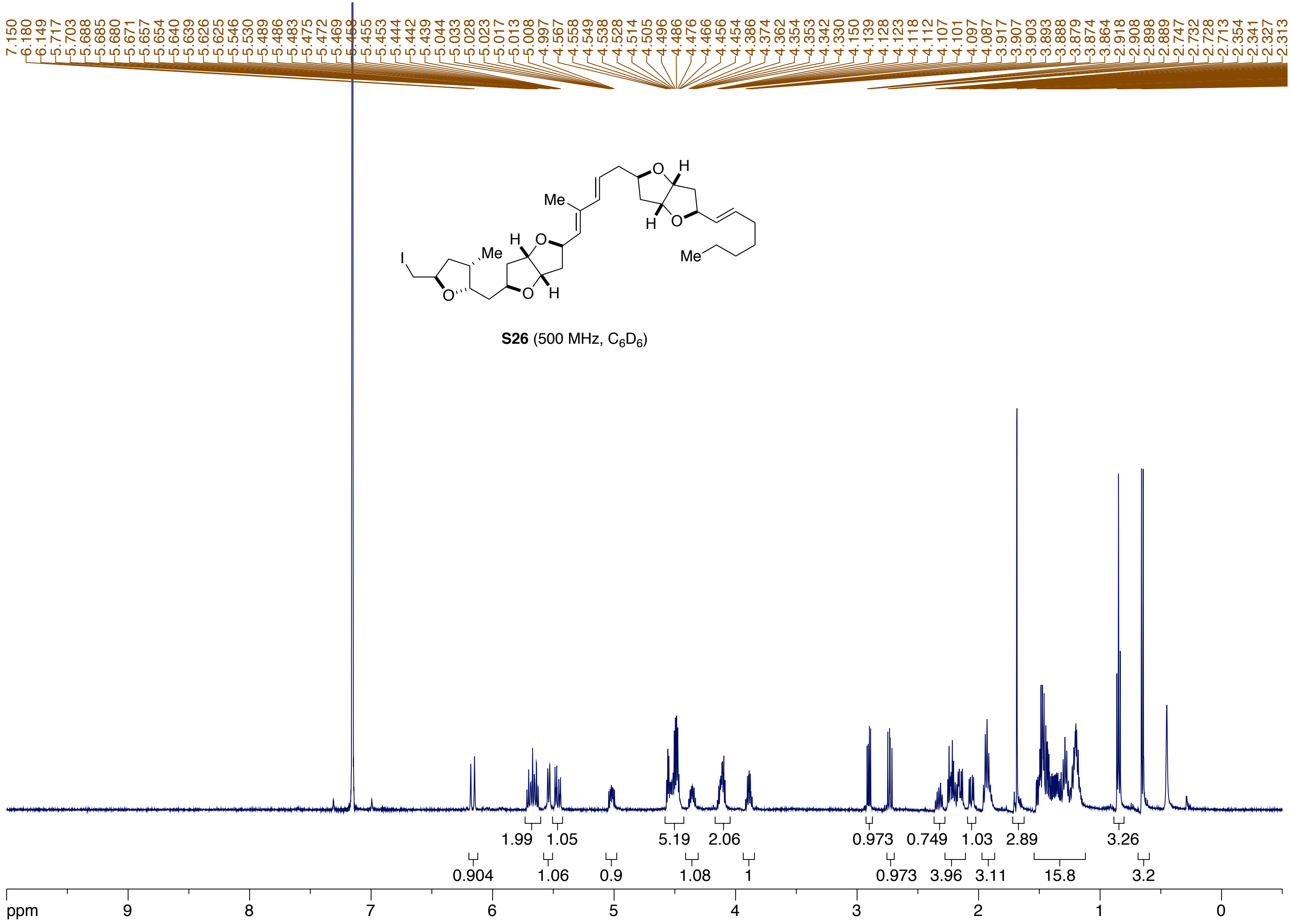


**S26** (500 MHz, $C_6D_6$)

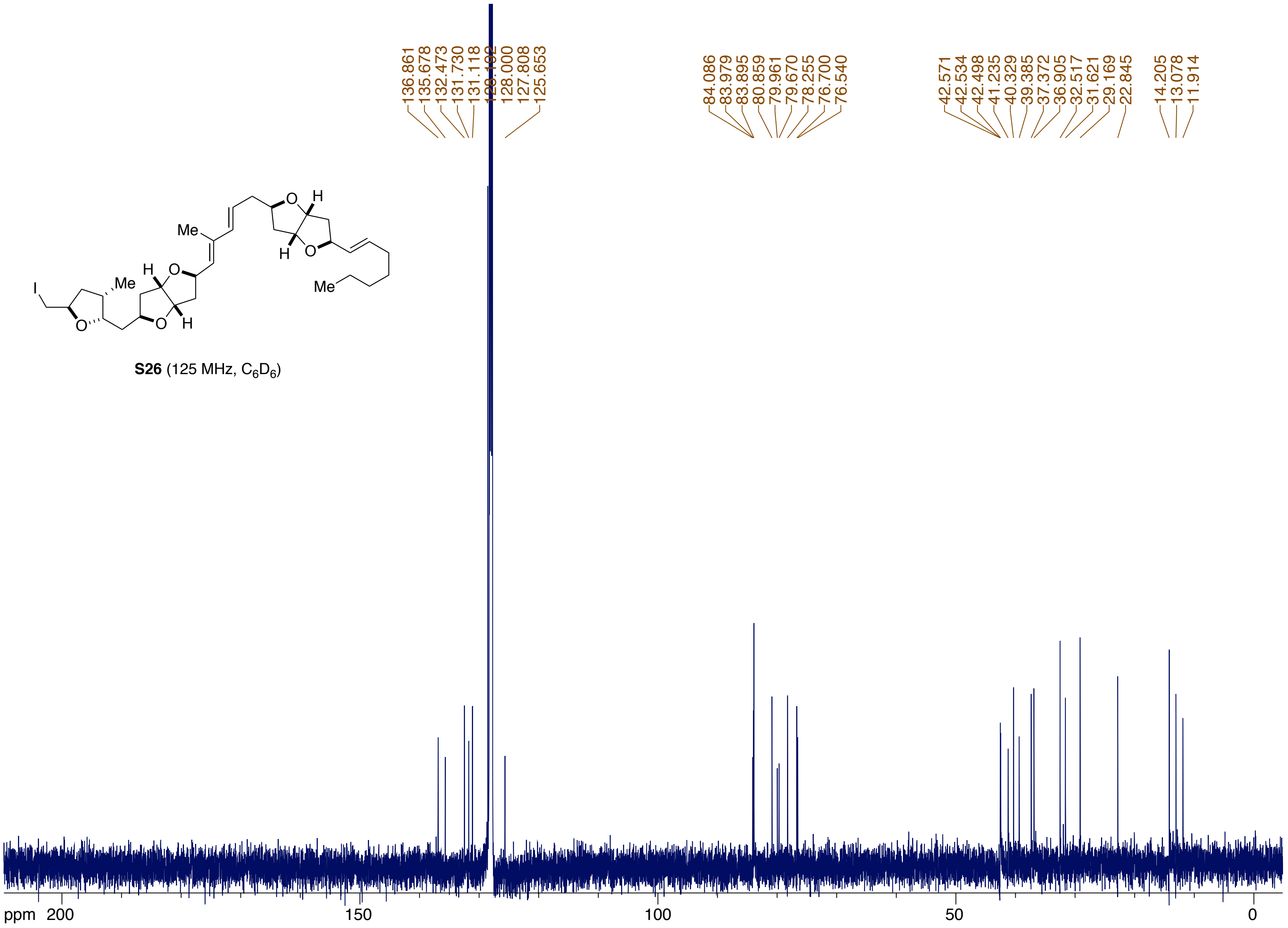


**S26** (125 MHz, $C_6D_6$)

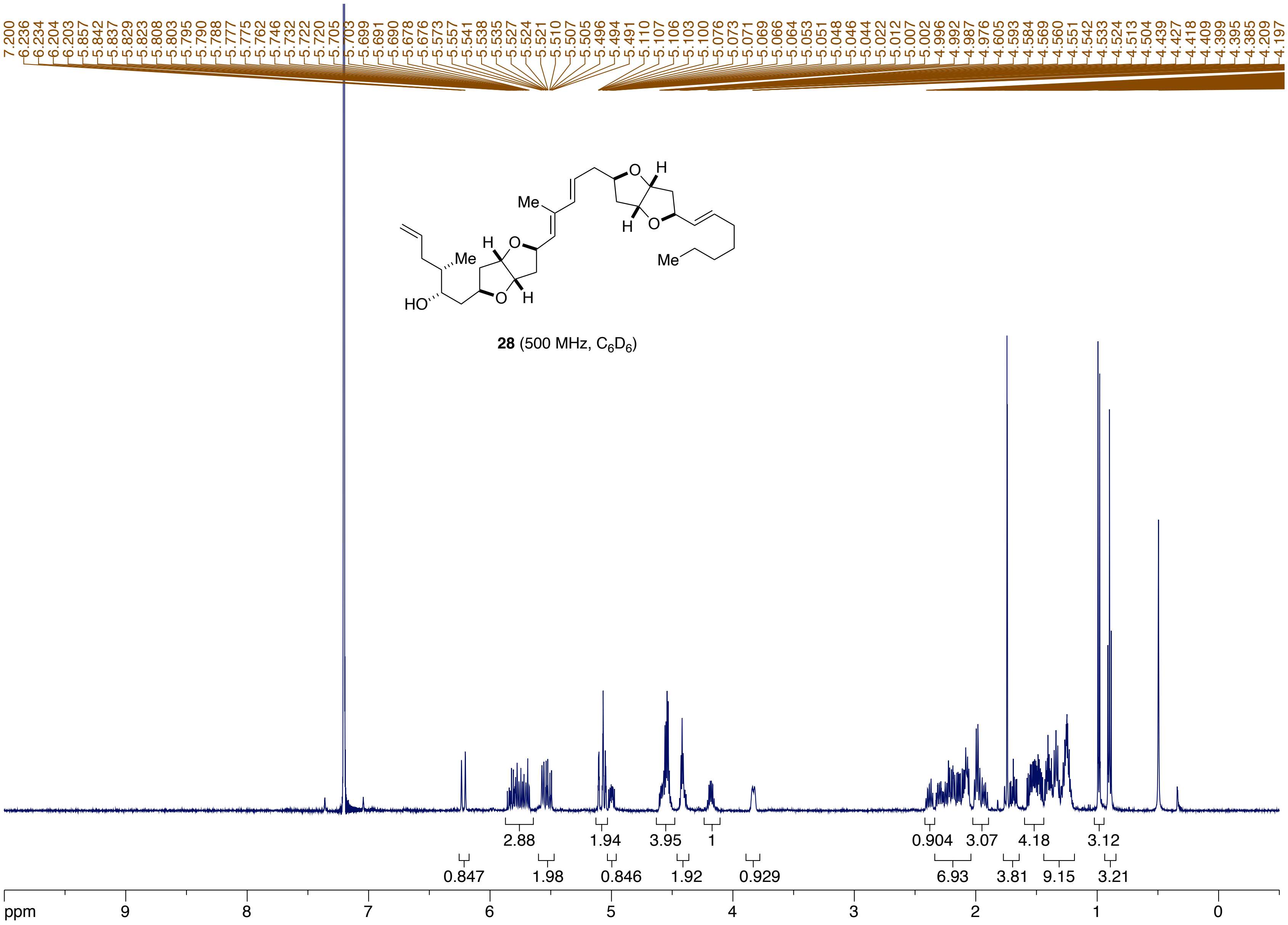
28 (500 MHz, C6D6)
ppm

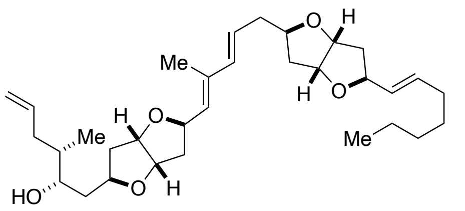


**28** (125 MHz, $C_6D_6$)

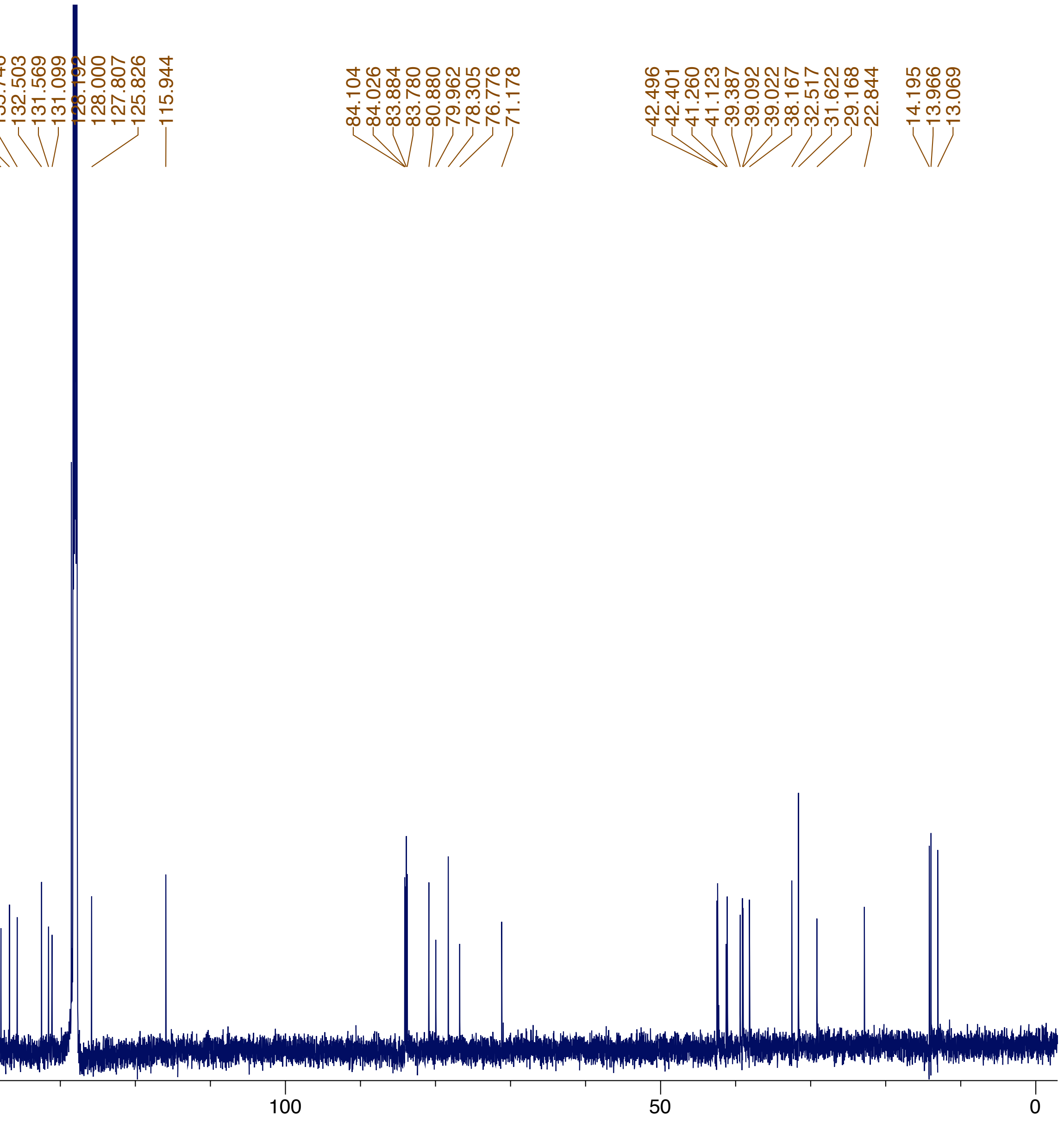

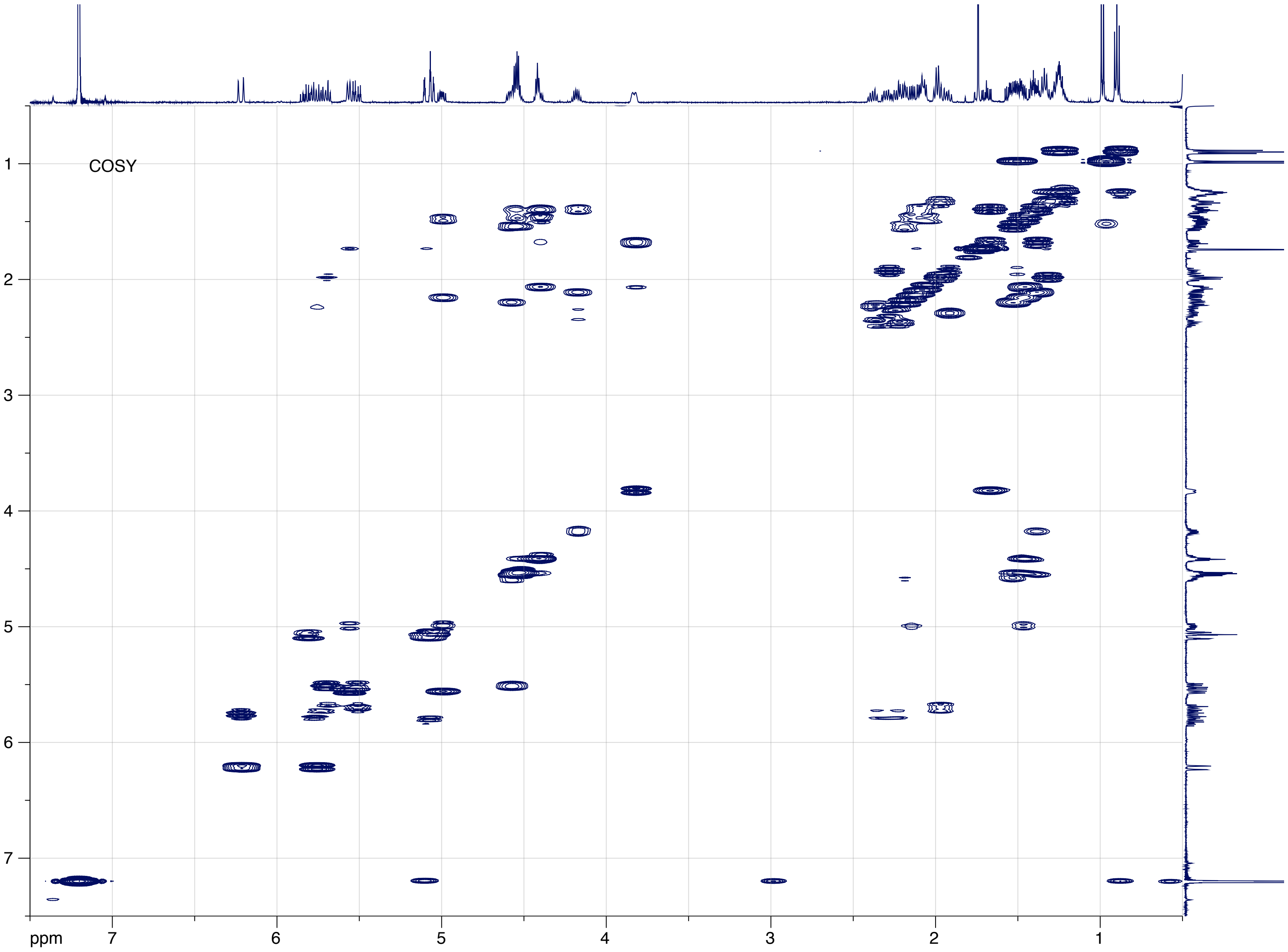
COSY
ppm
7
6
5
4
3
2
1
1
2
3
4
5
6
7

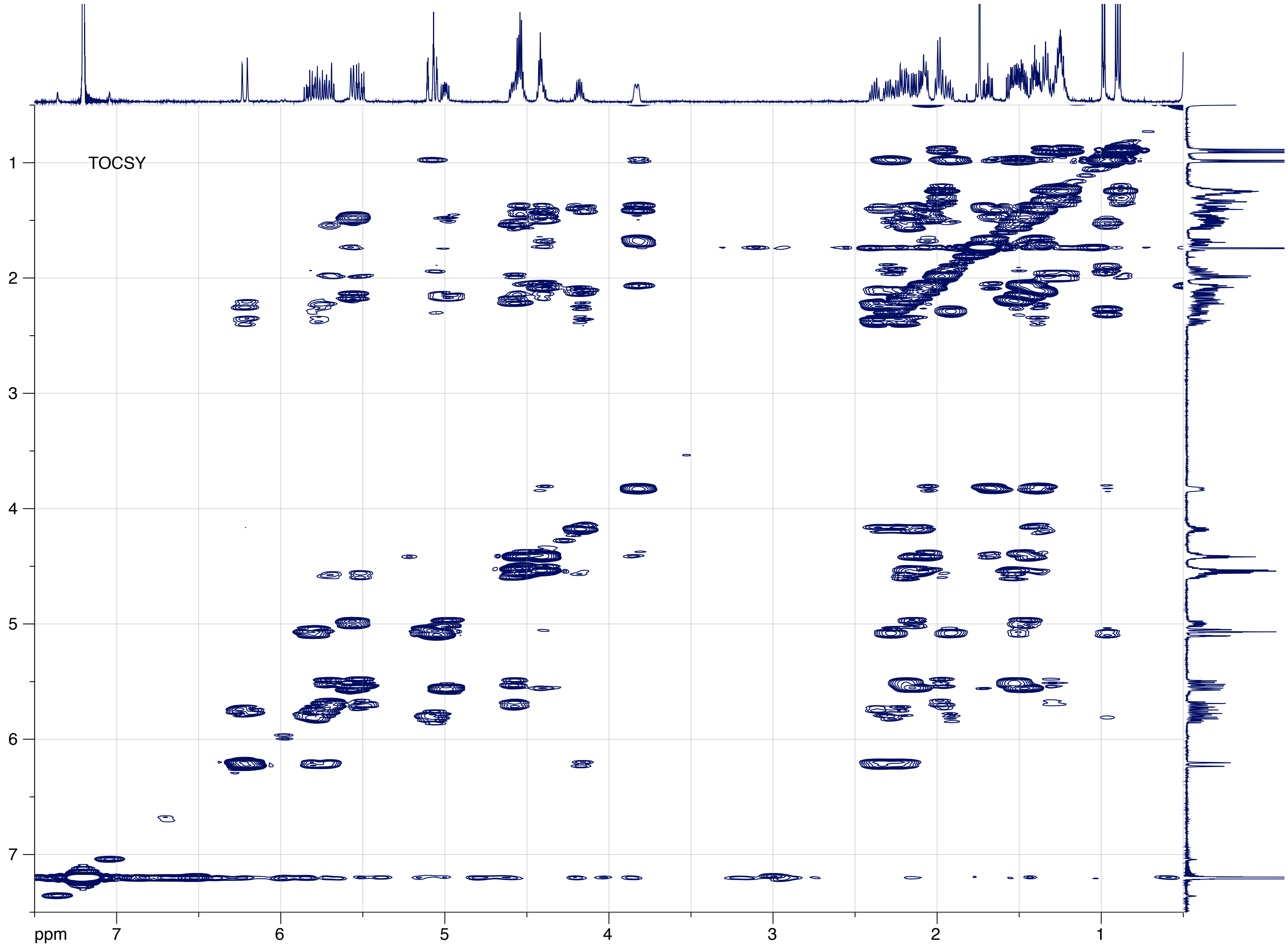
TOCSY
ppm
7
6
5
4
3
2
1

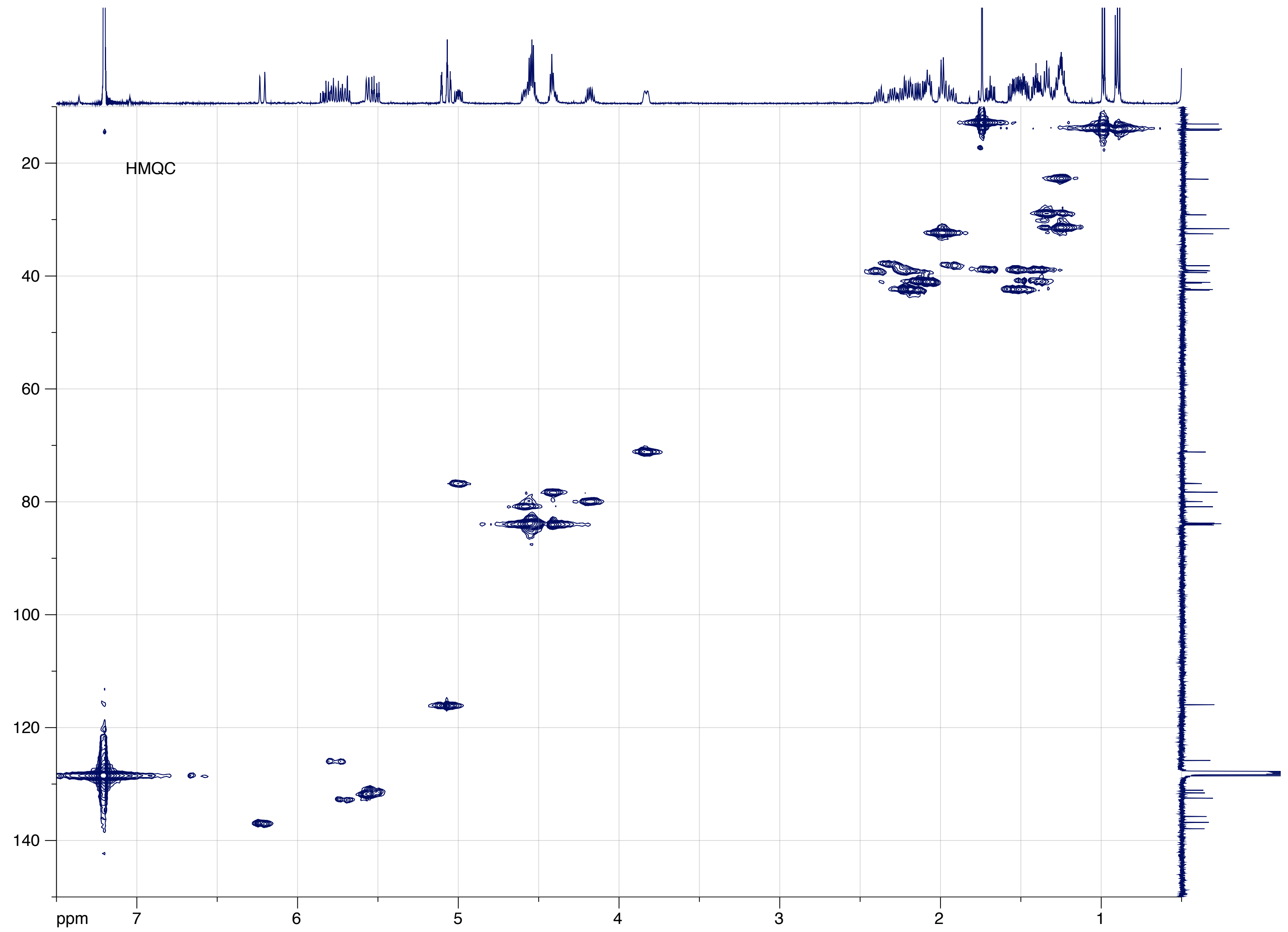
HMQC
ppm
20
40
60
80
100
120
140
7
6
5
4
3
2
1

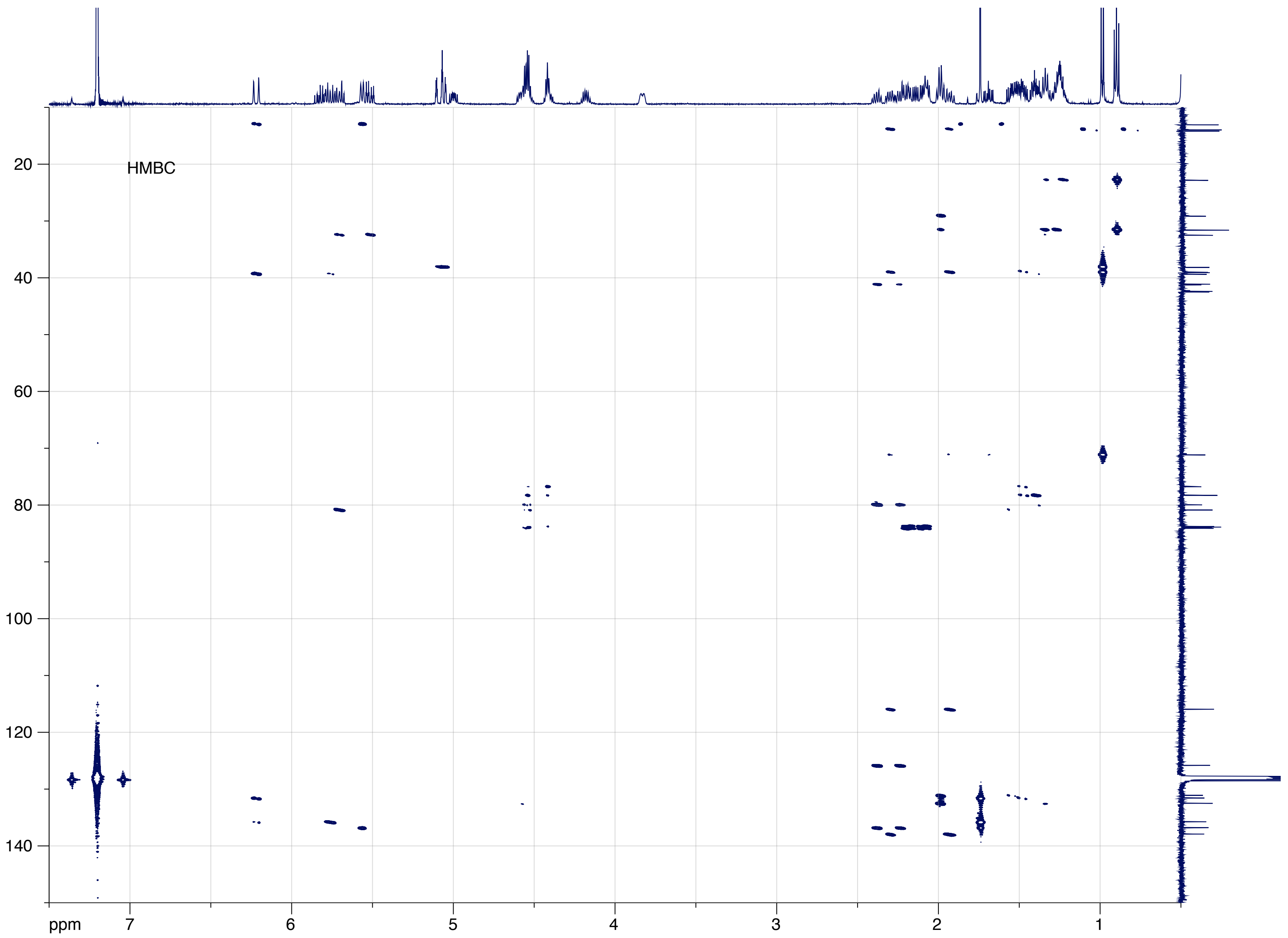
HMBC
ppm
7
6
5
4
3
2
1
20
40
60
80
100
120
140

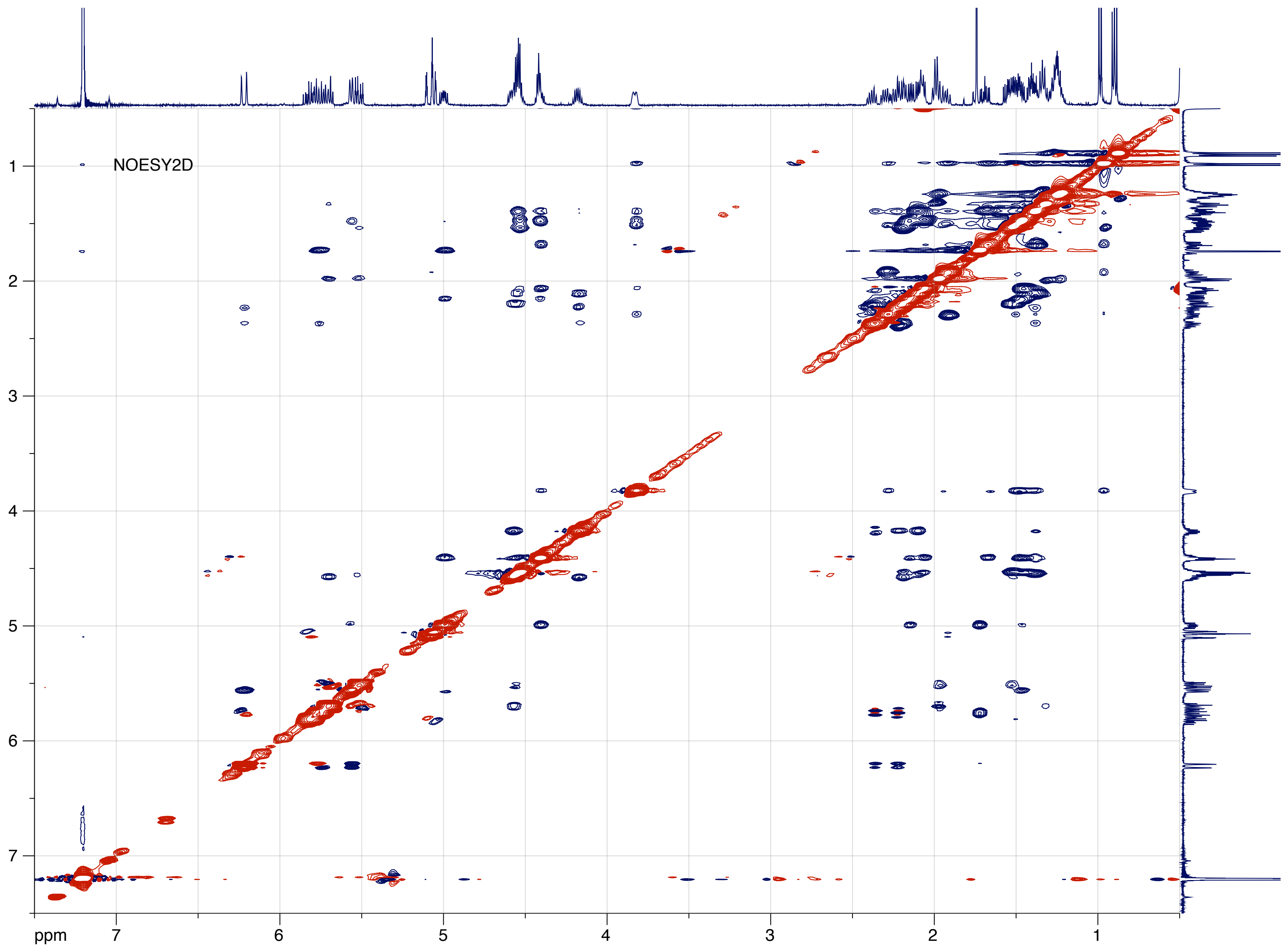
NOESY2D
ppm
7
6
5
4
3
2
1
1
2
3
4
5
6
7
